# Fluid balance neutralization secured by hemodynamic monitoring versus protocolized standard of care in critically ill patients requiring continuous renal replacement therapy: study protocol of the GO NEUTRAL randomized controlled trial

**DOI:** 10.1186/s13063-022-06735-6

**Published:** 2022-09-22

**Authors:** Laurent Bitker, Pierre Pradat, Claire Dupuis, Kada Klouche, Julien Illinger, Bertrand Souweine, Jean-Christophe Richard

**Affiliations:** 1 Service de Médecine Intensive – Réanimation, Hôpital de la Croix Rousse, Hospices Civils de Lyon, Lyon, France; 2 Université Claude Bernard Lyon 1, Lyon, France; 3 Univ Lyon, INSA-Lyon, Université Claude Bernard Lyon 1, CNRS, Inserm, CREATIS UMR 5220, U1294, Villeurbanne, France; 4 Centre de Recherche Clinique, Hôpital de la Croix Rousse, Hospices Civils de Lyon, Lyon, France; 5 Service de Médecine Intensive – Réanimation, hôpital Gabriel Montpied, Clermont Ferrand, France; 6 Service de Médecine Intensive – Réanimation, Hôpital Lapeyronnie, Montpellier, France; 7 Service de Médecine Intensive – Réanimation, hôpital Nord-Ouest, Villefranche sur Saône, France

**Keywords:** Acute kidney injury, Critical care, Hemodynamic monitoring, Fluid balance, Net ultrafiltration, Renal replacement therapy

## Abstract

**Background:**

Fluid overload is associated with worse outcome in critically ill patients requiring continuous renal replacement therapy (CRRT). Net ultrafiltration (UF_NET_) allows precise control of the fluid removal but is frequently ceased due to hemodynamic instability episodes. However, approximately 50% of the hemodynamic instability episodes in ICU patients treated with CRRT are not associated with preload dependence (i.e., are not related to a decrease in cardiac preload), suggesting that volume removal is not responsible for these episodes of hemodynamic impairment. The use of advanced hemodynamic monitoring, comprising continuous cardiac output monitoring to repeatedly assess preload dependency, could allow securing UF_NET_ to allow fluid balance control and prevent fluid overload.

**Methods:**

The GO NEUTRAL trial is a multicenter, open-labeled, randomized, controlled, superiority trial with parallel groups and balanced randomization with a 1:1 ratio. The trial will enroll adult patients with acute circulatory failure treated with vasopressors and severe acute kidney injury requiring CRRT who already have been equipped with a continuous cardiac output monitoring device. After informed consent, patients will be randomized into two groups. The control group will receive protocolized fluid removal with an UF_NET_ rate set to 0–25 ml h^−1^ between inclusion and H72 of inclusion. The intervention group will be treated with an UF_NET_ rate set on the CRRT of at least 100 ml h^−1^ between inclusion and H72 of inclusion if hemodynamically tolerated based on a protocolized hemodynamic protocol aiming to adjust UF_NET_ based on cardiac output, arterial lactate concentration, and preload dependence assessment by postural maneuvers, performed regularly during nursing rounds, and in case of a hemodynamic instability episode. The primary outcome of the study will be the cumulative fluid balance between inclusion and H72 of inclusion. Randomization will be generated using random block sizes and stratified based on fluid overload status at inclusion. The main outcome will be analyzed in the modified intention-to-treat population, defined as all alive patients at H72 of inclusion, based on their initial allocation group.

**Discussion:**

We present in the present protocol all study procedures in regard to the achievement of the GO NEUTRAL trial, to prevent biased analysis of trial outcomes and improve the transparency of the trial result report. Enrollment of patients in the GO NEUTRAL trial has started on June 31, 2021, and is ongoing.

**Trial registration:**

ClinicalTrials.gov NCT04801784. Registered on March 12, 2021, before the start of inclusion.

**Supplementary Information:**

The online version contains supplementary material available at 10.1186/s13063-022-06735-6.

## Administrative information

Note: The numbers in curly brackets in this protocol refer to the SPIRIT checklist item numbers. The order of the items has been modified to group similar items (see http://www.equator-network.org/reporting-guidelines/spirit-2013-statement-defining-standard-protocol-items-for-clinical-trials/).

**Table:** 

Title {1}	Fluid balance neutralization secured by hemodynamic monitoring versus protocolized standard-of-care in critically ill patients requiring continuous renal replacement therapy. Study protocol of the GO NEUTRAL randomized controlled trial
Trial registration {2a and 2b}.	Registered on ClinicalTrials.gov under identifier NCT04801784 on March 12, 2021 before the start of inclusion
Protocol version {3}	Protocol version 1, March 3, 2021
Funding {4}	French Ministry of Health: Inter-regional Hospital Clinical Research Program 2019 (Programme Hospitalier de Recherche Clinique inter-régional 2019)
Author details {5a}	Laurent Bitker^1–3^, Pierre Pradat^4^, Bertrand Souweine^5^, Kada Klouche^6^, Julien Illinger^7^, Jean-Christophe Richard^1–3^ 1. Service de Médecine Intensive – Réanimation, hôpital de la Croix Rousse, Hospices Civils de Lyon, Lyon, France 2. Université Claude Bernard Lyon 1, Lyon, France 3. Univ Lyon, INSA-Lyon, Université Claude Bernard Lyon 1, CNRS, Inserm, CREATIS UMR 5220, U1294, Villeurbanne, France. 4. Centre de Recherche Clinique, hôpital de la Croix Rousse, Hospices Civils de Lyon, Lyon, France 5. Service de Médecine Intensive – Réanimation, hôpital Gabriel Montpied, Clermont Ferrand, France 6. Service de Médecine Intensive – Réanimation, hôpital Lapeyronnie, Montpellier, France 7. Service de Médecine Intensive – Réanimation, hôpital Nord-Ouest, Villefranche sur Saône, France
Name and contact information for the trial sponsor {5b}	Mr Alexandre Pachot Direction de la Recherche en Santé, Hospices Civils de Lyon, BP 2251, 3 Quai des Célestins, 69229 LYON Cedex 02. Tel: + 33 4 72 40 68 52.
Role of sponsor {5c}	The study sponsor and funder had no part in the study design; collection, management, analysis, and interpretation of the data; writing of the report; and in the decision to submit the report for publication. They had no ultimate authority over any of these activities.

## Introduction

### Background and rationale {6a}

Critical illness-associated fluid overload, defined as a body weight increase above 10% of premorbid body weight, is frequent in the critically ill population and reaches a prevalence of 25% in patients with acute kidney injury requiring renal replacement therapy (RRT) due to reduced urine output and higher severity of disease [1, 2]. A positive fluid balance is associated with an increased risk of death in patients admitted to the intensive care unit (ICU) [3]; strategies aiming to control fluid balance have demonstrated effectiveness in shortening mechanical ventilation duration or improving renal recovery in critically ill patients without RRT [4, 5].

In patients treated with RRT, net ultrafiltration (UF_NET_) is an efficient modality to remove excessive extracellular fluid from the body [6]. However, disproportionate net ultrafiltration may induce a state of preload dependence and decreased cardiac output, which will in turn impair organ perfusion and potentially patient outcome [7, 8]. Indeed, hemodynamic instability episodes are frequent in this population; yet, in a single-center observational study performed in patients treated with continuous renal replacement therapy (CRRT), approximately 50% of the hemodynamic instability episodes were not associated with a preload dependent status (i.e., were not related to a decrease in cardiac preload), as identified by a positive postural maneuver (passive leg raising or Trendelenburg maneuver) [9]. Also, a positive preload dependence test at RRT start was associated with an increased risk of arterial hypotension during intermittent RRT [10]. Furthermore, hemodynamic instability episodes will frequently lead to UF_NET_ cessation, which will in turn further deteriorate fluid balance control. On the contrary, if UF_NET_ could be maintained despite hemodynamic impairment unrelated to volume depletion, improved fluid balance control could foster positive effects in terms of fluid overload prevention and organ failure recovery and patient outcomes [11–13].

Also, large observational registries have shown mixed results regarding UF_NET_ intensity and its association with patients’ outcome, with one showing an improved survival in case of high UF_NET_ (> 25 ml kg^−1^ day^−1^), while the other observed decreased survival in the group of patients with a UF_NET_ > 40 ml kg^−1^ day^−1^ [14, 15]. Taken together, these data suggest that a UF_NET_ strategy not tailored to the patient’s physiological characteristics may be deleterious and should motivate the use of tools to individualize and optimize UF_NET_ based on improved hemodynamic assessment.

Modern hemodynamic monitoring device which allows calibrated cardiac output assessment and continuous cardiac output monitoring now offers the opportunity to individualize UF_NET_ based on its consequences on systemic hemodynamics [16]. We hence make the hypothesis that a strategy targeting a significant UF_NET_ rate secured by a multi-modal hemodynamic protocol will allow fluid balance neutralization over the course of the first 72 h of CRRT in ICU patients treated with vasopressors.

### Objectives {7}

The main objective of the study is to assess the impact of fluid removal with net ultrafiltration ≥ 100 ml h^−1^, secured by an advanced hemodynamic protocol (neutral fluid balance strategy, intervention group) on the cumulative fluid balance measured over the first 72 h following inclusion in critically ill patients requiring CRRT and vasopressors, as compared to the current standard of care (UF_NET_ 0–25 ml h^−1^, standard care, control group).

#### Safety secondary objectives

1. To assess the hemodynamic safety of the intervention strategy, compared to that of the control strategy, by comparing the number of hemodynamic instability episodes observed during the first 72 h following inclusion in each study group
2. To describe the underlying mechanisms of hemodynamic instability episodes, by classifying them as being preload dependent or preload independent and comparing the rate of preload-dependent hemodynamic instability episodes observed during the first 72 h following inclusion in each study group
3. To evaluate the impact of the intervention strategy on main hemodynamic determinants (mean arterial pressure, cardiac index, central venous pressure, arterial lactate concentration, administered dose of vasopressors) over the first 72 h following inclusion, compared to the control group
4. To evaluate the impact of the intervention strategy on vasopressor-free days at day 28 of study participation, by comparing the number of days alive without vasopressors in the two study groups and censored at day 28 of participation
5. To assess the effects of the intervention strategy on organ failure severity, as compared to the control group, using the hemodynamic and total Sepsis-related Organ Failure Assessment (SOFA) score measured daily from inclusion to H72 in the two study groups [17]
6. To evaluate the effect of the intervention strategy on H72, day 28, and day 90 mortality, as compared to the control strategy

#### Efficacy secondary objectives

7. To assess the impact of the intervention strategy on the cumulative fluid balance (normalized to the observation period) over the first 24 h, 72 h, and 7 days following inclusion, respectively, as compared to the control strategy
8. To assess the impact of the intervention strategy on the cumulative net ultrafiltration (normalized to the observation period) over the first 24 h and 72 h following inclusion, respectively, as compared to the control strategy
9. To evaluate the effectiveness of the intervention to prevent or limit fluid overload-related hypoxemic respiratory failure, by comparing the arterial O_2_ partial pressure (PaO_2_) to O_2_ inspired fraction (FiO_2_) ratio on the one side, and the extravascular lung water index on the other, in the two study groups, between inclusion and H72 after inclusion
10. To evaluate the impact of the intervention strategy on ventilatory-free days at day 28 of study participation, by comparing the number of days alive without mechanical ventilation in the two study groups and censored at day 28 of participation
11. To evaluate the effect of the intervention strategy on long-term renal outcome, by comparing the rate of the composite outcome of major adverse kidney event (MAKE) evaluated at day 90 of inclusion in the two study groups. MAKE-90 comprises death, RRT dependence, or persistent stage 2 or 3 acute kidney injury at day 90 of inclusion [6]
12. To assess the ability of the intervention strategy to decrease hospital and intensive care length of stay, as compared to the control strategy

#### Feasibility secondary objectives

13. Rate of participant inclusion over time to evaluate the feasibility of a larger randomized controlled trial with a patient-centered primary outcome

### Trial design {8}

The GO NEUTRAL study is a multicenter, open-labeled, randomized, controlled, superiority trial with parallel groups and balanced randomization with a 1:1 ratio. The first version of the protocol was published on March 3, 2021, before the first inclusion (Supplemental Material 1, French version). The WHO Trial Registration Data Set is provided in Supplemental Material 2.

## Methods: participants, interventions, and outcomes

### Study setting {9}

The study is conducted in four ICUs located in both academic and non-academic French hospitals. The list of participating centers is presented in Supplemental Materials 3, Supplemental Table 1.

### Eligibility criteria {10}

#### Inclusion criteria

- Patients aged 18 years or older, affiliated to social security as per French regulation
- Requiring treatment by continuous intravenous infusion of epinephrine or norepinephrine for acute circulatory failure
- Presenting with stage 3 acute kidney injury as per the Kidney Disease: Improving Global Outcome guidelines [6]
- Treated for less than 24 h with CRRT at time of eligibility evaluation
- Equipped with a continuous cardiac output monitoring device using real-time arterial pulse contour analysis (PiCCO®, Pulsion Medical Systems, Feldkirchen, Germany), already in place at the time of eligibility evaluation

#### Exclusion criteria

- Patient under extra-corporeal membrane oxygenation
- Patient with active hemorrhage and receiving blood transfusion
- Patient under chronic maintenance dialysis or renal graft recipient
- Switch to intermittent RRT scheduled in the 72 h following inclusion
- Ischemic or hemorrhagic cerebral stroke complicated with coma and under mechanical ventilation
- Fulminant hepatitis, defined as the coexistence at time of eligibility evaluation of acute liver damage with hepatic encephalopathy, icterus, and decrease in prothrombin ratio < 50% in less than 15 days
- Contra-indications to postural maneuvers to assess preload dependence, such as lower limb amputations, inferior vena cava obstruction, and abdominal compartment syndrome
- Pregnancy or ongoing breastfeeding
- Withholding of life support decision regarding mechanic ventilation or resuscitation of cardiac arrest
- Moribund patient (expected to die in the next 12 h)
- Patient under legal protective measures
- Inclusion in another trial whose primary outcome would be fluid balance or whose intervention would impact hemodynamic, RRT settings, or modifying the fluid balance
- Patient already enrolled in the study

### Who will take informed consent? {26a}

Before inclusion in the trial, written informed consent of the patient will be sought by investigators. In case the patient is unable to receive information and give consent, informed consent will be sought from its legal representative. If the patient or his legal representative cannot be present at the time of eligibility, due to potential visiting restrictions related to the pandemic, a procedure for emergent inclusion of the patient will be used, and study participation approval from the patient or his legal representative will be sought later. In any case, patient’s written informed consent will be sought as soon as its medical condition will allow it.

### Additional consent provisions for collection and use of participant data and biological specimens {26b}

Potential future studies intending unplanned use of the data generated in this trial will require an additional consent of included patients. Unplanned use of biological specimens will not be performed.

## Intervention

### Explanation of the choice of comparators {6b}

The control group will be managed as per international guidelines regarding net ultrafiltration management in critically ill patients with acute circulatory failure and will reflect the current standard of care as reported by a large randomized controlled trial [18, 19]. The control group is hence characterized by a protocolized UF_NET_ rate set between 0 and 25 ml h^−1^ as a mean to limit or prevent the risk it contaminating the intervention arm of the study.

### Intervention description {11a}

#### Intervention strategy

Within 2 h after inclusion, the net ultrafiltration rate of the intervention group will be set to 100 ml h^−1^ or more (based on the clinician-in-charge decision and without exceeding 400 ml h^−1^) and maintained as such for the next 72 h. The targeted net ultrafiltration rate will aim to compensate the fluid input received by the patient over the same period of time and was estimated based on daily fluid input of 2500 ml reported in the IDEAL-ICU trial [18]. An UF_NET_ rate of 100 ml h^−1^ corresponds to a weight-corrected UF_NET_ rate of 1.2 ml kg^−1^ h^−1^ in an 80-kg patient, which was associated with improved outcome in a large observational study [14]. After H72 following inclusion, the UF_NET_ rate will be left at the discretion of the treating clinician.

In the intervention group, a hemodynamic protocol will allow the decrease or the cessation of the UF_NET_ based on the definition of threatening hemodynamic profiles described in SM3, Supplemental Fig. 1. In summary, the first part of the hemodynamic protocol uses a “routine” systematic assessment performed during nursing rounds every 4 h and comprising calibrated cardiac index values, arterial lactate levels, central venous pressure, and the results of a postural maneuver assessing preload dependence. Based on these hemodynamic elements, the protocol aims to maintain a UF_NET_ rate of 100 ml h^−1^ or more (green profiles, letters A, B, and D in the Protocol Figure) or half it (orange profiles, letters C, E, F, and I) or cease it (red profile, letters G and H) in case of threatening hemodynamic phenotypes. The hemodynamic profiles will be re-assessed at each nursing round, and the UF_NET_ rate re-adjusted accordingly. In the intervention arm, arterial lactate concentrations will be measured 8-hourly during the 72 h of the intervention period.

The postural maneuvers consist of a passive leg raising test in supine patients or a Trendelenburg maneuver in prone patients, using validated thresholds to classify patients as being preload dependent [20, 21]. A postural maneuver will be performed in both study groups at the time of inclusion and performed as part of the hemodynamic monitoring protocol in the intervention arm. Quality control criteria of postural maneuvers are defined by the study protocol and will be communicated/reminded to investigators and nursing staff. The quality control criteria comprise the correct position of arterial and central venous pressure captors at the phlebostatic point, recent calibration of the cardiac output monitoring device (< 1 h), proper installation of the patient prior to the maneuver (passive leg raising [supine patients]: semi-recumbent position > 45°; Trendelenburg [prone patients]: + 13° positive Trendelenburg position), correct completion of the maneuver (passive leg raising [supine patients]: rapid tilt to 0° of the trunk and passive leg raising > 45°; Trendelenburg [prone patients]: rapid tilt to −13° of the bed position), and maneuver duration of 1 min max during which the highest continuous cardiac index value is collected by the operator. The passive leg raising maneuver is deemed positive (preload dependence) if the continuous cardiac index increases > 10% of the pre-maneuver value. The Trendelenburg maneuver is deemed positive (preload dependence) if the continuous cardiac index increases > 8% of the pre-maneuver value. Postural maneuvers to judge preload dependence require real-time measurements of cardiac output to detect short-term changes in cardiac index. The device used in the trial is pulse contour analysis of the arterial femoral wave form by means of the PiCCO® device (Pulsion Medical Systems, Feldkirchen, Germany) after calibration of cardiac output by the thermodilution method.

The second part of the protocol aims to re-assess the UF_NET_ rate in case of a hemodynamic instability episode (as described above). In this case, the protocol was simplified, using only the results of a postural maneuver, the CVP value, or the clinician’s judgment to decrease or cease UF_NET_ (SM3, Supplemental Fig. 2). The UF_NET_ rate will then be re-adjusted at the next scheduled nursing round based on the protocol.

#### Control strategy

Within 2 h after inclusion, the net ultrafiltration rate of the control group will be set between 0 and 25 ml h^−1^ (based on the clinician-in-charge decision) and maintained as such for the next 72 h. This net ultrafiltration rate aims to reflect the current standard of care received by patients in a large randomized controlled trial evaluating the timing of RRT in the ICU [18]. After H72 of inclusion, the UF_NET_ rate will be left at the discretion of the treating clinician. No hemodynamic protocol will be applied in the control arm, and the management of hemodynamic instability episodes will be left at the treating clinician discretion. Hemodynamic variables and hemodynamic instability episodes are reported by the intervention group. In case of an episode of acute hypoxemic respiratory failure related to fluid overload (based on strict transthoracic echocardiography criteria), the clinician-in-charge will be allowed to increase transiently the UF_NET_ rate above 20 ml h^−1^ until normalization of the clinical situation. Acute hypoxemic respiratory failure due to fluid overload will be defined as the conjunction of rapid inset of tachypnea (respiratory rate > 25 bpm) with aggravating hypoxemia, ultrasounds or X-ray proof of de novo pulmonary interstitial infiltrates, and at least one of the following transthoracic echocardiography criteria: mitral Doppler E-wave > 1.5 times A-wave in patients > 65 years, E-wave deceleration time > 150 ms, E-wave maximum velocity > 1 m s^−1^, or an E-wave to spectral tissue Doppler at the medial mitral annulus velocity (E′)-wave ratio > 12.

### Criteria for discontinuing or modifying allocated interventions {11b}

Changes in UF_NET_ based on hemodynamic and/or respiratory evaluations as per each study group are described above.

Temporary contra-indications to the study procedures are:

- Transient suspension of RRT for less than 8 h due to patient transfer to the operating room or the imaging facility
- Occurrence of active hemorrhage requiring blood transfusion and whose resolution is expected in less than 8 h
- Transient contra-indication to a postural maneuver to assess preload dependence, such as lower limb immobilization or abdominal compartment syndrome, if they last less than 8 h

Permanent contra-indications to study procedures are:

- Consent removal or refuse to participate after an inclusion following the emergent procedure
- Permanent suspension of RRT for more than 8 h, whatever the reason
- Acute ischemic or hemorrhagic stroke complicated with coma and requiring mechanical ventilation
- Occurrence of active hemorrhage requiring blood transfusion and whose resolution is not expected in less than 8 h
- Permanent contra-indication to a postural maneuver to assess preload dependence, such as lower limb amputation or abdominal compartment syndrome, if they last more than 8 h
- Continuous cardiac output monitoring is no longer feasible due to irreversible technical issues
- Transfer to a non-participating ICU

Patients could be withdrawn from the study at their request or the request of their legal representative, and their data will not be analyzed. In case of harm related to the study procedures identified as such by the treating clinician, patients will be managed outside the protocol, but will be left in their allocated study arm.

### Strategies to improve adherence to interventions {11c}

Adherence to study interventions will be checked daily by the investigators during staff with the clinicians. The study procedures will be provided to the investigators and to the bedside staff.

### Relevant concomitant care or prohibited during the trial {11d}

#### Fluid resuscitation

Fluid resuscitation is authorized in both study groups. Clinicians will be recommended to use fluid bolus therapy based on the identification of a decrease in cardiac output associated with preload dependence, following international guidelines [22]. In the intervention group, fluid bolus therapy will be used to discriminate patients with low cardiac output, elevated lactate, and a negative postural maneuver based on the results of a fluid challenge with calibrated cardiac output.

#### Vasopressor therapy management

Vasopressors (and inotropes) will be introduced and titrated as per local ICU protocols, based on the target mean/systolic arterial pressures defined by the treating clinician. Clinicians will be recommended to systematically perform a hemodynamic assessment of the patient in case of occurrence of a hemodynamic instability episode, prior to vasopressor dose adjustment [16]. The dose of vasopressors and inotropes will be reported in the case report form.

#### Renal replacement therapy management

Patients will be receiving CRRT at the time of enrollment. Clinicians will be recommended to pursue a CRRT technique until 72 h of inclusion and will be free to switch to an intermittent method after that time. Of note, international and French guidelines recommend the use of CRRT in hemodynamically unstable patients [6, 23]. RRT modality (diffusion or convection, or a mix of both) and their settings will be left at the discretion of the treating clinician, apart from UF_NET_ during the 72 h of the study intervention.

#### Diuretic management

Clinicians will be recommended to cease diuretic administration in enrolled patients treated with RRT. The Kidney Disease: Improving Global Outcome (KDIGO) guidelines state that diuretics are probably not recommended to accelerate renal recovery or RRT weaning [6].

### Provisions for post-trial care {30}

None.

### Outcomes {12}

#### Primary outcome

The primary outcome is the cumulative fluid balance measured between inclusion and 72 h after inclusion in alive patients at H72, computed as the difference between fluid input and output, quantified in milliliters over the same period. The primary outcome will be assessed in the modified intention-to-treat (ITT) population (see below for the definition of study populations).

Fluid input will be defined as any intravenous (either intermittently or continuously administered) or orally administered drugs or electrolytes diluted into a volume, enteral or parenteral nutrition, fluid bolus therapy, intravenous maintenance hydration, or blood products. Fluid output will comprise urine output, UF_NET_, and surgical drain output. Hence, some unquantifiable input or output will not be measured such as subcutaneous injections, perspiration, or stool volume. The input and output volumes composite of the primary outcome will be collected during nursing rounds as part of routine care.

#### Safety secondary outcomes (study population is given in brackets)

1. The number of hemodynamic instability episodes observed in both study groups between inclusion and 72 h after inclusion or death, whichever comes first. Hemodynamic instability is defined as the occurrence of de novo tachycardia (heart rate > 120 bpm), de novo hypotension (systolic or mean arterial pressure below clinician-defined target and requiring hemodynamic resuscitation), de novo or extension of mottles, or de novo decrease in cardiac output (negative change in cardiac index > 15%) [*ITT population*].
2. The number of hemodynamic instability episodes (as defined above) associated with preload dependence (identified by a significant and positive increase in cardiac output following a postural maneuver), observed in both study groups between inclusion and 72 h after inclusion or death, whichever comes first [*ITT population*].
3. Mean arterial pressure values, cardiac index, central venous pressure, arterial lactate concentration reported every 4 h, and the vasopressor administered dose reported every 24 h, in both groups, between inclusion and 72 h after inclusion or death, whichever comes first [*ITT population*].
4. The number of vasopressor-free days in both study groups, quantified as the number of days alive without vasopressors, starting at the time of inclusion and censored at day 28 of inclusion. Vasopressor weaning will be defined if vasopressors are not required for a continuous period of 48 h or longer. A value of 0 is allocated if the patients died between inclusion and day 28. A value of 0 is also allocated if the patient is still receiving vasopressors at day 28 [24]. In case of multiple vasopressor weaning episodes, only the last weaning attempt will be considered [*ITT population*].
5. Organ failure severity evaluated by the total SOFA score, composed of the sum of the 6 organ-by-organ subscores (neurologic, respiratory, hemodynamic, hepatic, hematologic, and renal), and the hemodynamic SOFA subscore, in both study groups. SOFA subscores are allocated a value between 0 (no failure) and 4 (highest degree of failure) and will be collected once a day from inclusion to H72 or death, whichever comes first [17] [*ITT population*].
6. Vital status at H72, day 28, and day 90 after inclusion, collected from electronic medical files and hospital databases [*ITT population*].

#### Efficacy secondary outcomes (study population is given in brackets)

7. Normalized cumulative fluid balance measured in both groups at H24 and H72 after inclusion or death, whichever comes first, and at day 7 in alive patients. Fluid balance measured between inclusion and early time point (H24 or H72 or death) will be normalized by dividing it by the observation period duration and expressed in ml h^−1^. The fluid balance at H24 and H72 will be quantified using the same input and output items as per the primary outcome. The fluid balance at day 7 will be estimated based on the change in body weight between inclusion and day 7 [*ITT population*].
8. Normalized cumulative UF_NET_ volume in both study groups at H24 and H72 after inclusion or death, whichever comes first. Cumulative UF_NET_ measured between inclusion and H24 or H72 or death will be normalized by dividing it by the observation period duration and expressed in ml h^−1^. The cumulative UF_NET_ volume will be computed based on the UF_NET_ volumes reported every 4 h between inclusion and 24 or 72 h after inclusion or death, whichever comes first [*ITT population*].
9. PaO_2_ to FiO_2_ ratio and the extravascular lung water index measured with the cardiac output monitoring device, measured once a day from inclusion to H72 or death, whichever comes first. The FiO_2_ is the FiO_2_ set on the respirator or the high-flow nasal cannula device. In non-ventilated patients, the FiO_2_ will be estimated using the following formula: FiO_2_ = 0.21 + 0.03 × oxygen flow rate (in L min^−1^) [25] [*ITT population*].
10. The number of ventilator-free days in both study groups, quantified as the number of days alive without mechanical ventilation, starting at the time of inclusion and censored at day 28 of inclusion. Mechanical ventilation weaning will be defined if invasive mechanical ventilation is not required for a continuous period of 48 hours or longer. A value of 0 is allocated if the patients died between inclusion and day 28. A value of 0 is also allocated if the patient is still receiving invasive mechanical ventilation at day 28 [24]. In case of multiple mechanical ventilation weaning episodes, only the last weaning attempt will be considered [*ITT population*].
11. MAKE-90 assessed between inclusion and day 90 after inclusion in both groups. MAKE-90 comprises death before or at day 90, RRT dependence at day 90, and persistent stage 2 or 3 acute kidney injury (as per the KDIGO guidelines) at day 90. RRT dependence is present if the patient is still receiving CRRT at day 90 or if he has received an intermittent technique in the time frame of ±2 days around day 90. Persistent acute kidney injury is adjudicated based on a baseline plasma creatinine concentration (identified in the 6 months to 7 days before ICU admission), and the plasma creatinine measured at day 90 [6]. In case of missing baseline creatinine value, it will be retrospectively estimated [26]. Each element of the composite event will be evaluated separately [*ITT population*].
12. Hospital and ICU length of stay, quantified as the duration, in days, between inclusion and hospital and ICU discharge (alive or deceased), respectively [*ITT population*].

#### Feasibility secondary outcomes (study population is given in brackets)

13. The number of eligible patients per month and per participating center and the number of patients effectively enrolled in the trial per month and per participating center. [N/A]

### Participant timeline {13}

Participants’ timeline is shown in SM3, Supplemental Fig. 3, and the exact steps prior to and during enrollment are given in SM3, Supplemental Table 2.

### Sample size {14}

We make the hypothesis that the fluid balance will be of 4000 ± 4000 ml at H72 of inclusion in the control group and that the intervention strategy will generate a fluid balance of 0 ± 4000 ml at H72 of inclusion (absolute between groups difference of 4000 ml). The hypothesis is based on the data reported by a large randomized controlled trial evaluating the timing of RRT in the ICU, in which the H72 cumulative fluid balance was 3711 ml in the early arm and 3917 ml in the delayed arm of the study [18]. Based on this hypothesis, the required sample size to compare two means between two equally sized samples (and a bilateral hypothesis), with a *⍺* of 0.05 and a power 1–*β* of 0.8, would be 16 patients per arm, hence a total of 32 patients. Since (1) we expect a non-survival rate of 25% at 72 h of inclusion (corresponding to the time point of the primary outcome evaluation) and (2) the expected distribution of the primary outcome would not follow the normal distribution, justifying an increase of 25% in sample size, the final total number of patients to enroll would be 58, equally distributed between study groups. Patients deceased before 72 h of inclusion, or alive at H72 but with a missing primary outcome measure, will not be included in the analysis of the primary outcome. Once the required number of 21 patients per arm with an assessable primary outcome will be reached, inclusions will cease.

### Recruitment {15}

The participating ICUs are dedicated to the care of severely ill patients such as those fulfilling the inclusion criterion. All have experience in advanced hemodynamic monitoring, used in routine care by the nursing staff. The application of the hemodynamic assessment and determination of profiles takes less than 5 min every 4 h. Also, in an observational study assessing the causes of hypotension in critically ill patients requiring CRRT, the enrollment rate of the coordinating center was between 1 and 2 per month over the study period (12 to 24 enrolled patients per year) [9].

In order to achieve recruitment, all the clinicians of the participating ICU will receive a detailed information about the study. All patients treated with CRRT will be screened daily to evaluate study eligibility. To ensure an adequate number of participants will be enrolled in the required time frame, the participating centers will be asked to report on a regular basis their problems related to enrollment, in order to find adequate responses to improve the enrollment rate. Additional centers will be sought in case the enrollment rate in the trial is too low. Finally, a 1-year extension of the study time frame will be proposed in case the enrollment rate in the trial is too low.

## Assignment of interventions: allocation

### Sequence generation {16a}

Allocation sequence will be computer-generated with stratification based on the fluid overload status at the time of enrollment. Fluid overload will be defined as a 10% increase in body weight between ICU admission and trial enrollment [2]. Fluid overload is a known risk factor for ICU mortality. Randomization will be performed in each stratum, with a 1:1 ratio, and using a fixed block size. The block size will only be known by the statistician in charge of the randomization list.

### Concealment mechanism {16b}

Allocation concealment will be ensured via a central web-based system (Ennov Clinical® 7.5.720). The treatment to which a patient will be allocated will be disclosed only after enrollment in the study.

### Implementation {16c}

The randomization key will only be known to the biostatistician. Investigators at each study site will be responsible for patient enrollment in the study. Assignment of participants to each study group will be ensured by the central web-based system (Ennov Clinical® 7.5.720®) operated by local investigators, after verification of patient eligibility and inclusion in the study.

## Assignment of interventions: blinding

### Who will be blinded {17a}

Blinding of care providers will be unpracticable as knowledge of the UF_NET_ settings is required to adapt care and apply tested strategies and the hemodynamic protocol. Outcome assessors will not be blinded as group allocation could be deducted from UF_NET_ settings provided in the electronic medical files. Data analysts will be blinded to group allocation, although this may be deducted from the reported fluid balance measurements and applied UF_NET_ rates.

### Procedures for unblinding if needed {17b}

Due to the open design of the study, there is no unblinding procedure. After recording the main outcome criterion of the last included patient into the case report form, a quality control will be performed on the database with blinding of the study arm. Statistical analyses will begin after the database lock.

## Data collection and management

### Plans for assessment and collection of data {18a}

Investigators are responsible for the assessment and collection of outcomes, baseline, and other trial data. To improve the quality of data collected in regard to the hemodynamic protocol and its application every 4 h, a bedside case report form specific to the study arm will be made available, directly downloadable, and printable from the central web-based system Ennov Clinical® 7.5.720 after patient enrollment (Supplemental Materials 4 [intervention group] and 5 [control group], French versions). A laminated print of the hemodynamic protocol of the intervention group will be made available at the bedside of enrolled patients allocated to this group. Data will be entered in the electronic case report form by delegated team members and will be monitored by trained clinical research assistants designated by the sponsor. The digitalized version of the case report form is provided in Supplemental Material 6 (French version). Subjects will be assessed daily while hospitalized in the ICU.

Day 28 and day 90 assessments will be performed by investigators or delegated team members using (in ranked order): electronic medical records, phone calls to the patient’s general practitioner, phone calls to any medical doctor involved in the patient’s care after ICU discharge, and phone calls to the patients or his next-of-kin. A prescription to perform a plasma creatinine concentration measurement at day 90 will be sent by mail to ICU survivors weaned of RRT during their ICU stay, and its results communicated to the general practitioner as well as to the investigators to compute the MAKE-90 secondary outcome. Control of long-term renal function after acute kidney injury is recommended by experts [27].

### Plans to promote participant retention and complete follow-up {18b}

Incomplete follow-up during ICU stay is not expected, given the studied population. Given that most outcomes (hemodynamic and physiologic monitoring, CRRT settings including UF_NET_, vasopressor, and mechanical ventilation and their weaning date) are specific to the ICU environment and stay of the participants, we do not expect missing values. Especially, input and output components of the primary outcome measure are reported every 4 h in the electronic ICU medical records and charts, either automatically or by the nursing staff at the bedside.

The procedure regarding the collection of the input and output components of the primary outcome and the procedure in case of missing values are detailed in SM3, Supplemental Table 3.

Missing values at day 28 or day 90 (vital status, day 90 creatinine, RRT weaning date) could occur for surviving patients after hospital discharge. Upon enrollment, patients’ and their next-of-kin contact information will be stored in the digital health record at each study site. Data regarding the vital status and RRT at day 28 or day 90 of inclusion will be retrieved following the ranked procedure described above.

### Data management {19}

An electronic case report form will be created for each included patient using the central web-based system Ennov Clinical® 7.5.720. Subjects will be identified using the first letter of their first name, the first letter of their family name, the center identifier, and the inclusion number. This code will be the only information enabling a retrospective link to the patient. The data collected during the study will be processed electronically in accordance with the requirements of the French Data Protection Authority (CNIL) in compliance with French Reference Methodology MR001. The electronic case report form will be transmitted electronically and centralized in the data management department of the coordinating site. To ensure correct data entry, all data cells of the electronic case report form will be constrained in terms of the number of digits, number of decimal numbers, and range of acceptable values.

### Confidentiality {27}

Subject confidentiality is strictly held by the participating investigators, their staff, the sponsor, and their agents. This confidentiality is extended to cover clinical information relating to subjects, test results of biological samples, and all other information generated during participation in the study. All electronic transmission of data that leaves each study center will be identified only by a unique study identifier that is linked to a subject through a code key maintained at the clinical site, and eventually destroyed at the end of the study. All source records including electronic data will be stored in secured systems.

No identifiable information concerning enrolled subjects will be released to an unauthorized third party. Subject confidentiality will be maintained when the study results are published or presented in conferences. The study monitor, other authorized representatives of the sponsor, and representatives of regulatory agencies may inspect all documents and records required to be conserved by the investigator.

### Plans for collection, laboratory evaluation, and storage of biological specimens for genetic or molecular analysis in this trial/future use {33}

Not applicable. No biological specimen will be collected and stored in this trial. All biological assays reported in the case report forms are those performed in the usual care of critically ill patients with acute circulatory failure and requiring RRT.

## Statistical methods

### Statistical methods for primary and secondary outcomes {20a}

#### General comments and descriptive analysis

All analyses will be performed using the R software for statistical computing (R Core Team). A *p* value < 0.05 will be considered significant.

Patients’ characteristics at enrollment will be reported in each study group to help evaluate allocation balance. Continuous variables will be reported using median and interquartile range (with minimum and maximum values) and compared between the groups using the Mann-Whitney test. Categorical variables will be reported using count and percentage (including missing values) and compared between the groups using the chi-squared test or Fisher exact test, as appropriate. Missing value count and percentage of each reported variable will be given.

#### Primary outcome analysis

The primary outcome measure will be reported with median and interquartile range (with minimum and maximum values) in each study group and compared between groups using Mann-Whitney’s test in the modified ITT population (see section for definition).

#### Analysis of the secondary outcomes

All secondary outcome measures will be analyzed in the ITT population (see section for definition). The following outcomes will be reported with median and interquartile range (with minimum and maximum values) in each study group and compared between the groups using Mann-Whitney’s test:

- Vasopressor-free day (secondary outcome #4)
- Normalized cumulative fluid balance (secondary outcome #7)
- Normalized cumulative UF_NET_ (secondary outcome #8)
- Ventilator-free day (secondary outcome #10)
- Hospital/ICU length of stay (secondary outcomes #12)

The secondary outcomes listed below will be reported longitudinally over time (from inclusion to H72) in the ITT population and compared between the study groups using mixed effects regression models with a fixed interaction of group by time, and a random effect with the patient identifier to account for the repetition of measurements, as well as an offset accounting for baseline values. In case of a significant interaction, a post hoc multiple comparison analysis between study groups will be performed, adjusted for the repetition of tests by the Tukey method.

- Number of hemodynamic instability episodes (secondary outcome #1)
- Number of hemodynamic instability episodes with preload dependence (secondary outcome #2)
- Hemodynamic parameters (secondary outcome #3)
- Total SOFA score and hemodynamic SOFA score (secondary outcome #5)
- PaO_2_/FiO_2_ ratio and extravascular lung water index (secondary outcomes #9)

The following categorical outcomes will be reported with count and percentage in each study group and compared between groups using the chi-squared or Fisher exact test: severe adverse events and adverse events of special interest, vital status (secondary outcome #6), and MAKE-90 (secondary outcome #11). Finally, no statistical test will be performed with secondary outcome #12. The hemodynamic profiles identified by the hemodynamic protocol of the intervention group will be reported in the supplemental results of the study.

### Interim analyses {21b}

No interim analysis of the primary outcome is planned, owing to the small sample size. However, given the severity of the disease of enrolled patients, we plan an interim safety analysis of the mortality rate at 72 h of inclusion in both study groups, after the enrollment of the first 20 patients.

### Methods for additional analyses (e.g., subgroup analyses) {20b}

Exploratory subgroup analyses regarding the primary outcome will be performed by comparing both arms of the study in the following subgroups of patients:

- Diagnosis of septic shock at the time of inclusion [28]
- SOFA score at inclusion strictly greater (>) or lower (≤) than the median value of enrolled participants [17]
- Fluid overload strictly greater (>) or lower (≤) than 10% of admission body weight at inclusion, as per the stratification [2]
- Cardiac index at inclusion strictly greater (>) or lower (≤) than 2.5 l min^−1^ m^−2^
- Arterial lactate concentration at inclusion strictly greater (>) or lower (≤) than 2 mmol l^−1^
- Preload dependence status (dependent or independent) at inclusion, identified by the postural maneuver
- Vasopressor dose at inclusion strictly greater (>) or lower (≤) than the median value of enrolled participants
- PaO_2_ to FiO_2_ ratio at inclusion greater (≥) or strictly lower (<) than the median value of enrolled participants

### Methods in analysis to handle protocol non-adherence and any statistical methods to handle missing data {20c}

The ITT population will consist of all enrolled patients, fulfilling eligibility criteria (inclusion and exclusion), analyzed as per their allocation group, regardless of their adherence to the protocol and the UF_NET_ rates they received, and followed until day 90 after inclusion, loss to follow-up, or death, whichever comes first. All secondary outcomes will be analyzed in the ITT population.

The modified ITT is a subset of the ITT population comprising all patients alive at 72 h of inclusion, fulfilling eligibility criteria (inclusion and exclusion), analyzed as per their allocation group, regardless of their adherence to the protocol and the UF_NET_ rates they received, and followed until day 90 after inclusion, loss to follow-up, or death, whichever comes first. This population hence will not include patients who died before H72 or those who ceased trial participation before H72. The primary outcome will be analyzed in the modified ITT population.

The rate of missing data per variable will be reported. No imputation of missing data is planned, and missing data will not be replaced.

### Plans to give access to the full protocol, participant-level data, and statistical code {31c}

After the publication of the trial’s results, the full study protocol, participant-level data, and statistical code will be made available upon reasonable request. Data access request will be reviewed by the trial steering committee.

## Oversight and monitoring

### Composition of the coordinating center and trial steering committee {5d}

The steering committee will be composed of coordinating center investigators LB and JCR, the study methodologist PP, and the project manager Ms Loredana Baboi. The steering committee will be responsible for all aspects of the trial, including communication with investigators, updating the protocol and submitting amendments, and verifying compliance to study procedures. The steering committee will meet weekly. There is no adjudication committee due to the nature of the primary outcome.

### Composition of the data monitoring committee, its role, and reporting structure {21a}

The study will be conducted in concordance with Good Clinical Practices and French regulation. The study does not declare a data and safety monitoring board as the intervention is considered as low risk and is applied in a population of patients at high risk of death, regardless of study procedures.

### Adverse event reporting and harms {22}

Due to the severity of the disease of the enrolled population, the list of reported adverse events was restricted to significant severe adverse events and adverse events of special interest. Adverse events are graded using the Common Terminology Criteria for Adverse Events (CTCAE) [29]. The list of reported severe adverse events, reported adverse events of special interest, and non-reported adverse events is given in SM3, Supplemental Table 4.

Significant severe adverse events were as follows (all with CTCAE grade ≥ 3): any kind of shock, worsening hemodynamic status, ventricular arrhythmia, myocardial infarction without ST elevation, hemorrhagic stroke, worsening respiratory status without mechanical ventilation, acute liver failure, gastro-intestinal stress ulcer, ischemic colitis, severe metabolic alkalosis, catheter-related bloodstream infection, ventilator-acquired pneumonia, documented bloodstream infection, and other documented hospital-acquired infections [29]. Significant severe adverse events will be systematically reported in the case report form in a daily manner, from inclusion to the patient’s end of participation.

Adverse events of special interest were death (CTCAE grade 5), hypovolemic non-hemorrhagic shock, successfully resuscitated cardiac arrest, myocardial infarction with ST elevation, ischemic stroke, hydrostatic acute pulmonary edema, mesenteric ischemia, acute limb ischemia, any other de novo organ ischemia, and any adverse events of grade 3 or more [29].

Given the severity of the disease of participants, some adverse events will be excessively frequent and will not be reported. Adverse events of special interest will be immediately reported to the study sponsor (Hospices Civils de Lyon), in a structured report form. Any adverse events associated with death during the study period are systematically and immediately declared and reported. Any unexpected severe adverse event will be reported to the French Regulatory Agency and the human research ethics committee.

All adverse events will be managed as per the best available standard of care. All severe adverse events will be reported in the final results of the trial.

### Frequency and plans for auditing trial conduct {23}

Trial audit, including audit of all enrolled participant data, will be performed by a dedicated auditing team designated by the study’s sponsor and independent from the study steering committee, investigators, and sponsor.

Trial audit will consist of verifying participants’ consent procedures and signed consent forms, verifying inclusion and exclusion criteria of enrolled participants, controlling the data collection of the primary outcome measure, controlling adverse event reporting, and reporting any major violation of study procedures. The auditing team will have full access to all required documents, including electronic medical records, in participating centers. Audit visits on the trial site will be performed per batch of 2 to 4 enrolled participants.

### Plans for communicating important protocol amendments to relevant parties (e.g., trial participants, ethical committees) {25}

In the case of protocol amendments, all modifications must be approved by the human research ethics committee that initially evaluated the trial (Comité de Protection des Personnes Sud-Méditerranée I) and the French Regulatory Agency (Agence Nationale de Sécurité du Médicament). After approval of protocol amendments, we will communicate them to investigators, participants, sponsor, and registry.

### Dissemination plans {31a}

The trial is currently registered on ClinicalTrials.org under the reference number NCT04801784. The authors intend to submit the trial’s final results to a peer-review journal within 12 months of the last patient’s end-of-participation date. Results of the trial will also be presented at national and international conferences. The investigators will follow the ICMJE rules for authorship [30].

## Discussion

To date, there is no randomized controlled trial evaluating the impact of protocolized UF_NET_ secured by advanced hemodynamic monitoring. The current protocol describes a randomized, controlled, open-label trial in parallel groups that aims to demonstrate the effectiveness of the hemodynamically secured UF_NET_ to maintain neutral the fluid balance of critically ill patients requiring CRRT, compared to protocolized standard of care.

The current study has been preceded by two observational studies led by the coordinating center, whose conclusions have led the investigators to identify a lower than expected rate of preload-dependent hemodynamic instability episodes in the critically ill population and to test the hypothesis that UF_NET_ may be secured if a protocolized hemodynamic monitoring is performed thoroughly, aiming to individualize care and optimize fluid balance control [9, 31].

The first identified limitation of the trial is the selection of an intermediate end point (cumulative fluid balance at H72 of inclusion) as the primary outcome measure. This was justified by the fact that proof of effectiveness of the hemodynamic protocol coupled with UF_NET_ adjustments needed to be evaluated on a small number of patients, and its value assessed on altering the physiological target (fluid balance) that could potentially impact patient-centered outcomes (extubation, ICU length of stay, renal recovery, survival). The trial will help design a larger randomized controlled trial whose primary outcome will be hard and patient-centered, as well as help continue refine and/or simplify the hemodynamic protocol to improve compliance to its application at a larger scale.

The second limitation of the study is the rareness of advanced hemodynamic monitoring in most French ICUs, which might limit (1) the generalizability of the trial’s results and (2) the potential development of a larger-scale randomized controlled trial. However, patient recruitment was optimized by enrolling academic and non-academic centers already involved in these novel monitoring techniques.

### Trial status

The GO NEUTRAL trial is currently enrolling patients. Enrollment of the first participant was on June 31, 2021. All four participating centers are open and are currently enrolling patients. The trial expected date of conclusion is June 31, 2023.

## Supplementary Information

**Additional file 1: Supplemental material 1**. Protocol version 1, March 3, 2021 (French).

**Additional file 2: Supplemental material 2**. WHO trial registry data set.

**Additional file 3: Supplemental material 3**. Study sites, intervention group hemodynamic protocols, participants timelines and study procedures, and list of adverse events.

**Additional file 4: Supplemental material 4**. Bedside case report form of the intervention group (French).

**Additional file 5: Supplemental material 5**. Bedside case report form of the control group (French).

**Additional file 6: Supplemental material 6**. Printed version of the electronic case report form (French).

**Additional file 7: Supplemental material 7**. Funding declaration (English).

**Additional file 8: Supplemental material 8**. Approval letter by the human research ethics committee CPP Sud-Méditéranée I, sent on April 29, 2021 (French and English versions).

## Data Availability

After the publication of the trial’s results, the full study protocol, participant-level data, and statistical code will be made available upon reasonable request. Data access request will be reviewed by the trial steering committee.

## Abbreviations

CRRT: Continuous renal replacement therapy
CTCAE: Common Terminology Criteria for Adverse Events
FiO_2_: O_2_ inspired fraction
ICU: Intensive care unit
KDIGO: Kidney Disease: Improving Global Outcome
MAKE-90: Major adverse kidney events at day 90
mITT: Modified intention-to-treat
PaO_2_: Arterial O_2_ partial pressure
RRT: Renal replacement therapy
SOFA: Sepsis-related Organ Failure Assessment
UF_NET_: Net ultrafiltration
