# Supplementary material for: Fluid balance neutralization secured by hemodynamic monitoring versus protocolized standard of care in critically ill patients requiring continuous renal replacement therapy: study protocol of the GO NEUTRAL randomized controlled trial
Source: Trials. 2022 Sep 22;23:798. doi: 10.1186/s13063-022-06735-6 (PMC9494882; doi:10.1186/s13063-022-06735-6)
Supplement: Supplementary file 1 — Additional file 1: Supplemental material 1. Protocol version 1, March 3, 2021 (French). [file 13063_2022_6735_MOESM1_ESM.pdf]

Neutralisation de la balance hydrosodée guidée par l'hémodynamique fonctionnelle au cours de l'épuration extra-rénale continue en réanimation

Etude GO-NEUTRAL

Protocole impliquant la personne humaine du 1° hors produit de santé

Version 1 du 03/03/2021

Promoteur :

Hospices Civils de Lyon
BP 2251
3 quai des Célestins,
69229 LYON cedex 02

Investigateur coordonnateur : Dr Laurent BITKER
Service de Médecine Intensive – Réanimation
Hôpital de la Croix Rousse/Groupement Hospitalier Nord
Hospices Civils de Lyon
103, Grande rue de la Croix Rousse, 69004 Lyon
Tél : 04.26.10.94.93; Fax : 04.72.07.17.74
Email : laurent.bitker@chu-lyon.fr

Code promoteur : 69HCL20_1243
Numéro d'enregistrement clinicaltrials.gov : *NCTXXXXX*
Avis favorable du CPP XX le :
Autorisation de l'ANSM le :

RESUME

**TITRE**	Neutralisation de la balance hydrosodée guidée par l'hémodynamique fonctionnelle au cours de l'épuration extra-rénale continue en réanimation<br>**Etude GO-NEUTRAL**
**PROMOTEUR**	Hospices Civils de Lyon<br>BP 2251<br>3 quai des Célestins,<br>69229 LYON cedex 02
**INVESTIGATEUR PRINCIPAL**	Dr Laurent BITKER<br>Service de Médecine Intensive – Réanimation<br>Hôpital de la Croix Rousse/Groupement Hospitalier Nord<br>Hospices Civils de Lyon<br>103, Grande rue de la Croix Rousse, 69004 Lyon<br>Tél : 04.26.10.94.93; Fax : 04.72.07.17.74<br>Email : laurent.bitker@chu-lyon.fr
**VERSION DU PROTOCOLE**	Version 1 du 03/03/2021
**JUSTIFICATION / CONTEXTE**	Une balance hydrosodée positive (entrées > sorties) s'exprime cliniquement par l'accumulation de fluides dans les tissus ; elle participe à l'ischémie tissulaire et s'associe à une morbi-mortalité élevée chez les patients de réanimation. Des stratégies visant à contrôler l'apparition d'une balance entrées/sorties positive ont montré un bénéfice sur la durée de ventilation mécanique et la récupération rénale, et ceci sans dégrader la survie. *A contrario*, un excès de déplétion hydrosodée (entrées <<< sorties) pourrait faire courir le risque d'une hypoperfusion tissulaire par baisse du débit cardiaque, selon le rationnel reposant sur la relation entre précharge cardiaque et débit cardiaque (loi de Starling). En somme, il existe un rationnel physiologique soutenant le maintien d'une balance entrées/sorties neutre (entrées = sorties).<br><br>Les patients atteints d'insuffisance rénale aiguë sont particulièrement à risque de développer un état de surcharge hydrosodée, leur diurèse étant le plus souvent altérée. Chez les patients de réanimation traités par épuration extra-rénale (EER), l'ultrafiltration nette (UF_{NET}) s'ajoute aux sorties ; elle remplace la diurèse lorsque le patient est oligo-anurique. Malgré son utilisation, 25% des patients traités par EER présentent une accumulation hydrosodée supérieure à 10% de leur poids, état qui s'associe à un doublement de la mortalité dans cette population. De plus, l'effet d'une UF_{NET} élevée a montré un effet contradictoire sur la mortalité dans 2 études observationnelles et rétrospectives, quand un essai randomisé pilote comparant une stratégie de déplétion intensive chez des patients de réanimation, *versus* le standard de soins n'a pas observé d'évènements indésirables en lien avec l'intervention.<br><br>Ces études justifient d'individualiser la déplétion hydrosodée par UF_{NET}, en s'appuyant sur un monitorage hémodynamique fonctionnel comme estimateur de l'état volémique du patient. En effet, notre hypothèse princeps est que la surcharge hydrosodée s'installe du fait d'épisodes d'instabilité hémodynamique motivant l'arrêt à tort de l'UF_{NET}. En effet, dans un travail précédent, 80% des épisodes hypotensifs en EER intermittente en réanimation n'étaient pas associés à un état de précharge-dépendance. Ce qui signifie que, dans le cas où l'évaluation hémodynamique permettrait d'éliminer le risque d'hypovolémie induit par l'UF_{NET}, la déplétion pourrait être poursuivie.<br><br>**Hypothèse** : Une stratégie de déplétion hydrosodée ciblant 100 ml/h ou plus d'UF_{NET} guidée par un protocole de monitorage continu du débit cardiaque permet de sécuriser le maintien d'une balance entrées/sorties nulle pendant 72h chez les patients de

	réanimation traitées par vasopresseurs et EER continue, comparativement à une stratégie reflétant les pratiques standards.
**OBJECTIFS**	**Objectif principal** : Montrer l'efficacité d'un protocole de déplétion hydrosodée ciblant 100 ml/h ou plus d'UF_{NET} guidé par le monitorage hémodynamique à maintenir neutre la balance entrées/sorties à 72h de l'inclusion, chez les patients de réanimation sous amines vasopressives et traités par EER continue, comparativement au standard de soin actuel (UF_{NET} entre 0 et 25 ml/h pendant 72h).<br><br>**Objectifs secondaires** :<br>1. Evaluer la sécurité du protocole hémodynamique du bras interventionnel sur les épisodes d'instabilité hémodynamique.<br>2. Décrire les mécanismes sous-jacents aux épisodes d'instabilité hémodynamique dans les 2 groupes, afin de les classer comme étant en lien ou non avec une précharge-dépendance du débit cardiaque<br>3. Evaluer la sécurité de l'intervention sur la fonction rénale à long terme.<br>4. Evaluer l'effet de l'intervention sur les principaux paramètres hémodynamiques.<br>5. Evaluer l'efficacité de l'intervention à maintenir une balance entrées/sorties neutre à 24h et 7 jours de l'initiation de l'intervention.<br>6. Comparer le volume d'UF_{NET} à 24h et 72h de l'initiation de l'intervention entre les 2 groupes.<br>7. Evaluer l'effet de l'intervention sur le nombre de jours sans défaillances d'organes à J28.<br>8. Evaluer l'effet de l'intervention sur l'intensité des défaillances d'organes.<br>9. Evaluer la capacité de l'intervention à diminuer l'œdème pulmonaire.<br>10. Evaluer la capacité de l'intervention à diminuer la durée de séjour en réanimation et à l'hôpital.<br>11. Evaluer la capacité de l'intervention à diminuer la mortalité à J28 et à J90 de l'inclusion.<br>12. Taux et vitesse d'inclusion en vue de la réalisation d'une étude de plus grande ampleur.
**METHODOLOGIE / SCHEMA DE LA RECHERCHE**	Etude interventionnelle de catégorie 1, hors produit de santé, pilote, d'efficacité et de supériorité, randomisée contre traitement de référence, en 2 groupes parallèles, ouverte, et multicentrique (inter-régionale).
**CRITERES DE JUGEMENT**	Tous les critères primaires ou secondaires sont évalués sur la population en intention de traiter (soit les patients vivants à H72 dans les 2 bras), sauf si spécifier autrement.<br><br>**Critère principal** : Le critère de jugement principal est la balance entrées/sorties mesurée chez les patients vivants à H72 de l'initiation de l'intervention, calculée comme la différence entre les entrées et les sorties du patient, cumulée au cours des 72h suivant l'initiation de l'intervention (H0).<br><br>**Critères secondaires** :<br>1. Nombre d'épisodes d'instabilité hémodynamique observés durant la période d'intervention (jusqu'à 72h de l'initiation de l'intervention) dans les 2 groupes. Ce nombre sera pondéré par la durée de la période d'observation, afin de prendre en compte le risque compétitif (soit de H0 jusqu'à H72). La définition des épisodes d'instabilité hémodynamique comprend la survenue de novo d'une hypotension artérielle (pression artérielle moyenne inférieure à la cible définie par le clinicien, et justifiant d'une intervention), d'une baisse de l'index cardiaque supérieure à 15%, d'une tachycardie (fréquence cardiaque > 120 battements par minute), ou de marbrures.<br>2. Nombre d'épisodes d'instabilité hémodynamiques (comme définis ci-dessus) avec précharge dépendance dans les 2 groupes, survenus durant la période d'intervention (de H0 à H72), ce nombre étant pondéré sur la durée de celle-ci.

	La précharge dépendance cardiaque est définie par une augmentation de l'index cardiaque dépassant un seuil prédéterminé lors d'une manœuvre posturale.<br>3. Fréquence dans les 2 groupes d'un critère composite « évènements rénaux adverses et majeurs » (*major adverse kidney events*), composé du décès, de la dépendance à l'EER, ou la persistance d'une créatininémie > 2 fois celle de base à J90 de l'inclusion (MAKE-90). La créatinine de base correspond à la créatininémie la plus basse mesurée dans un délai se situant entre 6 mois et 7 jours avant l'admission index. La dépendance à l'EER est définie comme la poursuite de l'EER à J90 (EER le jour de l'évaluation si patient est toujours sous technique continue, ou EER réalisée dans un intervalle de ± 2 jours dans le cas de techniques intermittentes). Chacun des critères composant le MAKE sera aussi évalué indépendamment.<br>4. Valeurs de pression artérielle moyenne, index cardiaque, pression veineuse centrale, lactatémie, et doses d'amines vasopressives, relevées toutes les 4 heures dans les 2 groupes, jusqu'à 72h de l'inclusion.<br>5. Balance entrées/sorties mesurée chez les patients vivants à 24h et 7 jours de l'initiation de l'intervention dans les 2 groupes. La balance entrées/sorties à H24 est définie comme pour le critère de jugement principal. La balance entrées/sorties à J7 sera estimé par la variation du poids corporel, comparativement au poids d'inclusion.<br>6. Volume cumulé d'UF_{NET} chez les patients vivants à 24h et 72h de l'initiation de l'intervention dans les 2 groupes. Le volume cumulé d'UF_{NET} sera calculé par l'addition des UF_{NET} rapportées toutes les 4 heures.<br>7. Durée sans défaillances d'organes (organ failure free days) dans les 2 groupes, quantifiée par le nombre de jour sans recours à la ventilation mécanique, et aux amines vasopressives, respectivement, et borné à 28 jours. Le sevrage des techniques de suppléance vitale (ventilation mécanique ou vasopresseurs) est défini comme l'absence à leur recours pendant une durée ≥ 48h.<br>8. Intensité des défaillances d'organe dans les 2 groupes, quantifiée par le score SOFA, composé de 6 éléments (neurologique, hémodynamique, respiratoire, rénal, hématologique et hépatique) côtés de 0 (fonction normale) à 4 (niveau de défaillance le plus grave), et colligé une fois par jour entre l'inclusion et H72.<br>9. Rapport PaO_2 (mesurée sur gaz du sang artériel) sur FiO_2, et eau pulmonaire extravasculaire indexée (IEPEV), telle qu'estimé par le dispositif de monitorage du débit cardiaque, mesurés chez les patients vivants à 24h, 72h et J7 de l'inclusion<br>10. Durée de séjour à l'hôpital et en réanimation, correspondant au nombre de jours entre l'admission jusqu'à la sortie ou au décès, recueilli selon les données administratives des centres participants.<br>11. Statut vital à J28 et J90 de l'inclusion, recueilli après appel des patients ou à l'aide de l'interrogatoire des bases de données d'hospitalisation.<br>12. Nombre de patients éligibles par mois et par centre, et nombre de patients effectivement inclus par mois.
**POPULATION CIBLE**	Cette étude portera sur des sujets adultes admis en réanimation, présentant une insuffisance circulatoire aiguë traitée par amines vasopressives, et traités depuis moins de 24h par EER continue pour une insuffisance rénale aiguë de grade III de la classification Kidney Disease: Improving Global Outcome (KDIGO). Les patients seront de plus porteurs d'un dispositif de monitorage continu de la pression artérielle et du débit cardiaque, justifié par leur condition initiale de prise en charge.
**CRITERES D'INCLUSION**	- Patient majeur (âge > 18 ans) affilié à un régime de sécurité sociale<br>- Traité par amines vasopressives (noradrénaline et/ou ou adrénaline) pour insuffisance circulatoire aiguë, sans critère de dose<br>- Présentant une insuffisance rénale aiguë de grade 3 selon l'échelle de sévérité KDIGO

	- Traité par EER continue depuis moins de 24h, quel que soit le mode (hémofiltration, hémodialyse, ou hémodiafiltration)<br>- Porteur d'un dispositif de monitorage continu du débit cardiaque calibré par thermodilution déjà en place au moment du screening.
**CRITERES DE NON INCLUSION**	- Patient recevant une technique d'oxygénation extra-corporelle<br>- Patient présentant une hémorragie active justifiant la transfusion de produits sanguins labiles<br>- Patient sous dialyse chronique ou porteur d'un greffon rénal<br>- Relai par hémodialyse intermittente prévu dans les 72 heures<br>- Patient hospitalisé pour accident vasculaire cérébral de cause ischémique ou hémorragique compliqué d'un coma et sous ventilation mécanique<br>- Hépatite fulminante, définie par la coexistence d'une agression hépatique aiguë, d'une encéphalopathie hépatique, d'un ictère, et d'une baisse du taux de prothrombine < 50%, apparue depuis moins de 15 jours.<br>- Evaluation impossible de la précharge dépendance par réalisation d'une manœuvre posturale (amputation ou immobilisation d'un ou des membres inférieurs, thrombose de la veine cave inférieure connue ou syndrome du compartiment abdominal)<br>- Allaitement ou grossesse en cours, identifiée par un taux de béta-HCG positif chez la femme en âge de procréer, et réalisé juste avant l'inclusion<br>- Limitations des thérapeutiques actives en place pour le patient, portant sur la ventilation mécanique ou la réanimation d'un arrêt cardio-respiratoire<br>- Décès imminent<br>- Patient sous tutelle ou curatelle, ou autre protection de justice<br>- Inclusion dans une autre étude interventionnelle dont le critère de jugement principal serait la balance entrées/sorties ou dont l'intervention porte sur l'hémodynamique, l'EER, ou la modification de la balance entrées/sorties<br>- Patient déjà inclus précédemment dans l'étude
**CRITERES DE SORTIE D'ETUDE**	- Retrait du consentement, ou refus de continuer de participer à la recherche<br>- Transfert du patient dans une autre unité ne participant pas à la recherche, avant H72<br>- Accident vasculaire cérébral de cause ischémique ou hémorragique compliqué d'un coma et sous ventilation mécanique<br>- Impossibilité de poursuivre le monitorage continu du débit cardiaque, pour des raisons techniques irréversibles<br>- Evaluation impossible de la précharge dépendance par réalisation d'une manœuvre posturale (amputation d'un ou des membres inférieurs, thrombose de la veine cave inférieure, syndrome compartimental abdominal)
**PROCEDURES**	**Bras interventionnel**<br>L'objectif de la stratégie étudiée est de maintenir une balance entrées/sorties neutre durant 72 heures à partir de l'inclusion, en paramétrant l'UF_{NET} ≥ 100 ml/h afin que celle-ci compense l'excès d'apport hydrosodé durant cette période (estimée à 2500 ml par jour dans cette population).<br><br>Le protocole d'évaluation et de gestion hémodynamique (ci-dessous) permettra de suspendre ou de diminuer l'UF_{NET} dans le bras interventionnel en cas d'apparition d'un profil à risque d'aggravation hémodynamique induit par la déplétion hydrosodée. L'évaluation repose sur une évaluation séquentielle des paramètres suivants : index cardiaque, lactatémie, évaluation de la précharge-dépendance, et pression veineuse centrale. Cette évaluation est réalisée toutes les 4 heures par l'infirmière en charge du patient en collaboration avec le clinicien, et/ou en cas d'aggravation hémodynamique. La conséquence de l'évaluation peut être la poursuite, la diminution, ou la suspension de l'UF_{NET} selon le profil identifié. En cas de suspension de l'UF_{NET}, l'intervention est reprise lors de l'évaluation suivante en cas de disparition du profil à risque. L'UF_{NET} pourra être

ajustée à des taux plus élevées pendant la période de l'intervention si le clinicien le décide. L'UF_{NET} horaire ne pourra dépasser 400 ml.

BRAS INTERVENTIONEL

Evaluation hémodynamique toutes les 4 heures

Calibration systématique du PiCCO
(3 injections de 15 ml de SSI froid)
Lactates artériels de moins de 8 heures

- Vérifier position des capteurs, fixés au bras du patient (point phlébostatique)
- Rincer les lignes artérielle et de PVC (flush)
- PVC à mesurer en décubitus, à 0°, et en fin d'expiration
- Lever de jambe passif : bascule du lit depuis la position assise à 45° ou Trendelenburg +13°/-13° si DV

Objectif : UF_{NET} à 100 ml/h ou plus

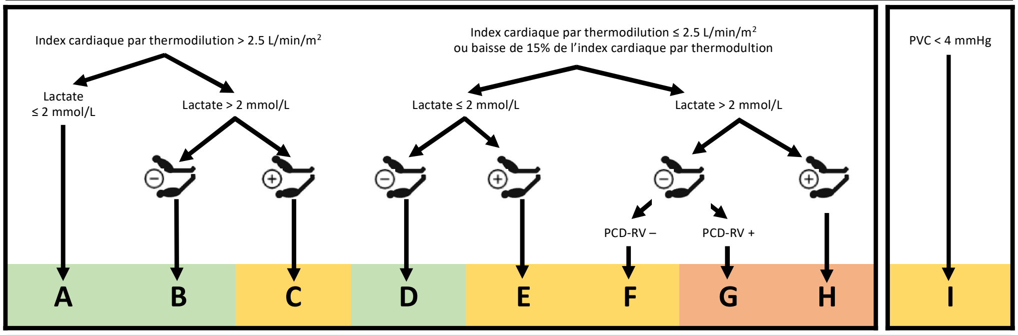

**Profil Vert (A, B ou D)**	**Profil Jaune (C, E, F ou I)**	**Profil Rouge (G ou H)**
UF_{NET} à 100 ml/h ou plus<br>⚠ UF_{NET} 50 à 100 ml/h si profil **rouge** au tour précédent	UF_{NET} à 50 à 100 ml/h<br>⚠ UF_{NET} 0 à 50 ml/h si profil **rouge** au tour précédent	UF_{NET} à 0 ml/h

Reporter la lettre et l'UF_{NET} réglée dans le CRF

PCD-RV : Epreuve de remplissage vasculaire par administration de 500 ml de crystalloïde en moins de 30 minutes. Calibration de l'IC par thermodilution systématique au décours. Epreuve positive si augmentation de plus de **15%** de l'IC par thermodilution par rapport à la valeur avant remplissage

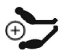

Patient en DD : épreuve de **lever de jambe** positive si augmentation de l'index cardiaque **continu** de plus de **10%** au cours de la première minute

Patient en DV : épreuve de **Trenbelenburg** positive si augmentation de l'index cardiaque **continu** de plus de **8%** au cours de la première minute

Bras contrôle

Le groupe contrôle reflète les pratiques actuelles de déplétion en EER continue, en paramétrant l'UF_{NET} entre 0 et 25 ml/h durant 72 heures à partir de H0. Le groupe contrôle bénéficiera du même niveau de monitorage hémodynamique, mais sans consigne d'ajustement de l'UF_{NET}.

Dans les 2 bras, l'heure d'initiation de l'intervention correspondra à H0. Au-delà de 72h, l'équipe en charge du patient sera libre de paramétrer l'UF_{NET} selon son choix dans les 2 groupes. Dans les 2 groupes, l'UF_{NET} pourra être augmentée en cas d'apparition d'une insuffisance respiratoire aiguë par œdème pulmonaire hydrostatique.

RAPPORT BENEFICES/RISQUES

Bénéfices

- Pour le patient randomisé dans le bras interventionnel, nous nous attendons aux bénéfices suivants : amélioration de l'oxygénation secondaire à une diminution de l'œdème pulmonaire hydrostatique, contrôle de la formation des œdèmes viscéraux se traduisant par une meilleure récupération de la fonction rénale à J90 de l'inclusion, et contrôle de la formation des œdèmes périphériques avec amélioration de la capacité de réhabilitation, se traduisant par une durée de séjour en réanimation plus courte.
- Sur le plan scientifique et médical, cette étude pourrait permettre une avancée nette dans notre compréhension de l'impact hémodynamique de l'UF_{NET} au cours de l'EER continue chez les patients de réanimation. Plus spécifiquement, elle

	pourrait induire un changement de paradigme, remettant en question l'association empirique entre UF_{NET} et instabilité hémodynamique.<br>**Risques et contraintes**<br>- Le principal risque couru par les patients randomisés dans le groupe interventionnel est celui d'une déplétion excessive entrainant une baisse du débit cardiaque, et donc une hypoperfusion systémique. Cependant, le rôle du protocole de monitorage hémodynamique est justement de détecter une situation à risque d'entrainer une baisse du débit cardiaque par excès de déplétion hydrosodée, et utilise des bornes de sécurité afin de stopper l'UF_{NET}, le cas échéant. Par ailleurs, l'objectif du protocole est donc bien de maintenir la balance entrées/sorties neutre, et non pas de la négativer systématiquement, bien que cette possibilité soit laissé au clinicien en charge du patient.<br>- A l'inverse, un risque potentiel est celui de développer un œdème pulmonaire hydrostatique avec insuffisance respiratoire aiguë. Afin de limiter ce risque, nous autorisons la déplétion hydrosodée en cas d'existence d'arguments en faveur d'une insuffisance respiratoire aiguë secondaire au développement d'une surcharge hydrosodée.<br>En conclusion, la balance bénéfice/risque de l'intervention et du protocole est en faveur de l'évaluation de celui-ci.
**NOMBRE DE SUJETS**	Nombre total de sujet : 58 sujets<br>Bras interventionnel : 29 sujets<br>Bras contrôle : 29 sujets
**DUREE DE L'ETUDE**	Durée de la période d'inclusion : 24 mois<br>Durée de la participation pour chaque sujet : 90 jours<br>Durée totale de l'étude : 27 mois
**LIEU DE LA RECHERCHE**	Nombre de centre participant : 4<br>- Service de Médecine Intensive – Réanimation, Hôpital de la Croix Rousse, Hospices Civils de Lyon, Lyon (centre coordinateur)<br>- Service de réanimation polyvalente, Hôpital Nord-Ouest, Villefranche-sur-Saône<br>- Service de Médecine Intensive – Réanimation, Hôpital Lapeyronie, CHU de Montpellier, Montpellier<br>- Service de Médecine Intensive – Réanimation, Hôpital Gabriel Montpied, CHU de Clermont-Ferrand, Clermont-Ferrand
**RETOMBEES ATTENDUES**	Cette étude pourrait entrainer un changement de paradigme majeur dans la gestion de la balance entrées/sorties chez les patients de réanimation traités par EER continue. En effet, une UF_{NET} sécurisée par le monitorage hémodynamique pourrait permettre de maintenir la balance hydro-sodée neutre, et ainsi limiter ses effets délétères suspectés sur le devenir des patients.<br><br>Le protocole d'évaluation hémodynamique du bras interventionnel pourrait permettre de prévenir la survenue d'une aggravation hémodynamique induite par la déplétion hydrosodée chez les patients à risque, tout en permettant la poursuite de la déplétion chez les autres patients, y compris les plus instables.<br><br>Les résultats de cette étude permettront de générer des hypothèses quant aux effets bénéfiques d'une stratégie de contrôle de la balance entrées/sorties sur le devenir des patients et des défaillances d'organes, tout en apportant des éléments importants de faisabilité en vue d'une étude de plus large ampleur.

LISTE DES ABREVIATIONS

**ANSM**	Agence Nationale de Sécurité des Médicaments et des produits de santé
**ARC**	Attaché de Recherche Clinique
**BPC**	Bonnes Pratiques Cliniques
**CNIL**	Commission Nationale Informatique et Liberté
**CPP**	Comité de Protection des Personnes
**CSP**	Code de Santé Publique
**eCRF**	Electronic Case Report Form (cahier d'observation dématérialisé)
**EER**	Epuration extra-rénale
**EI**	Evénement Indésirable
**EIG**	Evénement Indésirable Grave
**FC**	Fréquence cardiaque
**FiO_2**	Fraction inspirée en oxygène
**FN**	Fait Nouveau
**HCL**	Hospices Civils de Lyon
**IC**	Index cardiaque
**ICH**	International Conference on Harmonisation
**IDE**	Infirmier Diplomé d'Etat
**IEPEV**	Index d'eau pulmonaire extra-vasculaire
**ITT**	Intention to treat (analyse en intention de traiter)
**KDIGO**	Kidney Disease : Improving Global outcomes (recommendations internationales en néphrologie)
**MAKE**	Major Adverse Kidney Event (événement rénal, adverse et majeur)
**MR**	Méthodologie de Référence
**PAM**	Pression artérielle moyenne
**PVC**	Pression veineuse centrale
**RGPD**	Règlement Général sur la Protection des Données
**SDRA**	Syndrome de détresse respiratoire aiguë
**SOFA**	Sepsis-related Organ Failure Assessment (score d'évaluation des défaillances d'organes)
**SUSAR**	Suspected Unexpected Serious Adverse Reaction
**TEC**	Technicien d'Etude Clinique
**UF_{NET}**	Ultrafiltration nette
**VRB**	Volontaires pour les Recherches Biomédicales

SOMMAIRE

1 INFORMATIONS GENERALES 12
1.1. Titre 12
1.2. Identifiants du projet et historique des mises à jour 12
1.3. Promoteur 12
1.4. Investigateurs 13
1.4.1. Investigateur principal 13
1.4.2. Investigateurs associés 13
1.5. Méthodologiste – Biostatisticien 13
1.6. Comités 14
1.6.1. Comité scientifique 14
2 JUSTIFICATION SCIENTIFIQUE 14
2.1 Etat actuel des connaissances – Rationnel 14
2.1.1 Rationnel 14
2.1.2 Originalité de la recherche 15
2.2 Hypothèse de la recherche 15
2.3 Justification des choix méthodologiques 15
2.4 Population cible 16
2.5 Rapport bénéfices/risques 16
2.6 Retombées attendues 18
3 OBJECTIFS DE LA RECHERCHE 18
3.1 Objectif principal 18
3.2 Objectifs secondaires 18
4 CONCEPTION DE LA RECHERCHE 19
4.1 Type d'étude 19
4.2 Méthode pour la randomisation 19
4.3 Critères de jugement 20
4.3.1 Critère de jugement principal 20
4.3.2 Critères de jugement secondaires 21
5 CRITERES D'ELIGIBILITE 22
5.1 Critères de pré-inclusion 22
5.2 Critères d'inclusion 22
5.3 Critères de non inclusion 22
5.4 Critères de sortie prématurée 22
5.5 Modalités de recrutement et faisabilité 23
5.5.1 Modalités de recrutement 23
5.5.2 Faisabilité 23
6 STRATEGIES EXPERIMENTALES 24
6.1 Stratégie à l'étude 24
6.2 Stratégie de comparaison 30
6.3 Circuit des produits 31
6.4 Insu 31
6.5 Traitements associés autorisés et interdits 31
6.5.1 Remplissage vasculaire 31
6.5.2 Gestion des amines vasopressives 31
6.5.3 Gestion de l'EER 31
6.5.4 Gestion des diurètiques 32
6.5.5 Gestion de l'insuffisance respiratoire aiguë par oedème pulmonaire hydrostatique dans le groupe contrôle 32
6.5.6 Critères de qualité du monitorage hémodynamique 32

7 ORGANISATION GÉNÉRALE **33**
7.1 Calendrier de l'étude **33**
7.2 Schéma général **34**
7.3 Tableau récapitulatif **34**
7.4 Déroulement de l'étude **36**
7.4.1 Screening – Pré-inclusion 36
7.4.2 Visite d'inclusion / Randomisation / H-2 à H0 (ou J1), en réanimation 36
7.4.3 Visites de suivi de H0 à H72, en réanimation 38
7.4.4 Visite à J7, en réanimation 39
7.4.5 Visite de sortie de réanimation 39
7.4.6 Visite de suivi à J28, par appel téléphonique 40
7.4.7 Visite de suivi à J90, par appel téléphonique et biologie de ville 40
7.4.8 Echantillons biologiques 41
7.4.9 Distinction soins et recherches 41
7.5 Règles d'arrêt temporaire ou définitif **43**
7.6 Collection d'échantillons biologiques **43**
8 ÉVALUATION DE LA SÉCURITÉ **44**
8.1 Définitions **44**
8.1.1 Evénement indésirable (EI) 44
8.1.2 Evénement ou effet indésirable grave (EIG) 44
8.1.3 Effet indésirable (EI) 44
8.1.4 Effet indésirable inattendu 44
8.1.5 Fait nouveau 44
8.2 Responsabilités de l'investigateur **45**
8.2.1 Modalités de détection et de recueil des événements indésirables 45
8.2.2 Notification des EIG 45
8.2.3 Evaluation de la causalité 46
8.2.4 Période de notification des EIG sans délai au promoteur par l'investigateur et modalités de suivi des événements indésirables graves, restrictions des EI/EIG 46
8.3 Responsabilités du promoteur **50**
8.3.1 Déclaration aux autorités compétentes 50
8.3.2 Description des effets indésirables potentiellement liés à la recherche (référence de sécurité pour l'évaluation du caractère attendu/inattendu par le promoteur) 50
8.4 Comité de surveillance indépendant **51**
9 ASPECTS STATISTIQUES **52**
9.1 Nombre de sujets nécessaires **52**
9.2 Population d'analyse **52**
9.3 Méthodes statistiques **53**
9.4 Analyses intermédiaires **53**
9.5 Méthode de prise en compte des données manquantes **53**
9.6 Gestion des modifications apportées au plan d'analyse **54**
9.7 Responsable des analyses **54**
10 SURVEILLANCE DE LA RECHERCHE **54**
11 DROITS D'ACCES AUX DONNEES ET DOCUMENTS SOURCES **54**
11.1 Accès aux données **54**
11.2 Documents sources **55**
11.3 Confidentialité des données **55**
12 CONTROLE ET ASSURANCE DE LA QUALITE **55**
13 CONSIDERATIONS ETHIQUES **56**

13.1 Autorités compétentes 56
13.2 Modifications substantielles 56
13.3 Information du patient et formulaire de consentement écrit 56
13.4 Déclaration de conformité 57
13.5 Période d'exclusion 57
13.6 Indemnisation des sujets et inscription au fichier national des personnes se prêtant à une recherche interventionnelle sur la personne humaine du 1° 57
14 GESTION ET CONSERVATION DES DONNEES 58
14.1 Cahier d'observation 58
14.2 Gestion des données 59
14.3 CNIL 59
14.4 Archivage 59
15 FINANCEMENT ET ASSURANCE 59
15.1 Budget de l'étude 59
15.2 Assurance 60
16 REGLES RELATIVES A LA PUBLICATION 60
17 REFERENCES BIBLIOGRAPHIQUES 60

1 INFORMATIONS GENERALES

1.1. Titre

Neutralisation de la balance hydrosodée guidée par l'hémodynamique fonctionnelle au cours de l'épuration extra-rénale continue en réanimation – Etude GO-NEUTRAL

1.2. Identifiants du projet et historique des mises à jour

Code promoteur : 69HCL20_1243
Numéro d'enregistrement clinicaltrials.gov : *NCTXXXXX*
Avis favorable du CPP XX le :
Autorisation de l'ANSM le :

Historique des versions		
**Version**	**Date**	**Motif de la mise à jour**
0.1	16/08/2019	Rédaction initiale
1	03/03/2021	Version soumise aux autorités

1.3. Promoteur

- *Identité :*

Hospices Civils de Lyon
BP 2251
3 Quai des Célestins
69229 LYON Cedex 02

- *Signature du protocole au nom du Promoteur :*

Alexandre PACHOT, Directeur de la Recherche Clinique et de l'Innovation
Hospices Civils de Lyon, Direction de la Recherche Clinique et de l'Innovation, Siège Administratif, BP 2251, 3 Quai des Célestins, 69229 LYON Cedex 02
Tél : 04 72 40 68 52, Fax : 04 72 40 68 69

- *Responsable de la recherche au niveau du Promoteur :*

Valérie PLATTNER, médecin référent
Hospices Civils de Lyon, Direction de la Recherche Clinique et de l'Innovation , Siège Administratif, BP 2251, 3 Quai des Célestins, 69229 LYON Cedex 02
Tél : 04 72 40 68 40, Fax : 04 72 11 51 90

- *Responsable de la vigilance des essais au niveau du Promoteur :*

Marina NGUON, pharmacien référent
Hospices Civils de Lyon, Direction de la Recherche Clinique et de l'Innovation, Siège Administratif, BP 2251, 3 Quai des Célestins, 69229 LYON Cedex 02
Tél : 04 72 40 68 26, Fax : 04 72 11 51 90

1.4. Investigateurs

1.4.1. Investigateur principal

Dr Laurent BITKER
Service de Médecine Intensive – Réanimation
Hôpital de la Croix Rousse/Groupement Hospitalier Nord
Hospices Civils de Lyon
103, Grande rue de la Croix Rousse, 69004 Lyon
Tél : 04.26.10.94.93 ; Fax : 04.72.07.17.74
Email : laurent.bitker@chu-lyon.fr

1.4.2. Investigateurs associés

- Pr Jean-Christophe RICHARD
  Service de Médecine Intensive – Réanimation
  Hôpital de la Croix Rousse/Groupement Hospitalier Nord
  Hospices Civils de Lyon
  103, Grande rue de la Croix Rousse, 69004 Lyon
  Tél : 04.26.10.94.93 ; Fax : 04.72.07.17.74
  Email : j-christophe.richard@chu-lyon.fr

- Dr Julien ILLINGER
  Service de réanimation
  Hôpital Nord-Ouest
  Plateau d'Ouilly, Gleizé BP 436, 69655 Villefranche-sur-Saône
  Tél : 04.74.09.29.31
  Email : jillinger@lhopitalnordouest.fr

- Pr Kada KLOUCHE
  Service de Médecine Intensive – Réanimation
  Hôpital Lapeyronnie, CHU de Montpellier
  371, avenue du Doyen Gaston Giraud, 34090 Montpellier
  Tél : 04.67.33.67.33
  Email : k-klouche@chu-montpellier.fr

- Pr Bertrand SOUWEINE
  Service de Médecine Intensive – Réanimation
  Hôpital Gabriel Montpied, CHU de Clermont-Ferrand
  58, rue Montalembert, 63003 Clermont-Ferrand
  Tél : 04.73.75.07.50
  Email : bsouweine@chu-clermontferrand.fr

1.5. Méthodologiste – Biostatisticien

Mr Pierre PRADAT
Centre de Recherche Clinique
Hôpital de la Croix Rousse/Groupement Hospitalier Nord
Hospices Civils de Lyon
103, Grande rue de la Croix Rousse, 69004 Lyon
Tél : 04.26.73.27.15 ; Fax : 04.26.73.27.34
Email : pierre.pradat@univ-lyon1.fr

1.6. Comités

1.6.1. Comité scientifique

Le conseil scientifique de l'étude sera composé des investigateurs du centre coordinateur (Dr Laurent Bitker et Pr Jean-Christophe Richard), du méthodologiste (Mr Pierre Pradat) et de la coordinatrice d'étude clinique (Mme Loredana Baboi). Ils s'assureront du bon rythme des inclusions, de la collection des données et de leur qualité, et de la déclaration des EI et EIG.

2 JUSTIFICATION SCIENTIFIQUE

2.1 Etat actuel des connaissances – Rationnel

2.1.1 Rationnel

La balance entrées/sorties correspond à la différence entre le volume cumulé des entrées (perfusion, médicaments dilués dans des fluides, remplissage vasculaire, alimentation, transfusion…) administrées à un patient, et les sorties (diurèse, drains chirurgicaux, selles, sueur, et autres pertes insensibles). Une balance entrées/sorties positive (entrées > sorties) s'exprime cliniquement par l'accumulation de fluides dans les tissus ; elle participe à l'ischémie tissulaire et peut avoir des conséquences graves (œdème aigu pulmonaire, aggravation d'un œdème cérébral, hépatite hypoxique) [1]. On parle de surcharge hydrosodée lorsque celle-ci dépasse 10% du poids corporel ; cette observation chez un patient de réanimation est fortement associée au risque de décès [2, 3].

Des stratégies visant à contrôler l'apparition d'une balance entrées/sorties positive (entrées ≤ sorties) en limitant le volume de fluides administrés aux patients, ou en potentialisant leur diurèse par l'adjonction de traitements diurétiques ont montré un bénéfice sur la durée de ventilation mécanique et la récupération rénale, et ceci sans dégrader la survie [4-6]. *A contrario*, un excès de déplétion hydrosodée (entrées <<< sorties) pourrait faire courir le risque théorique d'une hypoperfusion tissulaire par baisse du débit cardiaque, selon le rationnel physiopathologique reposant sur la relation entre précharge cardiaque et débit cardiaque (loi de Starling) [7]. En somme, il existe un rationnel physiologique soutenant au minimum le maintien d'une balance entrées/sorties neutre (entrées = sorties) si l'état hémodynamique du patient le permet.

Les patients atteints d'insuffisance rénale aiguë sont particulièrement à risque de développer un état de surcharge hydrosodée patente, leur diurèse étant le plus souvent altérée (entrées > sorties) [3]. Chez les patients de réanimation présentant le grade le plus sévère d'insuffisance rénale aiguë et traités par épuration extra-rénale (EER), l'ultrafiltration nette (UF_{NET}, correspondant au volume d'ultrafiltrat non remplacé) s'ajoute aux sorties ; elle remplace la diurèse lorsque le patient est oligo-anurique. Malgré les recommandations portant sur le contrôle de la balance entrées/sorties, un quart des patients traités par EER présente une accumulation hydrosodée supérieure à 10% de leur poids, état qui s'associe à un doublement de la mortalité dans cette population [3, 8, 9]. De plus, chez les patients les plus graves avec choc septique et sous EER, elle était positive de près de 4L à 72h de l'inclusion dans un essai randomisé récent testant 2 stratégies de gestion de l'EER [10].

Deux études observationnelles et rétrospectives ont apporté des conclusions contradictoires sur l'impact d'une UF_{NET} élevée sur la mortalité des patients traitées par EER en réanimation. La première, américaine et monocentrique, a montré une baisse de la mortalité ajustée à 1 an chez les patients recevant une UF_{NET} cumulée > 25 ml/kg/jour, comparée à < 20 ml/kg/jour [11]. L'autre, fondée sur une analyse post-hoc de l'essai RENAL, a montré des résultats inverses, avec une surmortalité dans le groupe de patient recevant une UF_{NET} > 1.75 ml/kg/h (soit > 42 ml/kg/jour), comparativement à ceux avec une UF_{NET} < 1.01 ml/kg/h (< 24.2 ml/kg/jour) [12]. Surtout, une étude pilote scandinave a comparé une stratégie active de contrôle de la balance entrées/sorties chez les patients en surcharge avec insuffisance rénale aiguë, par l'application d'une déplétion horaire > 1 ml/kg (en utilisant soit un traitement diurétique, soit l'UF_{NET}) *versus* le standard de soin, mais a dû être arrêtée du fait d'un taux d'inclusion insuffisant [13]. Il n'a cependant pas été observé dans cette étude d'évènements indésirables en lien avec l'intervention.

Outre les biais inhérents à leurs méthodologies, ces résultats contradictoires sont probablement la conséquence de l'application d'une stratégie thérapeutique non adaptée à la physiologie d'un patient donné, et en particulier à son statut hémodynamique. Interprétées ensemble, ces études laissent entrevoir la nécessité d'individualiser la déplétion hydrosodée par UF_{NET}, en s'appuyant sur un monitorage hémodynamique fonctionnel comme estimateur de l'état volémique du patient [14-17]. En effet, notre hypothèse physiopathologique princeps est que la surcharge hydrosodée s'installe du fait d'épisodes d'instabilité hémodynamique motivant l'arrêt de l'UF_{NET}. En effet, il est empiriquement entendu que tout épisode d'instabilité hémodynamique survenant en EER est dû à une déplétion hydrosodée excessive entrainant une baisse du débit cardiaque. Or, dans un travail précédent, 80% des épisodes hypotensifs en EER intermittente en réanimation n'étaient pas associés à un état de précharge-dépendance [18]. Par ailleurs une équipe parisienne a montré que la présence d'un état de précharge dépendance du débit cardiaque à l'initiation de l'EER était associée au risque de survenue d'un épisode d'instabilité hémodynamique [14]. *A contrario*, et dans le cas où l'évaluation hémodynamique permettrait d'éliminer le risque d'hypovolémie, la déplétion par UF_{NET} pourrait être poursuivie, tout en introduisant par exemple un autre traitement ciblant la cause sous-jacente (vasoplégie, hypocontractilité myocardique,…).

Il semble donc réaliste d'évaluer une stratégie permettant d'individualiser la déplétion hydrosodée afin de contrôler la balance entrées/sorties chez les patients traités par EER continue, en la guidant par le monitorage continu du débit cardiaque.

2.1.2 Originalité de la recherche

Le premier caractère original de ce travail tient dans le fait que, bien que le contrôle de la balance entrées/sorties soit largement recommandé, aucune étude n'a à ce jour testé la possibilité d'utiliser l'UF_{NET} avec comme objectif de maintenir la balance entrées/sorties neutre, en sécurisant cette déplétion par un monitorage hémodynamique fonctionnel et continu.

Le second élément original repose sur l'utilisation du monitorage continu du débit cardiaque afin d'identifier les patients à risque d'hypoperfusion tissulaire induite par la déplétion hydrosodée, tel que décrit dans le rationnel physiologique. En d'autres termes, ce monitorage nous permettra de rationaliser le réglage de l'UF_{NET} en fonction du profil hémodynamique de chaque patient, ce qui n'a jamais été évalué à ce jour.

Cette étude fournira aussi des informations essentielles sur les mécanismes sous-jacents aux épisodes d'instabilité hémodynamique survenant sous EER continue, empiriquement considérés à ce jour comme étant induit par l'UF_{NET}. Pour finir, cette étude d'efficacité permettra d'obtenir les éléments essentiels à la mise en place d'un essai randomisé multicentrique de plus grande échelle (nombre de sujet nécessaire, puissance).

2.2 Hypothèse de la recherche

Notre hypothèse est qu'une stratégie de déplétion hydrosodée par $UF_{NET} \geq 100$ ml/h guidée par un protocole de monitorage continu du débit cardiaque permet de sécuriser le maintien d'une balance entrées/sorties nulle (critère de jugement principal) pendant 72h chez les patients de réanimation traitées par vasopresseurs et EER continue, comparativement à une stratégie reflétant les pratiques standards (UF_{NET} entre 0 et 25 ml/h) [10].

2.3 Justification des choix méthodologiques

Le critère de jugement principal est la balance entrées/sorties à 72 heures de l'inclusion.

Tout d'abord, nous avons choisi ce critère intermédiaire afin d'évaluer l'efficacité de l'intervention, car il n'existe à ce jour aucun élément dans la littérature s'étant donné un tel objectif. Le pendant de cette observation est qu'il n'a pas été encore identifié de seuil au-delà duquel une différence induite de balance entrées/sorties se traduirait par un bénéfice pour le patient de réanimation. Le choix de ce critère intermédiaire reflète donc la volonté de montrer l'efficacité du protocole à contrôler la balance entrées/sorties, avant d'envisager dans un second temps d'en évaluer les bénéfices pour les patients cibles. Ce délai de 72h depuis l'inclusion correspond donc aussi à la période durant laquelle l'intervention sera appliquée.

Nous avons choisi d'évaluer ce critère intermédiaire à 72h de l'inclusion car cela correspond tout d'abord à un point d'évaluation fréquemment utilisé dans les études observationnelles et interventionnelles, permettant ainsi de comparer nos résultats à ceux déjà publiés [7, 11, 19]. De plus, dans l'étude RENAL évaluant la dose d'EER en réanimation, les survivants avaient une balance entrées/sorties qui devenait neutre à partir de 72h de traitement par EER, alors qu'elle restait positive chez les patients non-survivants [20]. Ce point d'évaluation permet aussi de limiter le nombre de perdus de vue, en comparaison à un point plus tardif, en lien avec la mortalité élevée attendue dans cette catégorie de patient (> 25% après H72) [10]. A noter aussi que le premier quartile de la durée de traitement par EER était de 3 jours dans le bras conservateur de l'étude IDEAL-ICU, ceci permettant de justifier d'un nombre suffisant de patient sous EER (75%) au moment de l'évaluation du critère de jugement principal [10]. Pour finir, il semble difficile de poursuivre l'intervention au-delà de 72h sans prendre le risque de voir augmenter le nombre de violations du protocole. Les cliniciens seront donc libres d'appliquer l'UF_{NET} de leur choix au-delà de 72h.

Le schéma expérimental est celui d'un essai multicentrique, randomisé, et ouvert. Du fait de la nature intrinsèque de l'intervention (UF_{NET} ≥ 100 ml/h pour obtenir une balance entrées/sorties = 0 ml, *versus* UF_{NET} entre 0 et 25 ml/h, gestion hémodynamique spécifique et gérée par le clinicien dans le bras interventionnel), il ne sera pas possible de garantir l'insu. De plus, afin de limiter un effet de contamination du bras interventionnel par le bras contrôle, nous avons volontairement borné l'UF_{NET} horaire maximale du bras contrôle entre 0 ml/h et 25 ml/h, ce d'autant que celles-ci reflètent les pratiques actuelles de soustraction hydrosodée sur les 3 premiers jours d'épuration extra-rénale telles que rapportées dans l'étude IDEAL-ICU [10].

Nous avons choisi un dispositif de monitorage du débit cardiaque continu et pauci-invasif comme étant au centre du protocole de monitorage hémodynamique, car celui-ci permet tout d'abord le suivi continu des valeurs de débit cardiaque un échantillonnage temporel élevé grâce à l'analyse du contour de l'onde de pouls, permettant l'évaluation quasi instantanée des variations transitoires du débit cardiaque suite à une manœuvre posturale, ce qui permet de prédire la réponse au remplissage. L'autre avantage de ce dispositif est qu'il repose sur une mesure de la valeur absolue du débit cardiaque calibré par thermodilution transpulmonaire, méthode ayant montré une excellente corrélation avec des méthodes de mesure de référence (cathéter pulmonaire). Pour finir, le dispositif permet le suivi en continu de la fonction cardiaque, à une fréquence et avec une facilité supérieure à celle d'une échographie transthoracique par exemple.

Les modalités de mise en œuvre de la recherche sont celles habituelles d'une étude en réanimation.

2.4 Population cible

Cette étude portera sur des sujets adultes admis en réanimation, présentant une insuffisance circulatoire aiguë traitée par amines vasopressives, et traités depuis moins de 24h par EER continue pour une insuffisance rénale aiguë de grade III de la classification Kidney Disease: Improving Global Outcome (KDIGO) [8]. Les patients seront de plus porteurs d'un dispositif de monitorage continu de la pression artérielle et du débit cardiaque, justifié par leur condition initiale de prise en charge.

2.5 Rapport bénéfices/risques

Bénéfices

Pour le patient randomisé dans le bras interventionnel, nous nous attendons aux bénéfices théoriques suivants induits par le contrôle de la balance entrées/sorties :

- Contrôle de la surcharge hydrosodée se traduisant par une amélioration de l'oxygénation secondaire à une diminution de l'œdème pulmonaire hydrostatique, estimés par le ratio de la pression partielle artérielle en oxygène (PaO_2) avec la fraction inspirée en oxygène (FiO_2), et la mesure indexée de l'eau pulmonaire extravasculaire.
- Contrôle de la formation des œdèmes viscéraux se traduisant par une plus importante récupération de la fonction rénale à J90 de l'inclusion, et une plus forte incidence du sevrage de la dialyse.

- Contrôle de la formation des œdèmes périphériques et amélioration de la capacité de réhabilitation, se traduisant par une durée de séjour en réanimation plus courte.

L'ensemble de ces éléments se traduiraient alors par une durée de séjour en réanimation raccourcie, corolaire d'une accélération de l'amélioration du patient. Cette première étude ayant un dessin d'étude pilote, la puissance n'a cependant pas été calculée pour la mise en évidence d'effets centrés-patient significatifs. Les patients randomisés dans le bras contrôle recevront quant à eux le standard de soin tel qu'appliqué actuellement en France, et reflété par les résultats de l'étude IDEAL-ICU (large essai randomisé français comparant l'introduction précoce versus tardive de l'EER en réanimation, et dont les résultats ont été publiés fin 2018 dans le *New England Journal of Medicine*) [10].

Sur le plan général, cette étude pourrait permettre une avancée nette dans notre compréhension de l'impact hémodynamique de l'UF_{NET} au cours de l'EER continue chez les patients de réanimation. Plus spécifiquement, elle pourrait induire un changement de paradigme, remettant en question l'association empirique entre UF_{NET} et instabilité hémodynamique. La mise en place de ce protocole au sein d'une plus large étude aux critères de jugement cliniques permettra d'évaluer si cette optimisation de la prise en charge se traduirait par une amélioration clinique significative.

Risques et contraintes

Le principal risque couru par les patients randomisés dans le groupe interventionnel est celui d'une déplétion excessive par UF_{NET} entrainant une baisse de la précharge cardiaque et donc du débit cardiaque. La baisse de celui-ci peut entrainer une hypoperfusion systémique avec défaillance d'organes secondaire (ischémie mésentérique, ischémie rénale, accident vasculaire cérébral) [21].

Cependant, le rôle du protocole de monitorage hémodynamique est justement de détecter une situation à risque d'entrainer une baisse du débit cardiaque par excès de déplétion hydrosodée, et utilise des bornes de sécurité (protocole décrit dans le détail dans la section 6.1). Ces risques sont théoriques, et la responsabilité de la déplétion dans leurs survenues reste incertaine, ce d'autant qu'un excès d'œdème (pulmonaire ou viscéral) entraine les mêmes conséquences que celles citées ci-dessus [22, 23]. Ces éléments de physiologie laissent donc place à une « équipoïse clinique ». Pour finir, le risque de déplétion excessive (ou excessivement rapide) semble limité. En effet, en prenant comme base les données de l'étude IDEAL-ICU, les patients devraient nécessiter une UF_{NET} médiane de 2700 ml/jour pendant 3 jours (ou 110 ml/h pendant 72h) pour neutraliser leur BHS, soit environ 1.4 ml/kg/h chez un individu de 80 kg, ce qui est à considérer comme étant modérée [10]. L'objectif du protocole est donc bien de maintenir la balance entrées/sorties neutre, et non pas de la négativer systématiquement, bien que cette possibilité soit laissée au clinicien en charge du patient. Les effets indésirables attendus en lien avec l'intervention sont décrits dans la section 8.3.

Un risque potentiel est celui de développer un œdème pulmonaire hydrostatique avec insuffisance respiratoire aiguë, faisant courir le risque surajouté d'une ventilation mécanique invasive ou non-invasive. Afin de limiter ce risque, nous avons implémenté un protocole, dans les 2 bras de l'étude, autorisant la déplétion hydrosodée transitoire par UF_{NET} en cas d'existence d'arguments en faveur d'une insuffisance respiratoire aiguë secondaire au développement d'une surcharge hydrosodée.

Une épreuve d'évaluation de la précharge dépendance cardiaque sera réalisée dans les groupes par la réalisation d'une manœuvre posturale (lever de jambes passif en décubitus dorsal ou Trendelenburg en décubitus ventral). Cette épreuve, réalisée toutes les 4 heures, est indolore et est déjà intégrée dans la pratique courante des équipes participant à la recherche.

Les patients devront réaliser un prélèvement sanguin à J90 de la randomisation afin de mesurer leur taux de créatinine plasmatique, marqueur de leur fonction rénale. Cette contrainte est acceptable, dans le sens où il est recommandé de surveiller la fonction rénale au décours d'un épisode d'insuffisance rénale aiguë [24, 25].

Aucun effet secondaire tardif en lien avec l'intervention n'est attendu.

En conclusion, la balance bénéfice/risque de l'intervention et du protocole est en faveur de l'évaluation de celui-ci.

2.6 Retombées attendues

Cette étude pourrait entrainer un changement de paradigme majeur dans la gestion de la balance entrées/sorties chez les patients de réanimation traités par EER continue. En effet, une UF_{NET} sécurisée par le monitorage hémodynamique pourrait permettre un meilleur contrôle de la balance entrées/sorties afin de maintenir celle-ci neutre, et ainsi limiter les effets délétères suspectés de la surcharge hydrosodée sur les tissus et de l'accumulation d'œdèmes sur le fonctionnement des organes et le devenir des patients [1, 26].

Le protocole d'évaluation hémodynamique du bras interventionnel pourrait permettre de prévenir la survenue d'une aggravation hémodynamique induite par la déplétion hydrosodée chez les patients à risque, tout en permettant la poursuite de la déplétion chez les autres patients, y compris les plus instables, ceci afin de maintenir la balance entrées/sorties neutre. L'étude permettra donc, par extension, de discuter de la pertinence de l'arrêt systématique de la déplétion en cas d'instabilité hémodynamique, en s'appuyant sur les données hémodynamiques avancées recueillies.

Les résultats de cette étude permettront de générer des hypothèses quant aux effets bénéfiques d'une stratégie de contrôle de la balance entrées/sorties sur le devenir des patients et des défaillances d'organes, tout en apportant des éléments importants de faisabilité en vue d'une étude de plus large ampleur.

3 OBJECTIFS DE LA RECHERCHE

3.1 Objectif principal

Montrer l'efficacité d'un protocole de déplétion hydrosodée par UF_{NET} (≥100 ml/h) guidé par le monitorage hémodynamique à maintenir neutre la balance entrées/sorties à 72h de l'inclusion, chez les patients de réanimation sous amines vasopressives et traités par EER continue, comparativement au standard de soin actuel (UF_{NET} entre 0 et 25 ml/h pendant 72h).

Le groupe interventionnel correspond à la neutralisation (entrées – sorties = 0 ml) de la balance entrées/sorties par l'application d'une UF_{NET} équivalente aux entrées reçues par le patient, et guidée par le monitorage hémodynamique fonctionnel. Pour cela, nous faisons l'hypothèse qu'un taux d'UF_{NET} de 100 ml/h ou plus pendant 72h sera suffisant pour neutraliser les entrées, estimées sur la base des études précédemment publiées montrant des apports (entrées) estimées à 2500 ml par jour sur les 3 premiers jours de traitement par épuration extra-rénale [10].

Le groupe contrôle reflète les pratiques actuelles de contrôle de la balance entrées/sorties chez le patient de réanimation sous EER, par l'application d'une UF_{NET} comprise entre 0 et 25 ml/h [10].

Nous faisons l'hypothèse que la balance entrées/sorties sera de +4000 ml ± 4000 à 72h de l'inclusion dans le groupe contrôle [10], et que la procédure de neutralisation de la balance entrées/sorties (intervention) permettra d'obtenir une balance entrées/sorties égale à 0 ml ± 4000 (neutre) à 72h de l'inclusion, soit équivalent à une diminution de 4000 ml. En effet, cette valeur (4000 ml) correspond à la balance entrées/sorties médiane mesurée à H72 dans l'étude IDEAL ICU (3711 ml dans le bras intervention précoce et 3917 ml dans le bras intervention tardive), arrondie à 4000 ml de façon conservatrice [10].

3.2 Objectifs secondaires

1. Evaluer la sécurité du protocole hémodynamique du bras interventionnel sur les épisodes d'instabilité hémodynamique, par la comparaison du nombre d'épisodes d'instabilité hémodynamique observés durant la période d'intervention, ce nombre étant pondéré sur celle-ci (jusqu'à 72h de l'initiation de

l'intervention) entre les 2 groupes. La définition des épisodes d'instabilité hémodynamique comprend la survenue de novo d'une hypotension artérielle (pression artérielle moyenne inférieure à la cible définie par le clinicien, et justifiant d'une intervention), d'une baisse de l'index cardiaque supérieure à 15% depuis la dernière mesure, d'une tachycardie (fréquence cardiaque > 120 battements par minute), ou de marbrures.

2. Décrire les mécanismes sous-jacents aux épisodes d'instabilité hémodynamique dans les 2 groupes, afin de les classer comme étant en lien ou non avec une précharge-dépendance du débit cardiaque (et donc potentiellement secondaire à la déplétion hydrosodée par UF_{NET}), par la comparaison du nombre d'épisodes d'instabilité hémodynamique avec précharge dépendance observés durant la période d'intervention (ce nombre étant pondéré sur celle-ci) survenus dans les 2 groupes entre l'inclusion et jusqu'à 72h de celle-ci.
3. Evaluer la sécurité de l'intervention sur la fonction rénale à long terme, en comparant entre les 2 groupes la survenue d'un critère composite dénommé « événements rénaux adverses et majeurs » (*major adverse kidney events* ou MAKE) et évalué à J90 [27]. Ce critère composite associe le décès, la dépendance à l'EER ou la persistance d'une élévation de la créatinémie > 2 fois celle de base à J90 de l'inclusion.
4. Evaluer l'effet de l'intervention sur les principaux paramètres hémodynamiques (pression artérielle moyenne, index cardiaque, pression veineuse centrale, lactatémie, dose d'amines vasopressives), comparativement au groupe contrôle sur une période de 72h après l'inclusion.
5. Evaluer l'efficacité de l'intervention à maintenir une balance entrées/sorties neutre chez les patients vivants à 24h et 7 jours de l'initiation de l'intervention, par la comparaison des balances hydrosodées mesurées à 24h et 7 jours de l'inclusion, versus le groupe contrôle.
6. Comparer le volume d'UF_{NET} chez les patients vivants à 24h, et 72h de l'initiation de l'intervention entre les 2 groupes, mesuré par le volume cumulé d'UF_{NET} à 24h, et 72h de l'initiation de l'intervention.
7. Evaluer l'effet de l'intervention sur le nombre de jours sans défaillances d'organes à J28, en comparant entre les 2 groupes le nombre de jours sans recours à la ventilation mécanique ou aux amines vasopressives, censurés à J28.
8. Evaluer l'effet de l'intervention sur l'intensité des défaillances d'organes en comparant entre les 2 groupes le score SOFA total et par organe, mesurée quotidiennement de l'inclusion à H72 [28].
9. Evaluer la capacité de l'intervention à diminuer l'œdème pulmonaire, en comparant entre les 2 bras le ratio PaO_2/FiO_2 et la mesure de l'eau pulmonaire extravasculaire indexée à la surface corporelle chez les patients vivants à 24h, 72h et J7 de l'inclusion.
10. Evaluer la capacité de l'intervention à diminuer la durée de séjour en réanimation et à l'hôpital, en comparant entre les 2 bras la durée de séjour en réanimation et à l'hôpital.
11. Evaluer la capacité de l'intervention à diminuer la mortalité à J28 et à J90 de l'inclusion, en comparant le statut vital à J28 et J90 de l'inclusion dans les 2 bras.
12. Taux et vitesse d'inclusion en vue de la réalisation d'une étude de plus grande ampleur (report du nombre de patients éligibles par mois, et du nombre de patients inclus par mois).

4 CONCEPTION DE LA RECHERCHE

4.1 Type d'étude

Etude interventionnelle de catégorie 1, pilote, d'efficacité, de supériorité, randomisée contre traitement de référence, en 2 groupes parallèles, ouverte, multicentrique (inter-régionale).

4.2 Méthode pour la randomisation

La randomisation sera réalisée selon un rapport 1 pour 1 entre les 2 groupes, et sera stratifiée par centre investigateur et selon le statut de surcharge hydrosodée lors de l'inclusion (défini par un poids à l'inclusion > 10% du poids à l'admission en réanimation). Le groupe de traitement sera communiqué une fois le patient inclus. Le responsable de l'établissement de la liste sera le Centre de Recherche Clinique de l'Hôpital de la Croix Rousse, où la liste sera conservée. La randomisation se fera à l'aide de l'eCRF Ennov CliniCal, plateforme

sécurisée et disponible en ligne, après génération de l'identifiant d'étude du patient et vérification des critères d'inclusion et de non inclusion.

La stratification sur les centres participants permet de s'assurer d'une répartition homogène des 2 groupes dans chaque centre participant, ceci afin d'effacer les potentielles différences de prise en charge des patients par les équipes, et qui ne seraient pas encadrer par les co-interventions détaillées dans la section 6.5 (gestion du remplissage vasculaire, des amines, des diurétiques, ou encore de l'insuffisance respiratoire aiguë par œdème pulmonaire hydrostatique). La prise en charge des patients est par ailleurs rendue homogène par l'application de recommandations de prise en charge françaises et internationales des patients en insuffisance rénale aiguë, traités par EER, en état de choc et/ou en sepsis [24, 29-31]. Par ailleurs, la stratification sur l'existence d'une surcharge hydrosodée à l'inclusion permettra de prendre en compte les variations attendues de gravité clinique et de prise en charge entre les patients.

4.3 Critères de jugement

Les critères primaires ou secondaires sont évalués sur la population en intention de traiter (soit les patients vivants à H72 dans les 2 bras), sauf si spécifié autrement. La description de la population est reprise dans la section 9.2.

4.3.1 Critère de jugement principal

Le critère de jugement principal est la balance entrées/sorties mesurée chez les patients vivants à H72 de l'initiation de l'intervention, calculée comme la différence entre les entrées et les sorties (cf. infra) du patient, cumulée au cours des 72h suivant l'initiation de l'intervention (H0).

Une proportion de données manquantes allant jusqu'à 25% est attendue, en lien avec la mortalité élevée observée dans cette population [10]; le calcul du nombre de sujets nécessaires prend en compte ce risque. La liste de ce qui sera reporté comme entrées et sorties est présentée ci-dessous :

Les entrées sont définies comme le volume total :
- des médicaments ou électrolytes dilués dans des fluides vecteurs
- de la nutrition entérale et parentérale
- du remplissage vasculaire (solutés cristalloïdes et albumine)
- des produits sanguins labiles ou dérivés du sang

Les sorties sont définies comme le volume total :
- de la diurèse des 72h
- de l'UF_{NET} cumulée par 72h
- de drainage (thoracique, chirurgical)

$$Balance\ hydrosodée = \sum entrées - \sum sorties$$

Ces éléments seront reportés par l'équipe soignante toutes les 4 heures sur la pancarte de réanimation du patient, puis reportés par le technicien d'études cliniques de chaque centre participant dans l'eCRF Ennov CliniCal. Ces données sont systématiquement reportées sur les pancartes de surveillance des services de réanimation des centres participants, selon un intervalle allant de toutes les 2 heures à toutes les 4 heures en fonction des habitudes de service. Il ne sera pas possible de maintenir l'insu lors de ce recueil, l'intensité de l'UF_{NET} étant de facto liée à l'intervention. La liste ci-dessus ne contient pas les fluides non ou difficilement quantifiables et dont la participation à la balance entrées/sorties est théoriquement négligeable :
- Sueurs et perspiration
- Selles ou débit des stomies
- Rinçage et entretien des cathéters centraux et autres dispositifs

- Médicaments et fluides administrés par voie sous-cutanée

4.3.2 Critères de jugement secondaires

1. Nombre d'épisodes d'instabilité hémodynamique observés durant la période d'intervention (jusqu'à 72h de l'initiation de l'intervention) dans les 2 groupes. Ce nombre sera pondéré par la durée de la période d'observation, afin de prendre en compte le risque compétitif (soit de H0 jusqu'à H72) [32]. La définition des épisodes d'instabilité hémodynamique comprend :
    - Survenue *de novo* d'une hypotension artérielle, définie par une pression artérielle moyenne (PAM) inférieure à la PAM cible et justifiant l'intervention du clinicien
    - Survenue *de novo* d'une baisse de l'index cardiaque continu de plus de 15% par rapport à la précédente calibration ou survenue d'une baisse absolue de l'index cardiaque continu < 2.5 L/min/m^2 [33]
    - Apparition d'une tachycardie avec fréquence cardiaque > 120 battements par minute
    - Apparition ou extension de marbrures
2. Nombre d'épisodes d'instabilité hémodynamiques (comme définis ci-dessus) avec précharge dépendance dans les 2 groupes, survenus durant la période d'intervention (de H0 à H72), ce nombre étant pondéré sur la durée de celle-ci. La précharge dépendance cardiaque est définie par une augmentation significative de l'index cardiaque lors d'une manœuvre posturale (cf. infra).
3. Fréquence dans les 2 groupes d'un critère composite « évènements rénaux adverses et majeurs » (*major adverse kidney events*), composé du décès, de la dépendance à l'EER, ou la persistance d'une créatininémie > 2 fois celle de base à J90 de l'inclusion (MAKE-90) [27]. La créatinine de base correspond à la créatininémie la plus basse mesurée dans un délai se situant entre 6 mois et 7 jours avant l'admission index. La dépendance à l'EER est définie comme la poursuite de l'EER à J90 (EER le jour de l'évaluation si patient est toujours sous technique continue, ou EER réalisée dans un intervalle de ± 2 jours dans le cas de techniques intermittentes). Chacun des critères composant le MAKE sera aussi évalué indépendamment.
4. Valeurs de pression artérielle moyenne, index cardiaque, pression veineuse centrale, lactatémie relevées toutes les 4 heures, et doses d'amines vasopressives toutes les 24h, dans les 2 groupes, jusqu'à 72h de l'inclusion.
5. Balance entrées/sorties mesurée chez les patients vivants à 24h et 7 jours de l'initiation de l'intervention dans les 2 groupes. La balance entrées/sorties à H24 est définie comme pour le critère de jugement principal. La balance entrées/sorties à J7 sera estimée par la variation du poids corporel, comparativement au poids d'inclusion.
6. Volume cumulé d'UF_{NET} chez les patients vivants à 24h, et 72h de l'initiation de l'intervention dans les 2 groupes. Le volume cumulé d'UF_{NET} sera calculé par l'addition des UF_{NET} rapportées toutes les 4 heures durant la période d'intervention (H0 à H72).
7. Nombre de jours sans défaillances d'organes (organ failure free days) dans les 2 groupes, quantifié par le nombre de jour sans recours à la ventilation mécanique, et aux amines vasopressives, respectivement, et borné à 28 jours. Le sevrage des techniques de suppléance vitale (ventilation mécanique ou vasopresseurs) est défini comme l'absence à leur recours de façon définitive pendant une durée ≥ 48h. Une valeur de 0 jours sans défaillance d'organe est attribuée aux patients décédés avant J28, quel que soit leur statut vis-à-vis du sevrage de la ventilation mécanique ou des amines vasopressives. En cas d'intubations/extubations multiples ou de périodes répétées sous amines vasopressives avec multiples sevrages réussis, c'est la date correspondant au dernier sevrage qui est collectée.
8. Intensité des défaillances d'organe dans les 2 groupes, quantifiée par le score SOFA, composé de 6 éléments (neurologique, hémodynamique, respiratoire, rénal, hématologique et hépatique) côtés de 0 (fonction normale) à 4 (niveau de défaillance le plus grave), et colligé une fois par jour entre l'inclusion et H72 [28]
9. Rapport PaO_2 (mesurée sur gaz du sang artériel) sur FiO_2, et eau pulmonaire extravasculaire indexée (IEPEV), telle qu'estimé par le dispositif de monitorage du débit cardiaque (PiCCO®, Pulsion Medical Systems, Feldkirchen, Germany), mesurés chez les patients vivants à 24h, 72h et J7 de l'inclusion. La FiO2 est la FIO2 mesurée sur le respirateur chez les patients ventilés ou sous oxygénothérapie à haut débit. Chez les patients non ventilés la FiO2 est estimée selon la formule suivante : FiO2=0.21 +0.03×débit d'oxygène
10. Durée de séjour à l'hôpital et en réanimation, correspondant au nombre de jours entre l'admission jusqu'à la sortie ou au décès, recueilli selon les données administratives des centres participants.

11. Statut vital à J28 et J90 de l'inclusion, recueilli après appel des patients ou à l'aide de l'interrogatoire des bases de données d'hospitalisation.
12. Nombre de patients éligibles par mois et par centre, et nombre de patients effectivement inclus par mois.

5 CRITERES D'ELIGIBILITE

5.1 Critères de pré-inclusion

Il n'y a pas de critère de pré-inclusion en lien avec cette étude.

5.2 Critères d'inclusion

- Patient majeur (âge > 18 ans) affilié à un régime de sécurité sociale
- Traité par amines vasopressives (noradrénaline et/ou adrénaline) pour insuffisance circulatoire aiguë, sans critère de dose
- Présentant une insuffisance rénale aiguë de grade 3 selon l'échelle de sévérité des recommandations internationales KDIGO [8]
- Traité par EER continue depuis moins de 24h, quel que soit le mode (hémofiltration, hémodialyse, ou hémodiafiltration)
- Porteur d'un dispositif de monitorage continu du débit cardiaque calibré par thermodilution déjà en place au moment du screening.

5.3 Critères de non inclusion

- Patient recevant une technique d'oxygénation extra-corporelle
- Patient présentant une hémorragie active justifiant la transfusion de produits sanguins labiles
- Patient sous dialyse chronique ou porteur d'un greffon rénal
- Relai par hémodialyse intermittente prévu dans les 72 heures
- Patient hospitalisé pour accident vasculaire cérébral de cause ischémique ou hémorragique compliqué d'un coma et sous ventilation mécanique
- Hépatite fulminante, définie par la coexistence d'une agression hépatique aiguë, une encéphalopathie hépatique, un ictère, et d'une baisse du taux de prothrombine < 50%, apparue depuis moins de 15 jours.
- Evaluation impossible de la précharge dépendance par réalisation d'une manœuvre posturale (amputation ou immobilisation d'un ou des membres inférieurs, thrombose de la veine cave inférieure connue ou syndrome du compartiment abdominal connu). Le syndrome compartimental abdominal est défini par la coexistence d'une hyperpression intra-abdominale supérieure à 20 mmHg et d'au moins une défaillance d'organe survenant *de novo*.
- Allaitement ou grossesse en cours, identifiée par un taux de béta-HCG positif chez la femme en âge de procréer, et réalisé avant l'inclusion
- Limitations des thérapeutiques actives en place pour le patient, portant sur la ventilation mécanique ou la réanimation d'un arrêt cardio-respiratoire
- Décès imminent
- Patient sous tutelle ou curatelle, ou autre protection de justice
- Inclusion dans une autre recherche interventionnelle dont le critère de jugement principal serait la balance entrées/sorties ou dont l'intervention porte sur l'hémodynamique, l'EER, ou la modification de la balance entrées/sorties
- Patient déjà inclus précédemment dans l'étude

5.4 Critères de sortie prématurée

Les critères de sortie prématurée sont ceux ne permettant pas la poursuite de l'intervention jusqu'à H72 de son début. Ils comprennent :

- Retrait du consentement, ou refus de continuer de participer à la recherche
- Transfert du patient dans une autre unité ne participant pas à la recherche, avant H72
- Suspension permanente de l'intervention avant H72, pendant une durée supérieure à 8 heures, du fait du transfert du patient au bloc opératoire, en radiologie, ou autre.
- Mise en évidence d'une contre-indication permanente à l'intervention :
    - Accident vasculaire cérébral de cause ischémique ou hémorragique compliqué d'un coma et sous ventilation mécanique
    - Impossibilité de poursuivre le monitorage continu du débit cardiaque, pour des raisons techniques irréversibles
    - Evaluation impossible de la précharge dépendance par réalisation d'une manœuvre posturale (amputation d'un ou des membres inférieurs, thrombose de la veine cave inférieure, apparition d'un syndrome compartimental abdominal). Le syndrome compartimental abdominal est défini par la coexistence d'une hyperpression intra-abdominale supérieure à 20 mmHg et d'au moins une défaillance d'organe survenant *de novo*.

Le décès avant H72, ou l'arrêt de l'EER avant H72 ne sont pas des critères de sortie prématurée.

5.5 Modalités de recrutement et faisabilité

5.5.1 Modalités de recrutement

Les patients seront évalués et éventuellement recrutés au cours de leur hospitalisation dans les services de réanimation participants par l'équipe médicale ou l'équipe de recherche clinique, après avoir vérifié les critères d'inclusion et de non inclusion.

Le patient (ou un de ses proches) sera approché par l'équipe médicale en charge afin de lui apporter toutes les informations sur le protocole, ses risques et ses contraintes, et répondre à ses questions, suite à quoi un délai suffisant sera donné au patient (ou à un de ses proches) afin de prendre sa décision. En cas où le patient serait incapable de donner son accord (coma, anesthésie générale, patient vigile sous ventilation mécanique, ou tout autre présentation où il ne serait pas capable d'exprimer son avis ou poser des questions), un de ses proches sera alors approché. Le consentement du patient, ou celui de son proche sera alors recueilli dès que possible, après information claire loyale et adaptée.

Au cas où le patient serait incapable d'exprimer sa volonté, et où aucun de ses proches ne serait disponible malgré tous les moyens pour les contacter mis en œuvre, nous demanderons au Comité de Protection des Personnes évaluant le projet la possibilité d'utiliser une procédure d'inclusion en urgence. Celle-ci permettra l'inclusion du patient en l'absence de recueil possible de son consentement (coma, anesthésie générale, patient vigile sous ventilation mécanique, ou tout autre présentation où il ne serait pas capable d'exprimer son avis ou poser des questions), et dans la situation dans laquelle un proche n'est pas immédiatement disponible. Le consentement du patient ou celui de ses proches sera obtenu dès que possible à la suite de l'inclusion. Le patient devra confirmer son consentement, si celui-ci a été fourni par son proche, et dès qu'il sera en mesure de le faire, pour la poursuite de sa participation.

Une fois l'inclusion réalisée, la randomisation et l'initiation de l'intervention seront effectués dans les 2 heures suivant le recueil du consentement. Ce délai de 2 heures semble raisonnable, l'intervention consistant en la réalisation de mesures hémodynamiques et la modification de l'UF_{NET} sur le générateur de l'EER continue. Le délai de 72h durant lequel l'intervention est appliquée débute au moment de l'inclusion.

5.5.2 Faisabilité

Cette étude repose sur un rationnel physiologique simple (intégration du débit cardiaque et de la précharge-dépendance) déjà appliqué au lit du patient, et utilise une technique de monitorage hémodynamique déjà largement répandue [15, 17]. Le protocole d'évaluation hémodynamique dans le bras interventionnel a été dessiné afin d'être facile à mettre en place. Il nécessite toutes les 4 heures environ 10 min de temps infirmier intégré aux soins courants. Les résultats du protocole d'évaluation hémodynamique, et son impact sur la poursuite ou non de l'UF_{NET} seront colligés dans le CRF papier spécifique (document source) présent au lit du

patient (cahier IDE), comme déjà fait auparavant dans le centre coordinateur pour les études EHOSS et PRELOAD-CRRT (références : [15] et https://clinicaltrials.gov/ct2/show/NCT03139123).

Le nombre de patients **éligibles** prévu dans les centres participants est présenté dans la table ci-dessous.

Ville	Groupe Hospitalier	Nb lits	Nb patient éligible/mois	Total
Lyon	CHU Croix Rousse	15	1,5	36
Montpellier	CHU Lapeyronie	20	1	24
Villefranche-sur-Saône	CHG Hôpital Nord-Ouest	8	1	24
Clermont-Ferrand	CHU Gabriel Montpied	18	1	24
			**TOTAL**	**108**

Les centres participants ont été sélectionnés car experts dans la gestion hémodynamique avancée des patients de réanimation multi-défaillants. L'utilisation du monitorage continu du débit cardiaque y est standardisée et fait en routine, permettant de garantir 1) un nombre de sujets éligibles par mois et par centre suffisant, et 2) la qualité des données de monitorage hémodynamique recueillies. De plus, le centre coordinateur est expert dans l'évaluation hémodynamique fonctionnelle, ayant déjà recruté plus de 60 patients dans des études de schéma similaire (études EHOSS et PRELOAD-CRRT, [15] et https://clinicaltrials.gov/ct2/show/NCT03139123). Pour finir et à titre d'exemple, le centre coordinateur (Lyon) a inclus dans l'étude PRELOAD-CRRT entre le 1ier janvier 2019 et le 31 juillet 2019 8 patients ayant les mêmes caractéristiques que les patients-cibles de ce projet, soit un taux d'inclusion de 1.1 patient par mois, pour un taux d'inclusion attendu de 0.6 patient par mois.

6 STRATEGIES EXPERIMENTALES

6.1 Stratégie à l'étude

- **Définition et description de la stratégie / procédure**

L'objectif de la stratégie étudiée est de maintenir une balance entrées/sorties neutre durant 72 heures à partir de l'inclusion (ou jusqu'au décès, si plus précoce), en paramétrant l'UF_{NET} à 100 ml/h ou plus de soustraction hydrosodée, afin que celle-ci compense l'excès d'apport hydrosodé durant cette période. Le choix de garder neutre la balance entrées/sorties permet de limiter le risque de déplétion excessive.

En effet, sur la base des données d'entrées rapportées par l'étude IDEAL-ICU (entre 2500 ml et 3000 ml par jour entre J1 et J3), il devrait être nécessaire d'appliquer une UF_{NET} cumulée de 2500 ml par jour dans le bras interventionnel afin de neutraliser la balance entrées/sorties. Ceci correspond à une UF_{NET} horaire de 100 ml/h (ou 1,25 ml/kg/h chez un patient de 80 kg) [10]. Ces débits d'UF_{NET} restent modérées et à faible risque [13], et sont de l'ordre de ceux associés à une amélioration de la survie chez les patients de réanimation sous EER, comparativement à des taux d'UF_{NET} plus faibles [11].

L'intervention devra être débutée dans les 2 heures suivant l'inclusion, et sera poursuivie pour une durée totale de 72 heures. L'heure d'initiation de l'intervention correspondra à H0. Au-delà de 72h, l'équipe en charge du patient sera libre de paramétrer l'UF_{NET} selon son choix.

La procédure expérimentale n'est pas appliquée en cas de transfert temporaire sur un plateau technique (scanner, radiologie interventionnelle), si sa durée est < 4h. En cas de transfert dans un autre service, la procédure expérimentale est définitivement arrêtée.

L'UF_{NET} sera donc initialement réglée à 100 ml/h (ou plus si le clinicien en charge du patient l'indique) afin d'annuler la balance entrées/sorties selon la formule suivante :

$$Entrées = Sorties + UF_{NET}$$

ou

$$Entrées - Sorties - UF_{NET} = 0\ ml$$

L'UF_{NET} pourra être ajustée à des taux plus élevées pendant la période de l'intervention si le clinicien le décide. L'UF_{NET} horaire ne pourra dépasser 400 ml. Elle pourra aussi être diminuée, voire rendue nulle, en fonction du monitorage hémodynamique réalisé toutes les 4 heures et en cas d'épisodes d'instabilité hémodynamique. Ce protocole est décrit dans le détail ci-dessous.

Le **protocole d'évaluation et de gestion hémodynamique** permet de diminuer ou de suspendre l'UF_{NET} dans le bras interventionnel en cas d'apparition d'un profil à risque d'aggravation hémodynamique induit par la déplétion hydrosodée. Cette évaluation est réalisée toutes les 4 heures par l'infirmière en charge du patient en collaboration avec le clinicien, et plus souvent en cas d'aggravation hémodynamique. La conséquence de l'évaluation peut être la poursuite, la diminution, ou la suspension de l'UF_{NET} selon le profil identifié. Ce protocole est décrit dans la figure ci-dessous, et sa justification présentée dans le paragraphe suivant :

- **Justification de la stratégie / procédure choisie**

Une déplétion hydrosodée excessive par UF_{NET} peut diminuer la précharge cardiaque, et impacter le débit cardiaque si le patient se trouve dans la partie ascendante de la courbe de Starling (= précharge-dépendance, Figure 1) [34].

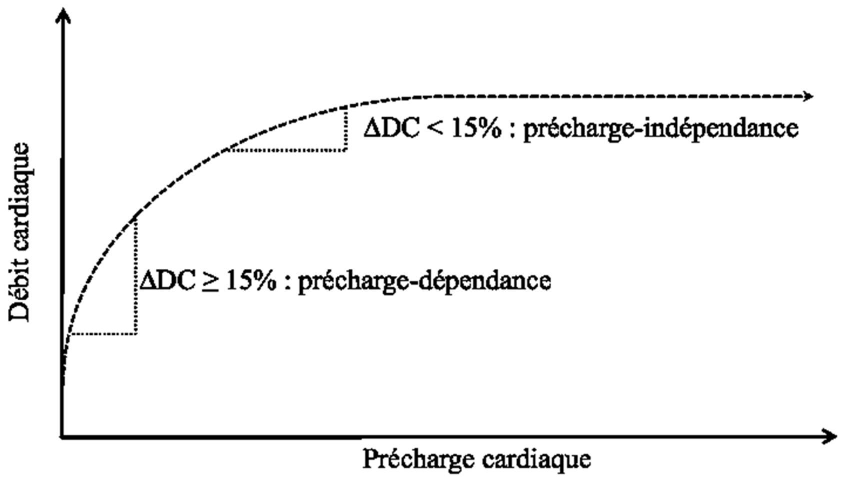

Figure 1. Relation entre précharge cardiaque et débit cardiaque selon la loi de Starling.
ΔDC : variation de la valeur absolue du débit cardiaque.

La précharge-dépendance est évaluable en pratique clinique grâce à :

1. L'utilisation d'un dispositif de mesure continue de l'index cardiaque par analyse de l'onde de pouls permettant un échantillonnage à haute temporalité au cours d'une épreuve dynamique, comme utilisé dans ce protocole ;
2. Et la réalisation d'une manœuvre posturale, dont le but est de mobiliser le sang veineux des membres inférieurs afin d'augmenter le retour veineux vers les cavités cardiaques. La manœuvre est réalisable de 2 façons :
    a. **Epreuve de lever de jambes passif** chez les patients en décubitus dorsal. Elle est positive en cas d'augmentation **≥ 10%** du débit cardiaque. Dans le cas de notre étude, l'épreuve de lever de jambes passif sera réalisée par la bascule du lit depuis une position semi-assise à 45° jusqu'à 0° avec le lit dans la même position et maintenue pendant 1 minute pendant laquelle la valeur maximale d'index cardiaque par analyse de l'onde de pouls sera recherchée.

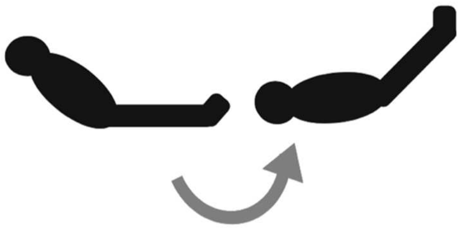

Figure 2. Modalité de réalisation de l'épreuve de lever de jambes passif

b. **Trendelenburg** chez les patients en décubitus ventral [35]. Elle est positive en cas d'augmentation **≥ 8%** du débit cardiaque. Dans le cas de notre étude, le Trendelenburg sera réalisé par la bascule du lit à plat depuis une position de +13° (tête vers le haut) jusqu'à -13° (tête en bas), et maintenue pendant 1 minute pendant laquelle la valeur maximale d'index cardiaque par analyse de l'onde de pouls sera recherchée.

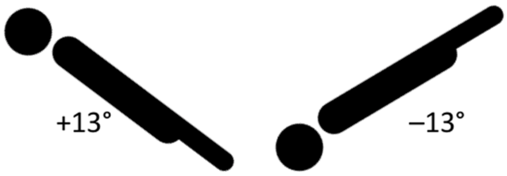

Figure 3. Modalité de réalisation du Trendelenburg

L'algorithme (Figure 4) utilise l'index cardiaque et la précharge dépendance, conjugués à la lactatémie artérielle et à la PVC afin de définir toutes les 4 heures 3 profils hémodynamiques de bas risque (profil vert), risque intermédiaire (profil orange), et haut risque (profil rouge). A chaque profil correspond un ajustement de l'UF_{NET} adapté, soit une poursuite de l'UF_{NET} en cas de profil vert, une diminution en cas de profil orange, et un arrêt de l'UF_{NET} en cas de profil rouge. Le protocole comprend 3 points particuliers :

- Nous avons choisi un seuil de PVC à 4 mmHg, en dessous duquel l'UF_{NET} est systématiquement diminuée, en rapport avec l'algorithme proposé par l'ARDS Network pour la gestion de la balance hydrique au cours du syndrome de détresse respiratoire aiguë (SDRA) [4]
- Suite à une suspension de l'UF_{NET} (haut risque hémodynamique, profil rouge de la figure) à un instant *t*, l'intervention (UF_{NET} ≥ 100 ml/h) n'est reprise à l'instant *t+4h* qu'en cas de profil hémodynamique normalisé (bas risque, profil vert). En cas de profil à risque intermédiaire (profil orange) à l'évaluation *t+4h*, l'UF_{NET} est reprise à une intensité diminuée de 50 ml/h.
- La mesure systématique de l'acide lactique dans les 8 heures précédentes dans le cadre du monitorage hémodynamique est habituelle dans le cadre du monitorage des patients les plus graves en réanimation (ceux avec défaillance multiviscérale). En cas de mesure de la lactatémie datant de moins de 8h, celle-ci ne sera pas renouvelée dans le cadre du monitorage.

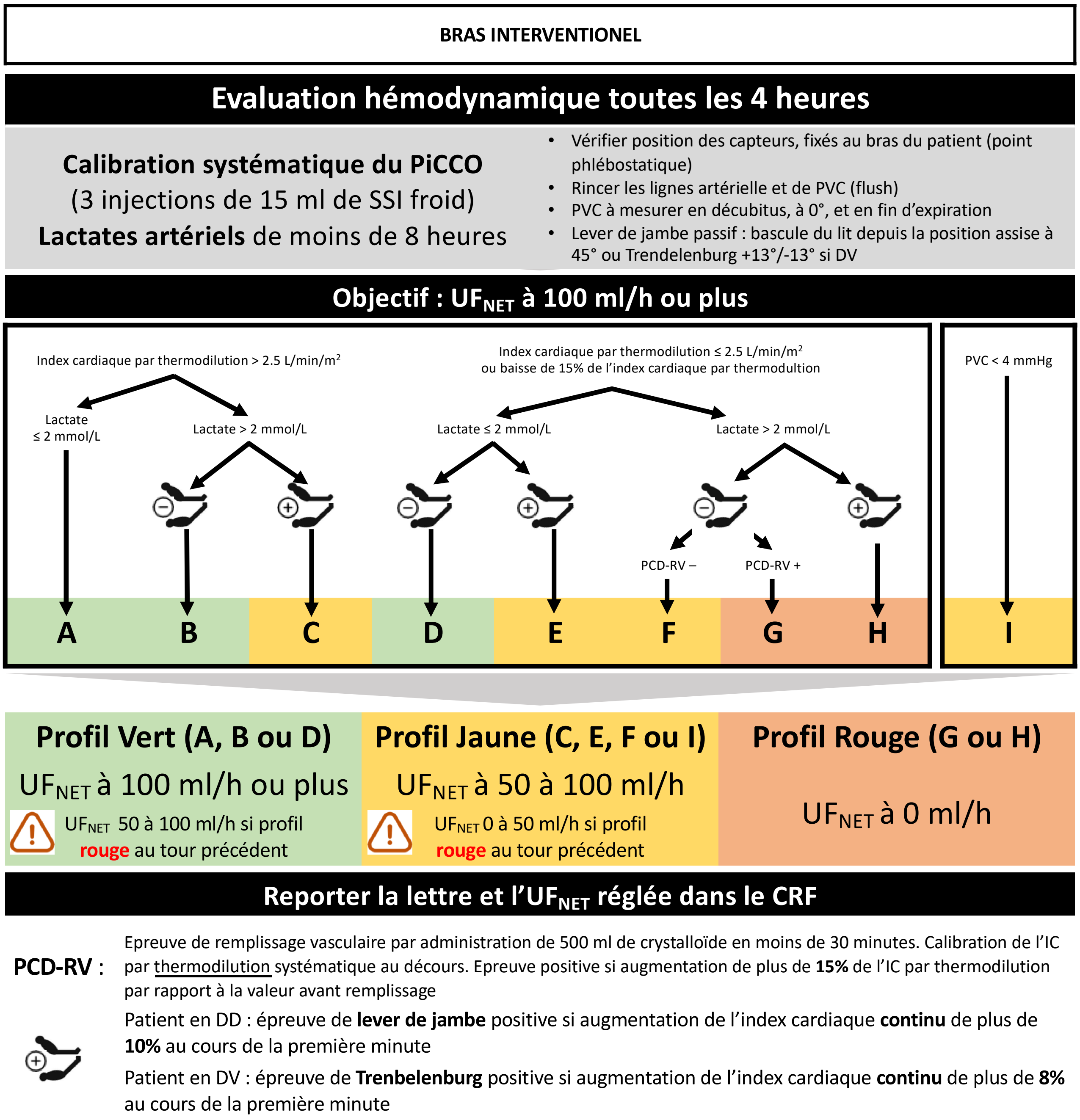

Figure 4. Protocole de gestion de l'UF_{NET} guidé par l'hémodynamique (bras interventionnel)
DD : décubitus dorsal ; DV : décubitus ventral ; PAM : pression artérielle moyenne ; PCD-RV : précharge-dépendance évaluée par une épreuve de remplissage (peut être soit positive + ou négative –) ; PVC : pression veineuse centrale ; UF_{NET} : ultrafiltration nette

Afin de standardiser les mesures, les capteurs de pression artérielle (et donc de mesure de l'index cardiaque en continu), et de PVC seront fixés au bras du patient, en regard du point phlébostatique, et ne seront donc pas impactés par les changements de position de celui-ci. La PVC sera mesurée en décubitus dorsal, à 0° et en fin d'expiration.

Nous faisons la distinction dans la collection des données entre index cardiaque calibré par la technique de thermodilution pulmonaire (IC_{TP}), et l'index cardiaque continu obtenu par le contour de l'onde de pouls artériel (ICC). A noter que dans les cas des profils F et G, c'est la valeur de l'IC_{TP} avant et après remplissage vasculaire qui est pris en compte pour définir la précharge dépendance.

A noter que dans le sous-groupe de patients au profil le plus menaçant (profil F et G), il est réalisé une épreuve de remplissage systématique par l'injection intravasculaire en moins de 30 minutes de 500 ml de soluté cristalloïde. Cette attitude diagnostique est recommandée chez les patients les plus graves afin de ne pas méconnaitre un faux négatif lors de la manœuvre posturale (estimé à environ 10% pour l'épreuve de lever de jambes passif dans la littérature) [36]. Ces remplissages diagnostiques ne pourront pas dépasser 2 x 500 ml par tranche de 24h de l'étude jusqu'à H72. Il ne contre-indique pas par contre la réalisation de remplissage vasculaire thérapeutique, réalisé selon les pratiques habituelles et recommandées décrites ci-dessous.
Cette injection est à considérer comme un traitement concomitant et non d'un traitement non expérimental.

Nous avons aussi intégré au protocole hémodynamique des événements déclencheurs, justifiant une réévaluation hémodynamique immédiate :

- Apparition ou extension de marbrures
- Apparition d'une tachycardie avec fréquence cardiaque > 120 battements par minute
- Survenue *de novo* d'une hypotension artérielle, définie par une pression artérielle moyenne (PAM) inférieure à la PAM cible et justifiant l'intervention du clinicien
- Survenue *de novo* d'une baisse de l'index cardiaque de plus de 15% par rapport à la précédente calibration ou survenue d'une baisse absolue de l'index cardiaque < 2.5 L/min/m^2

Nous avons choisi ces critères car ils sont soit associés à une surmortalité (marbrures, tachycardie, hypotension), ou directement en lien avec le rationnel physiopathologique de notre étude, à savoir qu'une baisse de l'index cardiaque ≥ 15% peut refléter une baisse significative de la précharge biventriculaire induite par un excès d'UF_{NET} [30, 33, 37]. Deux épisodes d'instabilité hémodynamiques devront être séparés d'au moins 1 heure pour être considérés comme indépendants l'un de l'autre, et donc mener à 2 évaluations hémodynamiques distinctes selon le protocole. Le protocole hémodynamique (Figure 5) en lien avec un épisode d'instabilité hémodynamique simplifie le processus d'évaluation de la précharge dépendance, en ne requérant que la réalisation que de la manœuvre posturale. Il ne nécessite donc pas de nouvelle calibration par thermodilution du dispositif de monitorage du débit cardiaque, ni la réalisation d'un lactate artériel. Le résultat de l'évaluation hémodynamique est lui aussi simplifié en 2 profils (jaune ou rouge) menant à l'ajustement de l'UF_{NET}. Ceci permet au clinicien d'intervenir en moins de 1 minute suite à l'apparition d'un tel épisode.

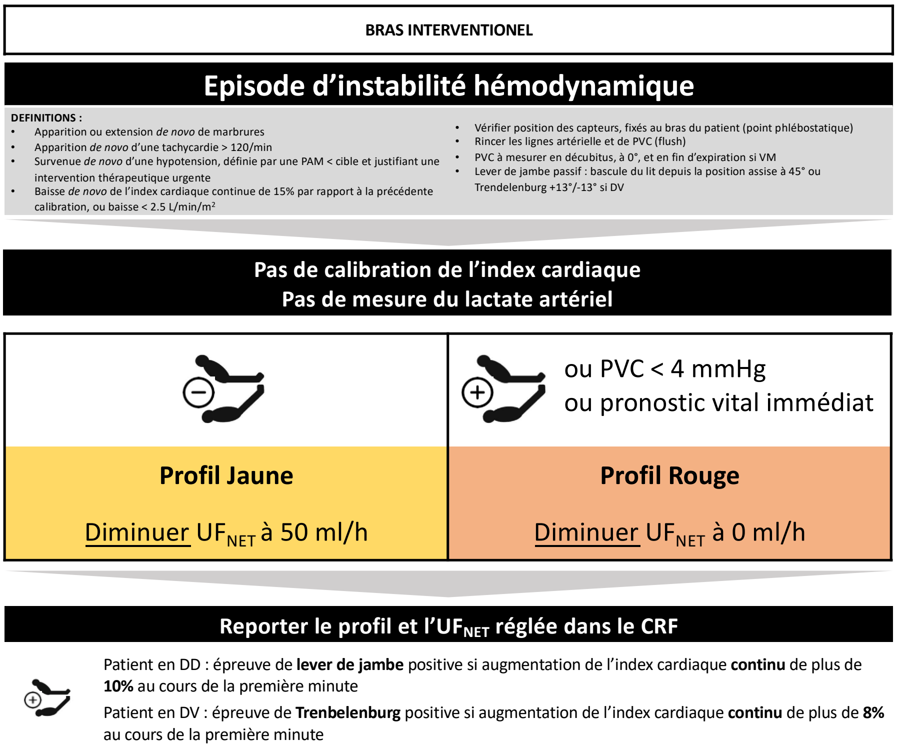

Figure 5. Protocole de gestion de l'UF_{NET} en cas d'instabilité hémodynamique (bras interventionnel)
DD : décubitus dorsal ; DV : décubitus ventral ; PAM : pression artérielle moyenne ; PVC : pression veineuse centrale ; UF_{NET} : ultrafiltration nette

La figure suivante (Figure 6) montre des exemples d'utilisation du protocole hémodynamique, et l'ajustement de l'UFNET découlant des évaluations hémodynamiques. En particuliers, on notera que dans les exemples 3, 4 et 5, l'UF_{NET} n'est pas reprise à dose maximale au décours d'un profil rouge, même en cas de profil vert, ceci afin de donner un délai suffisamment long (entre 4 et 8 heures) suite à l'apparition d'un profil à risque, et ne pas générer à nouveau un tel profil. Dans l'exemple 6, la survenue d'un épisode d'instabilité hémodynamique (éclair rouge) entraine un arrêt de l'UF_{NET}. Lors de l'évaluation suivante, l'identification d'un profil jaune (lettre C) <u>faisant suite à un profil rouge</u> fait reprendre une UF_{NET} diminuée.

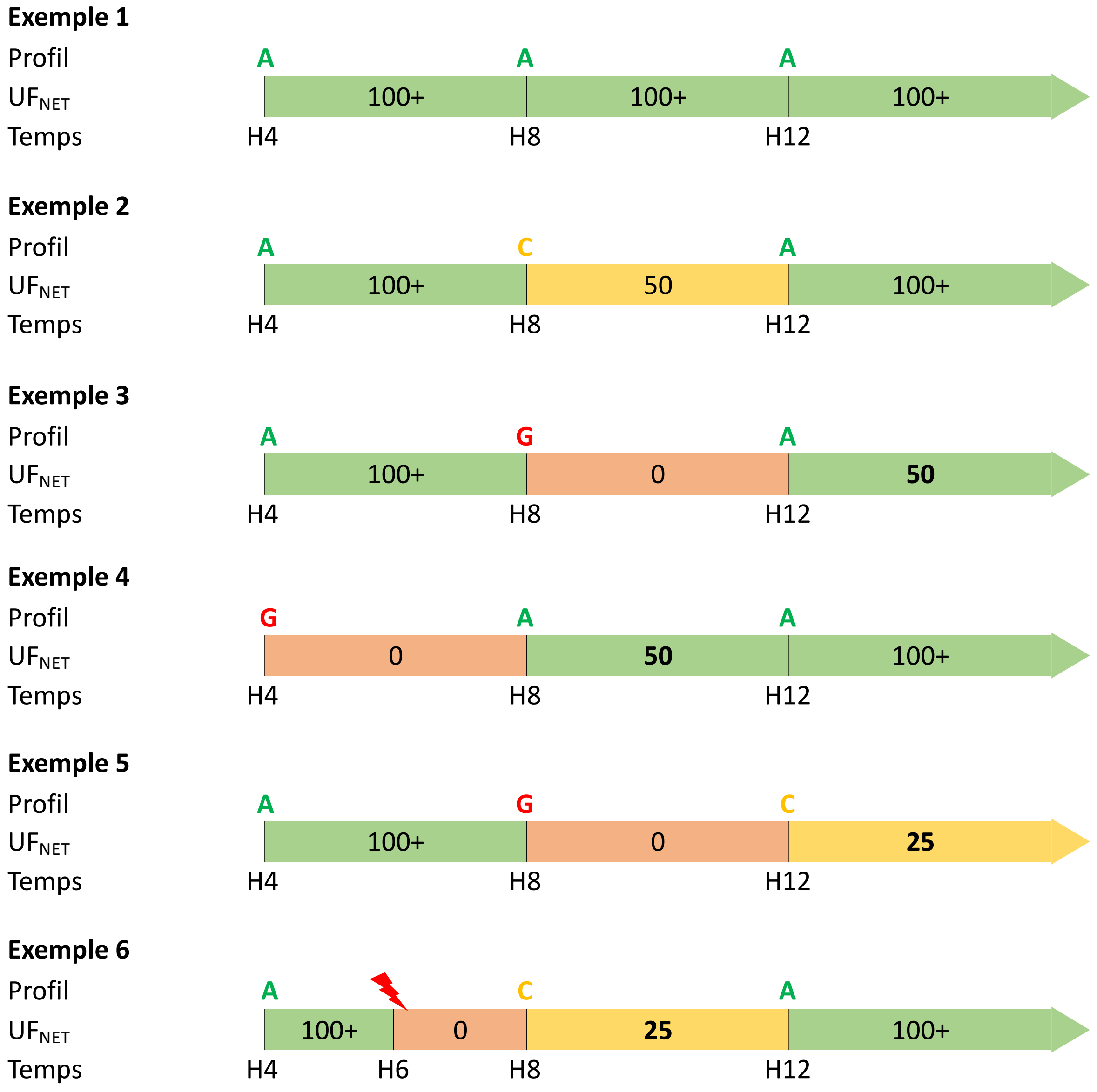

Figure 6. Exemples de gestion de l'UF_{NET} (bras interventionnel)
UF_{NET} : ultrafiltration nette ; éclair rouge : épisode d'instabilité hémodynamique.

Le résultat de l'évaluation réalisée par 4 heures – ou celui obtenu en cas d'événement hémodynamique déclencheur – sera reporté par l'infirmière ou le médecin en charge du patient dans un CRF papier présent au lit de patient, afin de rapporter l'évolution du profil hémodynamique des patients soumis au protocole. Les éléments de ce CRF papier (cahier IDE) seront ensuite colligés dans l'eCRF.

Une session de formation des personnels médicaux et paramédicaux portant sur le protocole, les manoeuvres posturales, l'utilisation du dispositif de monitorage du débit cardiaque, et les critères de validité des différentes

mesures (index cardiaque, PVC) sera proposée dans les centres participants avant leur ouverture. Ces éléments semblent tout à fait réalisables, étant déjà en place dans le centre coordinateur, qui a, par ailleurs, inclus plus de 60 patients dans des protocoles de recherche hémodynamique de complexité similaire (études EHOSS et PRELOAD-CRRT, [15] et https://clinicaltrials.gov/ct2/show/NCT03139123).

- **Description des contre-indications**

Les contre-indications à l'intervention sont celles où un état hypovolémique est certain, ou celles contre-indiquant la réalisation d'une manœuvre posturale. Elles mènent à la suspension transitoire ou permanente de la participation du patient à l'étude.

Contre-indications temporaires :

- Suspension transitoire de l'intervention avant H72, pendant une durée inférieure ou égale à 8 heures, du fait du transfert du patient au bloc opératoire, en radiologie, ou autre.
- Patient présentant une hémorragie active justifiant la transfusion de produits sanguins labiles, et dont la résolution est prévisible dans les 8 heures.
- Manœuvre posturale non réalisable de façon transitoire, soit moins de 8 heures (immobilisation d'un ou des membres inférieurs, ou syndrome compartimental intra-abdominal). Le syndrome compartimental abdominal est défini par la coexistence d'une hyperpression intra-abdominale supérieure à 20 mmHg et d'au moins une défaillance d'organe survenant *de novo*.

Contre-indications permanente ou prolongées :

- Accident vasculaire cérébral de cause ischémique ou hémorragique compliqué d'un coma et sous ventilation mécanique
- Manœuvre posturale non réalisable de façon permanente (amputation d'un ou des membres inférieurs, thrombose de la veine cave inférieure, immobilisation d'un ou des membres inférieurs, ou syndrome compartimental intra-abdominal durant plus de 8 heures). Le syndrome compartimental abdominal est défini par la coexistence d'une hyperpression intra-abdominale supérieure à 20 mmHg et d'au moins une défaillance d'organe survenant *de novo*.
- Hémorragie active justifiant la transfusion de produits sanguins labiles non contrôlée au bout de 8 heures
- Impossibilité de poursuivre le monitorage continu du débit cardiaque, pour des raisons techniques irréversibles

6.2 Stratégie de comparaison

- **Définition et description de la stratégie / procédure**

L'UF_{NET} dans le groupe contrôle devra refléter les pratiques actuelles de déplétion en EER continue, durant 72 heures à partir de l'inclusion, en la paramétrant entre 0 et 25 ml/h (soit une déplétion cumulée sur 24h s'échelonnant entre 0 ml et 600 ml par jour pendant les 3 premiers jours) [10]. L'heure d'initiation de l'intervention correspondra au H0 de l'étude. Au-delà de 72h, l'équipe en charge du patient sera libre de paramétrer l'UF_{NET} selon leur choix. Le groupe contrôle bénéficiera du même niveau de monitorage hémodynamique, mais sans consigne d'ajustement de l'UF_{NET}.

La procédure n'est pas appliquée en cas de transfert temporaire sur un plateau technique (scanner, radiologie interventionnelle), si sa durée est < 8h. En cas de transfert dans un autre service, la procédure expérimentale est définitivement arrêtée.

- **Justification de la stratégie / procédure choisie**

Nous avons choisi d'utiliser ces doses d'UF_{NET} afin de reproduire les données présentées dans l'étude IDEAL-ICU comparant une stratégie d'initiation précoce versus tardive de l'EER en réanimation. En effet, dans cette étude réalisée chez 488 patients avec choc septique et insuffisance rénale aiguë, les volumes d'UF_{NET} quotidienne était de 0 ml [écart interquartile 0–500] à J1 et 0 ml [0–1200] à J3 dans le bras précoce, et était comparable entre les 2 bras [10].

- **Description des contre-indications**

Il n'y a pas de contre-indications à la mise en place de stratégie contrôle.

6.3 *Circuit des produits*

Il n'y a pas de produit en lien avec la recherche.

6.4 *Insu*

Comme décrit ci-dessus, et du fait des caractéristiques intrinsèques de l'intervention, il ne sera pas possible de maintenir l'insu durant la durée de l'étude. En effet, d'une part l'UF_{NET} réglée sera visible par les cliniciens et laissera donc transparaître le groupe dans lequel le patient a été randomisé. Les cliniciens responsables de la prise en charge du patient devront par ailleurs appliquer le protocole de monitorage hémodynamique dans le bras interventionnel, intrinsèquement liée à l'intervention. D'autre part, les personnes collectant les données recueilleront entre autres l'UF_{NET} réglée quotidiennement, ainsi que les interventions induites par le protocole de monitorage hémodynamique dans le bras interventionnel.

6.5 *Traitements associés autorisés et interdits*

La prise en charge des patients de réanimation est complexe, multimodale, et fait intervenir un grand nombre d'évaluations et d'interventions, réalisées par des équipes pluridisciplinaires. Ceci pourrait théoriquement entrainer une grande hétérogénéité de prise en charge, pouvant impacter la mise en place du protocole et l'interprétation de ses résultats. Cependant, des recommandations françaises et internationales en réanimation permettent d'homogénéiser les pratiques en fondant une assise commune basée sur l'*evidence based medicine*. Dans le cas présent, celles-ci portent sur la gestion du patient en insuffisance rénale aiguë [8, 24], traité par EER [29], et en état d'insuffisance circulatoire aiguë [31], dans un contexte septique ou non [30]. Par ailleurs, certains éléments de prise en charge critique ont été rappelés pour cette étude et sont détaillés ci-dessous.

6.5.1 Remplissage vasculaire

Le remplissage vasculaire sera autorisé dans les 2 bras de l'étude. Un remplissage vasculaire est défini par un volume de soluté cristalloïde ≥ 250 ml administré en moins de 30 minutes. Le clinicien en charge du patient sera libre de réaliser un remplissage vasculaire, en particulier si celui-ci est motivé par l'existence d'une baisse de l'index cardiaque avec précharge dépendance. Dans le bras interventionnel, le remplissage vasculaire par soluté cristalloïde pour évaluation de la précharge dépendance sera recommandé en cas d'apparition d'un profil à risque avec risque de faux négatif à l'épreuve de lever de jambe passif (F et G). Dans le bras contrôle, le remplissage vasculaire pourra être utilisé selon les mêmes critères justifiant de la présence d'une précharge dépendance, évalué par l'existence d'une élévation du débit cardiaque au cours d'une manœuvre posturale. Ces pratiques d'évaluation de la réponse au remplissage vasculaire sont à la fois recommandées et courantes [31].

6.5.2 Gestion des amines vasopressives

La gestion des amines vasopressives et des traitements inotropes sera sous la responsabilité des cliniciens en charge du patient, et encadrée par les protocoles de soins spécifiques aux centres participants. Il sera recommandé aux équipes, en cas de survenue d'un épisode d'instabilité, de réaliser d'abord une évaluation hémodynamique avant de modifier la vitesse d'administration des amines. Par ailleurs, le protocole étant centré sur la gestion de l'UF_{NET}, les cliniciens seront libres d'ajouter ou d'ajuster les amines vasopressives, les inotropes, ou tout autre traitement à tropisme hémodynamique. Ces éléments seront rapportés dans l'eCRF.
Ces traitements sont à considérer comme des traitements concomitants et non des traitements non expérimentaux.

6.5.3 Gestion de l'EER

Les 2 groupes seront traités à l'inclusion par EER continue. Nous recommanderons aux cliniciens de poursuivre cette technique pendant 72h jusqu'à l'évaluation du critère de jugement principal. La méthode d'EER (continue ou intermittente) sera laissée à leur choix après 72h. Pour rappel, les recommandations françaises et

internationales proposent l'utilisation de l'EER continue chez le patient hémodynamiquement instable [8, 29]. La modalité de l'EER (convective ou diffusive) sera laissée au choix du clinicien, en recommandant toutefois de ne pas changer de technique au cours des 72 premières heures de l'étude, sans que cela soit considéré comme une violation de protocole.

6.5.4 Gestion des diurètiques

L'utilisation des diurétiques sera gérée de façon identique dans les 2 bras, selon les recommandations internationales KDIGO, dont nous rappelons les principaux éléments ci-dessous [8] :

- Les diurétiques sont recommandés dans la gestion de la surcharge hydrosodée (2C)
- Les diurétiques ne sont probablement pas recommandés pour accélérer la récupération rénale ou la cessation précoce de l'EER (2B) [38]

De plus, une analyse post-hoc de l'étude BEST avait montré une baisse de la performance prédictive de la diurèse (en ml par jour) pour prédiction de la cessation définitive de l'EER en réanimation lorsque les patients recevaient des diurétiques [39]. Dans l'étude IDEAL-ICU, aucune directive n'était donnée dans le protocole sur l'utilisation des diurétiques en EER continue [10]. Pour finir, dans l'étude pilote de Berthelsen et al., les diurétiques étaient arrêtés lorsque la diurèse était inefficace malgré leur utilisation à forte dose, et relayé par l'UF_{NET} [13]. Au total, nous recommanderons aux centres participants la cessation des traitements diurétiques chez les patients sous EER continue, quel que soit le groupe de randomisation.

6.5.5 Gestion de l'insuffisance respiratoire aiguë par oedème pulmonaire hydrostatique dans le groupe contrôle

En cas d'apparition de critères francs en faveur d'une insuffisance respiratoire aiguë secondaire à un œdème pulmonaire hydrostatique, il sera possible d'appliquer une dose d'UF_{NET} > 25 ml/h pendant 4 à 8 heures dans le groupe contrôle et une UF_{NET} pour obtenir une balance entrées/sorties négative dans le groupe interventionnel, jusqu'à réévaluation dans les 4 heures. Le débit réglée est laissé à la discrétion du clinicien. Nous définissons l'insuffisance respiratoire aiguë de cause hydrostatique comme l'association de :

- Une survenue rapide (< 24h)
- Une polypnée > 25 /min
- L'apparition ou l'aggravation d'une hypoxémie
- Une radiographie des poumons montrant un infiltrat bilatéral ou une échographie pulmonaire montrant l'apparition de lignes B échographiques
- Au moins un critère échocardiographique parmi les suivants : vitesse maximale de l'onde E supérieure à 1.5 fois la vitesse maximale de l'onde A chez un patient de plus 65 ans, temps de décélération de l'onde E < 150 ms, vitesse maximale de l'onde E > 1 m/s, rapport E/E' > 12.

6.5.6 Critères de qualité du monitorage hémodynamique

Les capteurs de pression artérielle et veineuse seront fixés à l'aide de sparadrap au bras du patient, au niveau du point phlébostatique. Cela permet de prévenir l'impact des changements de position du patient sur les mesures.

La pression veineuse centrale est systématiquement mesurée en décubitus à 0°, en fin d'expiration et après rinçage de la ligne veineuse (fast flush test) objectivant une réponse satisfaisante.

La calibration du dispositif de monitorage du débit cardiaque sera réalisée toutes les 4 heures avant l'évaluation hémodynamique, selon la technique de thermodilution transpulmonaire. Elle sera aussi réalisée en cas de survenue d'un épisode d'instabilité hémodynamique. Elle consiste en l'injection de 3 bolus de 15 ml de sérum salé froid sur la voie distale d'une voie veineuse centrale située en territoire cave supérieur. La température de l'injectat est détectée par une thermistance située sur la voie d'injection. Le capteur de température situé sur le cathéter artériel placé dans une artère fémorale permet la détermination de la valeur absolue du débit cardiaque selon la méthode de Stewart-Hamilton (index cardiaque par thermodilution). Trois injections sont nécessaires afin de moyenner la valeur de débit cardiaque obtenu. Le débit cardiaque est ensuite indexé à la surface corporelle. Le dispositif permet à l'aide d'algorithmes intégrés la détermination d'autres paramètres d'intérêt tel que l'index cardiaque continu (index cardiaque par analyse du contour de l'onde de pouls), l'eau pulmonaire extravasculaire indexée, ou l'index de perméabilité vasculaire pulmonaire.

En cas de dysfonction d'un des dispositifs de monitorage, les investigateurs recommandent le changement dès que possible du ou des cathéters défectueux.

7 ORGANISATION GÉNÉRALE

7.1 Calendrier de l'étude

Durée de la période d'inclusion : 24 mois
Durée de la participation pour chaque patient/sujet sain : 90 jours ± 7 jours
Durée totale de l'étude : 27 mois
Début des inclusions : second trimestre 2020

Dès la première inclusion pour les recherches interventionnelles de catégorie 1, le promoteur doit informer sans délai le CPP et l'ANSM de la date effective de démarrage de l'étude (date effective de démarrage = date de signature du consentement par la première personne qui se prête à la recherche).

La date de fin d'étude sera transmise par le promoteur au CPP et à l'ANSM dans un délai de 90 jours. La date de fin de la recherche correspond au terme de la participation de la dernière personne qui se prête à la recherche, ou le cas échéant, au terme défini dans le protocole.

7.2 Schéma général

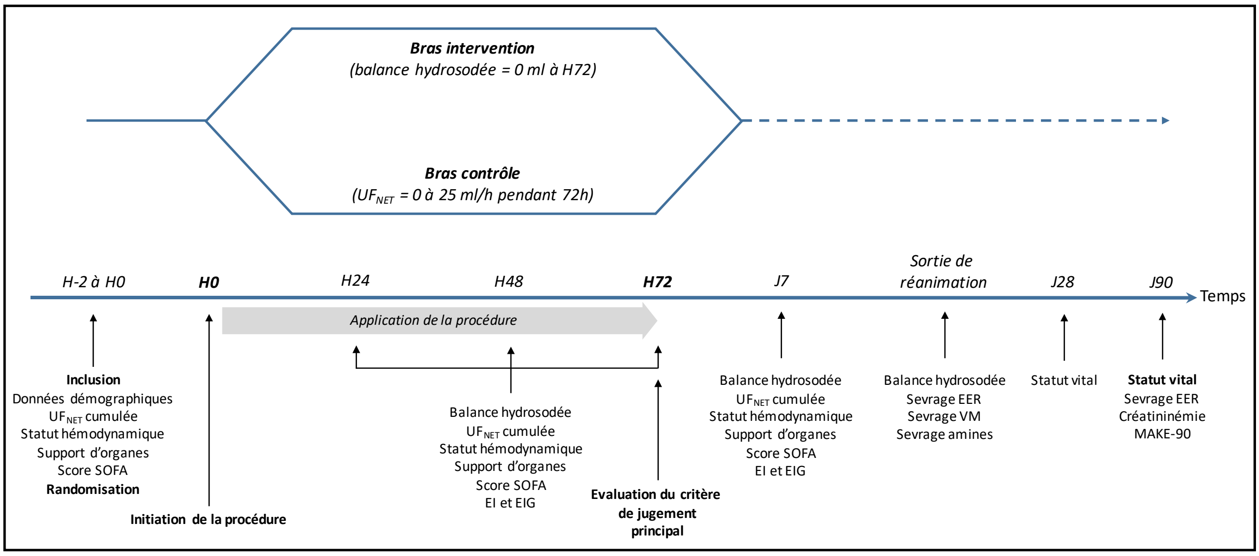

Figure 7. Schéma du déroulé de l'étude.
EER : épuration extra-rénale ; EI : événement indésirable ; EIG : événement indésirable graves ; MAKE-90 : major adverse kidney events at day 90 ; SOFA : sepsis-related organ failure assessment score [28] ; UF_{NET} : ultrafiltration nette ; VM : ventilation mécanique.

7.3 Tableau récapitulatif

ETAPES	*V1 Inclusion*	*V2 à V4 Hospitalisation*	*V5 Hospitalisation*	*V6 Hospitalisation*	*V7*	*V8 Fin d'étude*
Moment / ***Actions***	***H-2 – H0 ± 2h (J1)***	***H24, H48 et H72 ± 2h***	***J7 ± 0j***	***Sortie de réanimation***	***J28 ± 2j***	***J90 ± 7j***
Screening<br>- Critères d'inclusion<br>- Critères de non inclusion	*X*					
Consentement éclairé	*X*					
Email d'inclusion	*X*					
Randomisation	*X*					
Données démographiques<br>- Âge<br>- Genre<br>- Poids à l'admission, taille<br>- Catégorie d'admission<br>- Origine de l'admission<br>- Date d'admission<br>- Code diagnostic<br>- Présence d'un sepsis<br>- Score IGS-II [40]	*X*					
Antécédents<br>- Créatinine de base<br>- Comorbidités	*X*					
Balance entrées/sorties et EER à l'inclusion<br>- Poids à l'inclusion<br>- Balance entrées/sorties cumulée entre admission et inclusion	*X*					

- Diurèse des dernières 24h avant l'inclusion<br>- Date d'initiation de l'EER						
Evaluation hémodynamique<br>- Marbrures (oui/non)<br>- FC<br>- PAS, PAD, PAM<br>- IC et FC<br>- PVC<br>- IEPEV et PVPI<br>- VVS et ΔPP	*X*	*X*	*X (sauf marbrures)*			
Surveillance de la ventilation<br>- Invasive (oui/non)<br>- FiO_2 ou débit d'O_2<br>- PaO_2	*X*	*X*	*X*			
Score SOFA [28]<br>- Créatininémie<br>- Plaquettes<br>- Bilirubine totale<br>- Echelle de coma de Glasgow	*X*	*X*	*X*			
Autres analyses biologiques<br>Hémoglobine	*X*	*X*	*X*			
Traitement en cours<br>- Vasopresseurs (type, dose)<br>- Inotropes (type, dose)<br>- Diurétique (type, dose)	*X*	*X*	*X*			
Epuration extra-rénale<br>- Mode (CVVH, CVVD, CVVHDF)<br>- Dose (UF totale ou débit de dialysat)	*X*	*X*	*X*			
Statut hémodynamique (par 4h)<br>- PAM, IC, PVC, FC<br>- Acide lactique dans les 8h précédent l'évaluation<br>- Profil hémodynamique (lettre d'identification)<br>- UF_{NET} réglée	*X*	*X*				
Balance entrées/sorties (par 4h)<br>- Poids (1x par jour)<br>- Médicaments IV, par 4h<br>- Remplissage vasculaire, par 4h<br>- Transfusion de PSL, par 4h<br>- Nutrition artificielle, par 4h<br>- UF_{NET} cumulé des 24h<br>- Diurèse des 24h<br>- Débit des drains sur 24h	*X*	*X*				
Sortie de réanimation<br>- Poids<br>- Balance entrées/sorties<br>- Créatininémie<br>- Sevrage de l'EER en réanimation<br>- Date du sevrage des amines vasopressives*<br>- Date de l'intubation si intubé<br>- Date de l'extubation si intubé†<br>- Date de sortie de réanimation				*X*		
MAKE-90 et devenir<br>- Sevrage de l'EER<br>- Date du sevrage de l'EER						*X*

- Créatinine plasmatique (si sevré de l'EER)<br>- Date de sortie d'hospitalisation						
Statut vital<br>- Patient vivant ou décédé<br>- Date du décès		*X à H72*			*X*	*X*
Recherche des événements indésirables		*X*	*X*	*X*	*X*	*X*
Recherche des événements indésirables graves		*X*	*X*	*X*	*X*	*X*

* : Sevrage défini par une période libre d'amines vasopressives (noradrénaline, adrénaline) ≥ 48h. Dernier sevrage en cas d'administration d'amines répétées.
† : Sevrage défini par une période sans ventilation mécanique invasive ≥ 48h. Dernier sevrage en cas d'intubation répétées.

Catégorie d'admission : médical, chirurgical programmée, chirurgical en urgence
Code diagnostic : selon codage diagnostique du score APACHE (Acute Physiology, Age, and Chronic Health Evaluation) [41]
Sepsis : défini selon les critères de la conférence de consensus SEPSIS-3 [42]

CVVD : hémodialyse veino-veineuse continue ;CVVH : hémofiltration veino-veineuse continue ; CVVHDF : hémodiafiltration veino-veineuse continue ; ΔPP : variation respiratoire de la pression pulsée ; EER : épuration extra-rénale ; FC : fréquence cardiaque ; IC : index cardiaque ; IEPEV : eau pulmonaire extravasculaire indexée ; IGS-II : score de gravité simplifiée ; PAD : pression artérielle diastolique ; PAM : pression artérielle moyenne ; PAS : pression artérielle systolique ; PSL : produit sanguin labile ; PVC : pression veineuse centrale ; UF_{NET} : ultrafiltration nette ; VVS : variation respiratoire du volume d'éjection systolique

7.4 Déroulement de l'étude

7.4.1 Screening – Pré-inclusion

Il n'est pas envisagé de réaliser de visite de pré-inclusion, du fait de la population étudiée (population de réanimation).

Le screening sera réalisé pluri-quotidiennement dans les centres participants par l'équipe médicale ou celle de recherche clinique. Il n'est pas nécessaire de réaliser d'examen biologique afin d'étayer le screening. L'examen clinique du screening reprend les éléments des critères d'inclusion décrits plus haut.

Les patients (ou leur proche) seront informés par un médecin investigateur de l'existence du protocole. Toute explication nécessaire à la bonne compréhension de l'étude sera donnée au patient (ou à leur proche), ainsi qu'une lettre d'information expliquant les objectifs et le déroulement du protocole. Le praticien remettra également un formulaire de consentement au patient en double exemplaire. Il sera laissé au patient le délai de réflexion nécessaire pour décider de sa participation à l'étude.

Le formulaire de consentement doit être signé avant la réalisation de tout examen clinique ou paraclinique nécessité par la recherche. Si le patient (ou le proche) donne son accord de participation, le volontaire et l'investigateur datent et signent nominativement (nom et prénom en clair) deux exemplaires du formulaire de consentement. L'un est conservé par le patient, l'autre est conservé dans le classeur investigateur de l'étude.

En cas d'impossibilité d'obtenir l'accord du patient du fait de son état de santé (coma, anesthésie générale, ventilation mécanique), et dans l'impossibilité de contacter un proche afin de lui remettre les informations en lien avec l'étude, nous demanderons au Comité de Protection des Personnes l'autorisation de mettre en place pour cette étude une procédure d'inclusion en urgence (décrite dans la section 13). Celle-ci sera obligatoirement suivie de l'obtention du consentement du patient ou de celui de son proche dès que possible.

7.4.2 Visite d'inclusion / Randomisation / H-2 à H0 (ou J1), en réanimation

Immédiatement après l'inclusion du patient, la randomisation sera réalisée en ligne sur la plateforme Ennov EDC et le bras d'étude communiqué alors à l'équipe en charge du patient. L'intervention devra alors être initiée dans les 2 heures suivants l'inclusion.

Bien que nous fassions la distinction entre inclusion (H-2) et initiation de l'intervention (H0), nous considérons ces 2 temps comme une seule et même visite. Cette distinction est nécessaire car les données de balance entrées/sorties et d'UF_{NET} seront rapportées à partir de H0, quand les autres variables décrites ci-dessous pourront être rapportées durant la période allant de H-2 à H0. Cette visite correspond au J1 de l'étude.

Les données démographiques suivantes seront relevées par l'interrogatoire du patient, de sa personne de confiance ou de son dossier médical :
- Âge
- Genre
- Poids à l'admission et le jour de l'inclusion, taille
- Comorbidités (hypertension artérielle, diabète, artériopathie oblitérante des membres inférieurs, accident vasculaire cérébral (transitoire ou non), insuffisance rénale chronique, insuffisance cardiaque ou respiratoire, cirrhose, immunodépression)
- Motif et code diagnostic motivant le séjour en réanimation, date, origine (urgences, service d'hospitalisation, domicile) et type d'admission (médicale, chirurgicale urgente ou programmée)
- Code diagnostic en utilisant comme base le thesaurus de code du score APACHE (Acute Physiology, Age and Chronic Health Evaluation) [41].
- Existence d'un sepsis au moment de l'inclusion, par l'identification des critères définis par la conférence de consensus SEPSIS-3 [42]. En particulier, l'augmentation du SOFA de +2 points comme défini par la conférence de consensus se fera par rapport à l'état physiologique basal (chronique) du patient, à l'aide des données recueillies pour l'étude.
- Score IG2 à l'admission [40]
- Fonction rénale avant le séjour en réanimation, dite « pré-morbide » par la recherche d'une créatininémie mesurée antérieurement à l'hospitalisation actuelle en réanimation (créatinémie la plus basse dans un délai se situant entre 6 mois et 7 jours avant l'admission index). Si la créatinine de base est absente, elle sera estimée de façon rétrograde sur la base d'un débit de filtration glomérulaire estimée par la formule MDRD (Modification of Diet in Renal Disease) égale à 75 ml/min/1.73m^2 [43]

L'inclusion est complétée par la récupération des données de balance entrées/sorties antérieure à l'initiation de l'intervention et celle en rapport avec l'EER :
- Poids à l'inclusion. Le poids à l'inclusion permet la stratification de la randomisation, afin de juger de l'existence d'une surcharge hydrosodée acquise en réanimation, en le comparant au poids à l'admission en réanimation.
- Date et heure d'initiation de l'EER

Nous collectons aussi les données hémodynamiques, donc celles données par le dispositif de monitorage hémodynamique continu, après calibration du dispositif. Le monitorage du débit cardiaque sera calibré au moment de l'inclusion par une triple injection d'un bolus de 15 ml de cristalloïde froid avec mesure de de thermodilution transpulmonaire. Les capteurs de pression artérielle et de PVC seront fixés au bras du patient afin de s'assurer de leur positionnement en regard du point phlébostatique. Les lignes de pression seront rincées et la réponse du cathéter sera évaluée par le *fast flush test*. La PVC sera mesurée en position allongée à 0°. Une première manœuvre posturale sera réalisée à l'inclusion avec mesure de l'index cardiaque par analyse de l'onde de pouls avant l'épreuve et sa valeur maximale pendant l'épreuve :
- Présence ou extension de marbrures : examen cutanée des genoux et de l'abdomen à la recherche de marbrures
- Fréquence cardiaque
- Pressions artérielles moyenne, systolique et diastolique
- Pression veineuse centrale, mesurée selon les dispositions de validité décrits plus haut
- Index cardiaque par thermodilution
- Index cardiaque continu (ICC) par analyse de l'onde de pouls avant l'épreuve de lever de jambe et sa valeur maximale pendant l'épreuve
- Eau pulmonaire extravasculaire indexée et index de perméabilité vasculaire pulmonaire
- Variations respiratoires de la pression pulsée et du volume d'éjection systolique

Les autres modes de suppléances d'organes sont aussi décrits :

- Patient sous ventilation mécanique invasive (intubé ou trachéotomisé)
- FiO2 ou débit d'oxygène, selon le mode d'administration. Le débit d'oxygénothérapie sera converti dans un second temps en équivalent de FiO_2 à l'aide d'une table de conversion [44].

Les éléments nécessaires au calcul du score de SOFA sont aussi colligés à l'inclusion [28]. Le score est présenté en annexe du protocole. Ce score est très largement utilisé dans les études de réanimation car il permet d'estimer la gravité des défaillances de 6 systèmes physiologiques (hémodynamique, respiratoire, hépatique, hématologique, rénal et neurologique), et d'observer leurs évolutions au cours du temps :

- Score de coma de Glasgow au plus proche de l'inclusion, ou précédant l'intubation si patient sous anesthésie générale. Le score évalue l'état d'éveil par l'évaluation de la motricité, de la parole et de l'ouverture des yeux à divers niveaux de stimulus.
- Créatininémie, plaquettes et bilirubinémie.

Les paramètres permettant de juger du degré d'hémoconcentration des patients, ainsi que leur statut sur le plan acido-basique seront rapportés :

- Hémoglobinémie

Nous reporterons aussi les traitements reçus par le patient au moment de l'inclusion :

- Type et dose d'amines vasopressives, rapportés en dose équivalente de noradrénaline (selon un rapport 1 pour 1 pour l'adrénaline), et exprimé en µg/kg/min au moment de l'inclusion
- Type et dose d'inotrope, exprimé en µg/kg/min au moment de l'inclusion
- Type et dose de diurétiques de l'anse, rapporté en dose cumulée sur les 24h précédent l'inclusion
- Eléments de prescription d'EER au moment de l'inclusion : modalité d'épuration (CVVH, CVVD ou CVVHDF), dose d'EER (UF total ou débit de dialysat)

7.4.3 Visites de suivi de H0 à H72, en réanimation

L'heure de début de l'intervention correspond au H0 de l'étude. De H0 à H72, nous rapporterons les éléments suivants décrits plus haut et listés ci-dessous selon les mêmes modalités qu'à l'inclusion. Les bornes de début et de fin des 24h correspondent à [H0 à H24],]H24 à H48], et]H48 à H72]. Ils seront rapportés au moment le plus proche correspondant respectivement à 24h, 48h et 72h de l'inclusion, bornant ainsi la durée de l'intervention (avec une précision de ± 2 heures) :

- Variables hémodynamiques
- Données de ventilation et respiratoire
- Données nécessaires au calcul du score SOFA
- Données d'hémoconcentration
- Traitement vasopresseurs, inotropes et diurétiques administrés
- Caractéristiques de la prescription d'EER
- Poids
- Survenue d'un évènement indésirable grave ou non
- Statut vital à H72 (pour analyse intermédiaire de sécurité)

De H0 à H72, il sera aussi recueilli, selon les modalités du protocole de monitorage hémodynamique défini par le bras de randomisation, soit toutes les 4h (avec une précision de ± 1h) :

13. Paramètres hémodynamiques :
    - Fréquence cardiaque
    - Pression artérielle moyenne
    - Pression veineuse centrale
    - Index cardiaque par thermodilution
    - Acide lactique (valeur mesurée dans les 8h <u>précédant</u> l'évaluation)
    - Le résultat de la manœuvre posturale le cas échéant (index cardiaque par analyse de l'onde de pouls avant et sa valeur maximale pendant l'épreuve)

    - Survenue d'épisodes d'instabilité hémodynamique (apparition ou extension de marbrures, hypotension avec PAM < 65 mmHg, FC > 120 avec rythme sinusal, ou baisse de l'index cardiaque ≥ 15%)

14. Application du protocole :
    - UF_{NET} réglée
    - Résultat de l'évaluation hémodynamique dans le bras interventionnel, reporté à l'aide d'une lettre-code.
15. Balance entrées/sorties
    - Volume total de médicaments administrés par voie intraveineuse, par 4h
    - Volume total de remplissage vasculaire, par 4h
    - Volume total de transfusion de produit sanguin labile, par 4h
    - Volume total de nutrition entérale et parentérale, par 4h
    - Volume total d'UF_{NET} cumulée, par 4h
    - Volume de diurèse, par 4h
    - Volume total des drains, par 4h
16. Episode d'instabilité hémodynamique : nous collecterons les données définissant et caractérisant les épisodes d'instabilité hémodynamique comme décrits plus haut. Celles-ci sont :
    - Définition de l'épisode (hypotension, tachycardie, marbrures, baisse de l'IC)
    - Fréquence cardiaque
    - Pression artérielle moyenne
    - Pression veineuse centrale
    - Index cardiaque continu (pas de calibration)
    - UF_{NET} en cours au moment de l'épisode
    - Le résultat de la manœuvre posturale le cas échéant (index cardiaque par analyse de l'onde de pouls avant et sa valeur maximale pendant l'épreuve). Ce résultat est simplifié en 2 codes : jaune ou rouge
    - UF_{NET} réglée au décours de l'épisode

Les données de balance entrées/sorties, une fois additionnée, permettront le calcul de la balance entrées/sorties à H72, avec une précision de ± 2h. les éléments essentiels du protocole d'évaluation hémodynamique du groupe observationnel seront colligés au lit du patient à l'aide d'un CRF papier. Celui-ci permettra ensuite l'entrée de ces données dans le eCRF de l'étude.

7.4.4 Visite à J7, en réanimation

A J7, nous rapporterons les éléments suivants déjà décrits plus haut et listés ci-dessous selon les mêmes modalités qu'à l'inclusion. Ils seront rapportés au moment le plus proche correspondant J7 de l'inclusion. Si le patient a déjà quitté la réanimation ou est décédé, l'entrée sera complétée comme manquante.

- Variables hémodynamiques
- Données de ventilation et respiratoire
- Données nécessaires au calcul du score SOFA
- Données d'hémoconcentration
- Traitement vasopresseurs, inotropes et diurétiques administrés
- Caractéristiques de la prescription d'EER, dont l'UF_{NET}, si le patient est toujours traité par EER
- Poids
- Survenue d'un évènement indésirable grave ou non

7.4.5 Visite de sortie de réanimation

A la sortie de réanimation, nous collecterons :

- Poids mesuré le jour de la sortie. Ce poids nous servira à estimer la balance hydrosodée entre l'inclusion et le J7 pour les patients, la collection des entrées et sorties systématique pendant 7 jours continus nous exposant à un risque important de valeurs manquantes.
- Créatininémie mesurée le jour de la sortie (avec une tolérance de ± 24h)
- Sevrage de l'EER réalisée en réanimation, identifié par le non-recours à un service de dialyse après la sortie. Plus précisément, le sevrage de l'EER est défini comme le non-recours pendant 48h ou plus à

l'EER que ce soit en technique continue ou intermittente. En cas de sevrages multiples, c'est la dernière date de sevrage réussie qui est collectée (sevrage définitif)
- Date du sevrage des amines vasopressives, identifiée comme la date à laquelle les amines vasopressives ont été pour la dernière fois arrêtées pendant au moins 48h sans discontinuer. En cas de sevrages multiples des amines, c'est la date du dernier sevrage réussi qui est retenue
- Dates de l'intubation et de l'extubation si intubé. Date de l'extubation identifiée comme la date à laquelle le patient a été extubé (ou arrêt de la ventilation mécanique chez le patient trachéotomisé) pour la dernière fois sans nouveau recours à la ventilation mécanique invasive pendant 48h au moins En cas d'extubations multiples, c'est la date de la dernière extubation réussie qui est retenue
- Date de sortie de réanimation
- Survenue d'un évènement indésirable grave ou non

7.4.6 Visite de suivi à J28, par appel téléphonique

A J28, nous rapporterons le statut vital du patient, et la date de son décès le cas échéant. Le statut vital pourra être obtenu par l'appel du patient ou de son proche de confiance. En cas de non réponse aux appels, nous interrogerons le dossier médical informatisé. Si le patient est vivant, ce contact téléphonique sera l'occasion de vérifier que le patient a bien en sa possession l'ordonnance de mesure la créatinémie à J90. La visite pourra être faite avec une précision de ± 2 jours.
Survenue d'un évènement indésirable grave ou non

7.4.7 Visite de suivi à J90, par appel téléphonique et biologie de ville

A J90, nous rapporterons :
- Le sevrage effectif de l'EER, identifié par la poursuite de l'EER (EER le jour de l'évaluation si patient sous technique continue, ou EER réalisée dans un intervalle de ± 2 jours dans le cas de techniques intermittentes)
- La date de sevrage de l'EER, identifiée comme la date à laquelle la dernière séance d'EER a été réalisée
- Une mesure de la créatinine plasmatique réalisée en laboratoire chez les patients sevrés de l'EER
- Le statut vital (patient vivant ou décédé)
- La date du décès si patient décédé
- La date de sortie d'hospitalisation
- Survenue d'un évènement indésirable grave ou non

En cas de retour au domicile des patients, il leur sera remis ou communiqué une ordonnance afin de faire réaliser en laboratoire de ville une mesure de la créatininémie plasmatique. La mesure de la créatinine à distance d'une épisode d'insuffisance rénale aiguë est recommandée par les experts afin de ne pas méconnaître une insuffisance rénale chronique se développant au décours d'une insuffisance rénale aiguë ayant requis même transitoirement un traitement par épuration extra-rénale [24]. Le résultat du dosage de la créatinine sera communiqué aux investigateurs du centre participant, ainsi qu'au médecin traitant du patient.

Nous interrogerons de plus les éléments du dossier médical afin de d'identifier le statut vis-vis-du sevrage de l'EER. Les patients ou leurs proches pourront être aussi contactés par téléphone afin de confirmer le sevrage de l'EER. Le contact sera pris par le centre participant ayant inclus le patient. La créatinine à J90 ne sera pas mesurée chez les patients non sevrés de l'EER.

A ce moment de l'étude, nous estimons qu'environ 40% des patients seront décédés [10]. Le statut vital pourra éventuellement être confirmé par l'appel de leur proche ou l'interrogatoire du dossier médical informatisé.

L'ensemble de ces données permettra de connaître l'incidence des événements adverses rénaux majeurs à J90 (ou *major adverse kidney events* - MAKE), un critère composite (créatininémie > 2 x la créatinine de base, dépendance à l'EER au décours de l'insuffisance rénale aiguë, ou décès), de plus en plus souvent rapporté par les études de réanimation néphrologique [27].

Nous récupérerons aussi les données administratives en lien avec le séjour index (date de sortie de réanimation et d'hospitalisation). Nous accepterons que le recueil de la visite à J90 soit fait avec une précision de ± 7 jours. La fin de la recherche correspond à J90 + 7 jours.

7.4.8 Echantillons biologiques

Les prélèvements sanguins nécessaires au monitorage des fonctions d'organe et au calcul du score font partie du soin courant, leurs mesures étant quasi-systématique chez les patients en défaillance multiviscérale. Les prélèvements seront envoyés dans les laboratoires rattachés aux centres participants, et seront envoyés et traités selon la filière de traitement habituel du centre.

La mesure systématique de l'acide lactique toutes les 2 – 8 heures dans le cadre du monitorage hémodynamique est habituelle dans le cadre du monitorage des patients les plus graves en réanimation (ceux avec défaillance multiviscérale). L'acide lactique se mesure sur un prélèvement artériel de 2 ml, prélevé sur la ligne artérielle du patient. Le prélèvement est ensuite acheminé au laboratoire du centre participant et traité selon les mesures habituelles. En cas de mesure de la lactatémie datant de moins de 8h, celle-ci ne sera pas renouvelé dans le cadre du monitorage.

La mesure de la créatinine plasmatique à J90 de l'inclusion est à considérer comme du soin courant, le suivi néphrologique et la surveillance de la fonction rénale étant recommandés par la conférence d'experts française au décours d'un épisode d'insuffisance rénale aiguë afin de ne pas méconnaitre le développement d'un insuffisance rénale chronique [24]. Il sera alors nécessaire pour le patient, s'il l'accepte, de se rendre dans un laboratoire de ville afin de réaliser un prélèvement sanguin veineux (1 tube de 5 ml), et le résultat envoyé au centre investigateur. Cette analyse sera réalisée en fin d'étude. Le résultat de ce prélèvement sera aussi communiqué au médecin traitant.

7.4.9 Distinction soins et recherches

La quasi-totalité des soins prodigués et des données collectées dans ce protocole font partie des soins courants apportés aux patients de réanimation. L'application du protocole de surveillance hémodynamique avec l'identification de profil à risque est la seule procédure ajoutée du fait de la recherche.

Procédures effectuées au cours de la recherche	***Procédures effectuées dans le cadre du soin***	***Procédures ajoutées du fait de la recherche***
Données démographiques<br>- Âge<br>- Genre<br>- Poids à l'admission, taille<br>- Catégorie d'admission<br>- Origine de l'admission<br>- Date d'admission<br>- Code diagnostic<br>- Présence d'un sepsis<br>- Score IGS-II	*X*	
Antécédents<br>- Créatinine de base<br>- Comorbidités	*X*	
Balance entrées/sorties et EER à l'inclusion<br>- Poids à l'inclusion<br>- Balance entrées/sorties cumulée entre admission et inclusion<br>- Diurèse des dernières 24h avant l'inclusion<br>- Date d'initiation de l'EER<br>- UF_{NET} cumulée entre début d'EER et inclusion	*X*	

Evaluation hémodynamique<br>- Marbrures (oui/non)<br>- FC<br>- PAS, PAD, PAM<br>- IC et FC<br>- PVC<br>- IEPEV et PVPI<br>- VVS et ΔPP	*X*	
Surveillance de la ventilation<br>- Invasive (oui/non)<br>- FiO2 ou débit d'O2<br>- PaO2	*X*	
Score SOFA<br>- Créatininémie<br>- Plaquettes<br>- Bilirubine totale<br>- Echelle de coma de Glasgow	*X*	
Autres analyses biologiques<br>- Hémoglobine	*X*	
Traitement en cours<br>- Vasopresseurs (type, dose)<br>- Inotropes (type, dose)<br>- Diurétique (type, dose)	*X*	
Epuration extra-rénale<br>- Mode (CVVH, CVVD, CVVHDF)<br>- Dose (UF totale ou débit de dialysat)	*X*	
Statut hémodynamique (par 4h)<br>- PAM, IC, PVC<br>- Acide lactique<br>- UF_{NET} réglée	*X*	
Protocole hémodynamique (par 4h)<br>- Profil hémodynamique (lettre d'identification)<br>- Ajustement de l'UF_{NET}		*X*
Balance entrées/sorties<br>- Poids (1x par jour)<br>- Médicaments IV, par 4h<br>- Remplissage vasculaire, par 4h<br>- Transfusion de PSL, par 4h<br>- Nutrition artificielle, par 4h<br>- UF_{NET} cumulé des 4h<br>- Diurèse des 4h<br>- Débit des drains sur 4h	*X*	
Sortie de réanimation<br>- Poids<br>- Balance entrées/sorties<br>- Créatininémie<br>- Sevrage de l'EER en réanimation<br>- Date du sevrage des amines vasopressives<br>- Date de l'intubation si intubé<br>- Date de l'extubation si intubé<br>- Date de sortie de réanimation	*X*	

MAKE-90 et devenir<br>- Sevrage de l'EER<br>- Date du sevrage de l'EER<br>- Créatinine plasmatique<br>- Date de sortie d'hospitalisation	*X*	
Statut vital<br>- Patient vivant ou décédé<br>- Date du décès		*X*
Recherche des événements indésirables		*X*
Recherche des événements indésirables graves		*X*

7.5 Règles d'arrêt temporaire ou définitif

- *Arrêt de participation d'une personne à la recherche :*

Les sujets pourront retirer leur consentement et demander à sortir de l'étude à n'importe quel moment et quelle qu'en soit la raison. En cas de sortie prématurée, l'investigateur doit en documenter les raisons de façon aussi complète que possible.

L'investigateur pourra interrompre temporairement ou définitivement la participation d'un sujet à l'étude pour toute raison qui servirait au mieux les intérêts du sujet en particulier en cas d'événements indésirables graves.

En cas de sujet perdu de vue, l'investigateur mettra tout en œuvre pour reprendre contact avec la personne, et tentera si possible de recueillir la raison de la perte de vue.

En cas de retrait de consentement ou en cas d'identification d'un critère de sortie prématurée, les données recueillies jusqu'à la date du retrait/sortie seront analysées. Les patients retirant leur consentement avant H72 de participation ne seront pas inclus dans l'analyse finale ; les patients retirant leur consentement après H72 de participation seront inclus dans l'analyse finale (évaluation du critère de jugement principal réalisé et analysable).

- *Arrêt d'une partie ou de la totalité de la recherche :*

L'étude peut être interrompue prématurément en cas de survenue d'événements indésirables inattendus, graves nécessitant une revue du profil de la stratégie. De même, des événements imprévus ou de nouvelles informations relatives à la méthode d'investigation, au vu desquels les objectifs de l'étude ne seront vraisemblablement pas atteints, peuvent amener le promoteur à interrompre prématurément l'étude.

Les Hospices Civils de Lyon se réservent le droit d'interrompre l'étude à tout moment, s'il s'avère que les objectifs d'inclusion ne sont pas atteints.

En cas d'arrêt prématuré de l'étude pour des raisons de sécurité, l'information sera transmise par le promoteur sans délai à l'ANSM et au CPP par un fait nouveau et une mesure urgente de sécurité.

7.6 Collection d'échantillons biologiques

Il n'est pas prévu de collection d'échantillon pour cette recherche.

8 ÉVALUATION DE LA SÉCURITÉ

8.1 Définitions

Selon l'article R1123-46 du code de la santé publique

8.1.1 Evénement indésirable (EI)

Toute manifestation nocive survenant chez une personne qui se prête à une recherche impliquant la personne humaine que cette manifestation soit liée ou non à la recherche.

8.1.2 Evénement ou effet indésirable grave (EIG)

Tout événement ou effet indésirable :

- qui entraîne la **mort** ; ou
- qui **met en danger la vie de la personne** qui se prête à la recherche ; ou
- qui nécessite une **hospitalisation ou une prolongation d'hospitalisation** ; ou
- qui provoque une **incapacité ou un handicap importants ou durables** ; ou
- qui se traduit par une **anomalie ou une malformation congénitale** ; ou
- tout **autre événement médicaux important** ne répondant pas aux qualifications énumérées ci-dessus :
    - o mais pouvant être considéré comme **« potentiellement grave »** notamment certaines anomalies biologiques ;
    - o ou **événement médicalement pertinent selon le jugement de l'investigateur** ;
    - o ou encore **un événement nécessitant une intervention médicale** pour prévenir l'évolution vers un des états précités.

L'expression "mettre la vie en danger" est réservée à une menace vitale immédiate, au moment de l'événement indésirable, et ce, indépendamment des conséquences qu'aurait une thérapeutique correctrice ou palliative.

Certaines circonstances nécessitant une hospitalisation ne relèvent pas du critère de gravité. Se référer au paragraphe 8.2.3 pour les événements indésirables graves ne nécessitant pas une notification immédiate au promoteur.

8.1.3 Effet indésirable (EI)

Toute réaction nocive et non désirée due à la recherche impliquant une personne humaine dans sa globalité.

8.1.4 Effet indésirable inattendu

Effet indésirable dont la nature, la sévérité, la fréquence ou l'évolution ne concorde pas avec les informations de référence sur la sécurité mentionnés dans le protocole ou dans la brochure pour l'investigateur.

8.1.5 Fait nouveau

Toute nouvelle donnée pouvant conduire à une réévaluation du rapport des bénéfices et des risques de la recherche ou de la procédure objet de la recherche, à des modifications dans l'utilisation de cette procédure, dans la conduite de la recherche, ou des documents relatifs à la recherche, ou à suspendre ou interrompre ou modifier le protocole de la recherche ou des recherches similaires.

8.2 Responsabilités de l'investigateur

8.2.1 Modalités de détection et de recueil des événements indésirables

Tous les événements indésirables doivent être recherchés, rapportés et enregistrés, traités et évalués de la première visite (inclusion H0) jusqu'à la fin de l'étude et jusqu'à leur résolution.

Tous les événements indésirables seront notés sur les formulaires de recueil des événements indésirables du cahier d'observation. Chaque événement indésirable observé sera consigné individuellement. L'intensité des événements indésirables sera déterminée de la façon suivante :

- **légère (grade 1)** : pas d'interférence sur l'activité au quotidien du patient ;
- **modérée (grade 2)** : interférence modérée sur l'activité quotidienne du patient mais encore acceptable ;
- **sévère (grade 3)** : interférence importante sur l'activité quotidienne du patient et inacceptable ;
- **menace du pronostic vital (grade 4)** ;
- **décès (grade 5)**.

Tous les évènements indésirables doivent être gradés. Tous les événements indésirables **d'intensité sévère, menace du pronostic vital et décès (grade supérieur ou égal à 3 de l'échelle CTCAE) sont considérés comme GRAVE et doivent être notifiés sans délai au promoteur**, sauf s'ils sont décrits dans le tableau récapitulatif comme ne devant pas faire l'objet d'une notification sans délai auprès du promoteur.

Restriction des événements dans le eCRF :

Compte tenu du fort taux de mortalité de la population étudiée, certains événements ne seront pas collectés dans la section des événements indésirables du CRF, ni notifiés en EIG au promoteur avec le formulaire de notification d'EIG. Ces évènements sont rapportés, en fonction de leur niveau de sévérité, dans le tableau récapitulatif ci-dessous (identifiables avec la mention NON dans les colonnes « notification immédiate au promoteur » et « à collecter dans le CRF »).

Ces événements ne seront pas rapportés ni déclarés car attendus dans ce contexte d'EER en réanimation, et sont de risque égal dans les 2 bras, sans que ce risque soit impacté par la participation à la recherche.

8.2.2 Notification des EIG

L'investigateur évalue chaque événement indésirable au regard de sa gravité.

L'investigateur doit notifier au promoteur, sans délai et au plus tard dans les 24 heures à compter du jour où il en a connaissance, tous les événements indésirables graves survenus dans l'essai, à l'exception de ceux qui sont recensés dans le protocole ou dans la brochure pour l'investigateur comme ne nécessitant pas une notification sans délai (Tableau récapitulatif). Ces évènements sont rapportés dans le tableau récapitulatif ci-dessous (identifiable avec mention NON dans la colonne « notification immédiate au promoteur », et la mention OUI dans la colonne « à collecter dans le CRF » du tableau).

Cette notification initiale fait l'objet d'un rapport et doit être suivie par un ou des rapport(s) complémentaire(s) détaillé(s) dans les 8 jours suivant la première notification.
L'investigateur valide le formulaire de notification d'EIG, le date, et l'envoie par mail via l'eCRF à drci.eig-vigilance@chu-lyon.fr, dès lors qu'il a les 4 éléments minimum pour notifier un EIG :

- Un notificateur
- Un sujet
- Un produit expérimental (si applicable)
- Un événement indésirable

L'investigateur doit documenter au mieux l'événement (grâce aux copies des résultats de laboratoires ou des comptes rendus d'examens ou d'hospitalisation renseignant l'événement grave, y compris les résultats négatifs pertinents, **sans omettre de rendre ces documents anonymes** et d'inscrire le numéro et le code du patient), le diagnostic médic**al** et établir **un lien de causalité** entre l'événement indésirable grave et le(s) dispositif(s) médical(aux) et le geste de mise en oeuvre.

L'investigateur doit suivre le patient ayant présenté un EIG jusqu'à sa résolution, une stabilisation à un niveau jugé acceptable par l'investigateur ou le retour à l'état antérieur, même si le patient est sorti de l'essai et informer le promoteur en complétant l'eCRF, puis en envoyant par mail via eCRF à drci.eig-vigilance@chu-lyon.fr (remplir la partie FU [Follow Up] avec nom, date et signature sur l'eCRF).

Si l'eCRF est indisponible, l'investigateur peut scanner le formulaire de notification d'EIG signé et daté et l'envoyer par mail à drci.eig-vigilance@chu-lyon.fr ou par fax au 04 72 11 51 90. Il doit préciser dans l'objet du mail « NOM DE L'ETUDE-Critère de gravité*-N°centre-patient ».
*à remplir fonction du critère de gravité sélectionné dans le formulaire de notification d'EIG

8.2.3 Evaluation de la causalité

L'investigateur doit évaluer le lien de causalité des événements indésirables avec la recherche. Le lien de causalité est binaire (raisonnablement relié / non relié).

8.2.4 Période de notification des EIG sans délai au promoteur par l'investigateur et modalités de suivi des événements indésirables graves, restrictions des EI/EIG

L'investigateur doit notifier sans délai au promoteur les évènements indésirables graves :

- Dès l'inclusion du patient *(date de signature du 1er consentement)*;
- Jusqu'à la fin de sa participation dans l'étude.
- Sans limite de durée pour les évènements indésirables graves reliés à la recherche (par exemple : cancers, malformations congénitales survenant à long terme après exposition à la procédure…).

Les épisodes d'instabilité hémodynamique sont fréquents dans cette population de patients en insuffisance circulatoire aiguë traités par EER, et sont au cœur du dessin du protocole. Ils seront reportés dans l'eCRF et leur incidence surveillée par le comité scientifique. De même, les épisodes d'œdème aigu pulmonaire hydrostatique rentrent dans ce même cadre (complication fréquente et attendue de l'insuffisance rénale aiguë). De fait, ils seront rapportés dans la partie principale du CRF. Pour finir, l'aggravation de l'état hémodynamique, respiratoire ou d'autres fonctions d'organes (telles qu'objectivé par le score SOFA) sont fréquents dans cette population de réanimation, présentant d'emblée 2 défaillances à l'inclusion (hémodynamique et rénale) ; ces évènements sont rapportés dans l'eCRF, surveillés par les ARC de l'étude et le comité scientifique.

Tableau récapitulatif des EI/EIG

Intitulé de l'événement indésirable	*Notification immédiate au promoteur*	*Notification immédiate au promoteur à partir du grade*	*A collecter dans le CRF*	*A collecter dans le CRF à partir du grade*	*Commentaires*
**Décès**	**OUI**	**Tout événement listé ci-dessous ET fatal est gradé à 5 et déclaré immédiatement**	**OUI**	**Tout événement listé ci-dessous ET fatal est gradé à 5 et collecté**	
**Evt. cardiovasculaire non fatal**					
- Choc septique ou vasoplégique de novo	**NON**	5 (fatal)	**OUI**	≥3	*a*
- Choc cardiogénique de novo	**NON**	5 (fatal)	**OUI**	≥3	*a*
- Choc anaphylactique de novo	**NON**	5 (fatal)	**OUI**	≥3	*a*
- Choc hémorragique de novo (transfusion > 1/2 masse sanguine)	**NON**	5 (fatal)	**OUI**	≥3	*a*
- **Choc hypovolémique non hémorragique de novo (baisse de l'index cardiaque avec précharge dépendance et lactates > 2 mmol/L)**	**OUI**	**≥2**	**OUI**	**≥2**	
- Choc obstructif de novo	**NON**	5 (fatal)	**OUI**	≥3	*a*
- Aggravation de l'état cardiovasculaire ou hémodynamique	**NON**	5 (fatal)	**OUI**	≥3	
- Cœur pulmonaire aigu	**NON**	5 (fatal)	**NON**	5 (fatal)	
- **Arrêt cardiaque récupéré**	**OUI**	**≥2**	**OUI**	**≥2**	
- TPSV ou FA	**NON**	5 (fatal)	**NON**	5 (fatal)	
- Troubles du rythme ventriculaire	**NON**	5 (fatal)	**OUI**	≥3	
- Ischémie myocardique sans élévation du segment ST	**NON**	5 (fatal)	**OUI**	≥4	
- **Ischémie myocardique de novo, avec élévation du segment ST > 1 mm dans 2 dérivations contiguës**	**OUI**	**≥2**	**OUI**	**≥2**	
**Evt. neurologique non fatal**					
- Epilepsie authentifiée à l'EEG	**NON**	5 (fatal)	**NON**	5 (fatal)	
- Etat de mal épileptique authentifié à l'EEG	**NON**	5 (fatal)	**NON**	5 (fatal)	
- AVC hémorragique de novo authentifié à l'imagerie	**NON**	5 (fatal)	**OUI**	≥3	
- **AVC ischémique de novo authentifié à l'imagerie**	**OUI**	**≥2**	**OUI**	**≥2**	
- Coma (hors AVC ou épilepsie)	**NON**	5 (fatal)	**NON**	5 (fatal)	
- Neuromyopathie de réanimation	**NON**	5 (fatal)	**NON**	5 (fatal)	
**Evt. respiratoire non fatal**					
- **Aggravation de l'état respiratoire avec ventilation invasive**	**OUI**	**≥2**	**OUI**	**≥2**	

- Aggravation de l'état respiratoire sans ventilation invasive	NON	5 (fatal)	OUI	≥4	
- **Œdème aigu pulmonaire hydrostatique**	OUI	**≥2**	OUI	**≥2**	
- Atélectasie	NON	5 (fatal)	NON	5 (fatal)	
- Pneumothorax ou pneumomédiastin	NON	5 (fatal)	NON	5 (fatal)	
- Embolie pulmonaire	NON	5 (fatal)	NON	5 (fatal)	
**Evt. hépatique non fatal**					
- Cytolyse hépatique/élévation des transaminases	NON	5 (fatal)	NON	5 (fatal)	
- Cholestase/élévation des PAL, GGT ou bilirubine	NON	5 (fatal)	NON	5 (fatal)	
- Insuffisance hépatocellulaire aiguë, définie par un facteur V < 50%	NON	5 (fatal)	OUI	≥3	
**Evt. abdominal non fatal**					
- Hydrocholécyste	NON	5 (fatal)	NON	5 (fatal)	
- Cholécystite	NON	5 (fatal)	NON	5 (fatal)	
- Œsophagite	NON	5 (fatal)	NON	5 (fatal)	
- Ulcère gastro-duodénale	NON	5 (fatal)	OUI	≥3	
- **Ischémie mésentérique de novo authentifiée à l'imagerie**	OUI	**≥2**	OUI	**≥2**	
- Colite ischémique	NON	5 (fatal)	OUI	≥3	
- Péritonite	NON	5 (fatal)	NON	5 (fatal)	
**Evt. rénal non fatal**					
- Insuffisance rénale aiguë KDIGO 3	NON	5 (fatal)	NON	5 (fatal)	*b*
- Insuffisance rénale aiguë KDIGO 1 ou 2	NON	5 (fatal)	NON	5 (fatal)	
**Evt. lié à l'épuration extra-rénal non fatal**					
- Saignement sur site de cathéter de dialyse	NON	5 (fatal)	NON	5 (fatal)	
- Thrombose veineuse sur cathéter de dialyse	NON	5 (fatal)	NON	5 (fatal)	
- Thrombose de circuit d'EER	NON	5 (fatal)	NON	5 (fatal)	
**Evt. métabolique non fatal**					
- Hypokaliémie < 3.5 mmol/L	NON	5 (fatal)	NON	5 (fatal)	
- Hyperkaliémie > 5 mmol/L	NON	5 (fatal)	NON	5 (fatal)	
- Hyponatrémie < 135 mmol/L	NON	5 (fatal)	NON	5 (fatal)	
- Hypernatrémie > 145 mmol/L	NON	5 (fatal)	NON	5 (fatal)	
- Dyscalcémie	NON	5 (fatal)	NON	5 (fatal)	
- Dysphosphorémie ou dysmagnésémie	NON	5 (fatal)	NON	5 (fatal)	
- Alcalose métabolique sévère : pH > 7.60 et HCO_3^- > 40 mmol/L et PaCO2 ≤ 45 mmHg	NON	5 (fatal)	OUI	≥3	
- Alcalose métabolique non sévère	NON	5 (fatal)	NON	5 (fatal)	

- Alcalose respiratoire ou mixte (ne répondant pas à la définition de l'acidose métabolique sévère)	NON	5 (fatal)	NON	5 (fatal)	
- Acidose lactique (pH < 7.38 et lactates > 2 mmol/L)	NON	5 (fatal)	NON	5 (fatal)	
- Autres acidoses (pH < 7.38 et lactates ≤ 2 mmol/L)	NON	5 (fatal)	NON	5 (fatal)	
- Dysglycémie	NON	5 (fatal)	NON	5 (fatal)	
**Evt. hématologique non fatal**					
- Anémie < 120 g/L	NON	5 (fatal)	NON	5 (fatal)	
- Thrombopénie < 150 G/L	NON	5 (fatal)	NON	5 (fatal)	
- Leucopénie < 4000 G/L	NON	5 (fatal)	NON	5 (fatal)	
**Evt. infectieux non fatal**					
- Infection liée au cathéter	NON	5 (fatal)	OUI	≥4	
- PAVM documentée	NON	5 (fatal)	OUI	≥4	
- PAVM non documentée	NON	5 (fatal)	NON	5 (fatal)	
- Bactériémie documentée sur 2 sites distincts	NON	5 (fatal)	OUI	≥4	
- Autres bactériémies	NON	5 (fatal)	NON	5 (fatal)	
- Autres infections nosocomiales documentées	NON	5 (fatal)	OUI	≥4	
**Evt. autre non fatal**					
- Thrombophlébite de veines profondes, hors EP	NON	5 (fatal)	NON	5 (fatal)	
- **Ischémie artérielle aiguë de membre de novo authentifiée à l'imagerie**	OUI	**≥2**	OUI	**≥2**	
- **Autre ischémie aiguë d'organe de novo, authentifiée à l'imagerie**	OUI	**≥2**	OUI	**≥2**	
- **Tout événement indésirable non listé ci-dessus de grade 3 ou plus**	OUI	**≥3**	OUI	**≥3**	
- Tout événement indésirable non listé ci-dessus de grade 2 ou moins	NON	-	NON	-	

a. Uniquement si résolution de l'épisode initial ayant mené à l'inclusion
b. Critère d'inclusion

8.3 Responsabilités du promoteur

8.3.1 Déclaration aux autorités compétentes

Le Promoteur déclarera à l'ANSM:

- en cas de mise en jeu du pronostic vital ou de décès du sujet : toutes les suspicions d'effets indésirables graves inattendus sans délai à compter du jour où le promoteur en a connaissance et les informations complémentaires pertinentes devant être soumises sous forme de rapport de suivi dans un délai de 8 jours dès réception du suivi.
- pour tous les autres effets indésirables graves inattendus : au plus tard dans un délai de 15 jours à compter du jour où le promoteur en a connaissance, et les informations complémentaires pertinentes devant être soumises sous forme d'un rapport de suivi dans un nouveau délai de 8 jours dès réception du suivi.
- à l'ANSM, au CPP, les faits nouveaux sans délai à compter du jour où le promoteur en a connaissance, 8 jours dès connaissance d'une nouvelle information de sécurité pertinente pour les suivis (selon l'article R1123-59 du CSP).

Il établira un également rapport annuel de sécurité (RAS) qui sera transmis à l'ANSM et au CPP sous un délai de 60 jours après la date anniversaire de l'étude.

8.3.2 Description des effets indésirables potentiellement liés à la recherche (référence de sécurité pour l'évaluation du caractère attendu/inattendu par le promoteur)

Événements indésirables graves :

1. Décès
2. Choc hypovolémique non hémorragique de novo (baisse de l'index cardiaque avec précharge dépendance et lactates > 2 mmol/L)
3. Arrêt cardiaque récupéré
4. Ischémie myocardique de novo, avec élévation du segment ST > 1 mm dans 2 dérivations contiguës
5. AVC ischémique de novo authentifié à l'imagerie
6. Aggravation de l'état respiratoire avec ventilation invasive
7. Œdème aigu pulmonaire hydrostatique
8. Ischémie mésentérique de novo authentifiée à l'imagerie
9. Ischémie artérielle aiguë de membre de novo authentifiée à l'imagerie
10. Autre ischémie aiguë d'organe de novo, authentifiée à l'imagerie

L'ensemble des EIG décrits listés ci-dessus, incluant les décès, sont des événements fréquents dans ce contexte médical de réanimation, et potentiellement associés aux interventions de l'étude (que ce soit le groupe interventionnel ou contrôle), en particulier en dehors de toute protocolisation de la surveillance de la déplétion hydrosodée (monitorage hémodynamique). Il est donc attendu que leur incidence soit rendue rare par l'objet même évalué par le protocole, c'est-à-dire la sécurisation de l'UF_{NET} par le monitorage hémodynamique protocolisée, permettant de limiter l'incidence d'une baisse du débit cardiaque avec induction d'une ischémie d'organe (pour le groupe interventionnel), ou a contrario la génération d'une surcharge hydrosodée ayant pour conséquence une insuffisance respiratoire aiguë (pour le groupe contrôle). C'est aussi dans ce sens qu'a été choisi l'objectif principal, soit le maintien d'une balance hydrosodée **neutre (et non négative)** dans cette population de patients graves.

Les décès sont potentiellement attendus comme complications ultimes de ces EIG, car ces derniers viennent aggraver le plus souvent l'état physiologique déjà altéré de patients en état de défaillance multiviscérale. Comme expliqué ci-dessous, alors que le taux de mortalité attendu dans cette population est exceptionnellement élevée (probablement supérieur à 40%), nous nous attendons toutefois à une incidence rare des décès directement et isolément associé à l'intervention testée, pour les mêmes raisons qu'expliquées ci-dessus (monitorage du débit cardiaque, prise en charge de l'insuffisance respiratoire aiguë associée à la

surcharge hydrosodée).

8.4 Comité de surveillance indépendant

Il n'est pas prévu de comité de surveillance indépendant pour cette étude. Tout d'abord, la nature de l'intervention correspond à une protocolisation de pratique d'EER (soustraction hydrosodée par UF_{NET}) déjà largement utilisée depuis plus de 40 ans dans les unités de réanimation. De plus, la population étudiée est intrinsèquement exposée à un risque très élevé de décès (non lié à l'étude) du fait du syndrome de défaillance multiviscérale. Pour finir, le monitorage hémodynamique avancée est déjà très largement utilisée dans les unités participant à l'étude, ramenant l'intervention à une protocolisation des pratiques de soustraction hydrosodée et de monitorage.

Néanmoins, il est décidé d'évaluer l'incidence des décès survenus jusqu'à H72 dans les 2 groupes après l'inclusion de 20 patients. La mise en évidence d'un taux de mortalité à H72 anormalement élevé dans cette population et à ce temps d'analyse (éventuellement complété d'une analyse statistique évaluant la différence significative du nombre de décès dans l'un des 2 bras, comparativement à l'autre, en utilisant un test de Fisher), entrainera alors une réévaluation par le comité scientifique et la pharmacovigilance du promoteur de la poursuite des inclusions. Cette analyse ne portant pas sur le critère de jugement principal, elle n'impactera pas la puissance de l'étude.

9 ASPECTS STATISTIQUES

9.1 Nombre de sujets nécessaires

Dans cet essai, les patients sont randomisés en 2 groupes :
- groupe 1 : neutralisation de la balance entrées/sorties (intervention)
- groupe 2 : groupe contrôle

Le groupe interventionnel correspond à la neutralisation de la balance entrées/sorties par l'application d'une UF_{NET} ≥ 100 ml/h, afin d'être équivalente aux entrées reçues par le patient, et guidée par un monitorage hémodynamique intensif et fonctionnel. Le groupe contrôle reflète les pratiques actuelles de contrôle de la balance entrées/sorties chez le patient de réanimation sous EER, par l'application d'une UF_{NET} comprise entre 0 et 25 ml/h [10].

Le critère de jugement principal est la balance entrées/sorties mesurée à 72h de l'inclusion dans les 2 groupes.

Cet essai est une étude interventionnelle de 1ère catégorie pilote, d'efficacité, de supériorité, randomisée contre traitement de référence, en 2 groupes parallèles, ouverte, et multicentrique (inter-régionale).

Nous faisons l'hypothèse que la balance entrées/sorties sera de +4000 ml ± 4000 à 72h de l'inclusion dans le groupe contrôle, et que la procédure de neutralisation de la balance entrées/sorties (intervention) permettra d'obtenir une balance entrées/sorties égale à 0 ml ± 4000 à 72h de l'inclusion, soit équivalent à une diminution de 4000 ml. Cette valeur correspond à la balance entrées/sorties médiane mesurée à J3 dans l'étude IDEAL ICU (3711 ml dans le bras précoce et 3917 ml dans le bras tardif), arrondie à 4000 ml de façon conservatrice [10].

Dans cette situation, le calcul fondé sur la formule de comparaison de 2 moyennes issues de 2 échantillons indépendants, avec un risque α de 5% et une puissance $1-\beta$ de 80%, il faut inclure au moins 16 patients dans chaque groupe, soient 32 patients au total [45].

Afin de tenir compte d'une proportion de patients décédés à 72h de l'inclusion (évaluée à 25%) ainsi que de la distribution non paramétrique du critère de jugement principal (justifiant une augmentation de 25% de l'effectif), on décide de recruter 29 patients par groupe, soit 58 patients analysables au total [10]. Les patients non décédés avant H72 pour lesquels le critère de jugement principal est manquant (soit pour retrait de consentement, soit pour survenue d'une contre-indication définitive ou prolongée) ne seront pas inclus dans l'analyse finale.

Une fois le nombre de sujet nécessaires atteints (soit 21 patients dans chaque bras en tenant compte de l'augmentation justifiée par la distribution non paramétrique du critère de jugement principal) avec critère de jugement principal analysable (balance entrées/sorties à H72), les inclusions pourront être stoppées, afin de ne pas inclure inutilement de nouveaux patients.

9.2 Population d'analyse

L'analyse se fera en intention de traiter. La population en intention de traiter est définie comme l'ensemble des patients vivants à H72 inclus dans l'étude selon le bras alloué lors de la randomisation, quels que soient les critères d'éligibilité et la fréquence des violations du protocole, et évaluables pour le critère de jugement principal (à H72). Cette analyse n'inclut donc pas les patients pour lesquelles le critère de jugement principal n'a pu être évalué à H72, incluant les sorties d'étude précoces avant H72 et les patients décédés avant H72. L'ensemble de l'analyse des critères de jugement principal et secondaires seront réalisés sur cette population.

Nous réaliserons par ailleurs une analyse en intention de traiter modifiée. Celle-ci portera sur la population suivante : les patients vivants à H72, et les patients décédés avant H72, inclus dans l'étude selon le bras alloué lors de la randomisation, quels que soient les critères d'éligibilité et la fréquence des violations du protocole. Le critère de jugement sera la balance entrées/sorties à H72, ou au moment de décès, le cas échéant. La balance entrées/sorties sera rapportée toutes les 4 heures pour chaque patient (et non la somme cumulative des 72h). Nous réaliserons à partir de ces données une régression linéaire multivariée à effets mixtes avec la balance E/S par 4 heures en variable dépendante, le groupe de randomisation et le statut vital en variables indépendantes à effet fixe, et le temps écoulé depuis l'inclusion en variable avec effet aléatoire. Une analyse similaire portera sur le nombre total d'épisodes d'instabilité hémodynamiques, et ceux catégorisés comme étant associés ou non à une précharge dépendance, et survenus entre H0 et H72 (ou jusqu'au décès si plus précoce) dans cette population en ITT modifiée.

9.3 Méthodes statistiques

Les données quantitatives seront décrites pour l'ensemble de la population, à l'aide des statistiques descriptives suivantes : l'effectif, le nombre de valeurs manquantes, la médiane, le premier et le troisième quartile, ainsi que le minimum et le maximum. Les variables quantitatives pourront être catégorisées en utilisant leur médiane ou une valeur seuil indiscutable retrouvée dans la littérature médicale.

Les variables qualitatives seront résumées pour l'ensemble de la population, à l'aide des statistiques descriptives suivantes : les fréquences et les pourcentages pour chaque niveau de la variable et les valeurs manquantes (les valeurs manquantes seront dénombrées mais ne seront pas inclues dans le dénominateur du calcul des fréquences). Ces statistiques seront considérées comme les statistiques usuelles pour l'analyse de variables qualitatives.

L'analyse sera réalisée en intention de traiter sur toute la population de patients inclus. Elle portera sur le critère de jugement principal et les critères de jugements secondaires, et permettra leur comparaison entre les 2 groupes. Il sera aussi présenté les résultats du monitorage hémodynamique protocolisé du bras interventionnel, dont les données seront comparées à celles du bras contrôle.

Les variables quantitatives sans répétition seront comparées à l'aide du test de Wilcoxon-Mann-Whitney. Les variables qualitatives non dépendantes du temps seront comparées à l'aide du test de Fisher. En cas de répétition d'une mesure chez un même patient (par exemple, mesure pluriquotidienne de l'index cardiaque, score SOFA, analyse en ITT modifiée), nous analyserons la différence de moyenne entre les 2 bras à l'aide d'un modèle linéaire mixte dont l'effet fixe sera le groupe, et l'effet aléatoire comprendra le patient et l'ordre temporel de mesure [46].

Une valeur de $p < 0.05$ sera considérée comme étant statistiquement significative.

Toutes les analyses statistiques seront réalisées à l'aide du logiciel R (The R Project for Statistical Computing, R Core Team) [47].

9.4 Analyses intermédiaires

Il n'est pas prévu de réaliser d'analyse intermédiaire sur le critère de jugement principal pour cette recherche. Il sera par contre réalisé une analyse intermédiaire après 20 inclusions sur le taux de mortalité à H72 dans les 2 bras (10+10), afin de s'assurer de la sécurité de l'intervention et du groupe contrôle.

9.5 Méthode de prise en compte des données manquantes

La fréquence des données manquantes et leurs causes seront comparées par groupe de traitement. Les données manquantes et leur fréquence seront rapportées dans la publication finale. Les patients vivants à H72 mais dont le critère de jugement principal est manquant à ce moment, seront remplacés.

9.6 Gestion des modifications apportées au plan d'analyse

Un plan d'analyse statistique détaillé sera rédigé avant le gel de la base des données. Il tiendra compte de toute modification du protocole ou de tout événement inattendu survenu au cours de l'étude et ayant un impact sur les analyses présentées ci-dessus. Les analyses planifiées pourront être complétées en cohérence avec les objectifs de l'étude.

Toute modification apportée par la suite au plan d'analyse statistique devra être justifiée et donnera lieu à une nouvelle version du document. Ces déviations au plan d'analyse seront reportées dans le rapport final de l'étude. L'ensemble des documents sera conservé dans le dossier de l'étude.

9.7 Responsable des analyses

Dr Laurent BITKER
Service de Médecine Intensive – Réanimation
Hôpital de la Croix Rousse/Groupement Hospitalier Nord
Hospices Civils de Lyon
103, Grande rue de la Croix Rousse, 69004 Lyon
Tél : 04.26.10.94.93 ; Fax : 04.72.07.17.74
Email : laurent.bitker@chu-lyon.fr

Mr Pierre PRADAT
Centre de Recherche Clinique
Hôpital de la Croix Rousse/Groupement Hospitalier Nord
Hospices Civils de Lyon
103, Grande rue de la Croix Rousse, 69004 Lyon
Tél : 04.26.73.27.15 ; Fax : 04.26.73.27.34
Email : pierre.pradat@univ-lyon1.fr

10 SURVEILLANCE DE LA RECHERCHE

Le conseil scientifique de l'étude sera composé des investigateurs du centre coordinateur (Dr Laurent Bitker et Pr Jean-Christophe Richard), du méthodologiste (Mr Pierre Pradat) et de la coordinatrice d'étude clinique (Mme Loredana Baboi). Ils s'assureront du bon rythme des inclusions, de la collection des données et de leur qualité, et de la déclaration des EI et EIG.

11 DROITS D'ACCES AUX DONNEES ET DOCUMENTS SOURCES

11.1 Accès aux données

Conformément aux BPC :

- Le promoteur est chargé d'obtenir l'accord de l'ensemble des parties impliquées dans la recherche afin de garantir l'accès direct à tous les lieux de déroulement de la recherche, aux données sources, aux documents sources et aux rapports dans un but de contrôle de qualité et d'audit par le promoteur ;
- Les investigateurs mettront à disposition des personnes chargées du suivi, du contrôle de qualité ou de l'audit de la recherche impliquant la personne humaine, les documents et données individuelles strictement nécessaires à ce contrôle, conformément aux dispositions législatives et réglementaires en vigueur (articles L.1121-3 et R.5121-13 du code de la santé publique).

11.2 Documents sources

Les documents sources sont définis comme tout document ou objet original permettant de prouver l'existence ou l'exactitude d'une donnée ou d'un fait enregistrés au cours de l'étude clinique. Ils seront conservés pendant 25 ans par l'investigateur ou par l'hôpital s'il s'agit d'un dossier médical hospitalier.

Les documents sources suivants seront utilisés :
- Dossier médical
- Pancarte de réanimation
- Cahier d'observation papier au lit du malade (comprenant les données du protocole hémodynamique recueillies toutes les 4h par les équipes paramédicale et médicale en charge du patient) – Cahier IDE
- Original de résultats d'examens biologiques

11.3 Confidentialité des données

Conformément aux dispositions concernant la confidentialité des données auxquelles ont accès les personnes chargées du contrôle de qualité d'une recherche impliquant le personne humaine (article L.1121-3 du code de la santé publique), conformément aux dispositions relatives à la confidentialité des informations concernant notamment les essais, les personnes qui s'y prêtent et les résultats obtenus (article R. 5121-13 du code de la santé publique), les personnes ayant un accès direct aux données prendront toutes les précautions nécessaires en vue d'assurer la confidentialité des informations relatives aux essais, aux personnes qui s'y prêtent et notamment en ce qui concerne leur identité ainsi qu'aux résultats obtenus.

Ces personnes, au même titre que les investigateurs eux-mêmes, sont soumises au secret professionnel (selon les conditions définies par les articles 226-13 et 226-14 du code pénal).

Pendant la recherche impliquant la personne humaine ou à son issue, les données recueillies sur les personnes qui s'y prêtent et transmises au promoteur par les investigateurs (ou tous autres intervenants spécialisés) seront codées. Elles ne doivent en aucun cas faire apparaître en clair les noms des personnes concernées ni leur adresse.

Le code d'un patient correspondra à :
- La première lettre de son NOM et de son PRENOM
- Un code à 1 lettre et 2 chiffres, associant la lettre du centre participant (de A à D), et le numéro d'inclusion dans l'ordre chronologique d'inclusion (par exemple, le premier patient du centre A sera codé A01).

Le promoteur s'assurera que chaque personne qui se prête à la recherche a donné son accord par écrit pour l'accès aux données individuelles la concernant et strictement nécessaires au contrôle de qualité de la recherche.

12 CONTROLE ET ASSURANCE DE LA QUALITE

Un Attaché de Recherche Clinique (ARC) mandaté par le promoteur s'assurera de la bonne réalisation de l'étude, du recueil des données générées par écrit, de leur documentation, enregistrement et rapport, en accord avec les Procédures Opératoires Standards mises en application au sein de la DRCI des Hospices Civils de Lyon et conformément aux Bonnes Pratiques Cliniques ainsi qu'aux dispositions législatives et réglementaires en vigueur.

L'investigateur et les membres de son équipe acceptent de se rendre disponibles lors des visites de Contrôle de Qualité effectuées à intervalles réguliers par l'Attaché de Recherche Clinique. Lors de ces visites, les éléments suivant pourront être revus en fonction du niveau de monitoring adapté à l'étude et déterminé conformément aux POS du Promoteur, selon le plan de monitoring :

- Consentement éclairé
- Respect du protocole de l'étude et des procédures qui y sont définies
- Qualité des données recueillies dans le cahier d'observation : exactitude, données manquantes, cohérence des données avec les documents sources
- Gestion des procédures expérimentales
- Déclaration des événements indésirables graves.

Toute visite fera l'objet d'un rapport de monitoring par compte-rendu écrit adressé à l'investigateur du centre visité et à la structure de coordination de la recherche.

D'autre part, les investigateurs s'engagent à accepter les audits d'assurance qualité effectués par des personnes mandatées par le promoteur ainsi que les inspections effectuées par les Autorités Compétentes. Toutes les données, tous les documents et rapports peuvent faire l'objet d'audits et d'inspections réglementaires sans que puisse être opposé le secret médical.

13 CONSIDERATIONS ETHIQUES

13.1 Autorités compétentes

Le protocole, la notice d'information et le formulaire de consentement de l'étude seront soumis pour avis au Comité de Protection des Personnes XX (Préciser le numéro du CPP désigné après avis).

La notification de l'avis favorable du CPP sera transmise au promoteur de l'étude et à l'ANSM. Une demande d'autorisation d'étude sera également adressée par le promoteur à l'ANSM. Le promoteur s'engage à ce que le démarrage de l'étude ne se fasse qu'après obtention de l'avis favorable du CPP et de l'autorisation d'étude de l'ANSM.

13.2 Modifications substantielles

En cas de modification substantielle apportée au protocole par l'investigateur, elle sera approuvée par le promoteur. Ce dernier devra obtenir préalablement à sa mise en œuvre un avis favorable du CPP et une autorisation de l'ANSM dans le cadre de leurs compétences respectives. Un nouveau consentement des personnes participant à la recherche sera recueilli si nécessaire.

13.3 Information du patient et formulaire de consentement écrit

Du fait du contexte spécifique de la réanimation, l'évaluation de l'éligibilité des patients pourra se faire 24 heures sur 24, et leur inclusion de même, le cas échéant.

Le patient, ou son proche le cas échéant, sera informé de façon complète et loyale, en des termes compréhensibles, des objectifs et des contraintes de l'étude, des risques éventuels encourus, des mesures de surveillance et de sécurité nécessaires, de son droit de refuser de participer à l'étude ou de la possibilité de se rétracter à tout moment.

Toutes ces informations figurent sur un formulaire d'information et de consentement remis au patient, ou son représentant légal. Le consentement libre, éclairé et écrit du patient, ou de son représentant légal, sera recueilli par l'investigateur, ou un médecin qui le représente avant l'inclusion définitive dans l'étude. Une copie du formulaire d'information et de consentement signé par les deux parties sera remise au patient, ou à son représentant légal, l'investigateur en conservera l'original. Le recueil du consentement devra apparaître dans le dossier médical.

Du fait du contexte dans lequel se déroulera l'étude (étude en réanimation, introduction de technique de monitorage et ou d'EER en urgence) et de la proportion élevé de patients qui sera dans l'incapacité d'exprimer leur volonté initialement (anesthésie général, coma ou confusion, inconfort ou dyspnée), nous proposerons au

CPP de réaliser une **procédure d'inclusion par l'obtention du consentement du proche** du patient, qui recevra les mêmes informations que si le patient en avait eu les moyens. Une fois le patient dans un état clinique jugé compatible pour recevoir ces informations, le processus de consentement sera répété auprès de lui selon les mêmes modalités que décrites plus haut, en particulier de leurs droits de refuser de continuer à participer à l'étude ou de la possibilité de se rétracter à tout moment. Du fait que le patient ne sera pas en capacité d'être informé au moment son éligibilité, cette information et le recueil du consentement pour continuer à participer se fera donc a posteriori.

Du fait du contexte dans lequel se déroulera l'étude (étude en réanimation, introduction de technique de monitorage et ou d'EER en urgence) et de la proportion élevée de patients qui sera dans l'incapacité d'exprimer leur volonté initialement (anesthésie général, coma ou confusion, inconfort ou dyspnée), nous proposerons au CPP de réaliser une **procédure d'inclusion en urgence**. En effet, l'EER peut être débutée en urgence dans ce contexte (par exemple la nuit), et le patient pourrait être incapable d'exprimer sa volonté (anesthésie général, coma ou confusion, inconfort ou dyspnée) et/ou ses proches dans l'impossibilité d'être contactés ou de se déplacer. De plus, du fait de l'urgence de l'indication de la dialyse, il ne semble toujours possible de laisser un délai de réflexion suffisamment long avant d'initier l'intervention.

L'investigateur remplit alors un formulaire confirmant l'absence de présence des proches du patient sur le site. L'information des proches et le recueil de leur consentement devra être réalisée dès que possible. En cas de refus de consentement par ses proches, le patient inclus selon la procédure d'urgence sera exclu de l'étude. Toutefois, les patients inclus selon la procédure d'urgence, sans proche identifiable, et qui décèderaient sans avoir repris un état de conscience normale, resteront inclus dans l'étude. Une fois que le patient sera en état de donner son consentement, une notice d'information et un formulaire de confirmation de participation lui seront fournis (comme suite à l'inclusion avec accord du proche). Le formulaire de confirmation de participation sera signé par le patient et une copie lui sera fournie. L'investigateur en conservera l'original.

13.4 *Déclaration de conformité*

Le promoteur et l'investigateur s'engagent à ce que cette recherche soit conduite :
- conformément au protocole,
- conformément aux bonnes pratiques cliniques françaises et internationales actuellement en vigueur,
- conformément aux dispositions législatives et réglementaires actuellement en vigueur en France et au niveau international.

13.5 *Période d'exclusion*

Les patients inclus dans cette étude ne pourront pas participer à une recherche interventionnelle dont le critère de jugement principal serait la balance hydrosodée ou dont l'intervention porte sur l'hémodynamique, l'EER, ou la modification de la balance entrées/sorties. Il n'est pas prévu de période d'exclusion pour cette recherche.

13.6 *Indemnisation des sujets et inscription au fichier national des personnes se prêtant à une recherche interventionnelle sur la personne humaine du 1°*

Il n'est pas prévu d'indemniser les volontaires qui accepteront de participer à l'étude. L'objet de la recherche étant en rapport avec l'état pathologique des sujets, l'inscription au fichier national des Volontaires se prêtant à une Recherche Biomédicale (VRB) n'est pas requise.

14 GESTION ET CONSERVATION DES DONNEES

14.1 Cahier d'observation

Les données de l'étude seront recueillies dans un cahier d'observation électronique (eCRF). Cet eCRF, spécifique à l'étude, sera développé par un datamanager des Hospices Civils de Lyon sur le logiciel Ennov EDC. Ce logiciel respecte les recommandations de la *Food and Drug Administration* (FDA) sur les systèmes informatisés pour la gestion des essais cliniques (*Guidance for Computerized Systems Used in Clinical Trials*) ainsi que les recommandations FDA sur la signature électronique (21CFR part 11). Chaque personnel participant à la recherche se verra remettre un identifiant unique avec mot de passe à complexité élevée (choisi par l'individu) afin de se connecter au eCRF (et ainsi permettre la traçabilité des connections, entrées, et modification).

Les données hémodynamiques d'évaluation par le protocole présenté dans la figure 1 seront collectées sur un cahier d'observation/document source papier placé au lit du patient (cahier IDE), et complété toutes les 4h par le personnel médical ou paramédical. Ces données seront ensuite saisies dans l'eCRF.

Le eCRF n'inclura que les données nécessaires à la réalisation du protocole et à la publication scientifique. Les autres données des patients nécessaires à leur suivi en dehors de cette étude seront collectées dans le dossier médical du patient.

Les données de l'étude seront informatisées de façon codée, conformément à la loi informatique et liberté. Les sujets/patients de l'étude seront identifiés par leur numéro unique d'inclusion dans l'étude et par la première lettre de leur nom et de leur prénom. Une liste d'identification des participants à la recherche sera conservée dans le classeur investigateur.

Les données doivent être complétées, au fur et à mesure de leur collecte, par les personnes autorisées (investigateur et personnes apparaissant sur la délégation de tâches) et disposant de leurs propres identifiants, conformément à la loi informatique et liberté. Le remplissage du cahier d'observation via internet permet au centre de coordination et au promoteur de l'étude de visualiser rapidement et à distance les données. Lors de la saisie, les données sont immédiatement vérifiées grâce à des contrôles de cohérence. La personne en charge du remplissage doit valider et justifier toute modification de valeur dans le CRF. Les entrées et modifications font l'objet d'un *audit trail*.

L'investigateur est responsable de l'exactitude, de la qualité et de la pertinence de toutes les données saisies. A ce titre, chaque page du CRF patient doit être datée et signée électroniquement par l'investigateur, signifiant ainsi son accord et sa responsabilité vis-à-vis des données collectées.

14.2 *Gestion des données*

La saisie simple des données sera réalisée dans l'eCRF (Ennov EDC) par un opératoire du centre investigateur (TEC) et relue par l'ARC du centre coordinateur. Les données sont validées conformément au plan de data management défini conjointement entre l'investigateur coordinateur, le méthodologiste, le data manager et le statisticien. Le processus de gel/dégel des données est réalisé conformément à la procédure définie conjointement entre l'investigateur coordinateur, le méthodologiste et le data manager (gel des données brutes sur la plateforme Ennov CliniCal puis exportation au format EXCEL). L'ensemble des données est sauvegardé en temps réel sur les serveurs hébergeant l'eCRF, et archivé mensuellement sur disque dur protégé par mot de passe et enfermé dans une armoire fermée à clef.

Le données non-pseudonymisée (dossier médical) et le registre de concordance entre l'identité du patient et son identifiant d'étude sera conservée dans les centres participants, et ne sera pas communiqué au centre coordinateur. Le registre de concordance sera protégé dans chaque site dans un bureau fermé à clef sur un ordinateur protégé par mot de passe.

14.3 *CNIL*

Cette étude entre dans le cadre de la « Méthodologie de Référence » (MR-001) en application des dispositions de l'article 54 alinéa 5 de la loi n°78-17 du 6 janvier 1978 modifiée relative à l'informatique, aux fichiers et aux libertés. Ce changement a été homologué par décision du 5 janvier 2006 et modifiée le 21 juillet 2016. Les Hospices Civils de Lyon, promoteur de l'étude, ont signé un engagement de conformité à cette « Méthodologie de Référence ».

14.4 *Archivage*

Les documents suivants seront archivés par le nom de l'étude sous la responsabilité de l'investigateur coordonnateur et des investigateurs associés au niveau de chaque centre pendant 25 ans :

- Protocole et annexes, amendements éventuels,
- Formulaires d'information et consentements originaux signés
- Données individuelles (copies authentifiées de données brutes)
- Documents de suivi et courriers relatifs à la recherche

Le Promoteur est également responsable d'organiser la conservation des analyses statistiques et du rapport final de l'étude pendant la durée réglementaire d'archivage. Aucun déplacement ou destruction ne pourra être effectué sans l'accord du promoteur. Au terme des 25 ans, le promoteur sera consulté pour destruction. Toutes les données, tous les documents et rapports pourront faire l'objet d'audit ou d'inspection.

15 *FINANCEMENT ET ASSURANCE*

15.1 *Budget de l'étude*

Les frais liés à cette recherche sont les suivants :

- Temps médical (investigateurs principaux des centres participants)
- Temps Porteur du projet
- Temps Coordination du Projet
- Temps TEC
- Temps ARC du projet
- Temps Méthodologiste
- Data management et eCRF
- Statistiques
- Vigilance
- Promotion et assurance
- Frais de publications

- Frais de papeterie
- Frais de déplacement
- Archivage

Leurs descriptions et les montants associés sont détaillés dans la grille budgétaire. Le budget total de l'étude s'élève à **128 330 €**.

Un financement PHRC inter-régional Auvergne-Rhône-Alpes 2019 a été obtenu pour couvrir les coûts de ce projet.

15.2 Assurance

Le promoteur a souscrit pour toute la durée de l'étude une assurance garantissant sa propre responsabilité civile ainsi que celle de tout médecin impliqué dans la réalisation de l'étude. Il assurera également l'indemnisation intégrale des conséquences dommageables à la recherche pour la personne qui s'y prête et ses ayants droit, sauf preuve à sa charge que le dommage n'est pas imputable à sa faute ou à celle de tout intervenant, sans que puisse être opposé le fait d'un tiers ou le retrait volontaire de la personne qui avait initialement consenti à se prêter à la recherche.

Le contrat d'assurance a été souscrit avant le démarrage de l'étude auprès de la Société Hospitalière d'Assurance Mutuelle, 18 rue Edouard Rochet, 69008 Lyon, sous le numéro 159.077.

16 REGLES RELATIVES A LA PUBLICATION

Les communications et rapports scientifiques correspondant à cette étude seront réalisés sous la responsabilité de l'investigateur principal de l'étude avec l'accord des investigateurs associés. Les coauteurs du rapport et des publications seront les investigateurs principaux de chaque centre participant, au prorata de leur contribution à l'étude, ainsi que le biostatisticien associé. Les centres participants pourront ajouter un co-auteur supplémentaire par 7 inclusions réalisées par leur centre.

Les règles de publications suivront les recommandations internationales (N Engl J Med, 1997; 336 :309-315). L'étude sera enregistrée sur un registre des essais cliniques en libre accès (clinicaltrials.gov) avant l'inclusion du 1er patient. Le protocole de l'étude sera publié dans un journal dédié.

17 REFERENCES BIBLIOGRAPHIQUES

1. Claure-Del Granado R, Mehta RL: **Fluid overload in the ICU: evaluation and management**. *BMC Nephrol* 2016, **17**(1):109.
2. Sakr Y, Rubatto Birri PN, Kotfis K, Nanchal R, Shah B, Kluge S, Schroeder ME, Marshall JC, Vincent JL, Intensive Care Over Nations I: **Higher Fluid Balance Increases the Risk of Death From Sepsis: Results From a Large International Audit**. *Critical care medicine* 2016.
3. Vaara ST, Korhonen AM, Kaukonen KM, Nisula S, Inkinen O, Hoppu S, Laurila JJ, Mildh L, Reinikainen M, Lund V *et al*: **Fluid overload is associated with an increased risk for 90-day mortality in critically ill patients with renal replacement therapy: data from the prospective FINNAKI study**. *Critical care (London, England)* 2012, **16**(5):R197.
4. National Heart Lung Blood Institute, Acute Respiratory Distress Syndrome Clinical Trials Network, Wiedemann HP, Wheeler AP, Bernard GR, Thompson BT, Hayden D, deBoisblanc B, Connors AF, Jr., Hite RD *et al*: **Comparison of two fluid-management strategies in acute lung injury**. *N Engl J Med* 2006, **354**(24):2564-2575.
5. Macdonald SPJ, Keijzers G, Taylor DM, Kinnear F, Arendts G, Fatovich DM, Bellomo R, McCutcheon D, Fraser JF, Ascencio-Lane JC *et al*: **Restricted fluid resuscitation in suspected sepsis associated hypotension (REFRESH): a pilot randomised controlled trial**. *Intensive care medicine* 2018, **44**(12):2070-2078.
6. Hjortrup PB, Haase N, Bundgaard H, Thomsen SL, Winding R, Pettila V, Aaen A, Lodahl D, Berthelsen RE, Christensen H *et al*: **Restricting volumes of resuscitation fluid in adults with septic shock after initial management: the CLASSIC randomised, parallel-group, multicentre feasibility trial**. *Intensive care medicine* 2016, **42**(11):1695-1705.

7. Balakumar V, Murugan R, Sileanu FE, Palevsky P, Clermont G, Kellum JA: **Both Positive and Negative Fluid Balance May Be Associated With Reduced Long-Term Survival in the Critically Ill**. *Critical care medicine* 2017, **45**(8):e749-e757.
8. **KDIGO Clinical Practice Guideline for Acute Kidney Injury**. *Kidney Int Suppl* 2012, **2**(1):1-138.
9. Payen D, de Pont AC, Sakr Y, Spies C, Reinhart K, Vincent JL, Sepsis Occurrence in Acutely Ill Patients I: **A positive fluid balance is associated with a worse outcome in patients with acute renal failure**. *Critical care (London, England)* 2008, **12**(3):R74.
10. Barbar SD, Clere-Jehl R, Bourredjem A, Hernu R, Montini F, Bruyere R, Lebert C, Bohe J, Badie J, Eraldi JP *et al*: **Timing of Renal-Replacement Therapy in Patients with Acute Kidney Injury and Sepsis**. *N Engl J Med* 2018, **379**(15):1431-1442.
11. Murugan R, Balakumar V, Kerti SJ, Priyanka P, Chang CH, Clermont G, Bellomo R, Palevsky PM, Kellum JA: **Net ultrafiltration intensity and mortality in critically ill patients with fluid overload**. *Critical care (London, England)* 2018, **22**(1):223.
12. Murugan R, Kerti SJ, Chang CH, Gallagher M, Clermont G, Palevsky PM, Kellum JA, Bellomo R: **Association of Net Ultrafiltration Rate With Mortality Among Critically Ill Adults With Acute Kidney Injury Receiving Continuous Venovenous Hemodiafiltration: A Secondary Analysis of the Randomized Evaluation of Normal vs Augmented Level (RENAL) of Renal Replacement Therapy Trial**. *JAMA Netw Open* 2019, **2**(6):e195418.
13. Berthelsen RE, Itenov T, Perner A, Jensen JU, Ibsen M, Jensen AEK, Bestle M: **Forced fluid removal versus usual care in intensive care patients with high-risk acute kidney injury and severe fluid overload (FFAKI): study protocol for a randomised controlled pilot trial**. *Trials* 2017, **18**(1):189.
14. Monnet X, Cipriani F, Camous L, Sentenac P, Dres M, Krastinova E, Anguel N, Richard C, Teboul JL: **The passive leg raising test to guide fluid removal in critically ill patients**. *Ann Intensive Care* 2016, **6**(1):46.
15. Richard JC, Bayle F, Bourdin G, Leray V, Debord S, Delannoy B, Stoian A, Wallet F, Yonis H, Guerin C: **Preload dependence indices to titrate volume expansion during septic shock: a randomized controlled trial**. *Critical care (London, England)* 2015, **19**(1):5.
16. Pinsky MR: **Functional haemodynamic monitoring**. *Curr Opin Crit Care* 2014, **20**(3):288-293.
17. Monnet X, Rienzo M, Osman D, Anguel N, Richard C, Pinsky MR, Teboul JL: **Passive leg raising predicts fluid responsiveness in the critically ill**. *Critical care medicine* 2006, **34**(5):1402-1407.
18. Bitker L, Bayle F, Yonis H, Gobert F, Leray V, Taponnier R, Debord S, Stoian-Cividjian A, Guérin C, Richard J-C: **Prevalence and risk factors of hypotension associated with preload-dependence during intermittent hemodialysis in critically ill patients**. *Critical Care* 2016, **20**(1).
19. Silversides JA, Fitzgerald E, Manickavasagam US, Lapinsky SE, Nisenbaum R, Hemmings N, Nutt C, Trinder TJ, Pogson DG, Fan E *et al*: **Deresuscitation of Patients With Iatrogenic Fluid Overload Is Associated With Reduced Mortality in Critical Illness**. *Critical care medicine* 2018, **46**(10):1600-1607.
20. Investigators RRTS, Bellomo R, Cass A, Cole L, Finfer S, Gallagher M, Lo S, McArthur C, McGuinness S, Myburgh J *et al*: **Intensity of continuous renal-replacement therapy in critically ill patients**. *N Engl J Med* 2009, **361**(17):1627-1638.
21. Burton JO, Jefferies HJ, Selby NM, McIntyre CW: **Hemodialysis-induced repetitive myocardial injury results in global and segmental reduction in systolic cardiac function**. *Clin J Am Soc Nephrol* 2009, **4**(12):1925-1931.
22. Bouchard J, Soroko SB, Chertow GM, Himmelfarb J, Ikizler TA, Paganini EP, Mehta RL, Program to Improve Care in Acute Renal Disease Study G: **Fluid accumulation, survival and recovery of kidney function in critically ill patients with acute kidney injury**. *Kidney Int* 2009, **76**(4):422-427.
23. Prowle JR, Echeverri JE, Ligabo EV, Ronco C, Bellomo R: **Fluid balance and acute kidney injury**. *Nat Rev Nephrol* 2010, **6**(2):107-115.
24. Ichai C, Vinsonneau C, Souweine B, Canet E, Clec'h C, Constantin J, Darmon M, Duranteau J, Gaillot T, Garnier A *et al*: **Recommandations formalisées d’experts : Insuffisance rénale aiguë en périopératoire et en réanimation (à l'exclusion des techniques d'épuration extrarénale)**. In.: RFE commune SFAR-SRLF; 2015.
25. Harel Z, Wald R, Bargman JM, Mamdani M, Etchells E, Garg AX, Ray JG, Luo J, Li P, Quinn RR *et al*: **Nephrologist follow-up improves all-cause mortality of severe acute kidney injury survivors**. *Kidney Int* 2013, **83**(5):901-908.
26. Prowle JR, Kirwan CJ, Bellomo R: **Fluid management for the prevention and attenuation of acute kidney injury**. *Nat Rev Nephrol* 2014, **10**(1):37-47.
27. De Corte W, Dhondt A, Vanholder R, De Waele J, Decruyenaere J, Sergoyne V, Vanhalst J, Claus S, Hoste EA: **Long-term outcome in ICU patients with acute kidney injury treated with renal replacement therapy: a prospective cohort study**. *Critical care (London, England)* 2016, **20**(1):256.
28. Vincent JL, Moreno R, Takala J, Willatts S, De Mendonca A, Bruining H, Reinhart CK, Suter PM, Thijs LG: **The SOFA (Sepsis-related Organ Failure Assessment) score to describe organ dysfunction/failure. On behalf of the Working Group on Sepsis-Related Problems of the European Society of Intensive Care Medicine**. *Intensive care medicine* 1996, **22**(7):707-710.
29. Vinsonneau C, Allain-Launay E, Blayau C, Darmon M, Ducheyron D, Gaillot T, Honore PM, Javouhey E, Krummel T, Lahoche A *et al*: **Renal replacement therapy in adult and pediatric intensive care : Recommendations by an**

expert panel from the French Intensive Care Society (SRLF) with the French Society of Anesthesia Intensive Care (SFAR) French Group for Pediatric Intensive Care Emergencies (GFRUP) the French Dialysis Society (SFD). *Ann Intensive Care* 2015, **5**(1):58.

30. Rhodes A, Evans LE, Alhazzani W, Levy MM, Antonelli M, Ferrer R, Kumar A, Sevransky JE, Sprung CL, Nunnally ME *et al*: **Surviving Sepsis Campaign: International Guidelines for Management of Sepsis and Septic Shock: 2016**. *Intensive care medicine* 2017.
31. Teboul J, Etlegroupedexpertsdelasrlf: **Recommandations d'experts de la SRLF« Indicateurs du remplissage vasculaire au cours de l'insuffisance circulatoire »**. *Réanimation* 2004, **13**(4):255-263.
32. Yehya N, Harhay MO, Curley MAQ, Schoenfeld DA, Reeder RW: **Re-appraisal of Ventilator-free Days in Critical Care Research**. *Am J Respir Crit Care Med* 2019.
33. Monnet X, Persichini R, Ktari M, Jozwiak M, Richard C, Teboul JL: **Precision of the transpulmonary thermodilution measurements**. *Critical care (London, England)* 2011, **15**(4):R204.
34. Guyton AC, Hall JE: **Textbook of medical physiology**, 11th edn. Philadelphia: Elsevier Saunders; 2006.
35. Yonis H, Bitker L, Aublanc M, Perinel Ragey S, Riad Z, Lissonde F, Louf-Durier A, Debord S, Gobert F, Tapponnier R *et al*: **Change in cardiac output during Trendelenburg maneuver is a reliable predictor of fluid responsiveness in patients with acute respiratory distress syndrome in the prone position under protective ventilation**. *Critical Care* 2017, **21**(1).
36. Cavallaro F, Sandroni C, Marano C, La Torre G, Mannocci A, De Waure C, Bello G, Maviglia R, Antonelli M: **Diagnostic accuracy of passive leg raising for prediction of fluid responsiveness in adults: systematic review and meta-analysis of clinical studies**. *Intensive care medicine* 2010, **36**(9):1475-1483.
37. Ait-Oufella H, Lemoinne S, Boelle PY, Galbois A, Baudel JL, Lemant J, Joffre J, Margetis D, Guidet B, Maury E *et al*: **Mottling score predicts survival in septic shock**. *Intensive care medicine* 2011, **37**(5):801-807.
38. van der Voort PH, Boerma EC, Koopmans M, Zandberg M, de Ruiter J, Gerritsen RT, Egbers PH, Kingma WP, Kuiper MA: **Furosemide does not improve renal recovery after hemofiltration for acute renal failure in critically ill patients: a double blind randomized controlled trial**. *Critical care medicine* 2009, **37**(2):533-538.
39. Uchino S, Bellomo R, Morimatsu H, Morgera S, Schetz M, Tan I, Bouman C, Macedo E, Gibney N, Tolwani A *et al*: **Discontinuation of continuous renal replacement therapy: a post hoc analysis of a prospective multicenter observational study**. *Critical care medicine* 2009, **37**(9):2576-2582.
40. Le Gall JR, Lemeshow S, Saulnier F: **A new Simplified Acute Physiology Score (SAPS II) based on a European/North American multicenter study**. *JAMA* 1993, **270**(24):2957-2963.
41. Knaus WA, Wagner DP, Draper EA, Zimmerman JE, Bergner M, Bastos PG, Sirio CA, Murphy DJ, Lotring T, Damiano A *et al*: **The APACHE III prognostic system. Risk prediction of hospital mortality for critically ill hospitalized adults**. *Chest* 1991, **100**(6):1619-1636.
42. Singer M, Deutschman CS, Seymour CW, Shankar-Hari M, Annane D, Bauer M, Bellomo R, Bernard GR, Chiche JD, Coopersmith CM *et al*: **The Third International Consensus Definitions for Sepsis and Septic Shock (Sepsis-3)**. *JAMA* 2016, **315**(8):801-810.
43. Bagshaw SM, Uchino S, Cruz D, Bellomo R, Morimatsu H, Morgera S, Schetz M, Tan I, Bouman C, Macedo E *et al*: **A comparison of observed versus estimated baseline creatinine for determination of RIFLE class in patients with acute kidney injury**. *Nephrol Dial Transplant* 2009, **24**(9):2739-2744.
44. Frat J-P, Thille AW, Mercat A, Girault C, Ragot S, Perbet S, Prat G, Boulain T, Morawiec E, Cottereau A *et al*: **High-Flow Oxygen through Nasal Cannula in Acute Hypoxemic Respiratory Failure**. *New England Journal of Medicine* 2015, **372**(23):2185-2196.
45. Chow S, Shao J, Wang H: **Sample Size Calculations in Clinical Research**, 2nd edn; 2008.
46. Lindstrom ML, Bates DM: **Nonlinear mixed effects models for repeated measures data**. *Biometrics* 1990, **46**(3):673-687.
47. R Development Core Team: **R: A language and environment for statistical computing**. In. Vienna, Austria: R Foundation for Statistical Computing; 2008.
