# Supplementary material for: Fluid balance neutralization secured by hemodynamic monitoring versus protocolized standard of care in critically ill patients requiring continuous renal replacement therapy: study protocol of the GO NEUTRAL randomized controlled trial
Source: Trials. 2022 Sep 22;23:798. doi: 10.1186/s13063-022-06735-6 (PMC9494882; doi:10.1186/s13063-022-06735-6)
Supplement: Supplementary file 2 — Additional file 2: Supplemental material 2. WHO trial registry data set. [file 13063_2022_6735_MOESM2_ESM.pdf]

1. **Primary Registry and Trial Identifying Number**	ClinicalTrials.gov: NCT04801784
2. **Date of Registration in Primary Registry**	March 12, 2021
3. **Secondary Identifying Numbers**	
o Identifiers assigned by the sponsor	Hospices Civils de Lyon: 69HCL20_1243
o Identifiers issued by ethics committees	IDRCB : 2021-A00692-39
4. **Source(s) of Monetary or Material Support**<br>Major source(s) of monetary or material support for the trial (e.g. funding agency, foundation, company, institution).	French Ministry of Health: Inter-regional Hospital Clinical Research Program 2019 (Programme Hospitalier de Recherche Clinique inter-régional 2019)
5. **Primary Sponsor**	Hospices Civils de Lyon
6. **Secondary Sponsor(s)**	None
7. **Contact for Public Queries**	Alexandre PACHOT<br>E-mail: alexandre.pachot@chu-lyon.fr<br>Tel.: 04 72 40 68 40<br>Address : Direction de la Recherche Clinique – Hospices Civils de Lyon. 3 quai des Célestins 69229 Lyon cedex 02, FRANCE
8. **Contact for Scientific Queries**	Principal Investigator<br>Dr Laurent BITKER<br>E-mail: laurent.bitker@chu-lyon.fr<br>Tel.: + 33 4 26 10 94 93<br>Address: Service de Médecine Intensive Réanimation.103 grande rue de la Croix-Rousse. 69004 Lyon, France
9. **Public Title**	Fluid balance neutralization secured by hemodynamic monitoring versus protocolized standard-of-care in critically ill patients requiring continuous renal replacement therapy. Study protocol of the GO NEUTRAL randomized controlled trial
10. **Scientific Title**	Fluid balance neutralization secured by hemodynamic monitoring versus protocolized standard-of-care in critically ill patients requiring continuous renal replacement therapy. Study protocol of the GO NEUTRAL randomized controlled trial
11. **Countries of Recruitment**	France
12. **Health Condition(s) or Problem(s) Studied**	Critically ill patients with stage 3 acute kidney injury treated with continuous renal replacement therapy and vasopressors
13. **Intervention(s)**	Control group: Protocolized control group with no fluid removal during the first 72 hours of study participation, by mean of zero or near-zero net ultrafiltration flow rate.<br><br>Intervention group: Fluid balance neutralization using increased net ultrafiltration, aiming to neutralize the cumulative fluid input received over the first 72 hours of study participation.
14. **Key Inclusion and Exclusion Criteria**<br>Inclusion and exclusion criteria for participant selection, including age and sex. Other selection criteria may relate to clinical diagnosis and co-morbid conditions; exclusion criteria are often used to ensure patient safety.<br><br>If the study is conducted in healthy human volunteers not belonging to the target population (e.g. a preliminary safety study),	Inclusion criteria:<br>- Patients aged 18 years or older, affiliated to social security as per French regulation<br>- Requiring treatment by continuous intravenous infusion of epinephrine or norepinephrine for acute circulatory failure<br>- Presenting with stage 3 acute kidney injury as per the Kidney Disease : Improving Global Outcome guidelines (6)<br>- Treated for less than 24 hours with CRRT at time of eligibility evaluation<br>- And equipped with a continuous cardiac output monitoring device using real-time arterial pulse contour analysis (PiCCO®, Pulsion medical Systems, Feldkirchen, Germany), already in place at time of

enter "healthy human volunteer".	eligibility evaluation<br>Exclusion criteria:<br>- Patient under extra-corporeal membrane oxygenation<br>- Patient with active hemorrhage and receiving blood transfusion<br>- Patient under chronic maintenance dialysis or renal graft recipient<br>- Switch to intermittent RRT scheduled in the 72 hours following inclusion<br>- Ischemic or hemorrhagic cerebral stroke complicated with coma and under mechanical ventilation<br>- Fulminant hepatitis, defined as the coexistence at time of eligibility evaluation of acute liver damage with hepatic encephalopathy, icterus, and decrease in prothrombin ratio < 50% in less than 15 days<br>- Contra-indications to postural maneuvers to assess preload dependence, such as lower limb amputations, inferior vena cava obstruction, abdominal compartment syndrome<br>- Pregnancy or ongoing breastfeeding<br>- Withholding of life support decision regarding mechanic ventilation or resuscitation of cardiac arrest<br>- Moribund patient (expected to die in the next 12 hours)<br>- Patient under legal protective measures<br>- Inclusion in another trial whose primary outcome would be fluid balance, or whose intervention would impact hemodynamic, RRT settings or modifying the fluid balance.<br>- Patient already enrolled in the study
15. **Study Type**<br>Study type consists of:	Multicenter, open labeled, randomized, controlled, superiority trial with parallel groups, and balanced randomization with a 1:1 ratio.
16. **Date of First Enrollment**	31/06/2021
17. **Sample Size**	
o Number of participants that the trial plans to enroll in total.	58
o Number of participants that the trial has enrolled.	45
18. **Recruitment Status**<br>Recruitment status of this trial:	Ongoing recruitment on August 25th, 2022
19. **Primary Outcome(s)**	
o The name of the outcome	Cumulative fluid balance measured between inclusion and 72 hours after inclusion in alive patients at H72
o The metric or method of measurement used (be as specific as possible)	The primary outcome is computed as the difference between fluid input and output, quantified in ml between inclusion and 72 hours after inclusion in alive patients at H72. The primary outcome will be assessed in the modified intention-to-treat (ITT) population (see below for the definition of study populations).
o The timepoint(s) of primary interest	72 hours after inclusion
**Key Secondary Outcomes**<br>Secondary outcomes are outcomes which are of secondary interest or that are measured at timepoints of secondary interest. A secondary outcome may involve the same event, variable, or experience as the primary outcome, but measured at timepoints other than those of primary interest.<br><br>As for primary outcomes, for each secondary outcome provide:	

o The name of the outcome<br>o The metric or method of measurement used<br>o The timepoint(s) of interest	
Outcome #1	• Number of hemodynamic instability episodes<br>• Hemodynamic instability is defined as the occurrence of *de novo* tachycardia (heart rate > 120 bpm), *de novo* hypotension (systolic or mean arterial pressure below clinician-defined target and requiring hemodynamic resuscitation), *de novo* or extension of mottles, or *de novo* decrease in cardiac output (negative change in cardiac index > 15%).<br>• Assessed between inclusion and 72 hours after inclusion
Outcome #2	• Number of hemodynamic instability episodes associated with preload dependence<br>• Hemodynamic instability is defined as the occurrence of *de novo* tachycardia, *de novo* hypotension, *de novo* or extension of mottles, or *de novo* decrease in cardiac output<br>• Assessed between inclusion and 72 hours after inclusion
Outcome #3	• Hemodynamic safety<br>• Mean arterial pressure values, cardiac index, central venous pressure, arterial lactate concentrations and the vasopressor administered dose<br>• Measured between inclusion and 72 hours after inclusion
Outcome #4	• Vasopressor-free days at day 28<br>• Vasopressor weaning will be defined if vasopressors are not required for a continuous period of 48 hours or longer. A value of 0 is allocated if the patients died between inclusion and day 28. A value of 0 is also allocated if the patient is still receiving vasopressors at day 28<br>• Evaluated between inclusion and day 28 of inclusion
Outcome #5	• Organ failure severity<br>• Total SOFA score, composed of the sum of the 6 organ-by-organ sub-scores (neurologic, respiratory, hemodynamic, hepatic, hematologic and renal), and the hemodynamic SOFA subscore<br>• Measured daily between inclusion and 72 hours of inclusion
Outcome #6	• Vital status<br>• Collected from electronic medical records<br>• Assessed at H72 of inclusion, day 28 and day 90 of inclusion
Outcome #7	• Normalized cumulative fluid balance in all included patients<br>• The fluid balance at H24 and H72 will be quantified using the same input and output items as per the primary outcome, normalized by dividing it by the observation period duration. The fluid balance at day 7 will be estimated based on the change in body weight between inclusion and day 7. Fluid balance will be quantified until time point or death, whichever comes first.<br>• Assessed at H24, H72 and day 7 of inclusion
Outcome #8	• Normalized cumulative UF_{NET} volume in all included patients<br>• Cumulative UF_{NET} measured between inclusion and H24 or H72 or death will be normalized by dividing it by the observation period duration, and expressed in $ml.h^{-1}$. The cumulative UF_{NET} volume will be computed based on the UF_{NET} volumes<br>• Assessed at H24 and H72 of inclusion
Outcome #9	• Respiratory efficacy<br>• PaO_2 to FiO_2 ratio and the extravascular lung water index measured with the cardiac output monitoring device

	• Measured once a day from inclusion to H72 or death, whichever comes first.
Outcome #10	• Ventilator-free days at day 28<br>• Mechanical ventilation weaning will be defined if invasive mechanical ventilation is not required for a continuous period of 48 hours or longer. A value of 0 is allocated if the patients died between inclusion and day 28. A value of 0 is also allocated if the patient is still receiving invasive mechanical ventilation at day 28.<br>• Evaluated between inclusion and day 28 of inclusion
Outcome #11	• Major adverse kidney events at day-90 (MAKE-90)<br>• MAKE-90 comprises death before or at day 90, RRT dependence at day 90, persistent stage 2 or 3 acute kidney injury (as per the KDIGO guidelines) at day 90.<br>• Evaluated between inclusion and day 90 of inclusion
Outcome #12	• Hospital and ICU length of stay<br>• Collected from electronic medical records<br>• Time of hospital and ICU discharge respectively, censored at day-90
Outcome #13	• Feasibility<br>• Number of eligible patients per month and per participating centre, and number of patients effectively enrolled in the trial per month and per participating centre<br>• Assessed at time of trial's end
20. **Ethics Review**:	
o Status	Approved
o Date of approval	April 29th, 2019
o Name and contact details of Ethics committee(s)	Comité de Protection des Personnes Sud-Méditerrannée I<br>Hôpital Sainte Marguerite - Bâtiment Direction - 270, Bd Sainte Marguerite - 13274 Marseille Cedex 9<br>Tel.: 04.91.74.42.56<br>Mail : cppsudmed1@gmail.com
21. **Completion date**	Ongoing trial
22. **Summary Results**<br>It consists of:	
o Date of posting of results summaries	Planned on second quarter of 2022
o Date of the first journal publication of results	Planned on third quarter of 2022
o URL hyperlink(s) related to results and publications	Planned on third quarter of 2022
o Baseline Characteristics: Data collected at the beginning of a clinical study for all participants and for each arm or comparison group. These data include demographics, such as age and sex, and study-specific measures.	Will be included in journal publication of the study results
o Participant flow: Information to document the progress and numbers of research participants through each stage of a study in a flow diagram or tabular format.	Will be included in journal publication of the study results
o Adverse events: An unfavorable change in the health of a	Will be included in journal publication of the study results

participant, including abnormal laboratory findings, and all serious adverse events and deaths that happen during a clinical study or within a certain time period after the study has ended. This change may or may not be caused by the intervention being studied.	
o Outcome measures: A table of data for each primary and secondary outcome measure and their respective measurement of precision (eg a 95% confidence interval) by arm (that is, initial assignment of participants to arms or groups) or comparison group (that is, analysis groups), including the result(s) of scientifically appropriate statistical analyses that were performed on the outcome measure data, if any.	Will be included in journal publication of the study results
o URL link to protocol file(s) with version and date	Is included in the current publication
o Brief summary	Will be included in journal publication of the study results
23. **IPD sharing statement** Statement regarding the intended sharing of deidentified individual clinical trial participant-level data (IPD). Should indicate whether or not IPD will be shared, what IPD will be shared, when, by what mechanism, with whom and for what types of analyses. It consists of:	
o Plan to share IPD (Yes, No)	IPD of the whole dataset will be shared upon reasonable request.
o Plan description	Plan description will be shared upon reasonable request.
