# Supplementary material for: Fluid balance neutralization secured by hemodynamic monitoring versus protocolized standard of care in critically ill patients requiring continuous renal replacement therapy: study protocol of the GO NEUTRAL randomized controlled trial
Source: Trials. 2022 Sep 22;23:798. doi: 10.1186/s13063-022-06735-6 (PMC9494882; doi:10.1186/s13063-022-06735-6)
Supplement: Supplementary file 3 — Additional file 3: Supplemental material 3. Study sites, intervention group hemodynamic protocols, participants timelines and study procedures, and list of adverse events. [file 13063_2022_6735_MOESM3_ESM.pdf]

Fluid balance neutralization secured by hemodynamic monitoring versus protocolized standard-of-care in critically ill patients requiring continuous renal replacement therapy. Study protocol of the GO NEUTRAL randomized controlled trial

SUPPLEMENTAL MATERIALS 2

Authors : Laurent Bitker[1,2,3], Pierre Pradat[4], Bertrand Souweine[5], Kada Klouche[6], Julien Illinger[7], Jean-Christophe Richard[1,2,3]

Supplemental Table 1. Study sites

Unit	Hospital	City	Country	Investigators	Contact
Medical Intensive Care Unit	Croix Rousse academic hospital	Lyon	France	Dr Laurent Bitker (PI)<br>Pr J-Christophe Richard (BI)	laurent.bitker@chu-lyon.fr
Intensive Care Unit	Nord-Ouest hospital	Villefranche sur Saône	France	Dr Julien Illinger	
Medical Intensive Care Unit	Lapeyronnie academic hospital	Montpellier	France	Pr Kada Klouche	
Medical Intensive Care Unit	Gabriel Montpied academic hospital	Clermont Ferrand	France	Pr Bertrand Souweine	

BI: back-up principal investigator; PI: principal investigator

Supplemental Figure 1. Intervention hemodynamic protocol 1

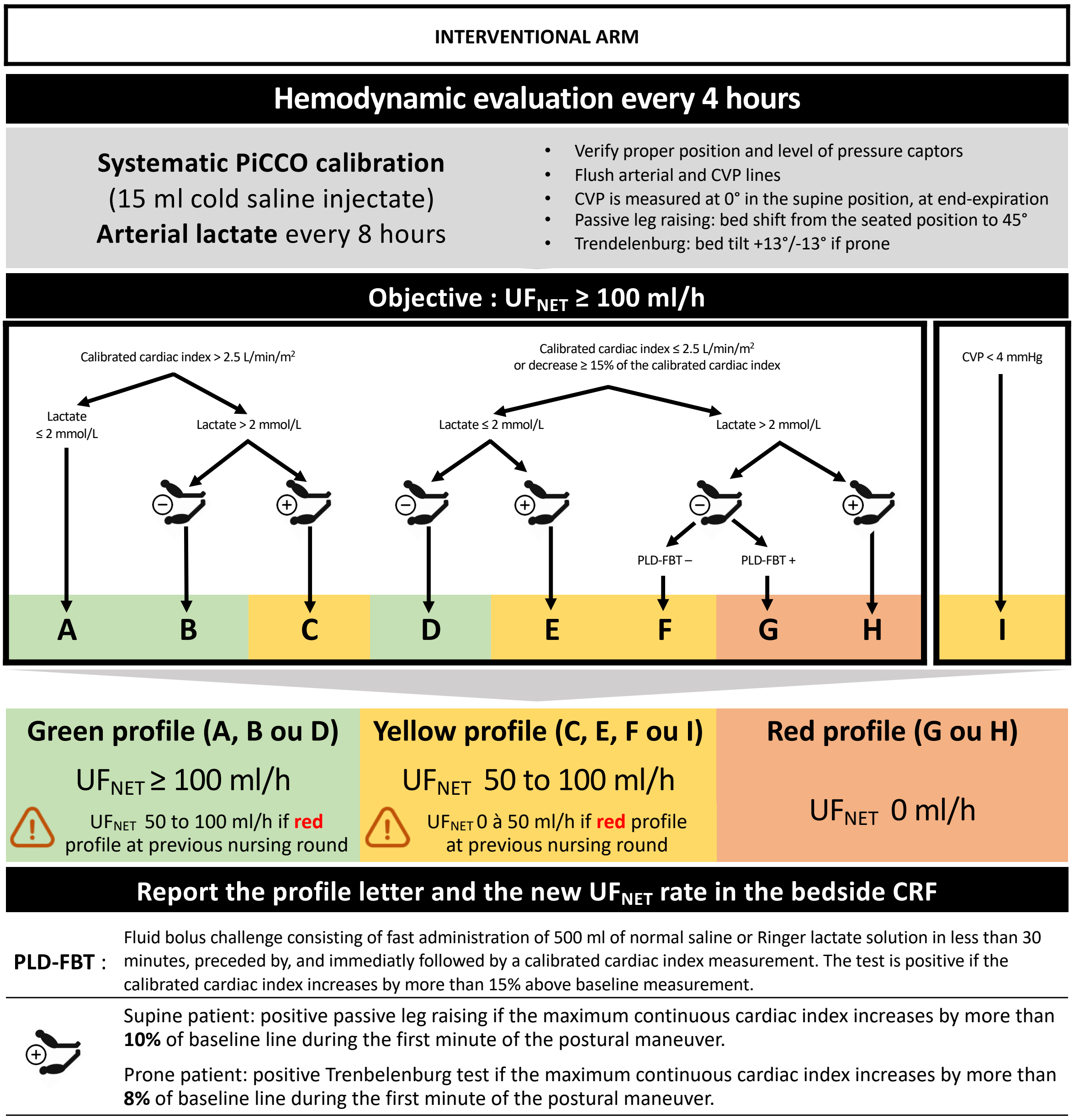

CVP: central venous pressure; PLD-FBT: preload dependence evaluated by a fluid bolus challenge; UF$_{NET}$: net ultrafiltration

Supplemental Figure 2. Intervention hemodynamic protocol 2

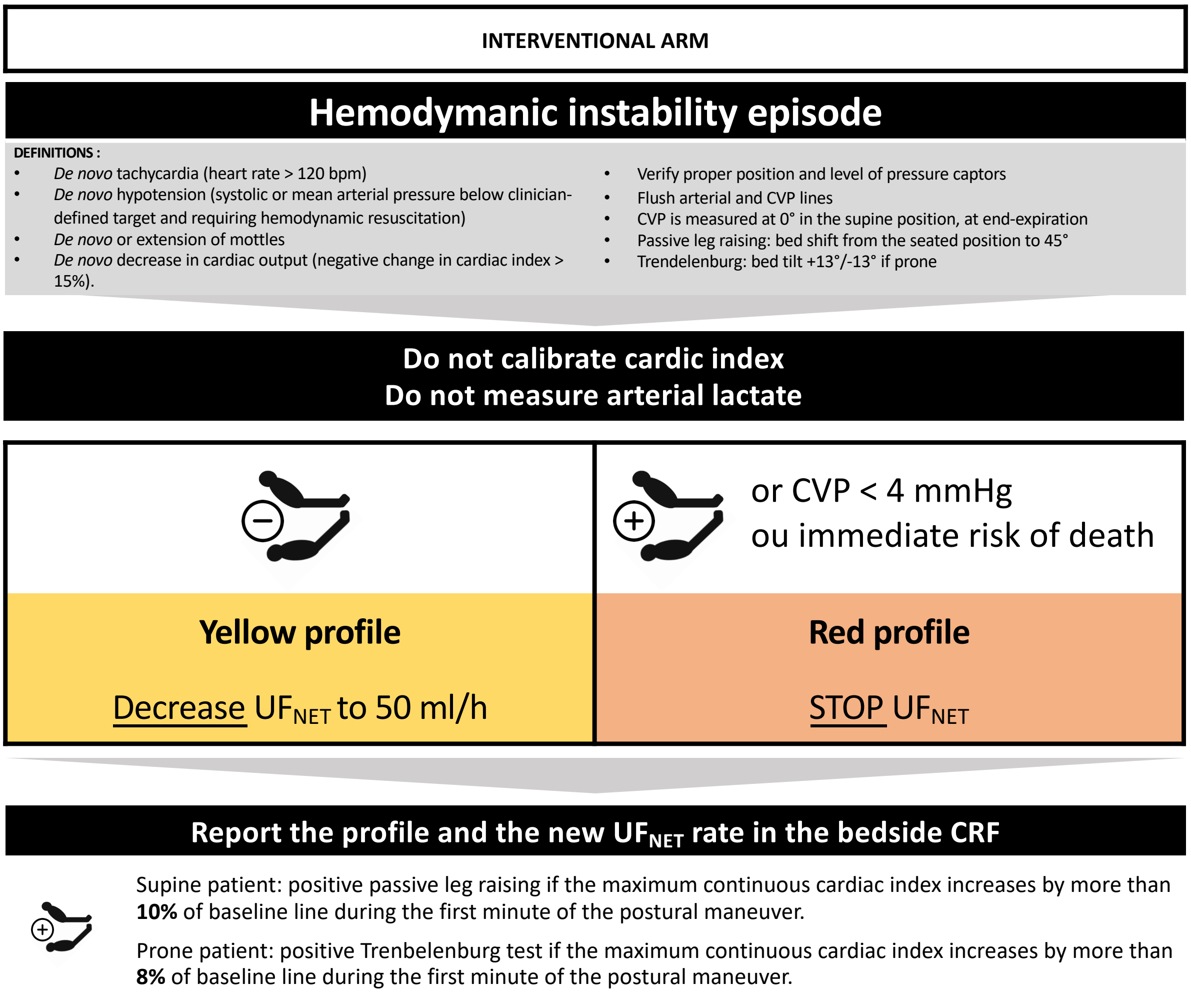

CVP: central venous pressure; UF_{NET}: net ultrafiltration

Supplemental Figure 3. Patient participation timeline

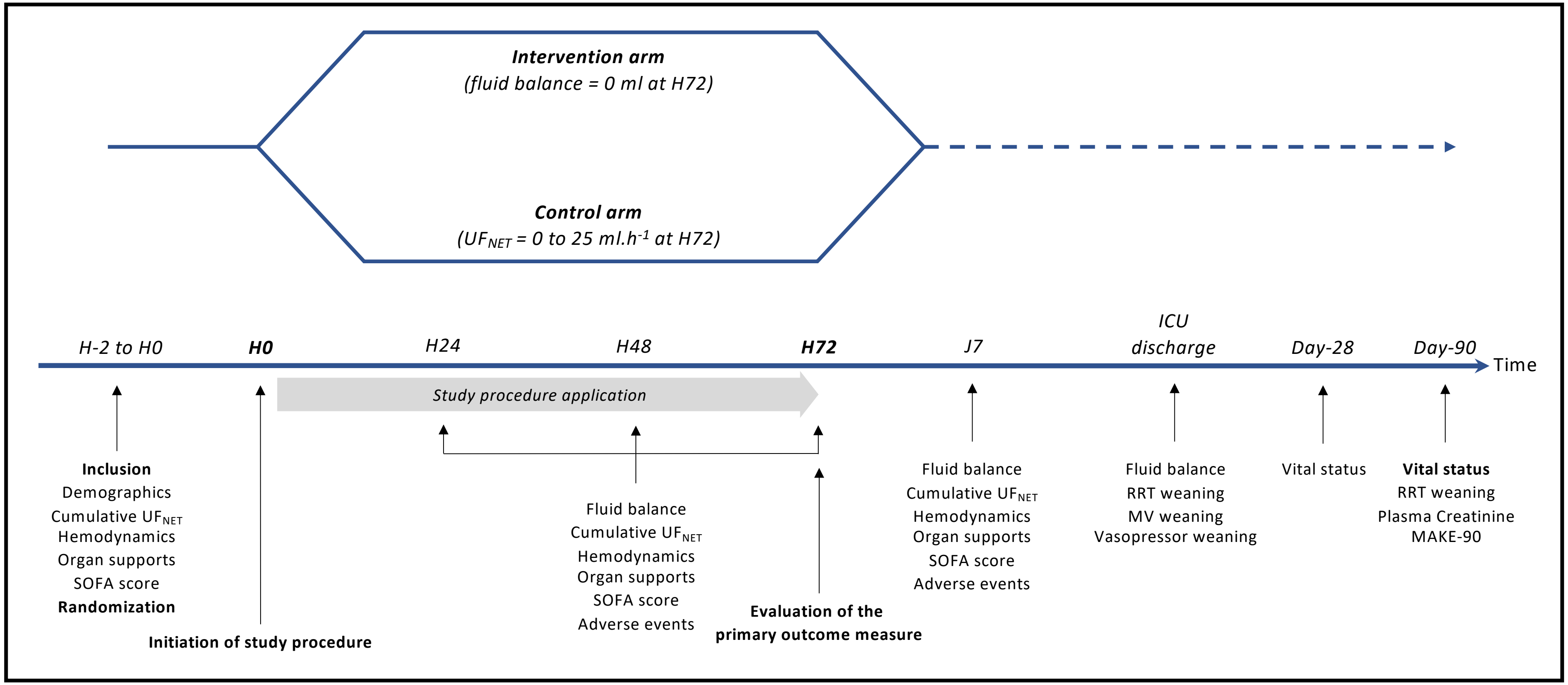

ICU: intensive care unit; MAKE-90: make adverse kidney events at day 90; MV: mechanical ventilation; RRT: renal replacement therapy; SOFA: sepsis-related organ failure assessment; UF_{NET} : net ultrafiltration;

Supplemental Table 2. Study procedures and assessments

			**STUDY PERIOD**						
	Enrolment	*Allocation*	*Post allocation*						
TIMEPOINT	*H-2 to H0*	*H0/Day 1*	*H24*	*H48*	*H72*	*Day-7*	*ICU discharge*	*Day-28*	*Day-90*
ENROLMENT									
Eligibility	*X*								
Informed consent	*X*								
Randomization/allocation		*X*							
INTERVENTIONS									
Intervention		◆———	———	———	———◆				
Control		◆———	———	———	———◆				
ASSESSMENTS									
Demographics		*X*							
Comorbidities		*X*							
Severity of disease		*X*							
Fluid balance (primary outcome)		*X*	*X*	*X*	***X (PO)***	*X*			
Hemodynamic status and support		*X*	*X*	*X*	*X*	*X*			
Hemodynamic profile letter		◆———	———	———	———◆				
Respiratory status and support		*X*	*X*	*X*	*X*	*X*			
CRRT settings, including UF_{NET}		*X*	*X*	*X*	*X*	*X*			
SOFA score (1)		*X*	*X*	*X*	*X*	*X*			
Hemoglobin		*X*	*X*	*X*	*X*	*X*			
ICU discharge assessment							*X*		
Vital status					*X*			*X*	*X*
MAKE-90 (2)									*X*
Adverse events		*X*	*X*	*X*	*X*	*X*	*X*	*X*	*X*

CRRT: continuous renal replacement therapy; ICU: intensive care unit; MAKE-90: majory adverse kidney events at day 90; PO: primary outcome measure; SOFA: sepsis-related organ failure score;

**Supplemental Table 3.** Procedures regarding collection and management of missing values of the components of the primary outcome			
The table aims to resolve cases in which the given item volume is not reported as administered in ICU charts. The table reads itself from left to right, following a step-by-step procedure defined for each item composing the primary outcome measure. Any available flow rate (per day, per minute) should be converted to hourly flow rate (in ml per hour).<br><br>If more than 1 item composing the primary outcome is missing (NA) at a given 4-hourly timepoint, then the fluid balance at this time point will be missing. We will tolerate a maximum of 3 missing 4-hourly fluid balance values for the final calculation of the primary outcome (calculated over 72 hours). In case of more than 3 missing 4-hourly fluid balance values, the primary outcome measure will be missing.			
Input components	Step #1	Step #2	Step #3
IV or oral drugs/electrolytes/vitamins, intermittent administration	Item is prescribed but no volume is reported in the EMR	• If a protocol giving the drug's dilution exists, enter the protocolized dilution volume<br>• If the drug's volume of dilution is fixed by its manufacturer, enter the fixed volume<br>• If the drug's volume is known at its previous administration (max timeframe ≤ 24h), enter its volume	If no information exists regarding the drug volume of dilution, enter NA for the whole item (even if other drugs' volume are known)
IV drugs/electrolytes, continuous administration	Item is prescribed but no volume is reported in the EMR	• If the cumulative volume over 4h is missing, enter the **mean** hourly flow rate (in $ml.h^{-1}$) over the period multiplied by 4 hours (at least 1 flow rate must be available)<br>• If a constant flow rate is fixed by the prescription, enter the flow rate (converted to $ml.h^{-1}$) multiplied by 4 hours<br>• In case of adjustable/varying flow rates (e.g. norepinephrine), and if the flow rate is missing at this timepoint, enter the **preceding** timepoint (max timeframe ≤ 4h) mean hourly flow rate (in $ml.h^{-1}$) multiplied by 4 hours	If no information exists regarding the drug rate of administration or administered volume, enter NA for the whole item (even if other drugs' volume are known)
Enteral or parenteral nutrition, enteral water	Item is prescribed but no volume is reported in the EMR	• If the cumulative volume over 4h is missing, enter the **mean** hourly flow rate (in $ml.h^{-1}$) over the period multiplied by 4 hours (at least 1 flow rate must be available)<br>• If a constant flow rate is given by the prescription, enter the flow rate (converted to $ml.h^{-1}$) multiplied by 4 hours<br>• If the flow rate is missing at this timepoint, enter the **preceding** timepoint (max timeframe ≤ 24h) mean hourly flow rate (in $ml.h^{-1}$) multiplied by 4 hours	If no information exists regarding the nutrition rate of administration or administered volume, enter NA for the whole item (even if other volumes are known)
Fluid bolus therapy	Item is prescribed but no volume is reported in the EMR	• If the administered volume is missing, enter a volume of 500 ml per prescribed FBT	-

Blood products	Item is prescribed but no volume is reported in the EMR	• If the administered volume is missing, enter the volume reported on the French Blood Agency (Etablissement Français du Sang) traceability documents in the patient's medical record	If volume is missing despite step #2, enter NA
IV maintenance hydration	Item is prescribed but no volume is reported in the EMR	• If the cumulative volume over 4h is missing, enter the **mean** hourly flow rate (in $ml.h^{-1}$) over the 4h period multiplied by 4 hours (at least 1 flow rate must be available)<br>• If a constant flow rate is given by the prescription, enter the flow rate (converted to $ml.h^{-1}$) multiplied by 4 hours<br>• If the flow rate is missing at this timepoint, enter the **preceding** timepoint (max timeframe ≤ 24h) mean flow rate (in $ml.h^{-1}$) multiplied by 4 hours	If no information exists regarding the fluid's rate of administration or administered volume, enter NA for the whole item (even if other fluids' volume are known)
Output components			
Urine output	-	If no value appears at the expected timepoint (±2h timeframe), enter NA	-
UF_{NET}	Item is prescribed but no volume is reported in the EMR	If the cumulative volume over 4h is missing, enter the median flow rate (in $ml.h^{-1}$) over the 4h period multiplied by 4 hours	If no information exists regarding the UF_{NET} rate or volume, enter NA
Drains	-	If no value appears at the expected timepoint (±2h timeframe), enter NA	-

IV: intravenous; UF_{NET}: net ultrafiltration

25

**Supplemental Table 4.** List of adverse events based on the CTCAE grading system (3)		
Item	*Immediate reporting to sponsor (CTCAE grade)*	*Reported in the eCRF (CTCAE grade)*
**Death**	**YES**	**YES**
**Non fatal cardiovascular event**		
- De novo septic (4) or vasoplegic shock	**NO** (5)	**YES** (≥3)[a]
- De novo cardiogenic shock	**NO** (5)	**YES** (≥3)[a]
- De novo anaphylactic shock	**NO** (5)	**YES** (≥3)[a]
- De novo hemorrhagic shock	**NO** (5)	**YES** (≥3)[a]
- **De novo hypovolemic non hemorrhagic shock**	**YES** (≥2)	**YES** (≥2)
- De novo obstructive shock	**NO** (5)	**YES** (≥3)[a]
- Worsening hemodynamic status	**NO** (5)	**YES** (≥3)
- Acute cor pulmonale	**NO** (5)	**NO** (5)
- **Cardiac arrest with successful resuscitation**	**YES** (≥2)	**YES** (≥2)
- Supra-ventricular arrythmia	**NO** (5)	**NO** (5)
- Ventricular arrythmia	**NO** (5)	**YES** (≥3)
- Myocardial infarction without ST elevation	**NO** (5)	**YES** (≥4)
- **Myocardial infarction with ST elevation**	**YES** (≥2)	**YES** (≥2)
**Non fatal neurological event**		
- Epileptic seizures	**NO** (5)	**NO** (5)
- Status epilepticus	**NO** (5)	**NO** (5)
- De novo hemorrhagic stroke	**NO** (5)	**YES** (≥3)
- **De novo ischemic stroke**	**YES** (≥2)	**YES** (≥2)
- Coma	**NO** (5)	**NO** (5)
- ICU-acquired neuro-muscular weakness	**NO** (5)	**NO** (5)
**Non fatal respiratory event**		
- **Worsening of respiratory status with mechanical ventilation**	**YES** (≥2)	**YES** (≥2)
- Worsening of respiratory status without mechanical ventilation	**NO** (5)	**YES** (≥4)
- **Hydrostatic acute pulmonary edema**	**YES** (≥2)	**YES** (≥2)
- Atelectasis	**NO** (5)	**NO** (5)
- Pneumothorax or pneumomediastinum	**NO** (5)	**NO** (5)
- Pulmonary embolism	**NO** (5)	**NO** (5)
**Non fatal hepatic event**		
- Hepatic cytolysis	**NO** (5)	**NO** (5)
- Cholestasis	**NO** (5)	**NO** (5)
- Acute liver failure	**NO** (5)	**YES** (≥3)
**Non fatal gastro-intestinal event**		
- Hydrocholecystis	**NO** (5)	**NO** (5)
- Cholecystitis	**NO** (5)	**NO** (5)
- Esophagitis	**NO** (5)	**NO** (5)
- Gastro-intestinal stress ulcer	**NO** (5)	**YES** (≥3)
- **Acute mesenteric ischemia**	**YES** (≥2)	**YES** (≥2)
- Ischemic colitis	**NO** (5)	**YES** (≥3)
- Peritonitis	**NO** (5)	**NO** (5)
**Non fatal renal event**		
- KDIGO stage 3 acute kidney injury (2)	**NO** (5)	**NO** (5)[b]
- KDIGO stage 1 ou 2 acute kidney injury (2)	**NO** (5)	**NO** (5)
**Non fatal RRT-related event**		
- Dialysis catheter-related hemorrhage	**NO** (5)	**NO** (5)
- Dialysis catheter-related deep vein thrombosis	**NO** (5)	**NO** (5)
- RRT extra-corporeal circulation thrombosis	**NO** (5)	**NO** (5)
**Non fatal metabolic event**		
- Hypokalemia < 3.5 mmol/L	**NO** (5)	**NO** (5)

- Hyperkalemia > 5 mmol/L	**NO** (5)	**NO** (5)
- Hyponatremia < 135 mmol/L	**NO** (5)	**NO** (5)
- Hypernatremia > 145 mmol/L	**NO** (5)	**NO** (5)
- Dyscalcemia	**NO** (5)	**NO** (5)
- Dysphosphoremia ou dysmagnesemia	**NO** (5)	**NO** (5)
- Severe metabolic alcalosis (pH > 7.60 and HCO_3^- > 40 mmol/L and PaCO2 ≤ 45 mmHg)	**NO** (5)	**YES** (≥3)
- Non severe metabolic alcalosis	**NO** (5)	**NO** (5)
- Respiratory or mixt alcalosis	**NO** (5)	**NO** (5)
- Lactic acidosis	**NO** (5)	**NO** (5)
- Other acidosis	**NO** (5)	**NO** (5)
- Dysglycemia	**NO** (5)	**NO** (5)
**Non fatal hematologic event**		
- Anemia < 120 g/L	**NO** (5)	**NO** (5)
- Thrombopenia < 150 G/L	**NO** (5)	**NO** (5)
- Leucopenia < 4000 G/L	**NO** (5)	**NO** (5)
**Non fatal infectious event**		
- Catheter-related bloodstream infection	**NO** (5)	**YES** (≥4)
- Documented ventilator-acquired pneumonia	**NO** (5)	**YES** (≥4)
- Non-documented ventilator-acquired pneumonia	**NO** (5)	**NO** (5)
- Documented bloodstream infection (2 sites)	**NO** (5)	**YES** (≥4)
- Other bloodstream infection	**NO** (5)	**NO** (5)
- Other ICU-acquired documented infections	**NO** (5)	**YES** (≥4)
**Other non fatal event**		
- Deep vein thrombosis	**NO** (5)	**NO** (5)
- **De novo limb acute ischemia**	**YES** (≥2)	**YES** (≥2)
- **Other de novo organ acute ischemia**	**YES** (≥2)	**YES** (≥2)
- **Any non fatal adverse events of grade 3 or more not listed above**	**YES** (≥3)	**YES** (≥3)
- Any non fatal adverse events of grade 2 or less not listed above	**NO**	**NO**

Items in bold are adverse events of special interest. The grade in parenthesis in each column indicates the CTCAE grade at which the adverse events should be reported and/or collected.
a. Only if the initial cause of acute circulatory failure has resolved.
b. Inclusion criterion.
CTCAE: common terminology criteria for adverse events; eCRF: electronic case report form

References

1. Vincent JL, Moreno R, Takala J, Willatts S, De Mendonca A, Bruining H, et al. The SOFA (Sepsis-related Organ Failure Assessment) score to describe organ dysfunction/failure. On behalf of the Working Group on Sepsis-Related Problems of the European Society of Intensive Care Medicine. Intensive care medicine. 1996;22(7):707-10.
2. KDIGO Clinical Practice Guideline for Acute Kidney Injury. Kidney Int Suppl. 2012;2(1):1-138.
3. National Cancer Institute. Common terminology criteria for adverse events : (CTCAE). 2010.
4. Singer M, Deutschman CS, Seymour CW, Shankar-Hari M, Annane D, Bauer M, et al. The Third International Consensus Definitions for Sepsis and Septic Shock (Sepsis-3). JAMA. 2016;315(8):801-10.
