# Supplementary material for: Fluid balance neutralization secured by hemodynamic monitoring versus protocolized standard of care in critically ill patients requiring continuous renal replacement therapy: study protocol of the GO NEUTRAL randomized controlled trial
Source: Trials. 2022 Sep 22;23:798. doi: 10.1186/s13063-022-06735-6 (PMC9494882; doi:10.1186/s13063-022-06735-6)
Supplement: Supplementary file 4 — Additional file 4: Supplemental material 4. Bedside case report form of the intervention group (French). [file 13063_2022_6735_MOESM4_ESM.pdf]

Etude GO-NEUTRAL

Cahier IDE

Version 1 du 09/06/2021

BRAS INTERVENTIONNEL

Merci de ne pas jeter ce document

Centre de l'étude :

____

Identifiant du patient pour l'étude : |_|-|_|_|-|_|-|_|
(lettre du centre, numéro d'inclusion, initiales)

Ce cahier contient 2 sections :

- **Section 1** : monitorage par 4h de H4 à H72
- **Section 2** : report des épisodes d'instabilité hémodynamiques de H0 à H72

Section 1

Monitorage par 4 heures

H4 à H24

TOUTES LES 4 HEURES

Date de la visite (jj/mm/aaaa) |_|_|/|_|_|/|_|_|_|_|
Heure de la visite |_|_|:|_|_|

Temps de la visite

J1	☒ H4	☐ H8	☐ H12	☐ H16	☐ H20	☐ **H24**
J2	☐ H28	☐ H32	☐ H36	☐ H40	☐ H44	☐ **H48**
J3	☐ H52	☐ H56	☐ H60	☐ H64	☐ H68	☐ **H72**

Rappels :

- Lactates artériels dans les 8 heures précédentes, selon indication du clinicien
- Vérifier position des capteurs, fixés au bras du patient (point phlébostatique)
- Rincer les lignes artérielle et de PVC (flush)
- PVC à mesurer en décubitus, à 0°, et en fin d'expiration
- **Calibration systématique du PiCCO** (3 injections de 15 ml de SSI froid)
- Lever de jambe passif : bascule complète du lit depuis la position assise à 45° pendant 1 minute
- Trendelenburg si DV : bascule du lit de + 13° à – 13°

Apparition ou extension de marbrures ☐ Oui ☐ Non

Fréquence cardiaque |_|_|_| bpm
Pression artérielle moyenne |_|_|_| mmHg
Index cardiaque calibré par thermodilution |_|_|,|_| L/min/m²
Pression veineuse centrale |_|_| mmHg

Acide lactique artériel (dans les 8h précédentes) |_|_|,|_| mmol/L

Débit d'UF nette en cours (mettre 0 si EER suspendue) |_|_|_|_| ml/h

Appliquer le protocole hémodynamique

ICC **avant** manœuvre posturale |_|_|,|_| L/min/m²
ICC **après** manœuvre posturale |_|_|,|_| L/min/m²
*Le test est positif si l'**index cardiaque continu** augmente de plus de 10% lors du lever de jambe passif, ou de plus de 8% lors du Trendelenburg*

Lettre du profil hémodynamique : |_|
Débit d'UF nette réglée à la suite de l'évaluation (ml/h) |_|_|_|_|

Profil F ou G ? (= profil à haut risque) : ☐ Oui ☐ Non
IC par thermodilution avant remplissage |_|_|,|_| L/min/m²
IC par thermodilution après remplissage |_|_|,|_| L/min/m²

TOUTES LES 4 HEURES

Date de la visite (jj/mm/aaaa) |_|_|/|_|_|/|_|_|_|_|
Heure de la visite |_|_|:|_|_|

Temps de la visite

J1	☐ H4	☒ H8	☐ H12	☐ H16	☐ H20	☐ **H24**
J2	☐ H28	☐ H32	☐ H36	☐ H40	☐ H44	☐ **H48**
J3	☐ H52	☐ H56	☐ H60	☐ H64	☐ H68	☐ **H72**

Rappels :

- Lactates artériels dans les 8 heures précédentes, selon indication du clinicien
- Vérifier position des capteurs, fixés au bras du patient (point phlébostatique)
- Rincer les lignes artérielle et de PVC (flush)
- PVC à mesurer en décubitus, à 0°, et en fin d'expiration
- **Calibration systématique du PiCCO** (3 injections de 15 ml de SSI froid)
- Lever de jambe passif : bascule complète du lit depuis la position assise à 45° pendant 1 minute
- Trendelenburg si DV : bascule du lit de + 13° à – 13°

Apparition ou extension de marbrures ☐ Oui ☐ Non

Fréquence cardiaque |_|_|_| bpm
Pression artérielle moyenne |_|_|_| mmHg
Index cardiaque calibré par thermodilution |_|_|,|_| L/min/m²
Pression veineuse centrale |_|_| mmHg

Acide lactique artériel (dans les 8h précédentes) |_|_|,|_| mmol/L

Débit d'UF nette en cours (mettre 0 si EER suspendue) |_|_|_|_| ml/h

Appliquer le protocole hémodynamique

ICC **avant** manœuvre posturale |_|_|,|_| L/min/m²
ICC **après** manœuvre posturale |_|_|,|_| L/min/m²

*Le test est positif si l'**index cardiaque continu** augmente de plus de 10% lors du lever de jambe passif, ou de plus de 8% lors du Trendelenburg*

Lettre du profil hémodynamique : |_|
Débit d'UF nette réglée à la suite de l'évaluation (ml/h) |_|_|_|_|

Profil F ou G ? (= profil à haut risque) : ☐ Oui ☐ Non
IC par thermodilution avant remplissage |_|_|,|_| L/min/m²
IC par thermodilution après remplissage |_|_|,|_| L/min/m²

TOUTES LES 4 HEURES

Date de la visite (jj/mm/aaaa) |_|_|/|_|_|/|_|_|_|_|
Heure de la visite |_|_|:|_|_|

Temps de la visite

J1	☐ H4	☐ H8	☒ H12	☐ H16	☐ H20	☐ **H24**
J2	☐ H28	☐ H32	☐ H36	☐ H40	☐ H44	☐ **H48**
J3	☐ H52	☐ H56	☐ H60	☐ H64	☐ H68	☐ **H72**

Rappels :

- Lactates artériels dans les 8 heures précédentes, selon indication du clinicien
- Vérifier position des capteurs, fixés au bras du patient (point phlébostatique)
- Rincer les lignes artérielle et de PVC (flush)
- PVC à mesurer en décubitus, à 0°, et en fin d'expiration
- **Calibration systématique du PiCCO** (3 injections de 15 ml de SSI froid)
- Lever de jambe passif : bascule complète du lit depuis la position assise à 45° pendant 1 minute
- Trendelenburg si DV : bascule du lit de + 13° à – 13°

Apparition ou extension de marbrures ☐ Oui ☐ Non

Fréquence cardiaque |_|_|_| bpm
Pression artérielle moyenne |_|_|_| mmHg
Index cardiaque calibré par thermodilution |_|_|,|_| L/min/m²
Pression veineuse centrale |_|_| mmHg

Acide lactique artériel (dans les 8h précédentes) |_|_|,|_| mmol/L

Débit d'UF nette en cours (mettre 0 si EER suspendue) |_|_|_|_| ml/h

Appliquer le protocole hémodynamique

ICC **avant** manœuvre posturale |_|_|,|_| L/min/m²
ICC **après** manœuvre posturale |_|_|,|_| L/min/m²

*Le test est positif si l'**index cardiaque continu** augmente de plus de 10% lors du lever de jambe passif, ou de plus de 8% lors du Trendelenburg*

Lettre du profil hémodynamique : |_|
Débit d'UF nette réglée à la suite de l'évaluation (ml/h) |_|_|_|_|

Profil F ou G ? (= profil à haut risque) : ☐ Oui ☐ Non
IC par thermodilution avant remplissage |_|_|,|_| L/min/m²
IC par thermodilution après remplissage |_|_|,|_| L/min/m²

TOUTES LES 4 HEURES

Date de la visite (jj/mm/aaaa) |_|_|/|_|_|/|_|_|_|_|
Heure de la visite |_|_|:|_|_|

Temps de la visite

J1	☐ H4	☐ H8	☐ H12	☒ H16	☐ H20	☐ **H24**
J2	☐ H28	☐ H32	☐ H36	☐ H40	☐ H44	☐ **H48**
J3	☐ H52	☐ H56	☐ H60	☐ H64	☐ H68	☐ **H72**

Rappels :

- Lactates artériels dans les 8 heures précédentes, selon indication du clinicien
- Vérifier position des capteurs, fixés au bras du patient (point phlébostatique)
- Rincer les lignes artérielle et de PVC (flush)
- PVC à mesurer en décubitus, à 0°, et en fin d'expiration
- **Calibration systématique du PiCCO** (3 injections de 15 ml de SSI froid)
- Lever de jambe passif : bascule complète du lit depuis la position assise à 45° pendant 1 minute
- Trendelenburg si DV : bascule du lit de + 13° à – 13°

Apparition ou extension de marbrures ☐ Oui ☐ Non

Fréquence cardiaque |_|_|_| bpm
Pression artérielle moyenne |_|_|_| mmHg
Index cardiaque calibré par thermodilution |_|_|,|_| L/min/m^2
Pression veineuse centrale |_|_| mmHg

Acide lactique artériel (dans les 8h précédentes) |_|_|,|_| mmol/L

Débit d'UF nette en cours (mettre 0 si EER suspendue) |_|_|_|_| ml/h

Appliquer le protocole hémodynamique

ICC **avant** manœuvre posturale |_|_|,|_| L/min/m^2
ICC **après** manœuvre posturale |_|_|,|_| L/min/m^2
*Le test est positif si l'**index cardiaque continu** augmente de plus de 10% lors du lever de jambe passif, ou de plus de 8% lors du Trendelenburg*

Lettre du profil hémodynamique : |_|
Débit d'UF nette réglée à la suite de l'évaluation (ml/h) |_|_|_|_|

Profil F ou G ? (= profil à haut risque) : ☐ Oui ☐ Non
IC par thermodilution avant remplissage |_|_|,|_| L/min/m^2
IC par thermodilution après remplissage |_|_|,|_| L/min/m^2

TOUTES LES 4 HEURES

Date de la visite (jj/mm/aaaa) |_|_|/|_|_|/|_|_|_|_|
Heure de la visite |_|_|:|_|_|

Temps de la visite

J1	☐ H4	☐ H8	☐ H12	☐ H16	☒ H20	☐ **H24**
J2	☐ H28	☐ H32	☐ H36	☐ H40	☐ H44	☐ **H48**
J3	☐ H52	☐ H56	☐ H60	☐ H64	☐ H68	☐ **H72**

Rappels :

- Lactates artériels dans les 8 heures précédentes, selon indication du clinicien
- Vérifier position des capteurs, fixés au bras du patient (point phlébostatique)
- Rincer les lignes artérielle et de PVC (flush)
- PVC à mesurer en décubitus, à 0°, et en fin d'expiration
- **Calibration systématique du PiCCO** (3 injections de 15 ml de SSI froid)
- Lever de jambe passif : bascule complète du lit depuis la position assise à 45° pendant 1 minute
- Trendelenburg si DV : bascule du lit de + 13° à – 13°

Apparition ou extension de marbrures ☐ Oui ☐ Non

Fréquence cardiaque |_|_|_| bpm
Pression artérielle moyenne |_|_|_| mmHg
Index cardiaque calibré par thermodilution |_|_|,|_| L/min/m²
Pression veineuse centrale |_|_| mmHg

Acide lactique artériel (dans les 8h précédentes) |_|_|,|_| mmol/L

Débit d'UF nette en cours (mettre 0 si EER suspendue) |_|_|_|_| ml/h

Appliquer le protocole hémodynamique

ICC **avant** manœuvre posturale |_|_|,|_| L/min/m²
ICC **après** manœuvre posturale |_|_|,|_| L/min/m²
*Le test est positif si l'**index cardiaque continu** augmente de plus de 10% lors du lever de jambe passif, ou de plus de 8% lors du Trendelenburg*

Lettre du profil hémodynamique : |_|
Débit d'UF nette réglée à la suite de l'évaluation (ml/h) |_|_|_|_|

Profil F ou G ? (= profil à haut risque) : ☐ Oui ☐ Non
IC par thermodilution avant remplissage |_|_|,|_| L/min/m²
IC par thermodilution après remplissage |_|_|,|_| L/min/m²

TOUTES LES 4 HEURES

Date de la visite (jj/mm/aaaa) |_|_|/|_|_|/|_|_|_|_|
Heure de la visite |_|_|:|_|_|

Temps de la visite

J1	☐ H4	☐ H8	☐ H12	☐ H16	☐ H20	☒ **H24**
J2	☐ H28	☐ H32	☐ H36	☐ H40	☐ H44	☐ **H48**
J3	☐ H52	☐ H56	☐ H60	☐ H64	☐ H68	☐ **H72**

Rappels :

- Lactates artériels dans les 8 heures précédentes, selon indication du clinicien
- Vérifier position des capteurs, fixés au bras du patient (point phlébostatique)
- Rincer les lignes artérielle et de PVC (flush)
- PVC à mesurer en décubitus, à 0°, et en fin d'expiration
- **Calibration systématique du PiCCO** (3 injections de 15 ml de SSI froid)
- Lever de jambe passif : bascule complète du lit depuis la position assise à 45° pendant 1 minute
- Trendelenburg si DV : bascule du lit de + 13° à – 13°

Apparition ou extension de marbrures ☐ Oui ☐ Non

Fréquence cardiaque |_|_|_| bpm
Pression artérielle moyenne |_|_|_| mmHg
Index cardiaque calibré par thermodilution |_|_|,|_| L/min/m²
Pression veineuse centrale |_|_| mmHg

Acide lactique artériel (dans les 8h précédentes) |_|_|,|_| mmol/L

Débit d'UF nette en cours (mettre 0 si EER suspendue) |_|_|_|_| ml/h

Appliquer le protocole hémodynamique

ICC **avant** manœuvre posturale |_|_|,|_| L/min/m²
ICC **après** manœuvre posturale |_|_|,|_| L/min/m²
*Le test est positif si l'**index cardiaque continu** augmente de plus de 10% lors du lever de jambe passif, ou de plus de 8% lors du Trendelenburg*

Lettre du profil hémodynamique : |_|
Débit d'UF nette réglée à la suite de l'évaluation (ml/h) |_|_|_|_|

Profil F ou G ? (= profil à haut risque) : ☐ Oui ☐ Non
IC par thermodilution avant remplissage |_|_|,|_| L/min/m²
IC par thermodilution après remplissage |_|_|,|_| L/min/m²

H28 à H48

TOUTES LES 4 HEURES

Date de la visite (jj/mm/aaaa) |_|_|/|_|_|/|_|_|_|_|
Heure de la visite |_|_|:|_|_|

Temps de la visite

J1	☐ H4	☐ H8	☐ H12	☐ H16	☐ H20	☐ **H24**
J2	☒ H28	☐ H32	☐ H36	☐ H40	☐ H44	☐ **H48**
J3	☐ H52	☐ H56	☐ H60	☐ H64	☐ H68	☐ **H72**

Rappels :

- Lactates artériels dans les 8 heures précédentes, selon indication du clinicien
- Vérifier position des capteurs, fixés au bras du patient (point phlébostatique)
- Rincer les lignes artérielle et de PVC (flush)
- PVC à mesurer en décubitus, à 0°, et en fin d'expiration
- **Calibration systématique du PiCCO** (3 injections de 15 ml de SSI froid)
- Lever de jambe passif : bascule complète du lit depuis la position assise à 45° pendant 1 minute
- Trendelenburg si DV : bascule du lit de + 13° à – 13°

Apparition ou extension de marbrures ☐ Oui ☐ Non

Fréquence cardiaque |_|_|_| bpm
Pression artérielle moyenne |_|_|_| mmHg
Index cardiaque calibré par thermodilution |_|_|,|_| L/min/m²
Pression veineuse centrale |_|_| mmHg

Acide lactique artériel (dans les 8h précédentes) |_|_|,|_| mmol/L

Débit d'UF nette en cours (mettre 0 si EER suspendue) |_|_|_|_| ml/h

Appliquer le protocole hémodynamique

ICC **avant** manœuvre posturale |_|_|,|_| L/min/m²
ICC **après** manœuvre posturale |_|_|,|_| L/min/m²

*Le test est positif si l'**index cardiaque continu** augmente de plus de 10% lors du lever de jambe passif, ou de plus de 8% lors du Trendelenburg*

Lettre du profil hémodynamique : |_|
Débit d'UF nette réglée à la suite de l'évaluation (ml/h) |_|_|_|_|

Profil F ou G ? (= profil à haut risque) : ☐ Oui ☐ Non
IC par thermodilution avant remplissage |_|_|,|_| L/min/m²
IC par thermodilution après remplissage |_|_|,|_| L/min/m²

TOUTES LES 4 HEURES

Date de la visite (jj/mm/aaaa)	|_|_|/|_|_|/|_|_|_|_|
Heure de la visite	|_|_|:|_|_|

Temps de la visite

J1	☐ H4	☐ H8	☐ H12	☐ H16	☐ H20	☐ **H24**
J2	☐ H28	☒ H32	☐ H36	☐ H40	☐ H44	☐ **H48**
J3	☐ H52	☐ H56	☐ H60	☐ H64	☐ H68	☐ **H72**

Rappels :

- Lactates artériels dans les 8 heures précédentes, selon indication du clinicien
- Vérifier position des capteurs, fixés au bras du patient (point phlébostatique)
- Rincer les lignes artérielle et de PVC (flush)
- PVC à mesurer en décubitus, à 0°, et en fin d'expiration
- **Calibration systématique du PiCCO** (3 injections de 15 ml de SSI froid)
- Lever de jambe passif : bascule complète du lit depuis la position assise à 45° pendant 1 minute
- Trendelenburg si DV : bascule du lit de + 13° à – 13°

Apparition ou extension de marbrures	☐ Oui ☐ Non
Fréquence cardiaque	|_|_|_| bpm
Pression artérielle moyenne	|_|_|_| mmHg
Index cardiaque calibré par thermodilution	|_|_|,|_| L/min/m²
Pression veineuse centrale	|_|_| mmHg
Acide lactique artériel (dans les 8h précédentes)	|_|_|,|_| mmol/L
Débit d'UF nette en cours (mettre 0 si EER suspendue)	|_|_|_|_| ml/h

Appliquer le protocole hémodynamique

ICC **avant** manœuvre posturale	|_|_|,|_| L/min/m²
ICC **après** manœuvre posturale	|_|_|,|_| L/min/m²

*Le test est positif si l'**index cardiaque continu** augmente de plus de 10% lors du lever de jambe passif, ou de plus de 8% lors du Trendelenburg*

Lettre du profil hémodynamique :	|_|
Débit d'UF nette réglée à la suite de l'évaluation (ml/h)	|_|_|_|_|

**Profil F ou G ? (= profil à haut risque)** :	☐ Oui ☐ Non
IC par thermodilution avant remplissage	|_|_|,|_| L/min/m²
IC par thermodilution après remplissage	|_|_|,|_| L/min/m²

TOUTES LES 4 HEURES

Date de la visite (jj/mm/aaaa) |_|_|/|_|_|/|_|_|_|_|
Heure de la visite |_|_|:|_|_|

Temps de la visite

J1	☐ H4	☐ H8	☐ H12	☐ H16	☐ H20	☐ **H24**
J2	☐ H28	☐ H32	☒ H36	☐ H40	☐ H44	☐ **H48**
J3	☐ H52	☐ H56	☐ H60	☐ H64	☐ H68	☐ **H72**

Rappels :

- Lactates artériels dans les 8 heures précédentes, selon indication du clinicien
- Vérifier position des capteurs, fixés au bras du patient (point phlébostatique)
- Rincer les lignes artérielle et de PVC (flush)
- PVC à mesurer en décubitus, à 0°, et en fin d'expiration
- **Calibration systématique du PiCCO** (3 injections de 15 ml de SSI froid)
- Lever de jambe passif : bascule complète du lit depuis la position assise à 45° pendant 1 minute
- Trendelenburg si DV : bascule du lit de + 13° à – 13°

Apparition ou extension de marbrures ☐ Oui ☐ Non

Fréquence cardiaque |_|_|_| bpm
Pression artérielle moyenne |_|_|_| mmHg
Index cardiaque calibré par thermodilution |_|_|,|_| L/min/m²
Pression veineuse centrale |_|_| mmHg

Acide lactique artériel (dans les 8h précédentes) |_|_|,|_| mmol/L

Débit d'UF nette en cours (mettre 0 si EER suspendue) |_|_|_|_| ml/h

Appliquer le protocole hémodynamique

ICC **avant** manœuvre posturale |_|_|,|_| L/min/m²
ICC **après** manœuvre posturale |_|_|,|_| L/min/m²

*Le test est positif si l'**index cardiaque continu** augmente de plus de 10% lors du lever de jambe passif, ou de plus de 8% lors du Trendelenburg*

Lettre du profil hémodynamique : |_|
Débit d'UF nette réglée à la suite de l'évaluation (ml/h) |_|_|_|_|

Profil F ou G ? (= profil à haut risque) : ☐ Oui ☐ Non
IC par thermodilution avant remplissage |_|_|,|_| L/min/m²
IC par thermodilution après remplissage |_|_|,|_| L/min/m²

TOUTES LES 4 HEURES

Date de la visite (jj/mm/aaaa) |_|_|/|_|_|/|_|_|_|_|
Heure de la visite |_|_|:|_|_|

Temps de la visite

J1	☐ H4	☐ H8	☐ H12	☐ H16	☐ H20	☐ **H24**
J2	☐ H28	☐ H32	☐ H36	☒ H40	☐ H44	☐ **H48**
J3	☐ H52	☐ H56	☐ H60	☐ H64	☐ H68	☐ **H72**

Rappels :

- Lactates artériels dans les 8 heures précédentes, selon indication du clinicien
- Vérifier position des capteurs, fixés au bras du patient (point phlébostatique)
- Rincer les lignes artérielle et de PVC (flush)
- PVC à mesurer en décubitus, à 0°, et en fin d'expiration
- **Calibration systématique du PiCCO** (3 injections de 15 ml de SSI froid)
- Lever de jambe passif : bascule complète du lit depuis la position assise à 45° pendant 1 minute
- Trendelenburg si DV : bascule du lit de + 13° à – 13°

Apparition ou extension de marbrures ☐ Oui ☐ Non

Fréquence cardiaque |_|_|_| bpm
Pression artérielle moyenne |_|_|_| mmHg
Index cardiaque calibré par thermodilution |_|_|,|_| L/min/m²
Pression veineuse centrale |_|_| mmHg

Acide lactique artériel (dans les 8h précédentes) |_|_|,|_| mmol/L

Débit d'UF nette en cours (mettre 0 si EER suspendue) |_|_|_|_| ml/h

Appliquer le protocole hémodynamique

ICC **avant** manœuvre posturale |_|_|,|_| L/min/m²
ICC **après** manœuvre posturale |_|_|,|_| L/min/m²
*Le test est positif si l'**index cardiaque continu** augmente de plus de 10% lors du lever de jambe passif, ou de plus de 8% lors du Trendelenburg*

Lettre du profil hémodynamique : |_|
Débit d'UF nette réglée à la suite de l'évaluation (ml/h) |_|_|_|_|

Profil F ou G ? (= profil à haut risque) : ☐ Oui ☐ Non
IC par thermodilution avant remplissage |_|_|,|_| L/min/m²
IC par thermodilution après remplissage |_|_|,|_| L/min/m²

TOUTES LES 4 HEURES

Date de la visite (jj/mm/aaaa)	|_|_|/|_|_|/|_|_|_|_|
Heure de la visite	|_|_|:|_|_|

Temps de la visite

J1	☐ H4	☐ H8	☐ H12	☐ H16	☐ H20	☐ **H24**
J2	☐ H28	☐ H32	☐ H36	☐ H40	☒ H44	☐ **H48**
J3	☐ H52	☐ H56	☐ H60	☐ H64	☐ H68	☐ **H72**

Rappels :

- Lactates artériels dans les 8 heures précédentes, selon indication du clinicien
- Vérifier position des capteurs, fixés au bras du patient (point phlébostatique)
- Rincer les lignes artérielle et de PVC (flush)
- PVC à mesurer en décubitus, à 0°, et en fin d'expiration
- **Calibration systématique du PiCCO** (3 injections de 15 ml de SSI froid)
- Lever de jambe passif : bascule complète du lit depuis la position assise à 45° pendant 1 minute
- Trendelenburg si DV : bascule du lit de + 13° à – 13°

Apparition ou extension de marbrures	☐ Oui ☐ Non
Fréquence cardiaque	|_|_|_| bpm
Pression artérielle moyenne	|_|_|_| mmHg
Index cardiaque calibré par thermodilution	|_|_|,|_| L/min/m²
Pression veineuse centrale	|_|_| mmHg
Acide lactique artériel (dans les 8h précédentes)	|_|_|,|_| mmol/L
Débit d'UF nette en cours (mettre 0 si EER suspendue)	|_|_|_|_| ml/h

Appliquer le protocole hémodynamique

ICC **avant** manœuvre posturale	|_|_|,|_| L/min/m²
ICC **après** manœuvre posturale	|_|_|,|_| L/min/m²

*Le test est positif si l'**index cardiaque continu** augmente de plus de 10% lors du lever de jambe passif, ou de plus de 8% lors du Trendelenburg*

Lettre du profil hémodynamique :	|_|
Débit d'UF nette réglée à la suite de l'évaluation (ml/h)	|_|_|_|_|

**Profil F ou G ? (= profil à haut risque)** :	☐ Oui ☐ Non
IC par thermodilution avant remplissage	|_|_|,|_| L/min/m²
IC par thermodilution après remplissage	|_|_|,|_| L/min/m²

TOUTES LES 4 HEURES

Date de la visite (jj/mm/aaaa) |_|_|/|_|_|/|_|_|_|_|
Heure de la visite |_|_|:|_|_|

Temps de la visite

J1	☐ H4	☐ H8	☐ H12	☐ H16	☐ H20	☐ **H24**
J2	☐ H28	☐ H32	☐ H36	☐ H40	☐ H44	☒ **H48**
J3	☐ H52	☐ H56	☐ H60	☐ H64	☐ H68	☐ **H72**

Rappels :

- Lactates artériels dans les 8 heures précédentes, selon indication du clinicien
- Vérifier position des capteurs, fixés au bras du patient (point phlébostatique)
- Rincer les lignes artérielle et de PVC (flush)
- PVC à mesurer en décubitus, à 0°, et en fin d'expiration
- **Calibration systématique du PiCCO** (3 injections de 15 ml de SSI froid)
- Lever de jambe passif : bascule complète du lit depuis la position assise à 45° pendant 1 minute
- Trendelenburg si DV : bascule du lit de + 13° à – 13°

Apparition ou extension de marbrures ☐ Oui ☐ Non

Fréquence cardiaque |_|_|_| bpm
Pression artérielle moyenne |_|_|_| mmHg
Index cardiaque calibré par thermodilution |_|_|,|_| L/min/m²
Pression veineuse centrale |_|_| mmHg

Acide lactique artériel (dans les 8h précédentes) |_|_|,|_| mmol/L

Débit d'UF nette en cours (mettre 0 si EER suspendue) |_|_|_|_| ml/h

Appliquer le protocole hémodynamique

ICC **avant** manœuvre posturale |_|_|,|_| L/min/m²
ICC **après** manœuvre posturale |_|_|,|_| L/min/m²
*Le test est positif si l'**index cardiaque continu** augmente de plus de 10% lors du lever de jambe passif, ou de plus de 8% lors du Trendelenburg*

Lettre du profil hémodynamique : |_|
Débit d'UF nette réglée à la suite de l'évaluation (ml/h) |_|_|_|_|

Profil F ou G ? (= profil à haut risque) : ☐ Oui ☐ Non
IC par thermodilution avant remplissage |_|_|,|_| L/min/m²
IC par thermodilution après remplissage |_|_|,|_| L/min/m²

H52 à H72

TOUTES LES 4 HEURES

Date de la visite (jj/mm/aaaa) |_|_|/|_|_|/|_|_|_|_|
Heure de la visite |_|_|:|_|_|

Temps de la visite

J1	☐ H4	☐ H8	☐ H12	☐ H16	☐ H20	☐ **H24**
J2	☐ H28	☐ H32	☐ H36	☐ H40	☐ H44	☐ **H48**
J3	☒ H52	☐ H56	☐ H60	☐ H64	☐ H68	☐ **H72**

Rappels :

- Lactates artériels dans les 8 heures précédentes, selon indication du clinicien
- Vérifier position des capteurs, fixés au bras du patient (point phlébostatique)
- Rincer les lignes artérielle et de PVC (flush)
- PVC à mesurer en décubitus, à 0°, et en fin d'expiration
- **Calibration systématique du PiCCO** (3 injections de 15 ml de SSI froid)
- Lever de jambe passif : bascule complète du lit depuis la position assise à 45° pendant 1 minute
- Trendelenburg si DV : bascule du lit de + 13° à – 13°

Apparition ou extension de marbrures ☐ Oui ☐ Non

Fréquence cardiaque |_|_|_| bpm
Pression artérielle moyenne |_|_|_| mmHg
Index cardiaque calibré par thermodilution |_|_|,|_| L/min/m²
Pression veineuse centrale |_|_| mmHg

Acide lactique artériel (dans les 8h précédentes) |_|_|,|_| mmol/L

Débit d'UF nette en cours (mettre 0 si EER suspendue) |_|_|_|_| ml/h

Appliquer le protocole hémodynamique

ICC **avant** manœuvre posturale |_|_|,|_| L/min/m²
ICC **après** manœuvre posturale |_|_|,|_| L/min/m²

*Le test est positif si l'**index cardiaque continu** augmente de plus de 10% lors du lever de jambe passif, ou de plus de 8% lors du Trendelenburg*

Lettre du profil hémodynamique : |_|
Débit d'UF nette réglée à la suite de l'évaluation (ml/h) |_|_|_|_|

Profil F ou G ? (= profil à haut risque) : ☐ Oui ☐ Non
IC par thermodilution avant remplissage |_|_|,|_| L/min/m²
IC par thermodilution après remplissage |_|_|,|_| L/min/m²

TOUTES LES 4 HEURES

Date de la visite (jj/mm/aaaa) |_|_|/|_|_|/|_|_|_|_|
Heure de la visite |_|_|:|_|_|

Temps de la visite

J1	☐ H4	☐ H8	☐ H12	☐ H16	☐ H20	☐ **H24**
J2	☐ H28	☐ H32	☐ H36	☐ H40	☐ H44	☐ **H48**
J3	☐ H52	☒ H56	☐ H60	☐ H64	☐ H68	☐ **H72**

Rappels :

- Lactates artériels dans les 8 heures précédentes, selon indication du clinicien
- Vérifier position des capteurs, fixés au bras du patient (point phlébostatique)
- Rincer les lignes artérielle et de PVC (flush)
- PVC à mesurer en décubitus, à 0°, et en fin d'expiration
- **Calibration systématique du PiCCO** (3 injections de 15 ml de SSI froid)
- Lever de jambe passif : bascule complète du lit depuis la position assise à 45° pendant 1 minute
- Trendelenburg si DV : bascule du lit de + 13° à – 13°

Apparition ou extension de marbrures ☐ Oui ☐ Non

Fréquence cardiaque |_|_|_| bpm
Pression artérielle moyenne |_|_|_| mmHg
Index cardiaque calibré par thermodilution |_|_|,|_| L/min/m²
Pression veineuse centrale |_|_| mmHg

Acide lactique artériel (dans les 8h précédentes) |_|_|,|_| mmol/L

Débit d'UF nette en cours (mettre 0 si EER suspendue) |_|_|_|_| ml/h

Appliquer le protocole hémodynamique

ICC **avant** manœuvre posturale |_|_|,|_| L/min/m²
ICC **après** manœuvre posturale |_|_|,|_| L/min/m²

*Le test est positif si l'**index cardiaque continu** augmente de plus de 10% lors du lever de jambe passif, ou de plus de 8% lors du Trendelenburg*

Lettre du profil hémodynamique : |_|

Débit d'UF nette réglée à la suite de l'évaluation (ml/h) |_|_|_|_|

Profil F ou G ? (= profil à haut risque) : ☐ Oui ☐ Non

IC par thermodilution avant remplissage |_|_|,|_| L/min/m²
IC par thermodilution après remplissage |_|_|,|_| L/min/m²

TOUTES LES 4 HEURES

Date de la visite (jj/mm/aaaa) |_|_|/|_|_|/|_|_|_|_|
Heure de la visite |_|_|:|_|_|

Temps de la visite

J1	☐ H4	☐ H8	☐ H12	☐ H16	☐ H20	☐ **H24**
J2	☐ H28	☐ H32	☐ H36	☐ H40	☐ H44	☐ **H48**
J3	☐ H52	☐ H56	☒ H60	☐ H64	☐ H68	☐ **H72**

Rappels :

- Lactates artériels dans les 8 heures précédentes, selon indication du clinicien
- Vérifier position des capteurs, fixés au bras du patient (point phlébostatique)
- Rincer les lignes artérielle et de PVC (flush)
- PVC à mesurer en décubitus, à 0°, et en fin d'expiration
- **Calibration systématique du PiCCO** (3 injections de 15 ml de SSI froid)
- Lever de jambe passif : bascule complète du lit depuis la position assise à 45° pendant 1 minute
- Trendelenburg si DV : bascule du lit de + 13° à – 13°

Apparition ou extension de marbrures ☐ Oui ☐ Non

Fréquence cardiaque |_|_|_| bpm
Pression artérielle moyenne |_|_|_| mmHg
Index cardiaque calibré par thermodilution |_|_|,|_| L/min/m²
Pression veineuse centrale |_|_| mmHg

Acide lactique artériel (dans les 8h précédentes) |_|_|,|_| mmol/L

Débit d'UF nette en cours (mettre 0 si EER suspendue) |_|_|_|_| ml/h

Appliquer le protocole hémodynamique

ICC **avant** manœuvre posturale |_|_|,|_| L/min/m²
ICC **après** manœuvre posturale |_|_|,|_| L/min/m²
*Le test est positif si l'**index cardiaque continu** augmente de plus de 10% lors du lever de jambe passif, ou de plus de 8% lors du Trendelenburg*

Lettre du profil hémodynamique : |_|
Débit d'UF nette réglée à la suite de l'évaluation (ml/h) |_|_|_|_|

Profil F ou G ? (= profil à haut risque) : ☐ Oui ☐ Non
IC par thermodilution avant remplissage |_|_|,|_| L/min/m²
IC par thermodilution après remplissage |_|_|,|_| L/min/m²

TOUTES LES 4 HEURES

Date de la visite (jj/mm/aaaa) |_|_|/|_|_|/|_|_|_|_|
Heure de la visite |_|_|:|_|_|

Temps de la visite

J1	☐ H4	☐ H8	☐ H12	☐ H16	☐ H20	☐ **H24**
J2	☐ H28	☐ H32	☐ H36	☐ H40	☐ H44	☐ **H48**
J3	☐ H52	☐ H56	☐ H60	☒ H64	☐ H68	☐ **H72**

Rappels :

- Lactates artériels dans les 8 heures précédentes, selon indication du clinicien
- Vérifier position des capteurs, fixés au bras du patient (point phlébostatique)
- Rincer les lignes artérielle et de PVC (flush)
- PVC à mesurer en décubitus, à 0°, et en fin d'expiration
- **Calibration systématique du PiCCO** (3 injections de 15 ml de SSI froid)
- Lever de jambe passif : bascule complète du lit depuis la position assise à 45° pendant 1 minute
- Trendelenburg si DV : bascule du lit de + 13° à – 13°

Apparition ou extension de marbrures ☐ Oui ☐ Non

Fréquence cardiaque |_|_|_| bpm
Pression artérielle moyenne |_|_|_| mmHg
Index cardiaque calibré par thermodilution |_|_|,|_| L/min/m^2
Pression veineuse centrale |_|_| mmHg

Acide lactique artériel (dans les 8h précédentes) |_|_|,|_| mmol/L

Débit d'UF nette en cours (mettre 0 si EER suspendue) |_|_|_|_| ml/h

Appliquer le protocole hémodynamique

ICC **avant** manœuvre posturale |_|_|,|_| L/min/m^2
ICC **après** manœuvre posturale |_|_|,|_| L/min/m^2

*Le test est positif si l'**index cardiaque continu** augmente de plus de 10% lors du lever de jambe passif, ou de plus de 8% lors du Trendelenburg*

Lettre du profil hémodynamique : |_|
Débit d'UF nette réglée à la suite de l'évaluation (ml/h) |_|_|_|_|

Profil F ou G ? (= profil à haut risque) : ☐ Oui ☐ Non
IC par thermodilution avant remplissage |_|_|,|_| L/min/m^2
IC par thermodilution après remplissage |_|_|,|_| L/min/m^2

TOUTES LES 4 HEURES

Date de la visite (jj/mm/aaaa) |_|_|/|_|_|/|_|_|_|_|
Heure de la visite |_|_|:|_|_|

Temps de la visite

J1	☐ H4	☐ H8	☐ H12	☐ H16	☐ H20	☐ **H24**
J2	☐ H28	☐ H32	☐ H36	☐ H40	☐ H44	☐ **H48**
J3	☐ H52	☐ H56	☐ H60	☐ H64	☒ H68	☐ **H72**

Rappels :

- Lactates artériels dans les 8 heures précédentes, selon indication du clinicien
- Vérifier position des capteurs, fixés au bras du patient (point phlébostatique)
- Rincer les lignes artérielle et de PVC (flush)
- PVC à mesurer en décubitus, à 0°, et en fin d'expiration
- **Calibration systématique du PiCCO** (3 injections de 15 ml de SSI froid)
- Lever de jambe passif : bascule complète du lit depuis la position assise à 45° pendant 1 minute
- Trendelenburg si DV : bascule du lit de + 13° à – 13°

Apparition ou extension de marbrures ☐ Oui ☐ Non

Fréquence cardiaque |_|_|_| bpm
Pression artérielle moyenne |_|_|_| mmHg
Index cardiaque calibré par thermodilution |_|_|,|_| L/min/m²
Pression veineuse centrale |_|_| mmHg

Acide lactique artériel (dans les 8h précédentes) |_|_|,|_| mmol/L

Débit d'UF nette en cours (mettre 0 si EER suspendue) |_|_|_|_| ml/h

Appliquer le protocole hémodynamique

ICC **avant** manœuvre posturale |_|_|,|_| L/min/m²
ICC **après** manœuvre posturale |_|_|,|_| L/min/m²

*Le test est positif si l'**index cardiaque continu** augmente de plus de 10% lors du lever de jambe passif, ou de plus de 8% lors du Trendelenburg*

Lettre du profil hémodynamique : |_|
Débit d'UF nette réglée à la suite de l'évaluation (ml/h) |_|_|_|_|

Profil F ou G ? (= profil à haut risque) : ☐ Oui ☐ Non
IC par thermodilution avant remplissage |_|_|,|_| L/min/m²
IC par thermodilution après remplissage |_|_|,|_| L/min/m²

TOUTES LES 4 HEURES

Date de la visite (jj/mm/aaaa) |_|_|/|_|_|/|_|_|_|_|
Heure de la visite |_|_|:|_|_|

Temps de la visite

J1	☐ H4	☐ H8	☐ H12	☐ H16	☐ H20	☐ **H24**
J2	☐ H28	☐ H32	☐ H36	☐ H40	☐ H44	☐ **H48**
J3	☐ H52	☐ H56	☐ H60	☐ H64	☐ H68	☒ **H72**

Rappels :

- Lactates artériels dans les 8 heures précédentes, selon indication du clinicien
- Vérifier position des capteurs, fixés au bras du patient (point phlébostatique)
- Rincer les lignes artérielle et de PVC (flush)
- PVC à mesurer en décubitus, à 0°, et en fin d'expiration
- **Calibration systématique du PiCCO** (3 injections de 15 ml de SSI froid)
- Lever de jambe passif : bascule complète du lit depuis la position assise à 45° pendant 1 minute
- Trendelenburg si DV : bascule du lit de + 13° à – 13°

Apparition ou extension de marbrures ☐ Oui ☐ Non

Fréquence cardiaque |_|_|_| bpm
Pression artérielle moyenne |_|_|_| mmHg
Index cardiaque calibré par thermodilution |_|_|,|_| L/min/m²
Pression veineuse centrale |_|_| mmHg

Acide lactique artériel (dans les 8h précédentes) |_|_|,|_| mmol/L

Débit d'UF nette en cours (mettre 0 si EER suspendue) |_|_|_|_| ml/h

Appliquer le protocole hémodynamique

ICC **avant** manœuvre posturale |_|_|,|_| L/min/m²
ICC **après** manœuvre posturale |_|_|,|_| L/min/m²
*Le test est positif si l'**index cardiaque continu** augmente de plus de 10% lors du lever de jambe passif, ou de plus de 8% lors du Trendelenburg*

Lettre du profil hémodynamique : |_|
Débit d'UF nette réglée à la suite de l'évaluation (ml/h) |_|_|_|_|

Profil F ou G ? (= profil à haut risque) : ☐ Oui ☐ Non
IC par thermodilution avant remplissage |_|_|,|_| L/min/m²
IC par thermodilution après remplissage |_|_|,|_| L/min/m²

Section 2

Épisodes d’instabilité hémodynamique

EPISODE D'INSTABILITE HEMODYNAMIQUE (de H0 à H72)

Date de la visite (jj/mm/aaaa) |_|_|/|_|_|/|_|_|_|_|
Heure survenue de l'épisode |_|_|:|_|_|
Numéro de l'épisode |_|_|_|

Rappels :
- Pas plus de 1 évaluation par heure
- **Pas de calibration ou de mesure des lactates en situation urgente**
- Vérifier position des capteurs, fixés au bras du patient (point phlébostatique)
- Rincer les lignes artérielle et de PVC (flush)
- PVC à mesurer en décubitus, à 0°, et en fin d'expiration
- Lever de jambe passif : bascule complète du lit depuis la position assise à 45° pendant 1 minute
- Trendelenburg si DV : bascule du lit de + 13° à – 13°

Caractéristiques de l'épisode

Hypotension artérielle justifiant d'une intervention	☐ Oui	☐ Non
Tachycardie > 120 battements par minute	☐ Oui	☐ Non
Baisse de l'index cardiaque continu > 15%	☐ Oui	☐ Non
Apparition ou extension de marbrures	☐ Oui	☐ Non

Variables hémodynamiques

Fréquence cardiaque	|_|_|_| bpm
Pression artérielle moyenne	|_|_|_| mmHg
Index cardiaque continu	|_|_|,|_| L/min/m²
Pression veineuse centrale	|_|_| mmHg

Epuration extra-rénale

Débit d'UF nette en cours au moment de l'épisode |_|_|_|_| ml/h

ICC **avant** manœuvre posturale (L/min/m²) |_|_|,|_| L/min/m²
ICC **après** manœuvre posturale (L/min/m²) |_|_|,|_| L/min/m²

*Positif si l'**index cardiaque continu** augmente de plus de 10% lors du lever de jambe passif, ou de plus de 8% lors du Trendelenburg*

Appliquer le protocole INSTABILITE

Profil : ☐ **JAUNE** ☐ **ROUGE**

Débit d'UF nette réglée à la suite de l'évaluation |_|_|_|_| ml/h

EPISODE D'INSTABILITE HEMODYNAMIQUE
(de H0 à H72)

Date de la visite (jj/mm/aaaa) |_|_|/|_|_|/|_|_|_|_|
Heure survenue de l'épisode |_|_|:|_|_|
Numéro de l'épisode |_|_|_|

Rappels :

- Pas plus de 1 évaluation par heure
- **Pas de calibration ou de mesure des lactates en situation urgente**
- Vérifier position des capteurs, fixés au bras du patient (point phlébostatique)
- Rincer les lignes artérielle et de PVC (flush)
- PVC à mesurer en décubitus, à 0°, et en fin d'expiration
- Lever de jambe passif : bascule complète du lit depuis la position assise à 45° pendant 1 minute
- Trendelenburg si DV : bascule du lit de + 13° à – 13°

Caractéristiques de l'épisode

Hypotension artérielle justifiant d'une intervention	☐ Oui	☐ Non
Tachycardie > 120 battements par minute	☐ Oui	☐ Non
Baisse de l'index cardiaque continu > 15%	☐ Oui	☐ Non
Apparition ou extension de marbrures	☐ Oui	☐ Non

Variables hémodynamiques

Fréquence cardiaque	|_|_|_| bpm
Pression artérielle moyenne	|_|_|_| mmHg
Index cardiaque continu	|_|_|,|_| L/min/m²
Pression veineuse centrale	|_|_| mmHg

Epuration extra-rénale

Débit d'UF nette en cours au moment de l'épisode |_|_|_|_| ml/h

ICC **avant** manœuvre posturale (L/min/m²) |_|_|,|_| L/min/m²
ICC **après** manœuvre posturale (L/min/m²) |_|_|,|_| L/min/m²

*Positif si l'**index cardiaque continu** augmente de plus de 10% lors du lever de jambe passif, ou de plus de 8% lors du Trendelenburg*

Appliquer le protocole INSTABILITE

Profil : ☐ **JAUNE** ☐ **ROUGE**

Débit d'UF nette réglée à la suite de l'évaluation |_|_|_|_| ml/h

EPISODE D'INSTABILITE HEMODYNAMIQUE
(de H0 à H72)

Date de la visite (jj/mm/aaaa) |_|_|/|_|_|/|_|_|_|_|
Heure survenue de l'épisode |_|_|:|_|_|
Numéro de l'épisode |_|_|_|

Rappels :

- Pas plus de 1 évaluation par heure
- **Pas de calibration ou de mesure des lactates en situation urgente**
- Vérifier position des capteurs, fixés au bras du patient (point phlébostatique)
- Rincer les lignes artérielle et de PVC (flush)
- PVC à mesurer en décubitus, à 0°, et en fin d'expiration
- Lever de jambe passif : bascule complète du lit depuis la position assise à 45° pendant 1 minute
- Trendelenburg si DV : bascule du lit de + 13° à – 13°

Caractéristiques de l'épisode

Hypotension artérielle justifiant d'une intervention	☐ Oui	☐ Non
Tachycardie > 120 battements par minute	☐ Oui	☐ Non
Baisse de l'index cardiaque continu > 15%	☐ Oui	☐ Non
Apparition ou extension de marbrures	☐ Oui	☐ Non

Variables hémodynamiques

Fréquence cardiaque	|_|_|_| bpm
Pression artérielle moyenne	|_|_|_| mmHg
Index cardiaque continu	|_|_|,|_| L/min/m^2
Pression veineuse centrale	|_|_| mmHg

Epuration extra-rénale

Débit d'UF nette en cours au moment de l'épisode |_|_|_|_| ml/h

ICC **avant** manœuvre posturale (L/min/m^2) |_|_|,|_| L/min/m^2
ICC **après** manœuvre posturale (L/min/m^2) |_|_|,|_| L/min/m^2

*Positif si l'**index cardiaque continu** augmente de plus de 10% lors du lever de jambe passif, ou de plus de 8% lors du Trendelenburg*

Appliquer le protocole INSTABILITE

Profil : ☐ **JAUNE** ☐ **ROUGE**

Débit d'UF nette réglée à la suite de l'évaluation |_|_|_|_| ml/h

EPISODE D'INSTABILITE HEMODYNAMIQUE (de H0 à H72)

Date de la visite (jj/mm/aaaa) |_|_|/|_|_|/|_|_|_|_|
Heure survenue de l'épisode |_|_|:|_|_|
Numéro de l'épisode |_|_|_|

Rappels :

- Pas plus de 1 évaluation par heure
- **Pas de calibration ou de mesure des lactates en situation urgente**
- Vérifier position des capteurs, fixés au bras du patient (point phlébostatique)
- Rincer les lignes artérielle et de PVC (flush)
- PVC à mesurer en décubitus, à 0°, et en fin d'expiration
- Lever de jambe passif : bascule complète du lit depuis la position assise à 45° pendant 1 minute
- Trendelenburg si DV : bascule du lit de + 13° à – 13°

Caractéristiques de l'épisode

Hypotension artérielle justifiant d'une intervention	☐ Oui	☐ Non
Tachycardie > 120 battements par minute	☐ Oui	☐ Non
Baisse de l'index cardiaque continu > 15%	☐ Oui	☐ Non
Apparition ou extension de marbrures	☐ Oui	☐ Non

Variables hémodynamiques

Fréquence cardiaque	|_|_|_| bpm
Pression artérielle moyenne	|_|_|_| mmHg
Index cardiaque continu	|_|_|,|_| L/min/m^2
Pression veineuse centrale	|_|_| mmHg

Epuration extra-rénale

Débit d'UF nette en cours au moment de l'épisode |_|_|_|_| ml/h

ICC **avant** manœuvre posturale (L/min/m^2) |_|_|,|_| L/min/m^2
ICC **après** manœuvre posturale (L/min/m^2) |_|_|,|_| L/min/m^2

*Positif si l'**index cardiaque continu** augmente de plus de 10% lors du lever de jambe passif, ou de plus de 8% lors du Trendelenburg*

Appliquer le protocole INSTABILITE

Profil : ☐ **JAUNE** ☐ **ROUGE**
Débit d'UF nette réglée à la suite de l'évaluation |_|_|_|_| ml/h

EPISODE D'INSTABILITE HEMODYNAMIQUE
(de H0 à H72)

Date de la visite (jj/mm/aaaa) |_|_|/|_|_|/|_|_|_|_|
Heure survenue de l'épisode |_|_|:|_|_|
Numéro de l'épisode |_|_|_|

Rappels :
- <u>Pas plus de 1 évaluation par heure</u>
- **Pas de calibration ou de mesure des lactates en situation urgente**
- Vérifier position des capteurs, fixés au bras du patient (point phlébostatique)
- Rincer les lignes artérielle et de PVC (flush)
- PVC à mesurer en décubitus, à 0°, et en fin d'expiration
- Lever de jambe passif : bascule complète du lit depuis la position assise à 45° pendant 1 minute
- Trendelenburg si DV : bascule du lit de + 13° à – 13°

Caractéristiques de l'épisode

Hypotension artérielle justifiant d'une intervention	☐ Oui	☐ Non
Tachycardie > 120 battements par minute	☐ Oui	☐ Non
Baisse de l'index cardiaque continu > 15%	☐ Oui	☐ Non
Apparition ou extension de marbrures	☐ Oui	☐ Non

Variables hémodynamiques

Fréquence cardiaque |_|_|_| bpm
Pression artérielle moyenne |_|_|_| mmHg
Index cardiaque continu |_|_|,|_| L/min/m^2
Pression veineuse centrale |_|_| mmHg

Epuration extra-rénale

Débit d'UF nette en cours au moment de l'épisode |_|_|_|_| ml/h

ICC **avant** manœuvre posturale (L/min/m^2) |_|_|,|_| L/min/m^2
ICC **après** manœuvre posturale (L/min/m^2) |_|_|,|_| L/min/m^2
*Positif si l'**index cardiaque continu** augmente de plus de 10% lors du lever de jambe passif, ou de plus de 8% lors du Trendelenburg*

Appliquer le protocole INSTABILITE

Profil : ☐ **JAUNE** ☐ **ROUGE**
Débit d'UF nette réglée à la suite de l'évaluation |_|_|_|_| ml/h

EPISODE D'INSTABILITE HEMODYNAMIQUE
(de H0 à H72)

Date de la visite (jj/mm/aaaa) |_|_|/|_|_|/|_|_|_|_|
Heure survenue de l'épisode |_|_|:|_|_|
Numéro de l'épisode |_|_|_|

Rappels :

- Pas plus de 1 évaluation par heure
- **Pas de calibration ou de mesure des lactates en situation urgente**
- Vérifier position des capteurs, fixés au bras du patient (point phlébostatique)
- Rincer les lignes artérielle et de PVC (flush)
- PVC à mesurer en décubitus, à 0°, et en fin d'expiration
- Lever de jambe passif : bascule complète du lit depuis la position assise à 45° pendant 1 minute
- Trendelenburg si DV : bascule du lit de + 13° à – 13°

Caractéristiques de l'épisode

Hypotension artérielle justifiant d'une intervention	☐ Oui	☐ Non
Tachycardie > 120 battements par minute	☐ Oui	☐ Non
Baisse de l'index cardiaque continu > 15%	☐ Oui	☐ Non
Apparition ou extension de marbrures	☐ Oui	☐ Non

Variables hémodynamiques

Fréquence cardiaque |_|_|_| bpm
Pression artérielle moyenne |_|_|_| mmHg
Index cardiaque continu |_|_|,|_| L/min/m^2
Pression veineuse centrale |_|_| mmHg

Epuration extra-rénale

Débit d'UF nette en cours au moment de l'épisode |_|_|_|_| ml/h

ICC **avant** manœuvre posturale (L/min/m^2) |_|_|,|_| L/min/m^2
ICC **après** manœuvre posturale (L/min/m^2) |_|_|,|_| L/min/m^2

*Positif si l'**index cardiaque continu** augmente de plus de 10% lors du lever de jambe passif, ou de plus de 8% lors du Trendelenburg*

Appliquer le protocole INSTABILITE

Profil : ☐ **JAUNE** ☐ **ROUGE**

Débit d'UF nette réglée à la suite de l'évaluation |_|_|_|_| ml/h

EPISODE D'INSTABILITE HEMODYNAMIQUE (de H0 à H72)

Date de la visite (jj/mm/aaaa) |_|_|/|_|_|/|_|_|_|_|
Heure survenue de l'épisode |_|_|:|_|_|
Numéro de l'épisode |_|_|_|

Rappels :

- Pas plus de 1 évaluation par heure
- **Pas de calibration ou de mesure des lactates en situation urgente**
- Vérifier position des capteurs, fixés au bras du patient (point phlébostatique)
- Rincer les lignes artérielle et de PVC (flush)
- PVC à mesurer en décubitus, à 0°, et en fin d'expiration
- Lever de jambe passif : bascule complète du lit depuis la position assise à 45° pendant 1 minute
- Trendelenburg si DV : bascule du lit de + 13° à – 13°

Caractéristiques de l'épisode

Hypotension artérielle justifiant d'une intervention	☐ Oui	☐ Non
Tachycardie > 120 battements par minute	☐ Oui	☐ Non
Baisse de l'index cardiaque continu > 15%	☐ Oui	☐ Non
Apparition ou extension de marbrures	☐ Oui	☐ Non

Variables hémodynamiques

Fréquence cardiaque	|_|_|_| bpm
Pression artérielle moyenne	|_|_|_| mmHg
Index cardiaque continu	|_|_|,|_| L/min/m²
Pression veineuse centrale	|_|_| mmHg

Epuration extra-rénale

Débit d'UF nette en cours au moment de l'épisode |_|_|_|_| ml/h

ICC **avant** manœuvre posturale (L/min/m^2) |_|_|,|_| L/min/m^2
ICC **après** manœuvre posturale (L/min/m^2) |_|_|,|_| L/min/m^2

*Positif si l'**index cardiaque continu** augmente de plus de 10% lors du lever de jambe passif, ou de plus de 8% lors du Trendelenburg*

Appliquer le protocole INSTABILITE

Profil : ☐ **JAUNE** ☐ **ROUGE**

Débit d'UF nette réglée à la suite de l'évaluation |_|_|_|_| ml/h
