# Supplementary material for: Fluid balance neutralization secured by hemodynamic monitoring versus protocolized standard of care in critically ill patients requiring continuous renal replacement therapy: study protocol of the GO NEUTRAL randomized controlled trial
Source: Trials. 2022 Sep 22;23:798. doi: 10.1186/s13063-022-06735-6 (PMC9494882; doi:10.1186/s13063-022-06735-6)
Supplement: Supplementary file 5 — Additional file 5: Supplemental material 5. Bedside case report form of the control group (French). [file 13063_2022_6735_MOESM5_ESM.pdf]

Etude GO-NEUTRAL

Cahier IDE

Version 1 du 09/06/2021

BRAS CONTRÔLE

Merci de ne pas jeter ce document

Centre de l'étude :
____

Identifiant du patient pour l'étude : |_|-|_|_|-|_|-|_| (lettre du centre, numéro d'inclusion, initiales)

Ce cahier contient 3 sections :

- **Section 1** : monitorage par 4h de H4 à H72
- **Section 2** : report des épisodes d'instabilité hémodynamiques de H0 à H72
- **Section 3** : report des épisodes d'insuffisance respiratoire aiguë de H0 à H72

Section 1

Monitorage par 4 heures

H4 à H24

TOUTES LES 4 HEURES

Date de la visite (jj/mm/aaaa)	|_|_|/|_|_|/|_|_|_|_|
Heure de la visite	|_|_|:|_|_|

Temps de la visite

J1	☒ H4	☐ H8	☐ H12	☐ H16	☐ H20	☐ **H24**
J2	☐ H28	☐ H32	☐ H36	☐ H40	☐ H44	☐ **H48**
J3	☐ H52	☐ H56	☐ H60	☐ H64	☐ H68	☐ **H72**

Rappels :

- Lactates artériels dans les 8 heures précédentes, selon indication du clinicien
- Vérifier position des capteurs, fixés au bras du patient (point phlébostatique)
- Rincer les lignes artérielle et de PVC (flush)
- PVC à mesurer en décubitus, à 0°, et en fin d'expiration
- **Calibration systématique du PiCCO** (3 injections de 15 ml de SSI froid)

Apparition ou extension de marbrures	☐ Oui ☐ Non
Fréquence cardiaque	|_|_|_| bpm
Pression artérielle moyenne	|_|_|_| mmHg
Index cardiaque calibré par thermodilution	|_|_|,|_| L/min/m^2
Pression veineuse centrale	|_|_| mmHg
Acide lactique artériel (dans les 8h précédentes)	|_|_|,|_| mmol/L
Débit d'UF nette en cours (mettre 0 si EER suspendue)	|_|_|_|_| ml/h

TOUTES LES 4 HEURES

Date de la visite (jj/mm/aaaa) |_|_|/|_|_|/|_|_|_|_|
Heure de la visite |_|_|:|_|_|

Temps de la visite

J1	☐ H4	☒ H8	☐ H12	☐ H16	☐ H20	☐ **H24**
J2	☐ H28	☐ H32	☐ H36	☐ H40	☐ H44	☐ **H48**
J3	☐ H52	☐ H56	☐ H60	☐ H64	☐ H68	☐ **H72**

Rappels :

- Lactates artériels dans les 8 heures précédentes, selon indication du clinicien
- Vérifier position des capteurs, fixés au bras du patient (point phlébostatique)
- Rincer les lignes artérielle et de PVC (flush)
- PVC à mesurer en décubitus, à 0°, et en fin d'expiration
- **Calibration systématique du PiCCO** (3 injections de 15 ml de SSI froid)

Apparition ou extension de marbrures ☐ Oui ☐ Non

Fréquence cardiaque |_|_|_| bpm
Pression artérielle moyenne |_|_|_| mmHg
Index cardiaque calibré par thermodilution |_|_|,|_| L/min/m^2
Pression veineuse centrale |_|_| mmHg

Acide lactique artériel (dans les 8h précédentes) |_|_|,|_| mmol/L

Débit d'UF nette en cours (mettre 0 si EER suspendue) |_|_|_|_| ml/h

TOUTES LES 4 HEURES

Date de la visite (jj/mm/aaaa) |_|_|/|_|_|/|_|_|_|_|
Heure de la visite |_|_|:|_|_|

Temps de la visite

J1	☐ H4	☐ H8	☒ H12	☐ H16	☐ H20	☐ **H24**
J2	☐ H28	☐ H32	☐ H36	☐ H40	☐ H44	☐ **H48**
J3	☐ H52	☐ H56	☐ H60	☐ H64	☐ H68	☐ **H72**

Rappels :

- Lactates artériels dans les 8 heures précédentes, selon indication du clinicien
- Vérifier position des capteurs, fixés au bras du patient (point phlébostatique)
- Rincer les lignes artérielle et de PVC (flush)
- PVC à mesurer en décubitus, à 0°, et en fin d'expiration
- **Calibration systématique du PiCCO** (3 injections de 15 ml de SSI froid)

Apparition ou extension de marbrures ☐ Oui ☐ Non

Fréquence cardiaque |_|_|_| bpm
Pression artérielle moyenne |_|_|_| mmHg
Index cardiaque calibré par thermodilution |_|_|,|_| L/min/m^2
Pression veineuse centrale |_|_| mmHg

Acide lactique artériel (dans les 8h précédentes) |_|_|,|_| mmol/L

Débit d'UF nette en cours (mettre 0 si EER suspendue) |_|_|_|_| ml/h

TOUTES LES 4 HEURES

Date de la visite (jj/mm/aaaa) |_|_|/|_|_|/|_|_|_|_|

Heure de la visite |_|_|:|_|_|

Temps de la visite

J1	☐ H4	☐ H8	☐ H12	☒ H16	☐ H20	☐ **H24**
J2	☐ H28	☐ H32	☐ H36	☐ H40	☐ H44	☐ **H48**
J3	☐ H52	☐ H56	☐ H60	☐ H64	☐ H68	☐ **H72**

Rappels :

- Lactates artériels dans les 8 heures précédentes, selon indication du clinicien
- Vérifier position des capteurs, fixés au bras du patient (point phlébostatique)
- Rincer les lignes artérielle et de PVC (flush)
- PVC à mesurer en décubitus, à 0°, et en fin d'expiration
- **Calibration systématique du PiCCO** (3 injections de 15 ml de SSI froid)

Apparition ou extension de marbrures ☐ Oui ☐ Non

Fréquence cardiaque |_|_|_| bpm

Pression artérielle moyenne |_|_|_| mmHg

Index cardiaque calibré par thermodilution |_|_|,|_| L/min/m^2

Pression veineuse centrale |_|_| mmHg

Acide lactique artériel (dans les 8h précédentes) |_|_|,|_| mmol/L

Débit d'UF nette en cours (mettre 0 si EER suspendue) |_|_|_|_| ml/h

TOUTES LES 4 HEURES

Date de la visite (jj/mm/aaaa)	|_|_|/|_|_|/|_|_|_|_|
Heure de la visite	|_|_|:|_|_|

Temps de la visite

J1	☐ H4	☐ H8	☐ H12	☐ H16	☒ H20	☐ **H24**
J2	☐ H28	☐ H32	☐ H36	☐ H40	☐ H44	☐ **H48**
J3	☐ H52	☐ H56	☐ H60	☐ H64	☐ H68	☐ **H72**

Rappels :

- Lactates artériels dans les 8 heures précédentes, selon indication du clinicien
- Vérifier position des capteurs, fixés au bras du patient (point phlébostatique)
- Rincer les lignes artérielle et de PVC (flush)
- PVC à mesurer en décubitus, à 0°, et en fin d'expiration
- **Calibration systématique du PiCCO** (3 injections de 15 ml de SSI froid)

Apparition ou extension de marbrures	☐ Oui ☐ Non
Fréquence cardiaque	|_|_|_| bpm
Pression artérielle moyenne	|_|_|_| mmHg
Index cardiaque calibré par thermodilution	|_|_|,|_| L/min/m^2
Pression veineuse centrale	|_|_| mmHg
Acide lactique artériel (dans les 8h précédentes)	|_|_|,|_| mmol/L
Débit d'UF nette en cours (mettre 0 si EER suspendue)	|_|_|_|_| ml/h

TOUTES LES 4 HEURES

Date de la visite (jj/mm/aaaa) |_|_|/|_|_|/|_|_|_|_|
Heure de la visite |_|_|:|_|_|

Temps de la visite

J1	☐ H4	☐ H8	☐ H12	☐ H16	☐ H20	☒ **H24**
J2	☐ H28	☐ H32	☐ H36	☐ H40	☐ H44	☐ **H48**
J3	☐ H52	☐ H56	☐ H60	☐ H64	☐ H68	☐ **H72**

Rappels :

- Lactates artériels dans les 8 heures précédentes, selon indication du clinicien
- Vérifier position des capteurs, fixés au bras du patient (point phlébostatique)
- Rincer les lignes artérielle et de PVC (flush)
- PVC à mesurer en décubitus, à 0°, et en fin d'expiration
- **Calibration systématique du PiCCO** (3 injections de 15 ml de SSI froid)

Apparition ou extension de marbrures	☐ Oui ☐ Non
Fréquence cardiaque	|_|_|_| bpm
Pression artérielle moyenne	|_|_|_| mmHg
Index cardiaque calibré par thermodilution	|_|_|,|_| L/min/m^2
Pression veineuse centrale	|_|_| mmHg
Acide lactique artériel (dans les 8h précédentes)	|_|_|,|_| mmol/L
Débit d'UF nette en cours (mettre 0 si EER suspendue)	|_|_|_|_| ml/h

H28 à H48

TOUTES LES 4 HEURES

Date de la visite (jj/mm/aaaa)	|_|_|/|_|_|/|_|_|_|_|
Heure de la visite	|_|_|:|_|_|

Temps de la visite

J1	☐ H4	☐ H8	☐ H12	☐ H16	☐ H20	☐ **H24**
J2	☒ H28	☐ H32	☐ H36	☐ H40	☐ H44	☐ **H48**
J3	☐ H52	☐ H56	☐ H60	☐ H64	☐ H68	☐ **H72**

Rappels :

- Lactates artériels dans les 8 heures précédentes, selon indication du clinicien
- Vérifier position des capteurs, fixés au bras du patient (point phlébostatique)
- Rincer les lignes artérielle et de PVC (flush)
- PVC à mesurer en décubitus, à 0°, et en fin d'expiration
- **Calibration systématique du PiCCO** (3 injections de 15 ml de SSI froid)

Apparition ou extension de marbrures	☐ Oui ☐ Non
Fréquence cardiaque	|_|_|_| bpm
Pression artérielle moyenne	|_|_|_| mmHg
Index cardiaque calibré par thermodilution	|_|_|,|_| L/min/m^2
Pression veineuse centrale	|_|_| mmHg
Acide lactique artériel (dans les 8h précédentes)	|_|_|,|_| mmol/L
Débit d'UF nette en cours (mettre 0 si EER suspendue)	|_|_|_|_| ml/h

TOUTES LES 4 HEURES

Date de la visite (jj/mm/aaaa) |_|_|/|_|_|/|_|_|_|_|
Heure de la visite |_|_|:|_|_|

Temps de la visite

J1	☐ H4	☐ H8	☐ H12	☐ H16	☐ H20	☐ **H24**
J2	☐ H28	☒ H32	☐ H36	☐ H40	☐ H44	☐ **H48**
J3	☐ H52	☐ H56	☐ H60	☐ H64	☐ H68	☐ **H72**

Rappels :

- Lactates artériels dans les 8 heures précédentes, selon indication du clinicien
- Vérifier position des capteurs, fixés au bras du patient (point phlébostatique)
- Rincer les lignes artérielle et de PVC (flush)
- PVC à mesurer en décubitus, à 0°, et en fin d'expiration
- **Calibration systématique du PiCCO** (3 injections de 15 ml de SSI froid)

Apparition ou extension de marbrures ☐ Oui ☐ Non

Fréquence cardiaque |_|_|_| bpm
Pression artérielle moyenne |_|_|_| mmHg
Index cardiaque calibré par thermodilution |_|_|,|_| L/min/m^2
Pression veineuse centrale |_|_| mmHg

Acide lactique artériel (dans les 8h précédentes) |_|_|,|_| mmol/L

Débit d'UF nette en cours (mettre 0 si EER suspendue) |_|_|_|_| ml/h

TOUTES LES 4 HEURES

Date de la visite (jj/mm/aaaa) |_|_|/|_|_|/|_|_|_|_|
Heure de la visite |_|_|:|_|_|

Temps de la visite

J1	☐ H4	☐ H8	☐ H12	☐ H16	☐ H20	☐ **H24**
J2	☐ H28	☐ H32	☒ H36	☐ H40	☐ H44	☐ **H48**
J3	☐ H52	☐ H56	☐ H60	☐ H64	☐ H68	☐ **H72**

Rappels :

- Lactates artériels dans les 8 heures précédentes, selon indication du clinicien
- Vérifier position des capteurs, fixés au bras du patient (point phlébostatique)
- Rincer les lignes artérielle et de PVC (flush)
- PVC à mesurer en décubitus, à 0°, et en fin d'expiration
- **Calibration systématique du PiCCO** (3 injections de 15 ml de SSI froid)

Apparition ou extension de marbrures ☐ Oui ☐ Non

Fréquence cardiaque |_|_|_| bpm
Pression artérielle moyenne |_|_|_| mmHg
Index cardiaque calibré par thermodilution |_|_|,|_| L/min/m^2
Pression veineuse centrale |_|_| mmHg

Acide lactique artériel (dans les 8h précédentes) |_|_|,|_| mmol/L

Débit d'UF nette en cours (mettre 0 si EER suspendue) |_|_|_|_| ml/h

TOUTES LES 4 HEURES

Date de la visite (jj/mm/aaaa)	|_|_|/|_|_|/|_|_|_|_|
Heure de la visite	|_|_|:|_|_|

Temps de la visite

J1	☐ H4	☐ H8	☐ H12	☐ H16	☐ H20	☐ **H24**
J2	☐ H28	☐ H32	☐ H36	☒ H40	☐ H44	☐ **H48**
J3	☐ H52	☐ H56	☐ H60	☐ H64	☐ H68	☐ **H72**

Rappels :

- Lactates artériels dans les 8 heures précédentes, selon indication du clinicien
- Vérifier position des capteurs, fixés au bras du patient (point phlébostatique)
- Rincer les lignes artérielle et de PVC (flush)
- PVC à mesurer en décubitus, à 0°, et en fin d'expiration
- **Calibration systématique du PiCCO** (3 injections de 15 ml de SSI froid)

Apparition ou extension de marbrures	☐ Oui	☐ Non

Fréquence cardiaque	|_|_|_| bpm
Pression artérielle moyenne	|_|_|_| mmHg
Index cardiaque calibré par thermodilution	|_|_|,|_| L/min/m^2
Pression veineuse centrale	|_|_| mmHg
Acide lactique artériel (dans les 8h précédentes)	|_|_|,|_| mmol/L
Débit d'UF nette en cours (mettre 0 si EER suspendue)	|_|_|_|_|_| ml/h

TOUTES LES 4 HEURES

Date de la visite (jj/mm/aaaa) |_|_|/|_|_|/|_|_|_|_|
Heure de la visite |_|_|:|_|_|

Temps de la visite

J1	☐ H4	☐ H8	☐ H12	☐ H16	☐ H20	☐ **H24**
J2	☐ H28	☐ H32	☐ H36	☐ H40	☒ H44	☐ **H48**
J3	☐ H52	☐ H56	☐ H60	☐ H64	☐ H68	☐ **H72**

Rappels :

- Lactates artériels dans les 8 heures précédentes, selon indication du clinicien
- Vérifier position des capteurs, fixés au bras du patient (point phlébostatique)
- Rincer les lignes artérielle et de PVC (flush)
- PVC à mesurer en décubitus, à 0°, et en fin d'expiration
- **Calibration systématique du PiCCO** (3 injections de 15 ml de SSI froid)

Apparition ou extension de marbrures ☐ Oui ☐ Non

Fréquence cardiaque |_|_|_| bpm
Pression artérielle moyenne |_|_|_| mmHg
Index cardiaque calibré par thermodilution |_|_|,|_| L/min/m^2
Pression veineuse centrale |_|_| mmHg

Acide lactique artériel (dans les 8h précédentes) |_|_|,|_| mmol/L

Débit d'UF nette en cours (mettre 0 si EER suspendue) |_|_|_|_| ml/h

TOUTES LES 4 HEURES

Date de la visite (jj/mm/aaaa) |_|_|/|_|_|/|_|_|_|_|
Heure de la visite |_|_|:|_|_|

Temps de la visite

J1	☐ H4	☐ H8	☐ H12	☐ H16	☐ H20	☐ **H24**
J2	☐ H28	☐ H32	☐ H36	☐ H40	☐ H44	☒ **H48**
J3	☐ H52	☐ H56	☐ H60	☐ H64	☐ H68	☐ **H72**

Rappels :

- Lactates artériels dans les 8 heures précédentes, selon indication du clinicien
- Vérifier position des capteurs, fixés au bras du patient (point phlébostatique)
- Rincer les lignes artérielle et de PVC (flush)
- PVC à mesurer en décubitus, à 0°, et en fin d'expiration
- **Calibration systématique du PiCCO** (3 injections de 15 ml de SSI froid)

Apparition ou extension de marbrures ☐ Oui ☐ Non

Fréquence cardiaque |_|_|_| bpm
Pression artérielle moyenne |_|_|_| mmHg
Index cardiaque calibré par thermodilution |_|_|,|_| L/min/m^2
Pression veineuse centrale |_|_| mmHg

Acide lactique artériel (dans les 8h précédentes) |_|_|,|_| mmol/L

Débit d'UF nette en cours (mettre 0 si EER suspendue) |_|_|_|_| ml/h

H52 à H72

TOUTES LES 4 HEURES

Date de la visite (jj/mm/aaaa) |_|_|/|_|_|/|_|_|_|_|
Heure de la visite |_|_|:|_|_|

Temps de la visite

J1	☐ H4	☐ H8	☐ H12	☐ H16	☐ H20	☐ **H24**
J2	☐ H28	☐ H32	☐ H36	☐ H40	☐ H44	☐ **H48**
J3	☒ H52	☐ H56	☐ H60	☐ H64	☐ H68	☐ **H72**

Rappels :

- Lactates artériels dans les 8 heures précédentes, selon indication du clinicien
- Vérifier position des capteurs, fixés au bras du patient (point phlébostatique)
- Rincer les lignes artérielle et de PVC (flush)
- PVC à mesurer en décubitus, à 0°, et en fin d'expiration
- **Calibration systématique du PiCCO** (3 injections de 15 ml de SSI froid)

Apparition ou extension de marbrures ☐ Oui ☐ Non

Fréquence cardiaque |_|_|_| bpm
Pression artérielle moyenne |_|_|_| mmHg
Index cardiaque calibré par thermodilution |_|_|,|_| L/min/m²
Pression veineuse centrale |_|_| mmHg

Acide lactique artériel (dans les 8h précédentes) |_|_|,|_| mmol/L

Débit d'UF nette en cours (mettre 0 si EER suspendue) |_|_|_|_| ml/h

TOUTES LES 4 HEURES

Date de la visite (jj/mm/aaaa) |_|_|/|_|_|/|_|_|_|_|
Heure de la visite |_|_|:|_|_|

Temps de la visite

J1	☐ H4	☐ H8	☐ H12	☐ H16	☐ H20	☐ **H24**
J2	☐ H28	☐ H32	☐ H36	☐ H40	☐ H44	☐ **H48**
J3	☐ H52	☒ H56	☐ H60	☐ H64	☐ H68	☐ **H72**

Rappels :

- Lactates artériels dans les 8 heures précédentes, selon indication du clinicien
- Vérifier position des capteurs, fixés au bras du patient (point phlébostatique)
- Rincer les lignes artérielle et de PVC (flush)
- PVC à mesurer en décubitus, à 0°, et en fin d'expiration
- **Calibration systématique du PiCCO** (3 injections de 15 ml de SSI froid)

Apparition ou extension de marbrures ☐ Oui ☐ Non

Fréquence cardiaque |_|_|_| bpm
Pression artérielle moyenne |_|_|_| mmHg
Index cardiaque calibré par thermodilution |_|_|,|_| L/min/m^2
Pression veineuse centrale |_|_| mmHg

Acide lactique artériel (dans les 8h précédentes) |_|_|,|_| mmol/L

Débit d'UF nette en cours (mettre 0 si EER suspendue) |_|_|_|_| ml/h

TOUTES LES 4 HEURES

Date de la visite (jj/mm/aaaa) |_|_|/|_|_|/|_|_|_|_|

Heure de la visite |_|_|:|_|_|

Temps de la visite

J1	☐ H4	☐ H8	☐ H12	☐ H16	☐ H20	☐ **H24**
J2	☐ H28	☐ H32	☐ H36	☐ H40	☐ H44	☐ **H48**
J3	☐ H52	☐ H56	☒ H60	☐ H64	☐ H68	☐ **H72**

Rappels :

- Lactates artériels dans les 8 heures précédentes, selon indication du clinicien
- Vérifier position des capteurs, fixés au bras du patient (point phlébostatique)
- Rincer les lignes artérielle et de PVC (flush)
- PVC à mesurer en décubitus, à 0°, et en fin d'expiration
- **Calibration systématique du PiCCO** (3 injections de 15 ml de SSI froid)

Apparition ou extension de marbrures ☐ Oui ☐ Non

Fréquence cardiaque |_|_|_| bpm

Pression artérielle moyenne |_|_|_| mmHg

Index cardiaque calibré par thermodilution |_|_|,|_| L/min/m^2

Pression veineuse centrale |_|_| mmHg

Acide lactique artériel (dans les 8h précédentes) |_|_|,|_| mmol/L

Débit d'UF nette en cours (mettre 0 si EER suspendue) |_|_|_|_| ml/h

TOUTES LES 4 HEURES

Date de la visite (jj/mm/aaaa) |_|_|/|_|_|/|_|_|_|_|

Heure de la visite |_|_|:|_|_|

Temps de la visite

J1	☐ H4	☐ H8	☐ H12	☐ H16	☐ H20	☐ **H24**
J2	☐ H28	☐ H32	☐ H36	☐ H40	☐ H44	☐ **H48**
J3	☐ H52	☐ H56	☐ H60	☒ H64	☐ H68	☐ **H72**

Rappels :

- Lactates artériels dans les 8 heures précédentes, selon indication du clinicien
- Vérifier position des capteurs, fixés au bras du patient (point phlébostatique)
- Rincer les lignes artérielle et de PVC (flush)
- PVC à mesurer en décubitus, à 0°, et en fin d'expiration
- **Calibration systématique du PiCCO** (3 injections de 15 ml de SSI froid)

Apparition ou extension de marbrures ☐ Oui ☐ Non

Fréquence cardiaque |_|_|_| bpm

Pression artérielle moyenne |_|_|_| mmHg

Index cardiaque calibré par thermodilution |_|_|,|_| L/min/m²

Pression veineuse centrale |_|_| mmHg

Acide lactique artériel (dans les 8h précédentes) |_|_|,|_| mmol/L

Débit d'UF nette en cours (mettre 0 si EER suspendue) |_|_|_|_| ml/h

TOUTES LES 4 HEURES

Date de la visite (jj/mm/aaaa)	|_|_|/|_|_|/|_|_|_|_|
Heure de la visite	|_|_|:|_|_|

Temps de la visite

J1	☐ H4	☐ H8	☐ H12	☐ H16	☐ H20	☐ **H24**
J2	☐ H28	☐ H32	☐ H36	☐ H40	☐ H44	☐ **H48**
J3	☐ H52	☐ H56	☐ H60	☐ H64	☒ H68	☐ **H72**

Rappels :

- Lactates artériels dans les 8 heures précédentes, selon indication du clinicien
- Vérifier position des capteurs, fixés au bras du patient (point phlébostatique)
- Rincer les lignes artérielle et de PVC (flush)
- PVC à mesurer en décubitus, à 0°, et en fin d'expiration
- **Calibration systématique du PiCCO** (3 injections de 15 ml de SSI froid)

Apparition ou extension de marbrures	☐ Oui ☐ Non
Fréquence cardiaque	|_|_|_| bpm
Pression artérielle moyenne	|_|_|_| mmHg
Index cardiaque calibré par thermodilution	|_|_|,|_| L/min/m²
Pression veineuse centrale	|_|_| mmHg
Acide lactique artériel (dans les 8h précédentes)	|_|_|,|_| mmol/L
Débit d'UF nette en cours (mettre 0 si EER suspendue)	|_|_|_|_| ml/h

TOUTES LES 4 HEURES

Date de la visite (jj/mm/aaaa) |_|_|/|_|_|/|_|_|_|_|
Heure de la visite |_|_|:|_|_|

Temps de la visite

J1	☐ H4	☐ H8	☐ H12	☐ H16	☐ H20	☐ **H24**
J2	☐ H28	☐ H32	☐ H36	☐ H40	☐ H44	☐ **H48**
J3	☐ H52	☐ H56	☐ H60	☐ H64	☐ H68	☒ **H72**

Rappels :

- Lactates artériels dans les 8 heures précédentes, selon indication du clinicien
- Vérifier position des capteurs, fixés au bras du patient (point phlébostatique)
- Rincer les lignes artérielle et de PVC (flush)
- PVC à mesurer en décubitus, à 0°, et en fin d'expiration
- **Calibration systématique du PiCCO** (3 injections de 15 ml de SSI froid)

Apparition ou extension de marbrures ☐ Oui ☐ Non

Fréquence cardiaque |_|_|_| bpm
Pression artérielle moyenne |_|_|_| mmHg
Index cardiaque calibré par thermodilution |_|_|,|_| L/min/m^2
Pression veineuse centrale |_|_| mmHg

Acide lactique artériel (dans les 8h précédentes) |_|_|,|_| mmol/L

Débit d'UF nette en cours (mettre 0 si EER suspendue) |_|_|_|_| ml/h

Section 2

Épisodes d’instabilité hémodynamique

EPISODE D'INSTABILITE HEMODYNAMIQUE
(de H0 à H72)

Date de la visite (jj/mm/aaaa)	|_|_|/|_|_|/|_|_|_|_|
Heure survenue de l'épisode	|_|_|:|_|_|
Numéro de l'épisode	|_|_|_|

Rappels :

- <u>Pas plus de 1 évaluation par heure</u>
- **Pas de calibration ou de mesure des lactates en situation urgente**
- Vérifier position des capteurs, fixés au bras du patient (point phlébostatique)
- Rincer les lignes artérielle et de PVC (flush)
- PVC à mesurer en décubitus, à 0°, et en fin d'expiration

Caractéristiques de l'épisode

Hypotension artérielle justifiant d'une intervention	☐ Oui	☐ Non
Tachycardie > 120 battements par minute	☐ Oui	☐ Non
Baisse de l'index cardiaque continu > 15%	☐ Oui	☐ Non
Apparition ou extension de marbrures	☐ Oui	☐ Non

Variables hémodynamiques

Fréquence cardiaque	|_|_|_| bpm
Pression artérielle moyenne	|_|_|_| mmHg
Index cardiaque continu	|_|_|,|_| L/min/m^2
Pression veineuse centrale	|_|_| mmHg

Epuration extra-rénale

Débit d'UF nette en cours au moment de l'épisode	|_|_|_|_| ml/h

EPISODE D'INSTABILITE HEMODYNAMIQUE
(de H0 à H72)

Date de la visite (jj/mm/aaaa)	|_|_|/|_|_|/|_|_|_|_|
Heure survenue de l'épisode	|_|_|:|_|_|
Numéro de l'épisode	|_|_|_|

Rappels :

- <u>Pas plus de 1 évaluation par heure</u>
- **Pas de calibration ou de mesure des lactates en situation urgente**
- Vérifier position des capteurs, fixés au bras du patient (point phlébostatique)
- Rincer les lignes artérielle et de PVC (flush)
- PVC à mesurer en décubitus, à 0°, et en fin d'expiration

Caractéristiques de l'épisode

Hypotension artérielle justifiant d'une intervention	☐ Oui	☐ Non
Tachycardie > 120 battements par minute	☐ Oui	☐ Non
Baisse de l'index cardiaque continu > 15%	☐ Oui	☐ Non
Apparition ou extension de marbrures	☐ Oui	☐ Non

Variables hémodynamiques

Fréquence cardiaque	|_|_|_| bpm
Pression artérielle moyenne	|_|_|_| mmHg
Index cardiaque continu	|_|_|,|_| L/min/m^2
Pression veineuse centrale	|_|_| mmHg

Epuration extra-rénale

Débit d'UF nette en cours au moment de l'épisode	|_|_|_|_| ml/h

EPISODE D'INSTABILITE HEMODYNAMIQUE
(de H0 à H72)

Date de la visite (jj/mm/aaaa)	|_|_|/|_|_|/|_|_|_|_|
Heure survenue de l'épisode	|_|_|:|_|_|
Numéro de l'épisode	|_|_|_|

Rappels :

- <u>Pas plus de 1 évaluation par heure</u>
- **Pas de calibration ou de mesure des lactates en situation urgente**
- Vérifier position des capteurs, fixés au bras du patient (point phlébostatique)
- Rincer les lignes artérielle et de PVC (flush)
- PVC à mesurer en décubitus, à 0°, et en fin d'expiration

Caractéristiques de l'épisode

Hypotension artérielle justifiant d'une intervention	☐ Oui	☐ Non
Tachycardie > 120 battements par minute	☐ Oui	☐ Non
Baisse de l'index cardiaque continu > 15%	☐ Oui	☐ Non
Apparition ou extension de marbrures	☐ Oui	☐ Non

Variables hémodynamiques

Fréquence cardiaque	|_|_|_| bpm
Pression artérielle moyenne	|_|_|_| mmHg
Index cardiaque continu	|_|_|,|_| L/min/m^2
Pression veineuse centrale	|_|_| mmHg

Epuration extra-rénale

Débit d'UF nette en cours au moment de l'épisode	|_|_|_|_| ml/h

EPISODE D'INSTABILITE HEMODYNAMIQUE (de H0 à H72)

Date de la visite (jj/mm/aaaa)	|_|_|/|_|_|/|_|_|_|_|
Heure survenue de l'épisode	|_|_|:|_|_|
Numéro de l'épisode	|_|_|_|

Rappels :

- <u>Pas plus de 1 évaluation par heure</u>
- **Pas de calibration ou de mesure des lactates en situation urgente**
- Vérifier position des capteurs, fixés au bras du patient (point phlébostatique)
- Rincer les lignes artérielle et de PVC (flush)
- PVC à mesurer en décubitus, à 0°, et en fin d'expiration

Caractéristiques de l'épisode

Hypotension artérielle justifiant d'une intervention	☐ Oui	☐ Non
Tachycardie > 120 battements par minute	☐ Oui	☐ Non
Baisse de l'index cardiaque continu > 15%	☐ Oui	☐ Non
Apparition ou extension de marbrures	☐ Oui	☐ Non

Variables hémodynamiques

Fréquence cardiaque	|_|_|_| bpm
Pression artérielle moyenne	|_|_|_| mmHg
Index cardiaque continu	|_|_|,|_| L/min/m^2
Pression veineuse centrale	|_|_| mmHg

Epuration extra-rénale

Débit d'UF nette en cours au moment de l'épisode	|_|_|_|_| ml/h

EPISODE D'INSTABILITE HEMODYNAMIQUE
(de H0 à H72)

Date de la visite (jj/mm/aaaa) |_|_|/|_|_|/|_|_|_|_|
Heure survenue de l'épisode |_|_|:|_|_|
Numéro de l'épisode |_|_|_|

Rappels :

- Pas plus de 1 évaluation par heure
- **Pas de calibration ou de mesure des lactates en situation urgente**
- Vérifier position des capteurs, fixés au bras du patient (point phlébostatique)
- Rincer les lignes artérielle et de PVC (flush)
- PVC à mesurer en décubitus, à 0°, et en fin d'expiration

Caractéristiques de l'épisode

Hypotension artérielle justifiant d'une intervention	☐ Oui	☐ Non
Tachycardie > 120 battements par minute	☐ Oui	☐ Non
Baisse de l'index cardiaque continu > 15%	☐ Oui	☐ Non
Apparition ou extension de marbrures	☐ Oui	☐ Non

Variables hémodynamiques

Fréquence cardiaque	|_|_|_| bpm
Pression artérielle moyenne	|_|_|_| mmHg
Index cardiaque continu	|_|_|,|_| L/min/m²
Pression veineuse centrale	|_|_| mmHg

Epuration extra-rénale

Débit d'UF nette en cours au moment de l'épisode	|_|_|_|_| ml/h

EPISODE D'INSTABILITE HEMODYNAMIQUE
(de H0 à H72)

Date de la visite (jj/mm/aaaa) |_|_|/|_|_|/|_|_|_|_|
Heure survenue de l'épisode |_|_|:|_|_|
Numéro de l'épisode |_|_|_|

Rappels :
- Pas plus de 1 évaluation par heure
- **Pas de calibration ou de mesure des lactates en situation urgente**
- Vérifier position des capteurs, fixés au bras du patient (point phlébostatique)
- Rincer les lignes artérielle et de PVC (flush)
- PVC à mesurer en décubitus, à 0°, et en fin d'expiration

Caractéristiques de l'épisode

Hypotension artérielle justifiant d'une intervention	☐ Oui	☐ Non
Tachycardie > 120 battements par minute	☐ Oui	☐ Non
Baisse de l'index cardiaque continu > 15%	☐ Oui	☐ Non
Apparition ou extension de marbrures	☐ Oui	☐ Non

Variables hémodynamiques

Fréquence cardiaque	|_|_|_| bpm
Pression artérielle moyenne	|_|_|_| mmHg
Index cardiaque continu	|_|_|,|_| L/min/m^2
Pression veineuse centrale	|_|_| mmHg

Epuration extra-rénale

Débit d'UF nette en cours au moment de l'épisode	|_|_|_|_| ml/h

EPISODE D'INSTABILITE HEMODYNAMIQUE (de H0 à H72)

Date de la visite (jj/mm/aaaa) |_|_|/|_|_|/|_|_|_|_|
Heure survenue de l'épisode |_|_|:|_|_|
Numéro de l'épisode |_|_|_|

Rappels :

- Pas plus de 1 évaluation par heure
- **Pas de calibration ou de mesure des lactates en situation urgente**
- Vérifier position des capteurs, fixés au bras du patient (point phlébostatique)
- Rincer les lignes artérielle et de PVC (flush)
- PVC à mesurer en décubitus, à 0°, et en fin d'expiration

Caractéristiques de l'épisode

Hypotension artérielle justifiant d'une intervention	☐ Oui	☐ Non
Tachycardie > 120 battements par minute	☐ Oui	☐ Non
Baisse de l'index cardiaque continu > 15%	☐ Oui	☐ Non
Apparition ou extension de marbrures	☐ Oui	☐ Non

Variables hémodynamiques

Fréquence cardiaque	|_|_|_| bpm
Pression artérielle moyenne	|_|_|_| mmHg
Index cardiaque continu	|_|_|,|_| L/min/m²
Pression veineuse centrale	|_|_| mmHg

Epuration extra-rénale

Débit d'UF nette en cours au moment de l'épisode |_|_|_|_| ml/h

EPISODE D'INSTABILITE HEMODYNAMIQUE
(de H0 à H72)

Date de la visite (jj/mm/aaaa)	|_|_|/|_|_|/|_|_|_|_|
Heure survenue de l'épisode	|_|_|:|_|_|
Numéro de l'épisode	|_|_|_|

Rappels :

- <u>Pas plus de 1 évaluation par heure</u>
- **Pas de calibration ou de mesure des lactates en situation urgente**
- Vérifier position des capteurs, fixés au bras du patient (point phlébostatique)
- Rincer les lignes artérielle et de PVC (flush)
- PVC à mesurer en décubitus, à 0°, et en fin d'expiration

Caractéristiques de l'épisode

Hypotension artérielle justifiant d'une intervention	☐ Oui	☐ Non
Tachycardie > 120 battements par minute	☐ Oui	☐ Non
Baisse de l'index cardiaque continu > 15%	☐ Oui	☐ Non
Apparition ou extension de marbrures	☐ Oui	☐ Non

Variables hémodynamiques

Fréquence cardiaque	|_|_|_| bpm
Pression artérielle moyenne	|_|_|_| mmHg
Index cardiaque continu	|_|_|,|_| L/min/m^2
Pression veineuse centrale	|_|_| mmHg

Epuration extra-rénale

Débit d'UF nette en cours au moment de l'épisode	|_|_|_|_| ml/h

Section 3

Épisodes d’insuffisance respiratoire aiguë

EPISODE D'INSUFFISANCE RESPIRATOIRE AIGUE PAR OEDEME HYDROSTATIQUE (OAP)
(de H0 à H72)

Date de la visite (jj/mm/aaaa)	|_|_|/|_|_|/|_|_|_|_|
Heure de la visite	|_|_|:|_|_|
Numéro de l'épisode	|_|_|_|

Rappel :
- L'UF nette réglée dans le groupe contrôle doit être entre 0 et 25 ml/h
- En cas de survenue d'une **insuffisance respiratoire aiguë par œdème pulmonaire**, elle peut être augmentée pendant 4 à 8 heures selon la décision du clinicien à des débits plus élevés.

Caractéristiques de l'épisode

Apparition rapide (<24h)	☐ Oui	☐ Non
Fréquence respiratoire > 25 /min	☐ Oui	☐ Non
Apparition ou extension d'une hypoxémie	☐ Oui	☐ Non
Infiltrats pulmonaire bilatéraux (lignes B écho ou radio)	☐ Oui	☐ Non

Critères échographiques (au moins 1) :
- onde E > 1.5 x A chez un patient de plus 65 ans
- TDE < 150 ms
- onde E > 1 m/s
- rapport E/E' > 12

☐ Oui ☐ Non

Débit d'UF nette réglée à la suite de l'évaluation	|_|_|_|_| ml/h

EPISODE D'INSUFFISANCE RESPIRATOIRE AIGUE PAR OEDEME HYDROSTATIQUE (OAP) (de H0 à H72)

Date de la visite (jj/mm/aaaa)	|_|_|/|_|_|/|_|_|_|_|
Heure de la visite	|_|_|:|_|_|
Numéro de l'épisode	|_|_|_|

Rappel :
- L'UF nette réglée dans le groupe contrôle doit être entre 0 et 25 ml/h
- En cas de survenue d'une **insuffisance respiratoire aiguë par œdème pulmonaire**, elle peut être augmentée pendant 4 à 8 heures selon la décision du clinicien à des débits plus élevés.

Caractéristiques de l'épisode

Apparition rapide (<24h)	☐ Oui	☐ Non
Fréquence respiratoire > 25 /min	☐ Oui	☐ Non
Apparition ou extension d'une hypoxémie	☐ Oui	☐ Non
Infiltrats pulmonaire bilatéraux (lignes B écho ou radio)	☐ Oui	☐ Non

Critères échographiques (au moins 1) :
- onde E > 1.5 x A chez un patient de plus 65 ans
- TDE < 150 ms
- onde E > 1 m/s
- rapport E/E' > 12

☐ Oui ☐ Non

Débit d'UF nette réglée à la suite de l'évaluation	|_|_|_|_| ml/h

EPISODE D'INSUFFISANCE RESPIRATOIRE AIGUE PAR OEDEME HYDROSTATIQUE (OAP)
(de H0 à H72)

Date de la visite (jj/mm/aaaa)	|_|_|/|_|_|/|_|_|_|_|
Heure de la visite	|_|_|:|_|_|
Numéro de l'épisode	|_|_|_|

Rappel :
- L'UF nette réglée dans le groupe contrôle doit être entre 0 et 25 ml/h
- En cas de survenue d'une **insuffisance respiratoire aiguë par œdème pulmonaire**, elle peut être augmentée pendant 4 à 8 heures selon la décision du clinicien à des débits plus élevés.

**Caractéristiques de l'épisode**		
Apparition rapide (<24h)	☐ Oui	☐ Non
Fréquence respiratoire > 25 /min	☐ Oui	☐ Non
Apparition ou extension d'une hypoxémie	☐ Oui	☐ Non
Infiltrats pulmonaire bilatéraux (lignes B écho ou radio)	☐ Oui	☐ Non
**Critères échographiques** (au moins 1) :<br>• onde E > 1.5 x A chez un patient de plus 65 ans<br>• TDE < 150 ms<br>• onde E > 1 m/s<br>• rapport E/E' > 12	☐ Oui	☐ Non

Débit d'UF nette réglée à la suite de l'évaluation	|_|_|_|_| ml/h

EPISODE D'INSUFFISANCE RESPIRATOIRE AIGUE PAR OEDEME HYDROSTATIQUE (OAP) (de H0 à H72)

Date de la visite (jj/mm/aaaa)	|_|_|/|_|_|/|_|_|_|_|
Heure de la visite	|_|_|:|_|_|
Numéro de l'épisode	|_|_|_|

Rappel :
- L'UF nette réglée dans le groupe contrôle doit être entre 0 et 25 ml/h
- En cas de survenue d'une **insuffisance respiratoire aiguë par œdème pulmonaire**, elle peut être augmentée pendant 4 à 8 heures selon la décision du clinicien à des débits plus élevés.

Caractéristiques de l'épisode

Apparition rapide (<24h)	☐ Oui	☐ Non
Fréquence respiratoire > 25 /min	☐ Oui	☐ Non
Apparition ou extension d'une hypoxémie	☐ Oui	☐ Non
Infiltrats pulmonaire bilatéraux (lignes B écho ou radio)	☐ Oui	☐ Non

Critères échographiques (au moins 1) :
- onde E > 1.5 x A chez un patient de plus 65 ans
- TDE < 150 ms
- onde E > 1 m/s
- rapport E/E' > 12

☐ Oui ☐ Non

Débit d'UF nette réglée à la suite de l'évaluation |_|_|_|_| ml/h
