# Supplementary material for: Fluid balance neutralization secured by hemodynamic monitoring versus protocolized standard of care in critically ill patients requiring continuous renal replacement therapy: study protocol of the GO NEUTRAL randomized controlled trial
Source: Trials. 2022 Sep 22;23:798. doi: 10.1186/s13063-022-06735-6 (PMC9494882; doi:10.1186/s13063-022-06735-6)
Supplement: Supplementary file 6 — Additional file 6: Supplemental material 6. Printed version of the electronic case report form (French). [file 13063_2022_6735_MOESM6_ESM.pdf]

Etude GO-NEUTRAL

Cahier d'observation de l'étude

Version 1 du 03/03/2021

Identifiant du centre : |_|

- Lyon : A
- Villefranche : B
- Clermont-Ferrand : C
- Montpellier : D

Numéro d'ordre d'inclusion du patient dans le centre : |_|_|

Première initiale du NOM du patient : |_|
Première initiale du PRENOM du patient : |_|

Identifiant du patient pour l'étude : |_|-|_|_|-|_|-|_|
(lettre du centre, numéro d'inclusion, initiales)

**1. Visite d'inclusion 1/2**			
Date de la visite (jj/mm/aaaa)	|_|_|/|_|_|/|_|_|_|_|		
Critères d'inclusion			
		Oui	Non
1.1	Patient majeur (âge > 18 ans) affilié à un régime de sécurité sociale	☐	☐
1.2	Traité par amines vasopressives (noradrénaline et/ou adrénaline) pour insuffisance circulatoire aiguë, sans critère de dose	☐	☐
1.3	Présentant une insuffisance rénale aiguë de grade 3 selon l'échelle de sévérité des recommandations internationales KDIGO	☐	☐
1.4	Traité par EER continue depuis moins de 24h, quel que soit le mode (hémofiltration, hémodialyse, ou hémodiafiltration)	☐	☐
1.5	Porteur d'un dispositif de monitorage continu du débit cardiaque calibré par thermodilution déjà en place au moment du screening.	☐	☐
**SI L'UNE DES REPONSES EST NON, LES DONNEES DU PATIENT NE SONT PAS RECUEILLIES**			

**2. Visite d'inclusion 2/2**			
Date de la visite (jj/mm/aaaa)		|_|_|/|_|_|/|_|_|_|_|	
Critères de non-inclusion			
		Oui	Non
2.1	Patient recevant une technique d'oxygénation extra-corporelle	☐	☐
2.2	Patient présentant une hémorragie active justifiant la transfusion de produits sanguins labiles	☐	☐
2.3	Patient sous dialyse chronique ou porteur d'un greffon rénal	☐	☐
2.4	Relai par hémodialyse intermittente prévu dans les 72 heures	☐	☐
2.5	Patient hospitalisé pour accident vasculaire cérébral de cause ischémique ou hémorragique compliqué d'un coma et sous ventilation mécanique	☐	☐
2.6	Hépatite fulminante, définie par la coexistence d'une agression hépatique aiguë, une encéphalopathie hépatique, un ictère, et d'une baisse du taux de prothrombine < 50%, apparue depuis moins de 15 jours	☐	☐
2.7	Evaluation impossible de la précharge dépendance par réalisation d'une manœuvre posturale (amputation ou immobilisation d'un ou des membres inférieurs, thrombose de la veine cave inférieure connue ou syndrome du compartiment abdominal connu). Le syndrome compartimental abdominal est défini par la coexistence d'une hyperpression intra-abdominale supérieure à 20 mmHg et d'au moins une défaillance d'organe survenant *de novo*.	☐	☐
2.8	Allaitement ou grossesse en cours, identifiée par un taux de béta-HCG positif chez la femme en âge de procréer, et réalisé juste avant l'inclusion	☐	☐
2.9	Limitations des thérapeutiques actives en place pour le patient, portant sur la ventilation mécanique ou la réanimation d'un arrêt cardio-respiratoire	☐	☐
2.10	Décès imminent	☐	☐
2.11	Patient sous tutelle ou curatelle, ou autre protection de justice	☐	☐
2.12	Inclusion dans une autre recherche interventionnelle dont le critère de jugement principal serait la balance hydrosodée ou dont l'intervention porte sur l'hémodynamique, l'EER, ou la modification de la balance entrées/sorties	☐	☐
2.13	Patient déjà inclus précédemment dans l'étude	☐	☐
**SI L'UNE DES REPONSES EST OUI, LES DONNEES DU PATIENT NE SONT PAS RECUEILLIES**			

3. Procédure de consentement			
		Oui	Non
3.1	Recueil du consentement du patient à participer à l'étude	☐	☐
3.1.1	Si oui, date du recueil du consentement (jj/mm/aaaa)	|_|_|/|_|_|/|_|_|_|_|	
3.2	Recueil du consentement du patient à continuer à participer	☐	☐
3.2.1	Si oui, date du recueil du consentement (jj/mm/aaaa)	|_|_|/|_|_|/|_|_|_|_|	
3.3	Recueil du consentement du représentant du patient à participer à l'étude	☐	☐
3.3.1	Si oui, date du recueil du consentement (jj/mm/aaaa)	|_|_|/|_|_|/|_|_|_|_|	
3.5	Inclusion selon procédure d'urgence	☐	☐
3.5.1	Si oui, date de la procédure d'urgence (jj/mm/aaaa)	|_|_|/|_|_|/|_|_|_|_|	

4. Inclusion et randomisation		
4.1	Stratification sur la surcharge hydrosodée	
4.1.1	Poids à l'admission dans le service de réanimation participant à l'étude (kg)	|_|_|_|
4.1.2	Poids mesuré le jour de l'inclusion (kg)	|_|_|_|
4.3	Randomisation	
4.3.1	Date de la randomisation (jj/mm/aaaa)	|_|_|/|_|_|/|_|_|_|_|
4.3.2	Heure de la randomisation (hh:mm)	|_|_|:|_|_|
4.3.3	Groupe de randomisation :	
	☐ Groupe contrôle	☐ Groupe interventionnel
4.4	Début de l'étude **(H0)**	
4.4.1	**Date du début de l'intervention (jj/mm/aaaa)**	|_|_|/|_|_|/|_|_|_|_|
4.4.2	**Heure du début de l'intervention (hh:mm)**	|_|_|:|_|_|

**5. Visite initiale (J1) 1/4**			
Date de la visite (jj/mm/aaaa)		|_|_|/|_|_|/|_|_|_|_|	
5.1	Date de naissance (mm/aaaa)	|_|_|/|_|_|_|_|	
5.2	Sexe	☐ F	☐ H
5.4	Taille (cm)	|_|_|_|	
5.5	Catégorie d'admission		
5.5.1	Médicale	☐	
5.5.2	Chirurgie urgente	☐	
5.5.3	Chirurgie programmée	☐	
5.6	Origine d'admission		
5.6.1	Service d'urgences ou SAMU	☐	
5.6.2	Service d'hospitalisation conventionnelle	☐	
5.6.3	Service de réanimation	☐	
5.7	Date d'admission dans le service de réanimation participant (jj/mm/aaaa)	|_|_|/|_|_|/|_|_|_|_|	
5.8	Date d'admission à l'hôpital (jj/mm/aaaa)	|_|_|/|_|_|/|_|_|_|_|	
5.9	Code diagnostic APACHE III	|_|_|_|_|	
5.10	Présence d'un sepsis lors de l'inclusion		
5.10.1	Infection prouvée ou suspectée	☐ Oui	☐ Non
5.10.2	Augmentation de +2 points du SOFA	☐ Oui	☐ Non
5.11	Score IGS-2	|_|_|_|	
5.12	Créatinine de base		
5.12.1	Créatinine de base disponible	☐ Oui	☐ Non
5.12.2	Si oui, valeur de la créatinine (µmol/L)	|_|_|_|_|	
5.13	Comorbidités		
5.13.1	Hypertension artérielle	☐ Oui	☐ Non
5.13.2	Diabète	☐ Oui	☐ Non
5.13.3	Artériopathie oblitérante des membres inférieurs	☐ Oui	☐ Non
5.13.4	Accident vasculaire cérébral (transitoire ou non)	☐ Oui	☐ Non
5.13.5	Insuffisance rénale chronique	☐ Oui	☐ Non
5.13.6	Insuffisance cardiaque chronique	☐ Oui	☐ Non
5.13.7	Insuffisance respiratoire chronique	☐ Oui	☐ Non
5.13.8	Cirrhose	☐ Oui	☐ Non
5.13.9	Immunodépression	☐ Oui	☐ Non

**6. Visite initiale (J1) 2/4**			
Date de la visite (jj/mm/aaaa)			|_|_|/|_|_|/|_|_|_|_|
6.3	Diurèse des 24h précédant l'inclusion (ml)		|_|_|_|_|_|
6.4	Epuration extra-rénale		
6.4.1	Date d'initiation de l'EER		|_|_|/|_|_|/|_|_|_|_|
6.4.2	Heure d'initiation de l'EER		|_|_|:|_|_|
6.4.3	Modalité d'EER à l'inclusion		
	☐ CVVH	☐ CVVD	☐ CVVHDF
6.4.4	Débit de dialysat ou de substitution à l'inclusion (ml/h)		|_|_|_|_|
6.4.7	Débit d'UF nette à l'inclusion (ml/h)		|_|_|_|_|

**6. Visite initiale (J1) 3/4**			
Date de la visite (jj/mm/aaaa)		|_|_|/|_|_|/|_|_|_|_|	
6.4	Variables hémodynamiques à l'inclusion		
6.4.1	Marbrures	☐ Oui	☐ Non
6.4.2	Fréquence cardiaque (bpm)	|_|_|_|	
6.4.3	Pression artérielle systolique (mm Hg)	|_|_|_|	
6.4.4	Pression artérielle diastolique (mm Hg)	|_|_|_|	
6.4.5	Pression artérielle moyenne (mm Hg)	|_|_|_|	
6.4.6	Index cardiaque par thermodilution (L/min/m^2)	|_|_|,|_|	
6.4.7	Pression veineuse centrale (mm Hg)	|_|_|	
6.4.8	Eau pulmonaire extra-vasculaire indexée (ml/kg)	|_|_|,|_|	
6.4.9	Perméabilité vasculaire pulmonaire indexée	|_|_|,|_|	
6.4.10	Variation du volume d'éjection systolique (%)	|_|_|	
6.4.11	Variation de la pression pulsée (%)	|_|_|	
	ICC avant manœuvre posturale (L/min/m^2)	|_|_|,|_|	
	ICC après manœuvre posturale (L/min/m^2)	|_|_|,|_|	
6.5	Variables respiratoires à l'inclusion		
6.5.1	Ventilation mécanique	☐ Oui	☐ Non
6.5.2	Si oui, VM invasive	☐ Oui	☐ Non
6.5.3	Si oui, FiO2 (%) lors des gaz du sang	|_|_|_|	
6.5.5	Si non, débit d'oxygène (L/min) lors des gaz du sang	|_|_|_|	
7.3	Gaz du sang à l'inclusion (max 4 heures avant inclusion)		
7.3.1	PaO2 (mm Hg)	|_|_|_|	
7.3.4	Acide lactique artériel (mmol/L)	|_|_|,|_|	

**7. Visite initiale (J1) 4/4**		
Date de la visite (jj/mm/aaaa)		|_|_|/|_|_|/|_|_|_|_|
7.1	Score SOFA à l'inclusion	
7.1.1	Créatininémie à l'inclusion (µmol/L)	|_|_|_|_|
7.1.2	Score de Glasgow (/15)	|_|_|
7.1.3	Bilirubine totale (µmol/L)	|_|_|_|
7.1.4	Taux de plaquettes (G/L)	|_|_|_|_|
7.4	Hémogramme à l'inclusion	
7.4.1	Hémoglobinémie (g/L)	|_|_|_|
7.5	Traitements en cours à l'inclusion (max 4 heures avant inclusion)	
7.5.1	Vasopresseurs	☐ Oui ☐ Non
7.5.1.1	Si oui, type de vasopresseur	
	☐ Noradrénaline ☐ Adrénaline ☐ Vasopressine ☐ Autre	
7.5.1.2	Si oui, dose de noradrénaline (µg/kg/min)	|_|_|,|_|
7.5.1.3	Si oui, dose d'adrénaline (µg/kg/min)	|_|_|,|_|
	Si oui, dose de vasopressine (UI/min)	|_|_|,|_|
	Si oui, type autre vasopresseur	☐ ephedrine<br>☐ phenylephrine<br>☐ dopamine
	Si oui, dose autre vasopresseur (µg/kg/min)	
7.5.2	Inotropes	☐ Oui ☐ Non
7.5.2.1	Si oui, type d'inotrope	
	☐ Dobutamine ☐ Autre	
7.5.2.2	Si oui, dose de dobutamine (µg/kg/min)	|_|_|,|_|
	Si oui, type autre inotrope	☐ milrinone<br>☐ levosimendan<br>☐ isoproterenol
	Si oui, dose autre inotrope	
7.5.3	Diurétiques	☐ Oui ☐ Non
7.5.3.1	Si oui, type de diurétique	
	☐ Furosémide ☐ Bumétamide ☐ Autre	
7.5.3.2	Si oui, dose de furosémide (mg/j)	|_|_|_|_|
	Si oui, dose de bumétamide (mg/j)	|_|_|_|
	Si oui, type autre diurétique	☐ thiazidique<br>☐ spironolactone<br>☐ amiloride
	Si oui, dose autre diurétique (mg/j)	|_|_|_|

**8. Données toutes les 4 heures (de H0 à H72)**						
Date de la visite (jj/mm/aaaa)					|_|_|/|_|_|/|_|_|_|_|	
Heure de la visite					|_|_|:|_|_|	
Temps de la visite						
J1	☐ H4	☐ H8	☐ H12	☐ H16	☐ H20	☐ **H24**
J2	☐ H28	☐ H32	☐ H36	☐ H40	☐ H44	☐ **H48**
J3	☐ H52	☐ H56	☐ H60	☐ H64	☐ H68	☐ **H72**

8.1	Variables hémodynamiques		
	Apparition ou extension de marbrures	☐ Oui	☐ Non
8.1.1	Fréquence cardiaque (bpm)	|_|_|_|	
8.1.2	Pression artérielle moyenne (mm Hg)	|_|_|_|	
8.1.3	Index cardiaque par thermodilution (L/min/m^2)	|_|_|,|_|	
8.1.4	Pression veineuse centrale (mm Hg)	|_|_|	
8.2	Variables biologiques		
8.2.1	Acide lactique artériel (dans les 8h précédentes) (mmol/L)	|_|_|,|_|	
8.3	Epuration extra-rénale		
8.3.1	Débit d'UF nette en cours (ml/h)	|_|_|_|_|	
8.7	Survenue d'un ou plusieurs épisode d'instabilité hémodynamique sur les 4 dernières heures	☐ Oui	☐ Non
	Si oui, nombre d'épisodes	|_|	
	**Uniquement si groupe Intervention**		
8.4	**Résultat de l'évaluation hémodynamique**		
8.4.1	Lettre d'identifiant du profil	|_|	
	ICC avant manœuvre posturale (L/min/m^2)	|_|_|,|_|	
	ICC après manœuvre posturale (L/min/m^2)	|_|_|,|_|	
	Profil F ou G ? :	☐ Oui	☐ Non
	IC par thermodilution avant remplissage (L/min/m^2)	|_|_|,|_|	
	IC par thermodilution après remplissage (L/min/m^2)	|_|_|,|_|	
8.4.2	Débit d'UF nette réglée à la suite de l'évaluation (ml/h)	|_|_|_|_|	
	**Uniquement si groupe contrôle**		
	Survenue d'un épisode d'insuffisance respiratoire aiguë par œdème pulmonaire hydrostatique sur les 4 dernières heures (OAP)	☐ Oui	☐ Non
	**Critère de jugement principal**		
8.5	Balance hydrosodée - entrées cumulée des 4 dernières heures		
8.5.1	Médicaments IV intermittents (ml)	|_|_|_|_|	
8.5.2	Médicaments IV en administration continue (ml)	|_|_|_|_|	
8.5.3	Remplissage vasculaire (ml)	|_|_|_|_|_|	
8.5.4	Transfusion de produits sanguins labiles (ml)	|_|_|_|_|_|	
8.5.5	Hydratation IV/Garde-veine (ml)	|_|_|_|_|	
8.5.6	Nutrition entérale ou parentérale (ml)	|_|_|_|_|	
8.6	Balance hydrosodée (CJP) – sorties cumulée des 4 dernières heures		
8.6.1	UF nette cumulée (ml)	|_|_|_|_|_|	
8.6.2	Diurèse cumulée (ml)	|_|_|_|_|_|	
8.6.3	Volume des drains/aspiration cumulée (ml)	|_|_|_|_|	
	**Évènements indésirables**		

	Poursuite visite réa	☐ Oui	☐ Non
	En cas d'EI, merci de compléter la fiche EI		

**9. Données complémentaires toutes les 24h de H0 à H72 (1/2)**		
Date de la visite (jj/mm/aaaa)	|_|_|/|_|_|/|_|_|_|_|	
Heure de la visite	|_|_|:|_|_|	
Temps de la visite		
☐ H24	☐ H48	☐ H72

0	Poids du jour (kg)		|_|_|_|	
0.1	Modalité d'EER			
	☐ CVVH	☐ CVVD	☐ CVVHDF	
0.2	Débit de dialysat ou de substitution (ml/h)		|_|_|_|_|	
	Durée de suspension de l'EER sur les 24 dernières heures			
	Suspension #1 : date et heure de début	|_|_|/|_|_|/|_|_|_|_| |_|_| :|_|_|		
	Suspension #1 : date et heure de fin	|_|_|/|_|_|/|_|_|_|_| |_|_| :|_|_|		
	Suspension #2 : date et heure de début	|_|_|/|_|_|/|_|_|_|_| |_|_| :|_|_|		
	Suspension #2 : date et heure de fin	|_|_|/|_|_|/|_|_|_|_| |_|_| :|_|_|		
	Suspension #3 : date et heure de début	|_|_|/|_|_|/|_|_|_|_| |_|_| :|_|_|		
	Suspension #3 : date et heure de fin	|_|_|/|_|_|/|_|_|_|_| |_|_| :|_|_|		
	Suspension #4 : date et heure de début	|_|_|/|_|_|/|_|_|_|_| |_|_| :|_|_|		
	Suspension #4 : date et heure de fin	|_|_|/|_|_|/|_|_|_|_| |_|_| :|_|_|		
1	Variables hémodynamiques			
1.2	Pression artérielle systolique (mm Hg)		|_|_|_|	
1.3	Pression artérielle diastolique (mm Hg)		|_|_|_|	
1.4	Eau pulmonaire extra-vasculaire indexée (ml/kg)		|_|_|,|_|	
1.5	Perméabilité vasculaire pulmonaire indexée		|_|_|,|_|	
1.6	Variation du volume d'éjection systolique (%)		|_|_|	
1.7	Variation de la pression pulsée (%)		|_|_|	
2	Variables respiratoires			
2.1	Ventilation mécanique		☐ Oui	☐ Non
2.2	Si oui, VM invasive		☐ Oui	☐ Non
2.3	Si oui, FiO2 (%) lors des gaz du sang		|_|_|_|	
2.5	Si non, débit d'oxygène (L/min) lors des gaz du sang		|_|_|_|	
7.3	Gaz du sang			
7.3.1	PaO2 (mm Hg)		|_|_|_|	
7.3.4	Acide lactique artériel (mmol/L)		|_|_|,|_|	

<table>
<tr><td colspan="5">10. Données complémentaires toutes les 24h de H0 à H72 (2/2)</td></tr>
<tr><td colspan="2">Date de la visite (jj/mm/aaaa)</td><td colspan="3">|_|_|/|_|_|/|_|_|_|_|</td></tr>
<tr><td colspan="2">Heure de la visite</td><td colspan="3">|_|_|:|_|_|</td></tr>
<tr><td colspan="5">Temps de la visite</td></tr>
<tr><td colspan="2">☐ H24</td><td>☐ H48</td><td colspan="2">☐ H72</td></tr>
<tr><td>3</td><td colspan="2">Score SOFA</td><td colspan="2"></td></tr>
<tr><td>3.1</td><td colspan="2">Créatininémie (µmol/L)</td><td colspan="2">|_|_|_|_|_|</td></tr>
<tr><td>3.2</td><td colspan="2">Score de Glasgow (/15)</td><td colspan="2">|_|_|</td></tr>
<tr><td>3.3</td><td colspan="2">Bilirubine totale (µmol/L)</td><td colspan="2">|_|_|_|_|</td></tr>
<tr><td>3.4</td><td colspan="2">Taux de plaquettes (G/L)</td><td colspan="2">|_|_|_|_|_|</td></tr>
<tr><td>6</td><td colspan="2">Hémogramme</td><td colspan="2"></td></tr>
<tr><td>6.1</td><td colspan="2">Hémoglobinémie (g/L)</td><td colspan="2">|_|_|_|_|</td></tr>
<tr><td>7</td><td colspan="2">Traitements en cours</td><td colspan="2"></td></tr>
<tr><td>7.5.1</td><td colspan="2">Vasopresseurs</td><td>☐ Oui</td><td>☐ Non</td></tr>
<tr><td>7.5.1.1</td><td colspan="2">Si oui, type de vasopresseur</td><td colspan="2"></td></tr>
<tr><td></td><td>☐ Noradrénaline</td><td>☐ Adrénaline</td><td>☐ Vasopressine</td><td>☐ Autre</td></tr>
<tr><td>7.5.1.2</td><td colspan="2">Si oui, dose de noradrénaline (µg/kg/min)</td><td colspan="2">|_|_|,|_|</td></tr>
<tr><td>7.5.1.3</td><td colspan="2">Si oui, dose d'adrénaline (µg/kg/min)</td><td colspan="2">|_|_|,|_|</td></tr>
<tr><td></td><td colspan="2">Si oui, dose de vasopressine (UI/min)</td><td colspan="2">|_|_|,|_|</td></tr>
<tr><td></td><td colspan="2">Si oui, type autre vasopresseur</td><td colspan="2">☐ ephedrine
☐ phenylephrine
☐ dopamine</td></tr>
<tr><td></td><td colspan="2">Si oui, dose autre vasopresseur (µg/kg/min)</td><td colspan="2"></td></tr>
<tr><td>7.5.2</td><td colspan="2">Inotropes</td><td>☐ Oui</td><td>☐ Non</td></tr>
<tr><td>7.5.2.1</td><td colspan="2">Si oui, type d'inotrope</td><td colspan="2"></td></tr>
<tr><td></td><td colspan="2">☐ Dobutamine</td><td colspan="2">☐ Autre</td></tr>
<tr><td>7.5.2.2</td><td colspan="2">Si oui, dose de dobutamine (µg/kg/min)</td><td colspan="2">|_|_|,|_|</td></tr>
<tr><td></td><td colspan="2">Si oui, type autre inotrope</td><td colspan="2">☐ milrinone
☐ levosimendan
☐ isoproterenol</td></tr>
<tr><td></td><td colspan="2">Si oui, dose autre inotrope</td><td colspan="2"></td></tr>
<tr><td>7.5.3</td><td colspan="2">Diurétiques</td><td>☐ Oui</td><td>☐ Non</td></tr>
<tr><td>7.5.3.1</td><td colspan="2">Si oui, type de diurétique</td><td colspan="2"></td></tr>
<tr><td></td><td>☐ Furosémide</td><td>☐ Bumétamide</td><td colspan="2">☐ Autre</td></tr>
<tr><td>7.5.3.2</td><td colspan="2">Si oui, dose de furosémide (mg/j)</td><td colspan="2">|_|_|_|_|_|</td></tr>
<tr><td></td><td colspan="2">Si oui, dose de bumétamide (mg/j)</td><td colspan="2">|_|_|_|</td></tr>
<tr><td></td><td colspan="2">Si oui, type autre diurétique</td><td colspan="2">☐ thiazidique
☐ spironolactone
☐ amiloride</td></tr>
<tr><td></td><td colspan="2">Si oui, dose autre diurétique (mg/j)</td><td colspan="2">|_|_|_|</td></tr>
<tr><td></td><td colspan="2"></td><td colspan="2"></td></tr>
<tr><td></td><td colspan="2">Statut vital à H72</td><td colspan="2"></td></tr>
<tr><td></td><td colspan="2">Patient décédé</td><td>☐ Oui</td><td>☐ Non</td></tr>
<tr><td></td><td colspan="2">Si oui, date du décès</td><td colspan="2">|_|_|/|_|_|/|_|_|_|_|</td></tr>
<tr><td></td><td colspan="2">Si oui, heure du décès</td><td colspan="2">|_|_|:|_|_|</td></tr>
<tr><td></td><td colspan="2">Statut investigationnel à H72</td><td colspan="2"></td></tr>
<tr><td></td><td colspan="2">Patient sorti d'étude avant H72</td><td>☐ Oui</td><td>☐ Non</td></tr>
<tr><td></td><td>Si oui, indication :</td><td colspan="3">☐ Interruption EER > 8 h</td></tr>
<tr><td></td><td></td><td colspan="3">☐ Retrait de consentement</td></tr>
<tr><td></td><td></td><td colspan="3">☐ Transfert vers une unité de participant pas à l'étude</td></tr>
<tr><td></td><td></td><td colspan="3">☐ Retrait permanent du monitorage hémodynamique</td></tr>
</table>

		☐ Contre-indication permanente aux manœuvres posturales	
		☐ AVC avec coma et VM	
		☐ Décès	
	**Évènements indésirables**		
	Survenue d’un événement indésirable	☐ Oui	☐ Non

<table>
<tr><td colspan="4">11. Episodes d’instabilité hémodynamique (de H0 à H72)</td></tr>
<tr><td colspan="2">Date de la visite (jj/mm/aaaa)</td><td colspan="2">|_|_|/|_|_|/|_|_|_|_|</td></tr>
<tr><td colspan="2">Heure de la visite</td><td colspan="2">|_|_|:|_|_|</td></tr>
<tr><td colspan="2">Numéro de l’épisode</td><td colspan="2">|_|_|_|</td></tr>
<tr><td></td><td></td><td colspan="2"></td></tr>
<tr><td>11.1</td><td>Caractéristiques de l’épisode</td><td colspan="2"></td></tr>
<tr><td>11.1.1</td><td>Hypotension artérielle justifiant d’une intervention</td><td>☐ Oui</td><td>☐ Non</td></tr>
<tr><td>11.1.2</td><td>Tachycardie > 120 battements par minute</td><td>☐ Oui</td><td>☐ Non</td></tr>
<tr><td>11.1.3</td><td>Baisse de l’index cardiaque continu > 15%</td><td>☐ Oui</td><td>☐ Non</td></tr>
<tr><td>11.1.4</td><td>Apparition ou extension de marbrures</td><td>☐ Oui</td><td>☐ Non</td></tr>
<tr><td>11.2</td><td>Variables hémodynamiques</td><td colspan="2"></td></tr>
<tr><td>11.2.1</td><td>Fréquence cardiaque (bpm)</td><td colspan="2">|_|_|_|</td></tr>
<tr><td>11.2.2</td><td>Pression artérielle moyenne (mm Hg)</td><td colspan="2">|_|_|_|</td></tr>
<tr><td>11.2.3</td><td>Index cardiaque continu (L/min/m^2)</td><td colspan="2">|_|_|,|_|</td></tr>
<tr><td>11.2.4</td><td>Pression veineuse centrale (mm Hg)</td><td colspan="2">|_|_|</td></tr>
<tr><td>11.4</td><td>Epuration extra-rénale</td><td colspan="2"></td></tr>
<tr><td>11.4.1</td><td>Débit d’UF nette (ml/h)</td><td colspan="2">|_|_|_|_|</td></tr>
<tr><td></td><td>Uniquement si groupe Intervention</td><td colspan="2"></td></tr>
<tr><td>11.5</td><td>Résultat de l’évaluation hémodynamique</td><td colspan="2"></td></tr>
<tr><td>11.5.1</td><td>☐ Profil jaune</td><td colspan="2">☐ Profil rouge</td></tr>
<tr><td></td><td>ICC avant manœuvre posturale (L/min/m^2)</td><td colspan="2">|_|_|,|_|</td></tr>
<tr><td></td><td>ICC après manœuvre posturale (L/min/m^2)</td><td colspan="2">|_|_|,|_|</td></tr>
<tr><td>11.5.2</td><td>Débit d’UF nette réglée à la suite de l’évaluation (ml/h)</td><td colspan="2">|_|_|_|_|</td></tr>
</table>

<table>
<tr><td colspan="4">12. Episodes d’insuffisance respiratoire aiguë par œdème pulmonaire hydrostatique (OAP) (de H0 à H72) [groupe Contrôle]</td></tr>
<tr><td colspan="2">Date de la visite (jj/mm/aaaa)</td><td colspan="2">|_|_|/|_|_|/|_|_|_|_|</td></tr>
<tr><td colspan="2">Heure de la visite</td><td colspan="2">|_|_|:|_|_|</td></tr>
<tr><td colspan="2">Numéro de l’épisode</td><td colspan="2">|_|_|_|</td></tr>
<tr><td></td><td colspan="3"></td></tr>
<tr><td></td><td colspan="3">Caractéristiques de l’épisode</td></tr>
<tr><td></td><td>Apparition rapide (<24h)</td><td>☐ Oui</td><td>☐ Non</td></tr>
<tr><td></td><td>Fréquence respiratoire > 25 /min</td><td>☐ Oui</td><td>☐ Non</td></tr>
<tr><td></td><td>Apparition ou extension d’une hypoxémie</td><td>☐ Oui</td><td>☐ Non</td></tr>
<tr><td></td><td>Infiltrats pulmonaire bilatéraux (écho ou radio)</td><td>☐ Oui</td><td>☐ Non</td></tr>
<tr><td></td><td>Critères échographiques</td><td>☐ Oui</td><td>☐ Non</td></tr>
<tr><td></td><td>Débit d’UF nette réglée à la suite de l’évaluation (ml/h)</td><td colspan="2">|_|_|_|_|</td></tr>
</table>

**12. Visite à J7 de l'inclusion (1/2)**			
Date de la visite (jj/mm/aaaa)		|_|_|/|_|_|/|_|_|_|_|	
	Poids du jour		
	Epuration extra-rénale toujours en cours (non sevrée)	☐ Oui	☐ Non
	Modalité d'EER en cours		
	☐ CVVH ☐ CVVD ☐ CVVHDF		
	Débit de dialysat ou de substitution (ml/h)	|_|_|_|_|	
	Débit d'UF nette en cours (ml/h)	|_|_|_|_|	
	Variables hémodynamiques		
	Fréquence cardiaque (bpm)	|_|_|_|	
	Pression artérielle systolique (mm Hg)	|_|_|_|	
	Pression artérielle diastolique (mm Hg)	|_|_|_|	
	Pression artérielle moyenne (mm Hg)	|_|_|_|	
	Monitorage continu du DC/PVC en cours	☐ Oui	☐ Non
	Index cardiaque par thermodilution (L/min/m^2)	|_|_|,|_|	
	Pression veineuse centrale (mm Hg)	|_|_|	
	Eau pulmonaire extra-vasculaire indexée (ml/kg)	|_|_|,|_|	
	Perméabilité vasculaire pulmonaire indexée	|_|_|,|_|	
	Variation du volume d'éjection systolique (%)	|_|_|	
	Variation de la pression pulsée (%)	|_|_|	
	ICC avant manœuvre posturale (L/min/m^2)	|_|_|,|_|	
	ICC après manœuvre posturale (L/min/m^2)	|_|_|,|_|	
	Variables respiratoires		
	Ventilation mécanique	☐ Oui	☐ Non
	Si oui, VM invasive	☐ Oui	☐ Non
	Si oui, FiO2 (%) lors des gaz du sang	|_|_|_|	
	Si non, débit d'oxygène (L/min) lors des gaz du sang	|_|_|_|	
7.3	Gaz du sang		
7.3.1	PaO2 (mm Hg)	|_|_|_|	
7.3.4	Acide lactique artériel (mmol/L)	|_|_|,|_|	

**12. Visite à J7 de l'inclusion (2/2)**				
Date de la visite (jj/mm/aaaa)		|_|_|/|_|_|/|_|_|_|_|		
	Score SOFA			
	Diurèse des 24h (ml, minuit à minuit)	|_|_|_|_|_|_|		
	Créatininémie à l'inclusion (µmol/L)	|_|_|_|_|_|		
	Score de Glasgow (/15)	|_|_|		
	Bilirubine totale (µmol/L)	|_|_|_|_|		
	Taux de plaquettes (G/L)	|_|_|_|_|_|		
	Hémogramme			
	Hémoglobinémie (g/L)	|_|_|_|_|		
	Traitements en cours			
7.5.1	Vasopresseurs	☐ Oui	☐ Non	
7.5.1.1	Si oui, type de vasopresseur			
	☐ Noradrénaline	☐ Adrénaline	☐ Vasopressine	☐ Autre
7.5.1.2	Si oui, dose de noradrénaline (µg/kg/min)	|_|_|,|_|		
7.5.1.3	Si oui, dose d'adrénaline (µg/kg/min)	|_|_|,|_|		
	Si oui, dose de vasopressine (UI/min)	|_|_|,|_|		
	Si oui, type autre vasopresseur	☐ ephedrine<br>☐ phenylephrine<br>☐ dopamine		
	Si oui, dose autre vasopresseur (µg/kg/min)			
7.5.2	Inotropes	☐ Oui	☐ Non	
7.5.2.1	Si oui, type d'inotrope			
	☐ Dobutamine	☐ Autre		
7.5.2.2	Si oui, dose de dobutamine (µg/kg/min)	|_|_|,|_|		
	Si oui, type autre inotrope	☐ milrinone<br>☐ levosimendan<br>☐ isoproterenol		
	Si oui, dose autre inotrope			
7.5.3	Diurétiques	☐ Oui	☐ Non	
7.5.3.1	Si oui, type de diurétique			
	☐ Furosémide	☐ Bumétamide	☐ Autre	
7.5.3.2	Si oui, dose de furosémide (mg/j)	|_|_|_|_|_|		
	Si oui, dose de bumétamide (mg/j)	|_|_|_|		
	Si oui, type autre diurétique	☐ thiazidique<br>☐ spironolactone<br>☐ amiloride		
	Si oui, dose autre diurétique (mg/j)	|_|_|_|		
	**Évènements indésirables**			
	Survenue d'un événement indésirable	☐ Oui	☐ Non	

**13. Sortie de réanimation et devenir**			
Date de la visite (jj/mm/aaaa)		|_|_|/|_|_|/|_|_|_|_|	
1	Sortie de réanimation		
1.1	Date de la sortie de réanimation	|_|_|/|_|_|/|_|_|_|_|	
1.2	Poids le jour de la sortie (kg)	|_|_|_|	
1.3	Créatininémie (µmol/L)	|_|_|_|_|	
1.4	Sevrage des supports d'organe		
1.4.1	Epuration extra-rénale sevrée définitivement avec succès en réanimation	☐ Oui	☐ Non
1.4.1.2	Si oui, date du sevrage	|_|_|/|_|_|/|_|_|_|_|	
	Patient intubé au cours du séjour	☐ Oui	☐ Non
	Date de l'intubation	|_|_|/|_|_|/|_|_|_|_|	
1.4.2	Extubation définitive réussie	☐ Oui	☐ Non
1.4.2.1	Si oui, date de l'extubation réussie	|_|_|/|_|_|/|_|_|_|_|	
1.4.4	Amines vasopressives sevrée définitivement avec succès (48h hors amines)	☐ Oui	☐ Non
1.4.4.1	Si oui, date du sevrage	|_|_|/|_|_|/|_|_|_|_|	
	J28		
	Patient décédé	☐ Oui	☐ Non
	Si oui, date du décès	|_|_|/|_|_|/|_|_|_|_|	
2	J90		
2.1	Date de la sortie de l'hôpital	|_|_|/|_|_|/|_|_|_|_|	
2.2	Epuration extra-rénale sevrée avec succès	☐ Oui	☐ Non
2.2.1	Si oui, date du sevrage	|_|_|/|_|_|/|_|_|_|_|	
2.3	Créatininémie à J90 si sevrage de l'épuration extra-rénale (µmol/L)	|_|_|_|_|	
2.4	Patient décédé	☐ Oui	☐ Non
2.4.1	Si oui, date du décès	|_|_|/|_|_|/|_|_|_|_|	
	**Évènements indésirables**		
	Survenue d'un événement indésirable	☐ Oui	☐ Non

<table>
<tr><td colspan="4">X. Evénement indésirable</td></tr>
<tr><td colspan="2">Date de la visite (jj/mm/aaaa)</td><td colspan="2">|_|_|/|_|_|/|_|_|_|_|</td></tr>
<tr><td colspan="2">Numéro de l’évènement</td><td colspan="2">|_|_|_|</td></tr>
<tr><td></td><td>Identifiant du participant</td><td colspan="2">|_|-|_|_|-|_|-|_|</td></tr>
<tr><td></td><td>Site participant (lettre d’identification)</td><td colspan="2">|_|</td></tr>
<tr><td></td><td>Date de naissance</td><td colspan="2">|_|_|/|_|_|_|_|</td></tr>
<tr><td></td><td>Sexe</td><td>☐ F</td><td>☐ M</td></tr>
<tr><td></td><td>Bras de randomisation</td><td colspan="2"></td></tr>
<tr><td></td><td>☐ Groupe contrôle</td><td colspan="2">☐ Groupe interventionnel</td></tr>
<tr><td></td><td>Antécédents (texte libre)</td><td></td><td></td></tr>
<tr><td></td><td>Date du début de l’évènement</td><td colspan="2">|_|_|/|_|_|/|_|_|_|_|</td></tr>
<tr><td></td><td>Date de fin de l’évenement</td><td colspan="2">|_|_|/|_|_|/|_|_|_|_|</td></tr>
<tr><td></td><td>Symptômes (texte libre)</td><td></td><td></td></tr>
<tr><td></td><td>Description de l’évènement (texte libre)</td><td></td><td></td></tr>
<tr><td></td><td>Grade d’intensité</td><td></td><td></td></tr>
<tr><td></td><td colspan="3">☐ 1 ☐ 2 ☐ 3 ☐ 4 ☐ 5</td></tr>
<tr><td></td><td>L’évènement est-il classifié comme étant grave (grade 3 ou 4) ?</td><td>☐ Oui</td><td>☐ Non</td></tr>
<tr><td></td><td>L’évènement est-il considéré comme étant possiblement en rapport avec l’intervention ?</td><td>☐ Oui</td><td>☐ Non</td></tr>
<tr><td></td><td>L’événement doit-il être signaler au promoteur (événement sévère [grade 3 ou plus] ou inattendu ou d’intérêt particulier)</td><td>☐ Oui</td><td>☐ Non</td></tr>
<tr><td></td><td>Action prise suite à l’EI quant à l’intervention :</td><td colspan="2">☐ poursuite
☐ ajustement (hors protocole)
☐ suspension transitoire
☐ suspension permanente</td></tr>
<tr><td></td><td>Si l’EI n’est pas en rapport avec l’intervention, est-il lié à :</td><td colspan="2">☐ un autre traitement
☐ une pathologie intercurrente
☐ un acte de soin
☐ la pathologie initiale</td></tr>
<tr><td></td><td>Conséquence de l’évènement</td><td colspan="2"></td></tr>
<tr><td></td><td>☐ Décès ☐ Récupéré avec séquelles</td><td colspan="2">☐ Récupéré sans séquelles ☐ En cours</td></tr>
<tr><td></td><td>Décès (date décès = date début évènement)</td><td>☐ Oui</td><td>☐ Non</td></tr>
<tr><td></td><td>Evénement grave non-fatal</td><td></td><td></td></tr>
<tr><td></td><td>Tout événement indésirable non listé par ailleurs, de grade 3 ou plus</td><td>☐ Oui</td><td>☐ Non</td></tr>
<tr><td></td><td>Choc hypovolémique non hémorragique de novo (baisse de l’index cardiaque avec précharge dépendance et lactates > 2 mmol/L)</td><td>☐ Oui</td><td>☐ Non</td></tr>
<tr><td></td><td>Arrêt cardiaque récupéré</td><td>☐ Oui</td><td>☐ Non</td></tr>
<tr><td></td><td>Ischémie myocardique de novo, avec élévation du segment ST > 1 mm dans 2 dérivations contiguës</td><td>☐ Oui</td><td>☐ Non</td></tr>
<tr><td></td><td>AVC ischémique de novo authentifié à l’imagerie</td><td>☐ Oui</td><td>☐ Non</td></tr>
<tr><td></td><td>Ischémie mésentérique de novo authentifiée à l’imagerie</td><td>☐ Oui</td><td>☐ Non</td></tr>
<tr><td></td><td>Ischémie artérielle aiguë de membre de novo authentifiée à l’imagerie</td><td>☐ Oui</td><td>☐ Non</td></tr>
<tr><td></td><td>Autre ischémie aiguë d’organe de novo, authentifiée à l’imagerie</td><td>☐ Oui</td><td>☐ Non</td></tr>
<tr><td></td><td>Traitements concomitants</td><td></td><td></td></tr>
</table>
