# Supplementary material for: Fluid balance neutralization secured by hemodynamic monitoring versus protocolized standard of care in critically ill patients requiring continuous renal replacement therapy: study protocol of the GO NEUTRAL randomized controlled trial
Source: Trials. 2022 Sep 22;23:798. doi: 10.1186/s13063-022-06735-6 (PMC9494882; doi:10.1186/s13063-022-06735-6)
Supplement: Supplementary file 7 — Additional file 7: Supplemental material 7. Funding declaration (English). [file 13063_2022_6735_MOESM7_ESM.pdf]

Hospices Civils de Lyon

votre santé,
notre engagement

CERTIFICATE

I, Alexandre PACHOT–Director of the Clinical Research and Innovation Department, certify that the research project GO NEUTRAL:

"Hemodynamic-guided Fluid Balance Neutralization During Continuous Renal Replacement Therapy in Critically Ill Patients: the GO NEUTRAL Randomized Controlled Multi-center Study"

carried out by Doctor Laurent BITKER is receiving financial support from the Ministry of Health within the framework of the hospital clinical research programm – PHRC I 2019. The total amount allocated is: 128 330,00€

Lyon, 9th August 2022, for all legal intents and purposes.

Director of Clinical Research and Innovation Department

Alexandre PACHOT

3, quai des Célestins – 69002 Lyon – France B.P. 2251 – 69229 Lyon cedex 02
N° FINESS HCL : 690781810 | **www.chu-lyon.fr**
