# Supplementary material for: Fluid balance neutralization secured by hemodynamic monitoring versus protocolized standard of care in critically ill patients requiring continuous renal replacement therapy: study protocol of the GO NEUTRAL randomized controlled trial
Source: Trials. 2022 Sep 22;23:798. doi: 10.1186/s13063-022-06735-6 (PMC9494882; doi:10.1186/s13063-022-06735-6)
Supplement: Supplementary file 8 — Additional file 8: Supplemental material 8. Approval letter by the human research ethics committee CPP Sud-Méditéranée I, sent on April 29, 2021 (French and English versions). [file 13063_2022_6735_MOESM8_ESM.pdf]

COMITE DE PROTECTION DES PERSONNES SUD MEDITERRANEE I

President
Professor Stéphane Ranque

Marseille, April 29th, 2021
Mr Alexandre Pachot
Hospices Civils de Lyon
3 quai des C2lestins
69229 Lyon Cedex 02

- Object: favorable recommendation
- CPP reference number: 21 38
- SI reference number: 21.03.24.45706
- Your reference: 69HCL20_1243
- Identification number: 2021-A00692-39

Sir,

We are pleased to send you our favorable recommendation regarding the research protocol on human subjects entitled:

"FLUID BALANCE NEUTRALIZATION SECURED BY HEMODYNAMIC MONITORING VERSUS PROTOCOLIZED STANDARD-OF-CARE IN CRITICALLY ILL PATIENTS REQUIRING CONTINUOUS RENAL REPLACEMENT THERAPY. STUDY PROTOCOL OF THE GO NEUTRAL RANDOMIZED CONTROLLED TRIAL"

Sponsored by the Hospices Civils de Lyon, and whose principal investigator is Dr Laurent BITKER

Among all dispositions of the Public Health Code that govern the relationships between the sponsor and the comity, which govern subsequent relations between the sponsor and the Committee, we draw your attention to the points:

- It is your responsibility, in the event that the competent authority issues an authorization on versions of documents different from those cited in the attached notice, to transmit these new versions for information to the Committee;

- This favorable recommendation relates only to the documents mentioned in the notice, and it will be up to you to contact the Committee for any substantial modification that you may have to consider;

- The Committee must receive notification of the effective start date of the research (signature of the consent form of the first person included in France);

- In the event of the absence of start of the research within a period of two years following the date of this notice, the latter would expire, unless an extension is granted by the Committee.

Please accept, Sir, the expression of my consideration.

Signature
Professor Stéphane Ranque

COMITE DE PROTECTION DES PERSONNES SUD MEDITERRANEE I

President
Professor Stéphane Ranque

NOTICE

The Comité de Protection des Personnes (human research ethics committee), Sud-Méditerrannée I, approved by ministerial decree dated June 1st, 2018, and constituted according to the decree of the Regional Prefect of the Provence Alpes Côte d'Azur region dated August 12, 2020,

in application of the Public Health Code and the regulations relating to the research mentioned in the first paragraph of the article L. 1121-1 of the Public Health Code not relating to a product mentioned in article L. 5311 -1 of the Public Health Code,

having been seized by a letter from the Hospices Civils de Lyon sponsor of a research file entitled:

"FLUID BALANCE NEUTRALIZATION SECURED BY HEMODYNAMIC MONITORING VERSUS PROTOCOLIZED STANDARD-OF-CARE IN CRITICALLY ILL PATIENTS REQUIRING CONTINUOUS RENAL REPLACEMENT THERAPY. STUDY PROTOCOL OF THE GO NEUTRAL RANDOMIZED CONTROLLED TRIAL"

Identified under the IDRCB number: 2021-A00692-39, and whose investigator (coordinator) is Dr Laurent BITKER,

having, after verification of compliance to regulations, registered this file on March 27, 2021 under the internal reference 21 38,

During its plenary session of April 14, 2021 during which A. BOYER CHAMMARD, O. DALMONT D. CHANAUD, P. HERQUEL N. ROATTINO, C. SIMEONE, P. DE ALCALA, T. BEGE, J-P BINON, J. KHOUANI, S. RANQUE

After hearing the rapporteur from the technical college, the rapporteur from the social college and the opinion of the methodologist deliberated,

The Committee has requested minor changes to the information notices, upon receipt of which it will issue a favorable recommendation.

On April 23, 2021, the Committee received the responses to its expectations and having examined the research file thus constituted:
• Referral letter dated and signed March 12, 2021
• Letter in response to the Committee's requests dated April 28, 2021
• Letter requesting a recommendation dated and signed on March 23, 2021
• Recommendation request form, dated and signed March 12, 2021
• Additional document to the recommendation request, dated and signed on March 3, 2021
• Protocol Version 1 of March 3, 2021 and appendix
• Protocol summary Version 1 of March 03, 2021
• Declaration of conformity to MR001 regulation

• Insurance certificate dated and signed March 11, 2021
• Patient information notice and consent to enrol form version no. 2 of April 20, 2021
• Next-of-kin information notice and consent to enrol form version no. 2 of April 20, 2021
• Patient information notice and consent to continue form version no. 2 of April 20, 2021
• “Emergent enrolment procedure” form version n°1 of March 03, 2021
• List of investigators version n°1 of March 3, 2021 and related CVs and GCPs
• Justification of the adequacy of the necessary human, material and technical resources required for the research project and compatible with the requirements of the people who will participate, dated March 12, 2021

issued a **FAVORABLE RECOMMENDATION**

to the start of this research considering that the conditions of validity of the research, in particular those defined in article L. 1123-7 of the Public Health Code, were met.

Signature
Professor Stéphane Ranque

COMITE DE PROTECTION DES PERSONNES SUD MÉDITERRANÉE I

Président
Professeur Stéphane RANQUE

Marseille, le 29 avril 2021

Monsieur Alexandre PACHOT
Hospices Civils de Lyon
3 quai des Célestins
69229 Lyon cedex 02

- Objet : Avis favorable
- Référence CPP : 21 38
- Référence SI : 21.03.24.45706
- Votre Référence : 69HCL20_1243
- Numéro d'identification : 2021-A00692-39

Monsieur

Nous avons le plaisir de vous faire parvenir ci-joint l'avis favorable relatif au protocole de recherche impliquant la personne humaine intitulé :

«NEUTRALISATION DE LA BALANCE HYDROSODEE GUIDEE PARL'HEMODYNAMIQUE FONCTIONNELLERNAU COURS DE L'EPURATION EXTRA-RENALE CONTINUE EN REANIMATION - ETUDE GO-NEUTRAL»

Promu par les Hospices Civils de Lyon et dont est M. le Dr Laurent BITKER l'investigateur coordonnateur.

Parmi l'ensemble des dispositions du Code de la Santé Publique qui régissent les relations ultérieures entre le promoteur et le Comité, nous attirons votre attention sur les points suivants :

- il vous appartient, dans le cas où l'autorité compétente délivrerait une autorisation sur des versions de documents différentes de celles qui sont citées dans l'avis ci-joint, de transmettre pour information au Comité ces nouvelles versions.
- le présent avis favorable ne porte que sur les documents mentionnés sur l'avis, et il vous appartiendra de saisir le Comité pour toute modification substantielle que vous seriez amené à envisager ;
- le Comité devra recevoir la notification de la date effective de commencement de la recherche (signature du consentement de la première personne incluse en France) ;
- en cas d'absence de début de la recherche dans le délai de deux ans après la date du présent avis favorable, celui-ci deviendrait caduque sauf prorogation accordée par le Comité

Je vous prie d'agréer, Monsieur, l'expression de ma considération.

Professeur Stéphane RANQUE

Hôpital Sainte Marguerite
270 Bd Sainte-Marguerite 13274 MARSEILLE Cedex 9
Tél. : 04.91.74.42.56 - Fax : 04.91.74.42.25 - E-mail : cppsudmed1@gmail.com
Site Internet : http://www.cppsudmediterranee1.fr

Président
Professeur Stéphane RANQUE

AVIS

Collège technique
Personnes qualifiées en recherche

Yves JAMMES
Aurélie MORAND (Pédiatre)
Stéphane RANQUE (méthodologiste)
Agnès BOYER CHAMARD

Marc GAINIER
Thierry BEGE
Jean GAUDART (méthodologiste)
Karim BENDIANE

Médecins généralistes

Jean Charles REYNIER
Jérémy KHOUANI

Pharmaciens hospitaliers
Nicole ROATTINO
Charléric BORNET

Infirmières
Dominique CHANAUD

Collège social
Personnes qualifiées en éthique
Christine ASSAÏANTE
Célia CHISCHPORTICH

Psychologues
Pierre DE ALCALA
Christian BOCCARDI

Travailleurs sociaux
Lucie CAMILLI

Juristes
Jean-Pierre BINON
Coraline SIMEONE
Mélissa DRIOUCHE

Représentants d'associations et usagers
Perrine HERQUEL
Odile DALMONT

Le Comité de Protection des Personnes Sud-Méditerranée I, agréé par arrêté ministériel en date du 1er juin 2018, constitué selon l'arrêté du Préfet de la Région Provence Alpes Côte d'Azur en date du 12 août 2020,

en application du code de la santé publique et de la réglementation en vigueur relative aux recherches mentionnées au 1°de l'article L. 1121-1 du code de la santé publique ne portant pas sur un produit mentionné à l'article L. 5311-1 du Code de la santé publique.

ayant été saisi par un courrier des Hospices Civils de Lyon promoteur d'un dossier de recherche intitulée :

«NEUTRALISATION DE LA BALANCE HYDROSODEE GUIDEE PARL'HEMODYNAMIQUE FONCTIONNELLERNAU COURS DE L'EPURATION EXTRA-RENALE CONTINUE EN REANIMATION -ETUDE GO-NEUTRAL»

Identifiée sous le numéro IDRCB : 2021-A00692-39 et dont l'investigateur (coordinateur) est M. le Dr Laurent BITKER

ayant, après vérification de la conformité réglementaire, enregistré ce dossier le **27 mars 2021** sous la référence interne **21 38**

Lors de sa séance plénière du **14 avril 2021** au cours de laquelle
Mesdames A. BOYER CHAMMARD, O. DALMONT D. CHANAUD, P. HERQUEL N. ROATTINO, C. SIMEONE
Messieurs : P. DE ALCALA, T. BEGE, J-P BINON, J. KHOUANI, S. RANQUE

Après avoir entendu le rapporteur du collège technique, le rapporteur du collège social et l'avis du méthodologiste ont délibéré,

Le Comité a demandé des modifications mineures des notices d'information à réception desquelles il délivrera un avis favorable.

Le Comité reçu le 23 avril 2021 les réponses conformes à ses attentes et ayant examiné le dossier de recherche ainsi constitué :

- Courrier de saisine daté et signé du 12 mars 2021
- Courrier de réponses aux demandes du Comité daté du 28 avril 2021
- Courrier de demande d'avis daté et signé du 23 mars 2021
- Formulaire de demande d'avis, daté et signé du 12 mars 2021
- Document additionnel à la demande d'avis, daté et signé du 03 mars 2021
- Protocole Version 1 du 03 mars 2021 et annexes
- Résumé Version 1 du 03 mars 2021
- Déclaration de conformité à la MR001
- Attestation d'assurance datée et signée du 11 mars 2021

- Notice d'information patient et formulaire de consentement version n° 2 du 20 avril 2021
- Notice d'information proche et formulaire de consentement version n° 2 du 20 avril 2021
- Notice d'information poursuite et formulaire de consentement version n° 2 du 20 avril 2021
- Consentement « procédure d'urgence » version n°1 du 03 mars 2021
- Liste des investigateurs version n°1 du 03 mars 2021 et CVs et BPC afférents
- Justification de l'adéquation des moyens humains, matériels et techniques nécessaires au projet de recherche et compatibles avec les impératifs des personnes qui s'y prêtent datée du 12 mars 2021

a émis un **AVIS FAVORABLE**

à la mise en œuvre de cette recherche considérant que les conditions de validité de la recherche, notamment celles définies dans l'article L. 1123-7 du code de la santé publique, étaient réunies.

Professeur Stéphane RANQUE
